# DNA Modification under Mild Conditions by Suzuki–Miyaura Cross-Coupling for the Generation of Functional Probes**

**DOI:** 10.1002/anie.201304038

**Published:** 2013-08-13

**Authors:** Lukas Lercher, Joanna F McGouran, Benedikt M Kessler, Christopher J Schofield, Benjamin G Davis

**Affiliations:** Department of Chemistry, University of Oxford, Chemistry Research LaboratoryMansfield Road, Oxford OX1 3TA (UK); Target Discovery Institute, Nuffield Department of Medicine, University of Oxford, Henry Wellcome Building for Molecular PhysiologyRoosevelt Drive, Oxford OX3 7FZ (UK)

**Keywords:** cross-coupling, DNA, oligonucleotides, palladium, photoaffinity labeling

The study of DNA, as the carrier of genetic information, is a cornerstone of modern biology. DNA hybridization determined by complementarity has allowed the design of many DNA-based functional structures.^1^ Such man-made systems include walkers,^2^ DNAzymes,^3^ and logic gates.^4^ While the structural scaffold of canonical nucleobases is sufficient for many purposes, additional functionality is often desirable. The development of modified building blocks with novel electronic,^5^ fluorogenic,^6^ and metal-complexing^7^ properties is an active research area.^8^

Multiple methods for the incorporation of modified building blocks with desired properties into oligodeoxynucleotides (ODNs) have been developed. Typically, these employ a “linear assembly” approach where a modified monomer is installed at an early stage, prior to oligomerization. Most common is the incorporation of the desired modified base into ODNs through solid-phase synthesis (SPS).^9^ This necessitates the synthesis of appropriate novel phosphoramidite building blocks, which are typically oxidation prone.^10^ Stability of the desired modification during the synthesis is a prerequisite. Even when these requirements are met, the incorporation of bulky building blocks can decrease the coupling efficiency.^11^

Modified bases can also be incorporated enzymatically by DNA polymerases;^12^ this circumvents some of the stability and synthesis issues associated with SPS and can allow high-density modification of nucleic acids. However, site-selective incorporation of a single modified base is difficult when canonical base pairs are used, requiring a multi-step procedure.^13^ Base modifications in the major groove (5-position for pyrimidines, 7- or 8-position for purines) are more suited for enzymatic incorporation than minor-groove modifications;^14^ still the introduction of some substituents in the major groove can be difficult,^15^ although this can sometimes be circumvented by the use of long linkers.^16^ The use of nonnatural base pairs does allow the site-specific enzymatic incorporation of some modifications,^17^ but requires the synthesis of a nonnatural substrate ODN and the appropriate triphosphate. Although this is an elegant approach, when studying properties of natural DNA, the incorporation of artificial bases at all stages might not be desirable.

A third approach is site-selective post-synthetic modification of ODNs. Such a “convergent assembly” strategy is likely to be more efficient and has the powerful strategic potential for late-stage diversification. In this approach, a chemical tag is incorporated into the ODN by SPS or by enzymatic methods and subsequently modified in a second step. Current commonly used modification methods for this are Cu-catalyzed^18^ or Cu-free Huisgen–Dimroth triazole-forming reactions,^19^ Diels–Alder cycloaddition,^20^ hydrazone formation,^21^ reductive amination,^22^ Staudinger ligation,^23^ nucleophilic displacement,^24^ native chemical ligation,^25^ thiol–maleimide conjugation,^26^ or amide bond formation.^27^ While these conjugation reactions can be efficient, often a large moiety is retained from the reaction (e.g. triazolyl- or norbonenyl-), which might interfere with biological function. Further, not all of the handles, tags, or building blocks required for conjugation are readily available and SPS might therefore still be required, thereby undermining key strategic advantages. Here, we report a post-oligomerization strategy based on Suzuki–Miyaura cross-coupling under biologically benign conditions (37 °C, pH 8.5). This chemistry allows direct substitution at the nucleobase core, thereby greatly increasing the scope of functionality that can be introduced.

During the course of our investigations, initial post-synthetic modifications of ODNs by a Suzuki–Miyaura cross-coupling were reported.^28^ Omumi et al.28a reported the cross-coupling of 8-bromo-2′-deoxyguanosine (8-BrdG) to synthesize aryl-substituted guanines. The resulting aryl-Gs are known DNA “damage products” that induce significant destabilization of the double helix; this property is of biological interest but not desirable for technological applications.

Whilst these methods generally afforded good yields, they required elevated temperatures; prolonged reaction times; and inert conditions outside of the scope of our goals. Cahová et al.28b described cross-coupling to 5-iodo-2′-deoxyuridine (5-IdU) at 120 °C under inert conditions and in alkaline aqueous solution; yields, however, were variable and substantial quantities of deiodinated ODNs were isolated. Herein, we report that under optimized reaction conditions we obtain excellent yields with a broad range of substrates under mild conditions (≤37 °C) with minimal deiodination. In reported studies on Suzuki–Miyaura cross-coupling on proteins, we have disclosed 2-aminopyrimidine-4,6-diol (L1) as a particularly efficient catalyst ligand under buffered aqueous conditions and at biologically benign temperatures (37 °C).^29^ We show that both L1 and its *N*,*N*-dimethylated analogue L2 can catalyze Suzuki–Miyaura cross-coupling of a variety of boronic esters to unprotected nucleosides and ODNs. We propose the Suzuki–Miyaura cross-coupling of commercially available halogenated ODNs as a general convergent synthetic strategy to readily generate modified ODNs (Figure 1). We were able to generate DNA bearing multiple modification types, including sensitive reporter modifications (e.g., photocrosslinkers) combined with sensitive natural modifications (e.g., 5-hydroxymethylcytosine (hmC)) as probes of DNA–protein interactions.

**Figure 1:**
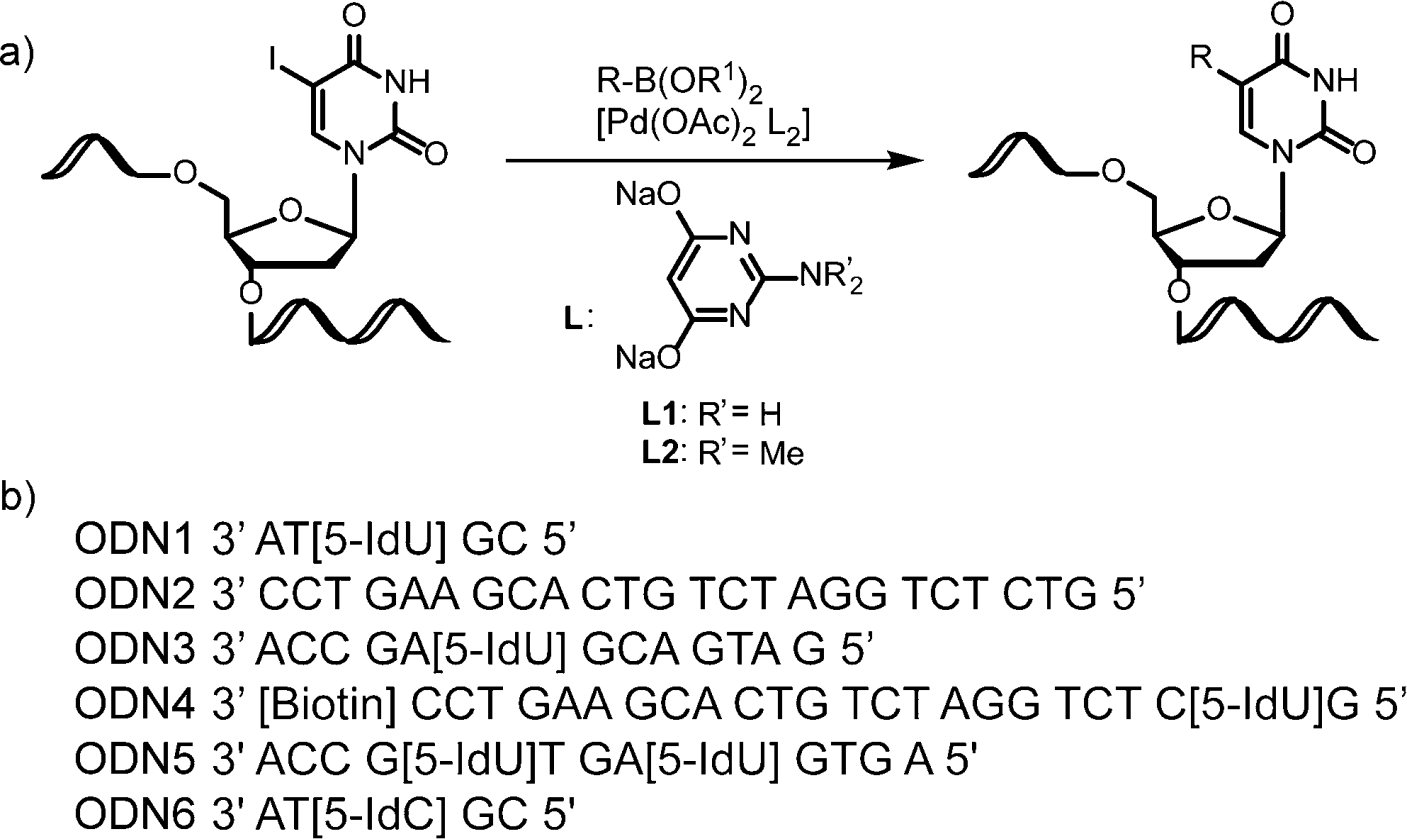
a) General reaction scheme for the Suzuki–Miyaura cross-coupling to 5-IdU in DNA. R^1^=H or pinacol ester. b) ODNs used in this work.

Firstly, we examined the cross-coupling of unprotected 5-iododeoxyuridine (5-IdU) and phenyl boronic acid, to exclude unproductive complexation of the Pd catalyst by the nucleobase. This proved immediately efficient, and when using commonly employed cross-coupling conditions (3 equiv base, 50 °C) we observed full conversion and 80 % yield of isolated product using only 5 mol % Pd catalyst (Figure 2).

**Figure 2:**
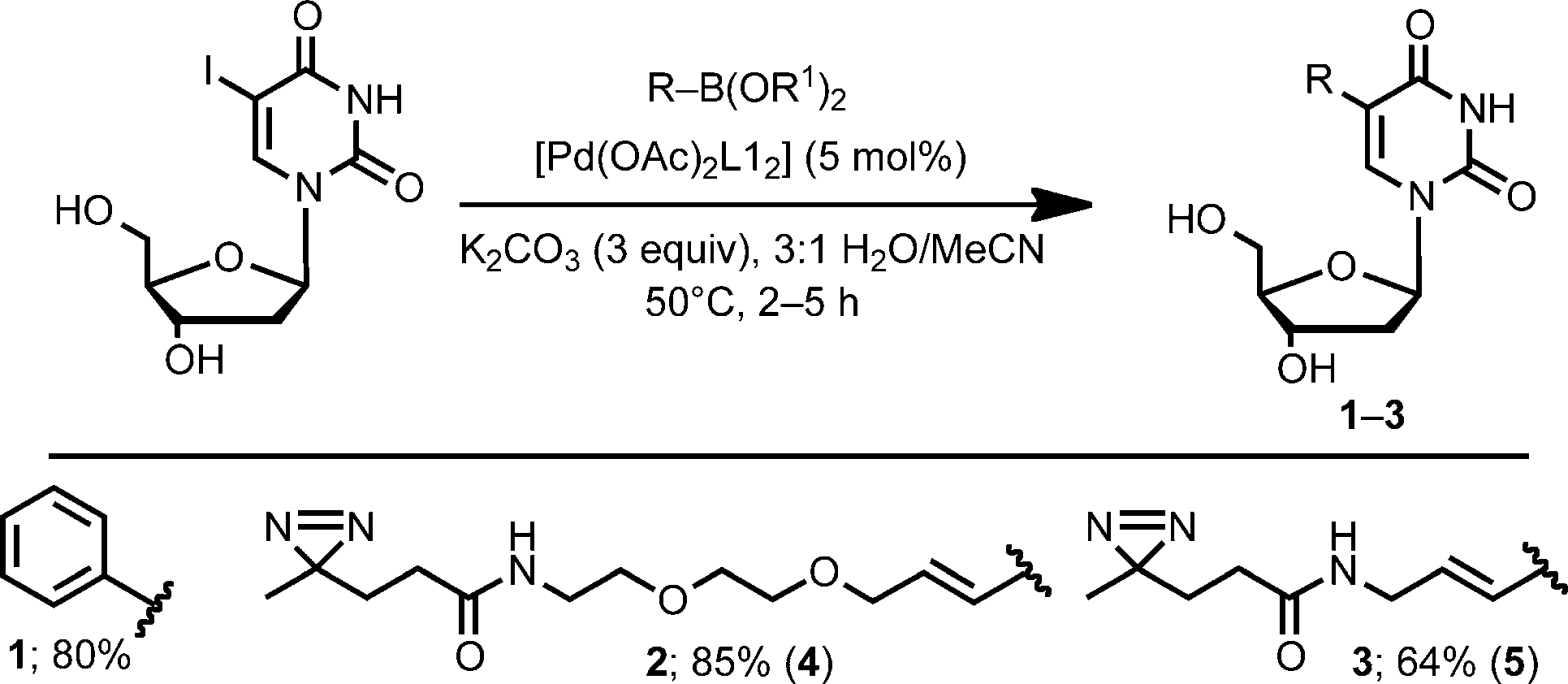
Suzuki–Miyaura cross-coupling with unprotected 5-IdU using phenylboronic acid and boronic esters **4**, **5** (esters in brackets). At the bottom the corresponding R groups and the yields of isolated products **1**–**3** are shown.

We refrained from optimizing the conditions for the monobase transformation, since many catalytic systems for the cross-coupling to single nucleosides are reported.^22^ We were interested in investigating the utility of the mild cross-coupling conditions and their tolerance towards representative “probe” functional groups used in DNA-based applications. Aliphatic diazirines are commonly-used photocrosslinkers to probe DNA–DNA and DNA–protein interactions;^30^ these crosslinkers have advantages over bulkier aromatic diazirines.^31^ Appropriate pinacol esters of boronic acids were constructed using a divergent synthetic route (see the Supporting Information, Figure S1). By using the alkene boronic ester reagents **4** and **5** we introduced photocrosslinkers with different linker lengths into single nucleosides (**2** and **3** respectively) with good yields. Importantly, under these mild conditions, no decomposition (thermal or photochemical) of the diazirine moiety was observed.

Encouraged by these results, we turned towards a 5-mer ODN containing all canonical bases and 5-IdU (ODN1) as a substrate (Figure 1 b and Table 1). We chose this substrate, because it would enable us to identify putative side reactions with different nucleobases. The 5-IdU residue was placed centrally to mimic the cross-coupling with longer ODNs. The reaction was monitored by HPLC and MALDI. When using conditions similar to those developed for protein modification^29^, ^32^ (50 mm Na_2_PO_4_, pH 8.0, 500 equiv R-B(OR^1^)_2_, 10 equiv [Pd(OAc_2_)L1_2_]) we observed successful product formation, which was accompanied by up to 22 % unwanted deiodinated side product. We systematically examined different biologically compatible buffer systems and a range of basic pH values (Table 1). Of these, we found that tris(hydroxymethyl)aminomethane (TRIS) buffer at pH 8.5 provided the most robust cross-coupling conditions. Full conversion of the starting material with no noticeable side products, except for trace deiodinated ODN was observed.

**Table 1:** Optimization of the reaction with boronic ester 5 and ODN1^[a]^

5(equiv)	Pd (equiv)	*T*	Buffer (pH)	*t* [h]	S [%]^[a]^	-I [%]^[b]^	P [%]^[c]^
500	10	37 °C	PO_4_ (8.0)	5	0	22	78
500	5	37 °C	PO_4_ (8.5)	5	0	19	81
500	5	37 °C	NH_4_OAc (8.0)	5	0	16	84
500	5	37 °C	TRIS (8.5)	5	0	5	95
100	0.5	37 °C	TRIS (8.5)	4	0	0	95
100	0.5	RT	TRIS (8.5)	4	51	0	49
100	0.5	RT	TRIS (8.5)	16	0	0	95
100	0.1	37 °C	TRIS (8.5)	4	72	2	26
100	0.1	37 °C	TRIS (8.5)	16	10	3	87

[a] S=recovered substrate ODN1. [b] -I=deiodinated substrate. [c] Product yield determined by relative integration in HPLC at 260 nm.

This effect of buffer is striking. One current mechanistic proposal for the Suzuki reaction involves a transmetalation step from the neutral boronic acid to an arylpalladium hydroxo complex.^33^ Hartwig and Carrow^34^ have shown that neutral boronic esters can undergo transmetalation with a Ar–Pd–OH complex without addition of base. Under their reaction conditions the transmetalation of the neopentyl glycol boronic ester is significantly faster than that of the pinacol ester (>2 min versus 1.5 h). The strong effect of TRIS may reflect an active role in the catalytic cycle plaued by formation of a more active boronic ester, or alternatively, it could facilitate hydrolysis.

Further optimization revealed that complete conversion was reached after 4 h at 37 °C using 100 equivalents of boronic ester and 50 mol % [Pd]. Palladium loading could even be reduced to 10 mol % over longer reaction times. The reaction proceeds at room temperature, albeit more slowly (ca. 50 % after 4 h), with full conversion after 16 h. These even milder conditions may be beneficial when utilizing other, even more thermally sensitive groups. Control reactions without boronic ester or palladium yielded no product (see the Supporting Information). A 21-mer (ODN2) that did not contain 5-IdU was subjected to the reaction conditions; no reaction was observed (see the Supporting Information), thus ruling out nonspecific reaction (e.g. C-H activation) of other bases and confirming site selectivity.

We then investigated the modification substrate scope of the reaction. To demonstrate utility we generated modified ODNs containing representative functionalities commonly used in DNA. Diazirine-containing boronic esters with both shorter and longer linkers were coupled in excellent yields (>90 %, ODN1d, ODN1f; Figure 3). Another often-used photocrosslinker moiety, benzophenone, was also readily installed in excellent yield (86 %) under these conditions to generate ODN1a. Azobenzene derivatives are used as photoswitches,^35^ and azobenzene variant ODN1b was generated in good (71 %) yield. Substitution of the azobenzene core often has substantial influence on the photochemical properties of the moiety;^35^ the convergent strategy demonstrated here enables rapid screening of such properties directly in the ODN, without the need for redesigned phosphoramidite synthesis and associated SPS. The sequence and spatially defined presentation of sugars on various oligomers has been elegantly exploited as a tool to investigate the effect of ligand display in binding to cognate protein receptors, such as lectins.^36^ Moreover, ODN glycosylation has been shown to stimulate and enhance cellular uptake.^37^ When using the appropriate unprotected vinyl boronic glucosyl pinacol ester **8**, D-glucosylation was readily achieved in the major groove of ODN1 to generate the glucosyl-ODN ODN1c. The reaction with **8** proceeded more slowly than other examples, requiring extended reaction times for good yield, presumably due to increased steric bulk. The pyrene-substituted boronic ester reagent **10** behaved similarly and required extended reaction times for good conversion, which is again presumably due to steric bulk but nonetheless gave a useful ultimate yield. Uridine residues substituted with various 5-membered heterocycles have been used as fluorescent reporters in nucleic acids.^38^, ^39^ During attempts to use 3-furanyl boronic acid (**11**) as a cross-coupling partner in reactions with L1, we observed full conversion but obtained only 30 % product formation and 70 % deiodinated starting material. Switching from L1 to the *N*,*N*-dimethyl derivative L2 and from the furan boronic acid to its pinacol ester **9** substantially increases the yield (78 %), thus demonstrating the installation of a seventh modification type in the creation of ODN1e and the need for reaction-specific optimization. Many of the functional groups introduced here may potentially have been introduced with other methods on a case-by-case basis, but the widespread compatibility shown here we believe will be useful. Moreover, direct substitution at the 5-position on the pyrimidine core is not readily achieved with other methodologies.

**Figure 3:**
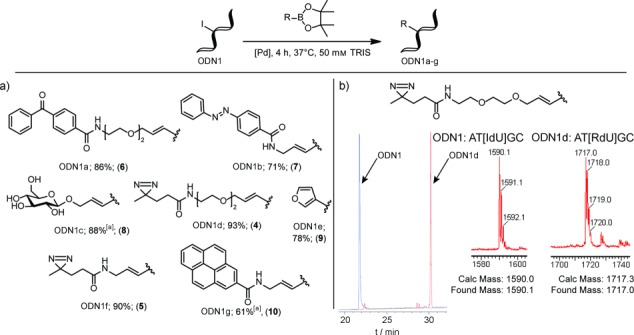
Suzuki–Miyaura cross-coupling of ODN1 with boronic esters **4**-**10** (also see the Supporting Information, Figure S1). a) The corresponding R groups and the yields of isolated products are shown. The boronic esters are given in brackets. [a] Reaction was shaken at RT for 16 h. b) Representative HPLC and MS analysis of the reaction of **4** with ODN1.

Interestingly, when analyzing the cross-coupling of **9** with ODN1, we noticed another significant peak in the HPLC chromatogram (see the Supporting Information). Isolation and MALDI-MS analysis surprisingly revealed a Pd-containing complex (Figure 4), with the mass corresponding to an intact palladated adduct (also carrying a single molecule of MALDI matrix, 3-hydroxypicolinic acid); any Pd-coordinating ligand was likely lost during the HPLC purification in triethylamine⋅acetate buffer. In TRIS buffer, this species was only observed during the cross-coupling with boronate ester **9**. Notably, treatment of starting ODN1 with [Pd]⋅L1 complex alone did not result in observation of the Pd-containing species. These observations suggest that the isolated Pd complex may reflect a catalytic intermediate likely formed after oxidative addition but prior to transmetalation. This would indicate that transmetalation is a rate-limiting step for these substrates (see the Supporting Information). To our knowledge, this is the first time such an apparent carbo-palladated DNA adduct has been isolated.

**Figure 4:**
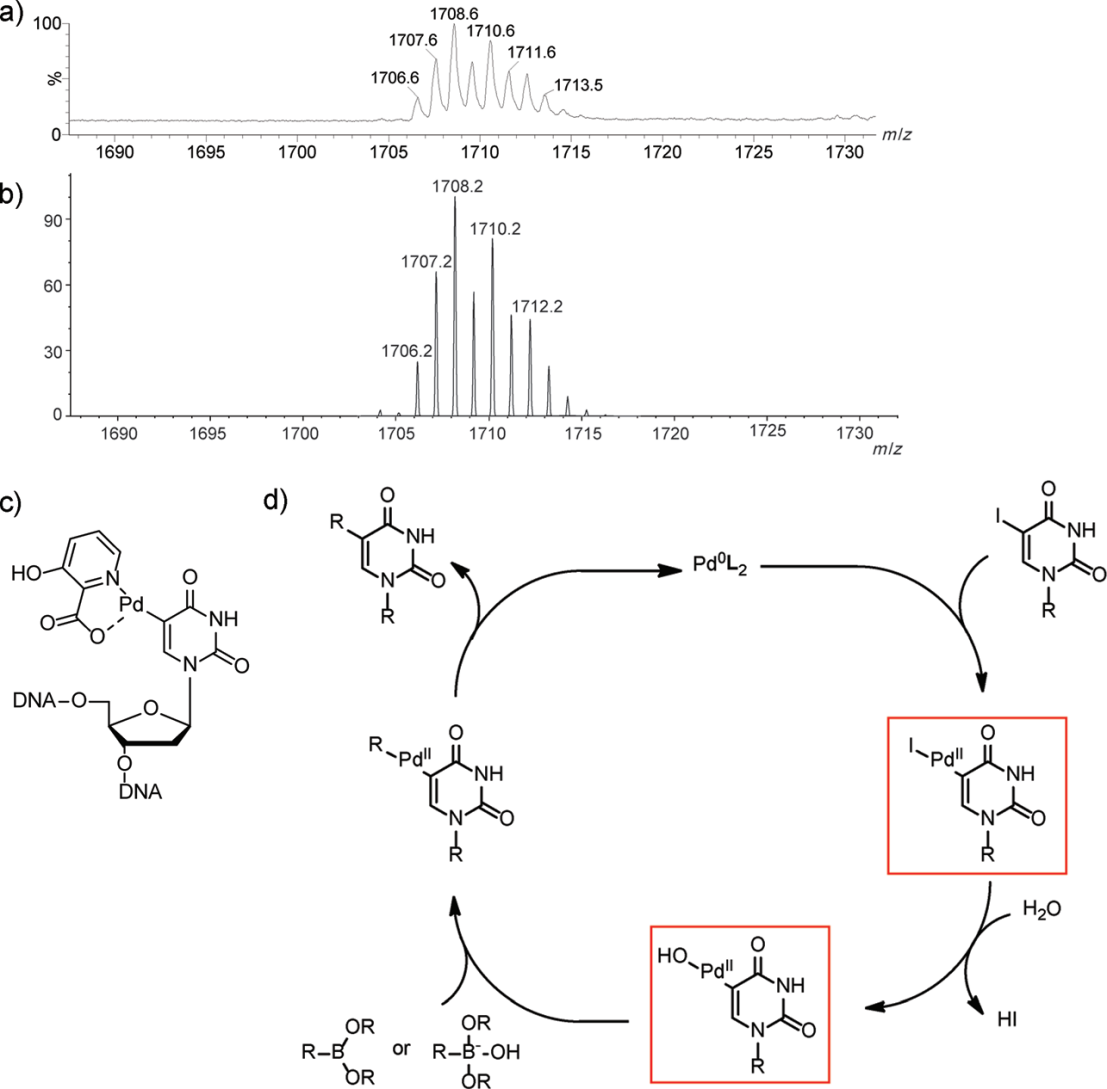
a) MALDI-TOF-MS analysis of the adduct corresponding to the second peak in the HPLC chromatogram of the reaction of **9** with ODN1. b) Calculated isotope distribution. c) Proposed palladated adduct (contains one molecule of MALDI matrix, 3-hydroxypicolinic acid). d) Suggested reaction mechanism for ODN Suzuki–Miyaura cross-coupling (possible precursors to isolated intermediate boxed).

To investigate how longer strands of DNA would behave under the reaction conditions, we performed the cross-coupling on a 13-mer (ODN3, Figure 1 b) and a biotin-containing ssDNA 21-mer (ODN4, Figure 1 b). The reactions gave good yields of 59–83 % even at these longer lengths (Table 2). While ODN3 has a central 5-IdU, the reactive base in ODN4 is positioned towards the 5′-end of the ODN. The differences in reactivity and yield between the two strands were small, thus suggesting that the position of the reactive base does not greatly influence the rate of reaction, highlighting the flexibility and generality of the methodology. The acceleration and increased yield upon the addition of TRIS were even more pronounced with these longer ODN (see the Supporting Information for comparison). Notably ODN4d,f are examples of ODNs containing dual nonnatural functional modifications (biotin+crosslinker).

**Table 2:** Conditions and yields for Suzuki–Miyaura cross-coupling on longer ODNs with different boronic esters

Product	Boronic ester	Pd (equiv), L	*t* [h]	Yield [%]
ODN3b	**7**	0.5, L2	6	59^[a]^
ODN3c	**8**	0.5, L2	16	83^[a]^
ODN3d	**4**	0.5, L2	6	73^[a]^
ODN3f	**5**	0.5, L2	6	71^[a]^
ODN4d	**4**	0.5, L1	6	67^[b]^
ODN4f	**5**	0.5, L1	6	78^[b]^
ODN5f	**5**	1, L1	16	56^[a]^
ODN6f	**5**	1, L2	8	69^[a]^

[a] Determined by HPLC (*λ*_max_ 260 nm) [b] Yield of isolated product.

We then tested whether multiple modifications can be introduced into an ODN containing two 5-IdU bases (ODN5). While requiring a longer reaction time for completion, the doubly modified product was isolated in acceptable yield. To further expand the substrate scope to other bases, we also examined a 5-IdC-containing ODN as substrate (ODN6) and obtained ODN6f in good yield. Finally, to test the possibility of direct modification of dsDNA we tested the reaction of **5** with dsODN3. Although unoptimized, initial results suggest successful reaction and the survival of even dsDNA under these conditions (see the Supporting Information).

To test whether a multiply modified retrievable, cross-linking probe could be introduced into modified DNA strands, we aimed to prepare a sequence also containing hmC bases. HmC has recently been identified as a common modification in CpG islands.^40^ This probe design (Figure 5) necessitated the generation of three simultaneous modifications: biotin (for affinity retrieval), diazirine (for crosslinking), and hydroxymethyl (the natural modification). Notably, the latter two modifications are unstable, necessitating mild conditions. While different chemical reactions for the introduction of these functional groups (and the others that we have explored) can be envisaged, many of these result in the retention of an undesired linker from the conjugation that could potentially interfere with protein binding.

**Figure 5:**
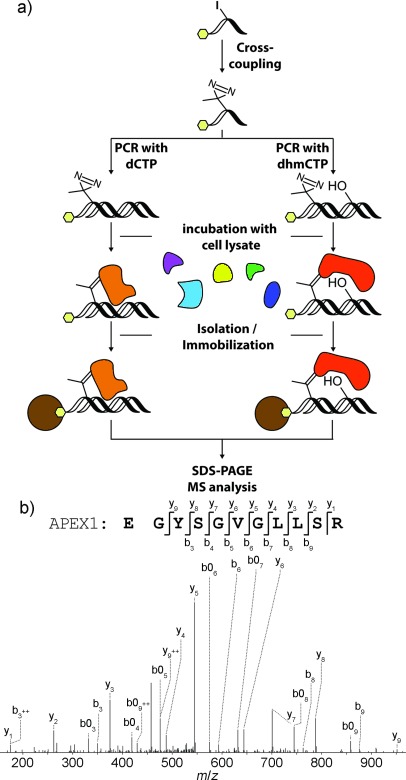
Creation of a triply modified probe system and its use in cross-linking to proteins from the nuclear extract of HeLa cells and the retrieval of cross-linked protein fragments. a) The probe system generated contains three modifications: biotin (nonnatural for affinity retrieval, yellow), diazirine (nonnatural for crosslinking), and hydroxymethylation (natural in hmC); these modifications allow affinity retrieval (using immobilized streptavidin beads, shown in brown) and identification of cross-linked products. b) MS/MS analysis of an identified peptide fragment, here from the DNA lyase APEX1 (IPI Nr IPI00215911; EGYSGVGLLSR), notably as an example of a DNA-binding protein.

We generated probes containing C (double modification) or hmC (triple modification) by PCR using ODN4d as a primer. To introduce hmC into DNA, dCTP was substituted with dhmCTP in the PCR reaction using the Vent *exo*-polymerase. No noticeable change in PCR efficiency was observed upon substitution. To confirm that the modification in ODN4d did not influence the PCR, we compared modified and unmodified ODN4 under standard PCR conditions (see the Supporting Information). The results were indistinguishable, thus suggesting that modification did not greatly influence polymerase efficiency.

To demonstrate the utility of the generated probes, we investigated their photo-crosslinking (Figure 5) to proteins from nuclear extracts (see the Supporting Information) and successfully generated cross-linked ODN-to-protein conjugates that could be retrieved using the biotin modification as an affinity tag. Retrieval occurred only after irradiation, consistent with the intended function of the cross-linking modification (diazirine). This was confirmed by MS analysis of the tryptic peptides from cross-linked proteins (see Table A/B in the Supporting Information for the list of associated proteins). We were also able to combine the probe’s use with the use of isotope-labeled amino acids in cell culture (SILAC)-based proteomics to quantitate the differential presence of proteins associated with hmC- versus C-containing DNA.^41^ In particular, we identified several DNA binding proteins such as p20-CGGBP, p52/p100, and LIG3, all of which are associated with binding directly to modified nucleotides or being involved in DNA repair mechanisms (see the Supporting Information for more detail). The preparation and use of such probes demonstrates that the mild, convergent Suzuki methodology described here can be successfully applied to the generation of even multiply modified DNA strands that contain useful and sensitive, natural and nonnatural moieties. Following this proof-of-principle, future work will investigate the application of these and related probes to elucidate differential protein binders of C and hmC in different biological contexts.

In conclusion, we have described an efficient method for the introduction of a variety of sensitive and useful functional groups by Suzuki–Miyaura cross-coupling to halogenated pyrimidine bases under mild/ambient conditions and temperature in aqueous TRIS-buffered solution. The method enables divergent synthesis of many different functionalized ODNs, without the need for expertise in DNA synthesis. In contrast to previous methodology, the direct conjugation to the nucleobase 5-position allows greater flexibility in the incorporated structure. The reported cross-coupling methodology is orthogonal to many reported conjugation methods and supplements these to enable the introduction of multiple different modifications.

## Experimental Section

Representative cross-coupling reaction: ODN (100 μm), Tris (50 mm, pH 8.5), [Pd(OAc)_2_L1_2_] (10–50 μm) and boronic ester (10 mm) were combined and shaken at 37 °C, the reaction centrifuged (2 min, 16k×g), and the supernatant analyzed by HPLC and MS.

Use of cross-linking DNA probe: Different hmC- and C-containing probes were incubated with HeLa cell nuclear extract and cross-linked (irradiation at 311 or 365 nm). ODN-to-protein conjugates were isolated using streptavidin and separated by SDS-PAGE. Proteins covalently bound to the probe ODNs were visualized by silver or Coomassie staining or Streptavidin-AP Western blot (see the Supporting Information). Cross-linking (clear, observed bands) occurred only after irradiation. Bound proteins were identified by LC–MS/MS in combination with SILAC-based quantitation^41^ (see the Supporting Information for the method and a full list of proteins identified).

## Supplementary material

As a service to our authors and readers, this journal provides supporting information supplied by the authors. Such materials are peer reviewed and may be re-organized for online delivery, but are not copy-edited or typeset. Technical support issues arising from supporting information (other than missing files) should be addressed to the authors.

## References

[1a] Seeman N (2007). Mol. Biotechnol.

[1b] Tørring T, Voigt NV, Nangreave J, Yan H, Gothelf KV (2011). Chem. Soc. Rev.

[1c] McLaughlin CK, Hamblin GD, Sleiman HF (2011). Chem. Soc. Rev.

[2a] Yin P, Yan H, Daniell XG, Turberfield AJ, Reif JH (2004). Angew. Chem.

[b01] (2004). Angew. Chem. Int. Ed.

[2b] He Y, Liu DR (2010). Nat. Nanotechnol.

[3a] Silverman SK (2010). Angew. Chem.

[b02] (2010). Angew. Chem. Int. Ed.

[3b] Breaker RR, Joyce GF (1994). Chem. Biol.

[3c] Silverman SK (2005). Nucleic Acids Res.

[3d] Silverman SK (2009). Acc. Chem. Res.

[4a] Genot AJ, Bath J, Turberfield AJ (2011). J. Am. Chem. Soc.

[4b] Qian L, Winfree E (2011). Science.

[5] Hocek M, Fojta M (2011). Chem. Soc. Rev.

[6] Østergaard ME, Hrdlicka PJ (2011). Chem. Soc. Rev.

[7a] Yang H, Metera KL, Sleiman HF (2010). Coord. Chem. Rev.

[7b] Clever GH, Kaul C, Carell T Angew. Chem.

[b03] (2007). Angew. Chem. Int. Ed.

[8] Endo M, Sugiyama H (2009). ChemBioChem.

[9] Beaucage SL, Iyer RP (1993). Tetrahedron.

[10] Krotz AH, Rentel C, Gorman D, Olsen P, Gaus HJ, McArdle JV, Scozzari AN (2004). Nucleosides Nucleotides Nucleic Acids.

[11] Malakhov AD, Malakhova EV, Kuznitsova SV, Grechishnikova IV, Prokhorenko IA, Skorobogatyi MV, Korshun VA, Berlin YA (2000). Russ. J. Bioorg. Chem.

[12] Jäger S, Rasched G, Kornreich-Leshem H, Engeser M, Thum O, Famulok M (2005). J. Am. Chem. Soc.

[13] Ménová P, Cahová H, Plucnara M, Havran L, Fojta M, Hocek M (2013). Chem. Commun.

[14a] Morales JC, Kool ET (1999). J. Am. Chem. Soc.

[14b] Spratt TE (2001). Biochemistry.

[15a] Vaught JD, Bock C, Carter J, Fitzwater T, Otis M, Schneider D, Rolando J, Waugh S, Wilcox SK, Eaton BE (2010). J. Am. Chem. Soc.

[15b] Sakthivel K, Barbas CF (1998). Angew. Chem.

[b04] (1998). Angew. Chem. Int. Ed.

[15c] Lee SE, Sidorov A, Gourlain T, Mignet N, Thorpe SJ, Brazier JA, Dickman MJ, Hornby DP, Grasby JA, Williams DM (2001). Nucleic Acids Res.

[16] Baccaro A, Steck AL, Marx A (2012). Angew. Chem.

[b05] (2012). Angew. Chem. Int. Ed.

[17] Seo YJ, Malyshev DA, Lavergne T, Ordoukhanian P, Romesberg FE (2011). J. Am. Chem. Soc.

[18a] Beyer C, Wagenknecht H-A (2010). Chem. Commun.

[18b] El-Sagheer AH, Brown T (2010). Chem. Soc. Rev.

[19] Gutsmiedl K, Wirges CT, Ehmke V, Carell T (2009). Org. Lett.

[20a] Borsenberger V, Howorka S (2009). Nucleic Acids Res.

[20b] Schoch J, Wiessler M, Jäschke A (2010). J. Am. Chem. Soc.

[21] Raindlová V, Pohl R, Šanda M, Hocek M (2010). Angew. Chem.

[b06] (2010). Angew. Chem. Int. Ed.

[22] Raindlová V, Pohl R, Hocek M (2012). Chem. Eur. J.

[23] Wang CCY, Seo TS, Li Z, Ruparel H, Ju J (2003). Bioconjugate Chem.

[24] Shigdel UK, Zhang J, He C (2008). Angew. Chem.

[b07] (2008). Angew. Chem. Int. Ed.

[25] Rohde H, Schmalisch J, Harpaz Z, Diezmann F, Seitz O (2011). ChemBioChem.

[26] Eberhard H, Diezmann F, Seitz O (2011). Angew. Chem.

[b08] (2011). Angew. Chem. Int. Ed.

[27] Schlegel MK, Hütter J, Eriksson M, Lepenies B, Seeberger PH (2011). ChemBioChem.

[28a] Omumi A, Beach DG, Baker M, Gabryelski W, Manderville RA (2010). J. Am. Chem. Soc.

[28b] Cahová H, Jäschke A (2013). Angew. Chem.

[b09] (2013). Angew. Chem. Int. Ed.

[29] Chalker JM, Wood CSC, Davis BG (2009). J. Am. Chem. Soc.

[30a] Winnacker M, Breeger S, Strasser R, Carell T (2009). ChemBioChem.

[30b] Winnacker M, Welzmiller V, Strasser R, Carell T (2010). ChemBioChem.

[31] Zhang M, Lin S, Song X, Liu J, Fu Y, Ge X, Fu X, Chang Z, Chen PR (2011). Nat. Chem. Biol.

[32] Spicer CD, Davis BG (2011). Chem. Commun.

[33] Amatore C, Jutand A, Le Duc G (2011). Chem. Eur. J.

[34] Carrow BP, Hartwig JF (2011). J. Am. Chem. Soc.

[35] Beharry AA, Woolley GA (2011). Chem. Soc. Rev.

[36] Scheibe C, Bujotzek A, Dernedde J, Weber M, Seitz O (2011). Chem. Sci.

[37] Yan H, Tram K (2007). Glycoconjugate J.

[38] Srivatsan SG, Tor Y (2007). J. Am. Chem. Soc.

[39a] Greco NJ, Tor Y (2005). J. Am. Chem. Soc.

[39b] Greco NJ, Sinkeldam RW, Tor Y (2009). Org. Lett.

[40a] Kriaucionis S, Heintz N (2009). Science.

[40b] Branco MR, Ficz G, Reik W (2012). Nat. Rev. Genet.

[41] Mann M (2006). Nat. Rev. Mol. Cell Biol.

