# Supplementary material for: DNA Modification under Mild Conditions by Suzuki–Miyaura Cross-Coupling for the Generation of Functional Probes
Source: Angew Chem Int Ed Engl. 2013 Aug 13;52(40):10553–8. doi: 10.1002/anie.201304038 (PMC3823066; doi:10.1002/anie.201304038)
Supplement: Supplementary file 1 [file anie0052-10553-SD1.pdf]

Angewandte Chemie
Eine Zeitschrift der Gesellschaft Deutscher Chemiker

Supporting Information

© Wiley-VCH 2013

69451 Weinheim, Germany

DNA Modification under Mild Conditions by Suzuki–Miyaura Cross-Coupling for the Generation of Functional Probes**

*Lukas Lercher, Joanna F. McGouran, Benedikt M. Kessler, Christopher J. Schofield, and Benjamin G. Davis**

anie_201304038_sm_miscellaneous_information.pdf

Contents

General considerations 3
Boronic esters used in this study 4
Synthesis of the short boronic ester amines for coupling 4
Synthesis of the long boronic ester amines for coupling 6
Amide couplings 8
Small molecule cross-couplings 11
Catalyst preparation 12
ODN crosscoupling 13
- General reaction conditions 13
- ODN1a 13
- ODN1b 14
- ODN1c 14
- ODN1d 15
- ODN1e 15
- ODN1e reaction with 3-furane boronic acid 16
- ODN1f 16
- ODN1g 17
- ODN1 Paladium adduct observed in PO4 buffer 17
- ODN1 reaction intermediates by ESI-MS 18
- ODN2 (no IdU control) 18
- ODN3b 19
- ODN3c 19
- ODN3d 20
- ODN3f 20
- ODN4 control reactions 21
- ODN4 – Phosphate and Tris buffer comparison 22
- ODN4d 23
- ODN4f 23
- ODN5f 24
- ODN6f 24
- dsDNA crosscoupling 25

PCR probe generation 27

Photocrosslinking 27
Streptavidin blot 28
Supplementary Results 28
Supplementary Table 2 31

General considerations

Proton nuclear magnetic resonance (^1H NMR) spectra were recorded on a Bruker DPX200 (200 MHz), a Bruker AV400 (400 MHz), or a Bruker AVII500 (500 MHz) spectrometer, as indicated. Carbon nuclear magnetic resonance (^{13}C NMR) spectra were recorded on a Bruker AV400 (100 MHz) or on a Bruker AVII500 (125 MHz) spectrometer, as indicated. NMR Spectra were assigned using COSY, HMQC, and DEPT 135. All chemical shifts are quoted on the δ scale in ppm using residual solvent as the internal standard (^1H NMR: $CDCl_3$ = 7.26, CD_3OD = 3.31; DMSO-d6 = 2.50; CD_3CN = 1.94 and ^{13}C NMR: $CDCl_3$ = 77.0; CD_3OD = 49.0; DMSO-d6 = 39.5; CD_3CN = 1.94). Coupling constants (*J*) are reported to the nearest 0.5 Hz with the following splitting abbreviations: s = singlet, d = doublet, t = triplet, q = quartet.

Infrared (IR) spectra were recorded on a Bruker Tensor 27 Fourier Transform spectrophotometer neat for both oils and solids. Absorption maxima (υ_{max}) are reported in wavenumbers (cm^{-1}). Low resolution mass spectra (LRMS) were recorded on a Waters Micromass LCT Premier TOF spectrometer Waters using electrospray ionization (ESI) and high resolution mass spectra (HRMS) were recorded on a Bruker MicroTOF ESI mass spectrometer. Nominal and exact m/z values are reported in Daltons.

Thin layer chromatography (TLC) was carried out using Merck aluminium backed sheets coated with 60F254 silica gel. Visualization of the silica plates was achieved using a UV lamp (λ_{max} = 254 nm), and/or ammonium molybdate (5% in 2 M H_2SO_4), and/or potassium permanganate (5% $KMnO_4$ in 1 M NaOH with 5% potassium carbonate).

Column chromatography was performed on a Biotage SP4 flash purification system using the indicated cartridges and solvent systems.

Anhydrous solvents were obtained from Fluka or Acros with the exception of CH_2Cl_2 and THF, which were dried in an alumina column under nitrogen. All other solvents were used as supplied (Analytical or HPLC grade), without prior purification. Deionized water was used for chemical reactions. Reagents were purchased from Sigma-Aldrich and used as supplied, unless otherwise indicated. All reactions using anhydrous conditions were performed using flame-dried apparatus under an atmosphere of argon or nitrogen.

Boronic ester **6** was prepared by Justin M. Chalker, as described previously[1].

DNA analysis was performed on an Agilent 1260 Infinity system using an Agilent Eclipse Plus C18 column (4.6 x 100 mm, 3.5 µm). 0.1M triethylamine acetate (TEAA) in water (solvent A) and 0.1M TEAA in 80:20 acetonitrile:water (solvent B) were used as mobile phase. The mixtures were separated by a linear gradient as stated in the example. After this the column was washed and equilibrated (5

min – 40->100% B; 10 min – 100% B; 15 min – 100->0% B; 20 min 0% B). The run was monitored by UV absorption at 260nm. Collected fractions were freeze-dried and analyzed by MALDI-TOF using 3-hydroxypicolinic acid (hpa) as matrix or by ESI. The hpa stock was prepared as a saturated solution in water:acetonitrile 1:1 and mixed with the sample 1:1 on the MALDI target. MALDI analysis was performed on a Waters Micromass MALDI micro MX using the manufacturer's recommended settings. For ESI analysis, samples were dissolved in water and injected in a flow of 1:1 Water:Methanol with 0.1mM NH_4OAc. The analysis was performed on a Micromass LCT (ESI-TOF-MS). The data was processed using MassLynx.

Boronic esters used in this study

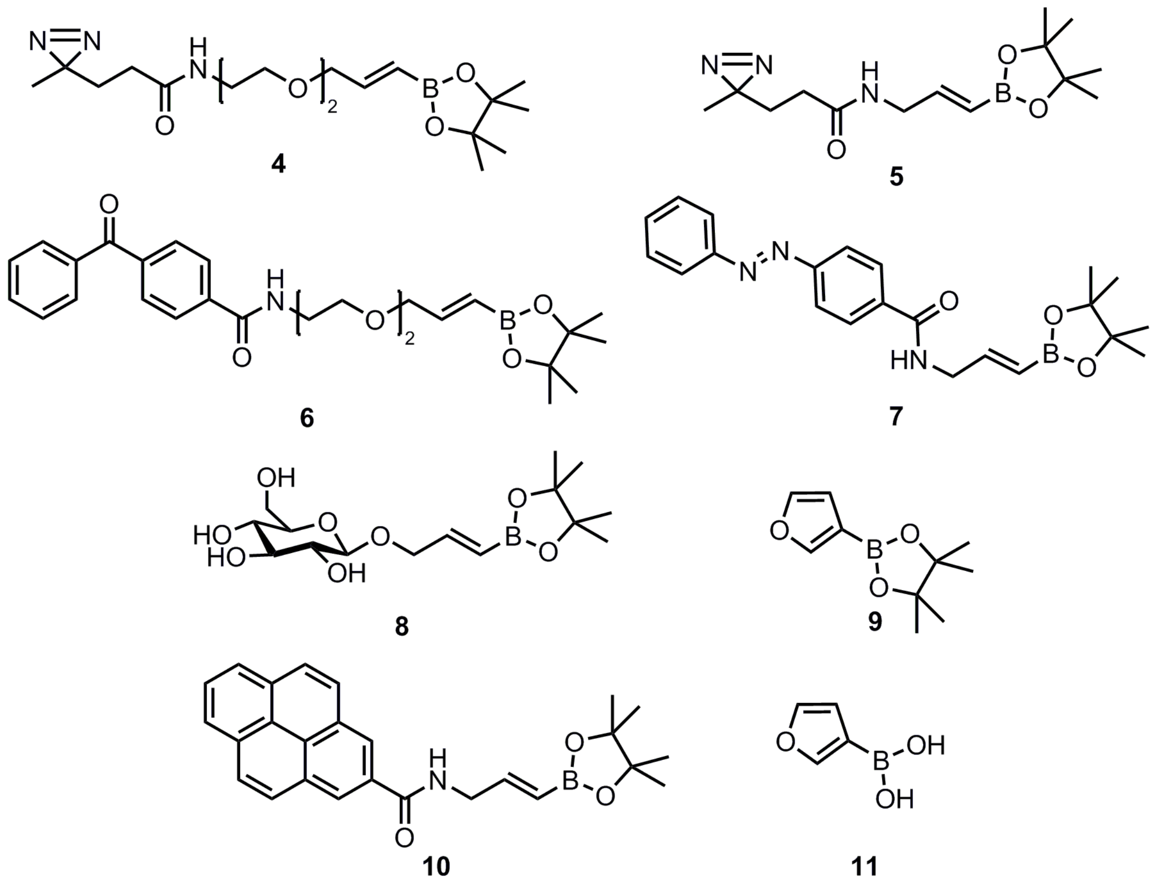

Supplemental Figure S1 Boronic acid pinacol esters used in this study.

For the synthesis of the different boronic esters, firstly two amine-containing boronic acid pinacol esters with different length linkers (see SI, with zero (**S1**) or two (**S2**) PEG units), starting from propargylamine or 2-(2-aminoethoxy)ethanol, were synthesized. The resulting amines were coupled to different carboxylic acids (mediated by carbodiimide), yielding the desired boronate esters.

Synthesis of the short boronic ester amines for coupling

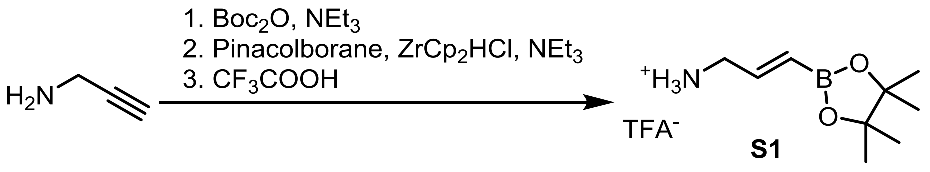

tert-Butyl prop-2-yn-1-ylcarbamate[2]

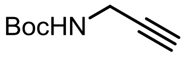

To a solution of di-*tert*-butyl dicarbonate (4.8 g, 22 mmol, 1.1 eq) in CH_2Cl_2 (20 mL) at 0°C was added dropwise propargylamine (1.3 mL, 1.1 g, 20 mmol, 1 eq) and subsequently triethylamine (5.6 mL, 4.05 g, 40 mmol, 2 eq). The resulting solution was warmed to rt and allowed to stir for 2 h. Then the reaction was quenched with sat. NH_4Cl (10 mL) and diluted with EtOAc (150 mL). The organic phase was washed with sat. NH_4Cl, sat. $NaHCO_3$ and brine; dried ($MgSO_4$), filtered and solvents were removed *in vacuo* yielding the title compound (2.68 g, 17 mmol, 85%) as a clear oil.

^{1}H NMR (400 MHz, $CDCl_3$) d ppm 1.46 (s, 9 H, $(C\underline{H}_3)_3C$) 2.22 (t, J=2.5 Hz, 1 H, $CC\underline{H}$) 3.92 (m, 2 H, $C\underline{H}_2$) 4.72 (br. s., 1 H, $N\underline{H}$); ^{13}C NMR (101 MHz, $CDCl_3$) d ppm 28.3 ($(\underline{C}H_3)_3C$) 30.4 ($\underline{C}H_2$) 71.2 ($C\underline{C}H$) 79.8 ($(CH_3)_3\underline{C}$) 80.0 (C) 155.3 (OCONH); LRMS m/z (ESI+): 178.09 $[M+Na]^+$; IR (neat): 3301, 2979, 2933, 1692, 1510, 1455 cm^{-1}.

(E)-_tert_-Butyl 3-(4,4,5,5-tetramethyl-1,3,2-dioxaborolan-2-yl)allylcarbamate[3]

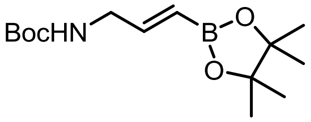

To neat *tert*-butyl prop-2-ynylcarbamate was added Pinacolborane (1.4 mL, 1.23 g, 9.6 mmol, 1.5 eq), NEt_3 (100 uL, 0.64 mmol, 0.1 eq) and $ZrCp_2HCl$ (165 mg, 0.64 mmol, 0.1 eq). The solution was stirred at 65°C for 16 h. After completion the reaction was allowed to cool to rt and was diluted with EtOAc (25 mL) and quenched with sat. NH_4Cl. The mixture was further diluted with EtOAc (50 mL) and the organic layer was washed with sat. NH_4Cl, sat. $NaHCO_3$ and brine, dried ($MgSO_4$), filtered and solvents were removed *in vacuo*. The product was purified by automated column chromatography (Biotag SNAP 50g cartridge 0-40% over 10 CV EtOAc in petrol) yielding the title compound as a yellow oil (1.27g, 4.5 mmol, 70%).

^{1}H NMR (400 MHz, $CDCl_3$) d ppm 1.26 (s, 12 H, $(C\underline{H}_3)_2COB$) 1.44 (s, 9 H, $(C\underline{H}_3)_3C$) 3.84 (s, 2 H, $C\underline{H}_2$) 4.67 (s, 1 H, $N\underline{H}$) 5.57 (dd, J=18, 2 Hz, 1 H, $C\underline{H}B$) 6.58 (d, J=18 Hz, 1 H, $C\underline{H}CH_2$); ^{13}C NMR (101 MHz, $CDCl_3$) d ppm 24.7 ($\underline{C}H_2C\underline{C}H_2$) 28.4 ($(\underline{C}H_3)_3C$) 44.0 ($CNH\underline{C}H_2$) 79.4 ($\underline{C}OB$) 83.3 ($(CH_3)_3\underline{C}$) 149.4 (CHB) 155.7 (OCONH); LRMS m/z (ESI+): 306.20 $[M+Na]^+$; IR (neat): 3356, 2978, 2932, 2361, 2341, 1697, 1643, 1519 cm^{-1}.

(E)-3-(4,4,5,5-_tetra_-Methyl-1,3,2-dioxaborolan-2-yl)prop-2-en-1-amine TFA salt (S1)

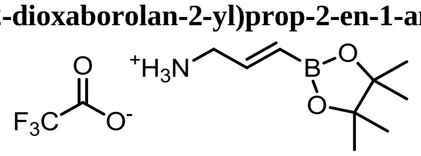

(E)-*tert*-Butyl 3-(4,4,5,5-tetramethyl-1,3,2-dioxaborolan-2-yl)allylcarbamate (250 mg, 0.88 mmol, 1 eq) was dissolved in 10 mL 9:1 CH_2Cl_2:CF_3COOH and stirred at rt for 1h. Then solvents were removed *in vacuo* and remaining CF_3COOH was removed by coevaporation (3x Toluene, 3x CH_2Cl_2) yielding **S1** (240 mg, 0.81 mmol, 92%) as a brown oil.

^{1}H NMR (400 MHz, $CDCl_3$) d ppm 1.23 (s, 12 H, $(C\underline{H}_3)_2COB$) 3.64 (br. s., 2 H, $C\underline{H}_2$) 5.73 (d, J=18 Hz, 1 H, $C\underline{H}B$) 6.46 - 6.60 (m, 1 H, $C\underline{H}CH_2$) 8.21 (br. s., 3 H, $N\underline{H}$); ^{13}C NMR (101 MHz, CHLOROFORM-*d*) d ppm 24.6 ($(\underline{C}H_3)_2COB$) 42.8 ($\underline{C}H_2$) 83.8 ($\underline{C}OB$) 116.1 (J=293 Hz, $\underline{C}F_3$) 124.1 ($\underline{C}HCHB$) 142.1 ($\underline{C}HB$) 162.1 (J=36 Hz, $CF_3\underline{C}OOH$; LRMS m/z (ESI$^+$): 184.16 $[M+H]^+$ IR (neat): 2982, 1675, 1651, 1533 cm^{-1} HRMS *m/z* (ESI+): Found 184.1503 $(M+H)^+$; $C9H_{19}BNO_2$ requires 184.1503

Synthesis of the long boronic ester amines for coupling

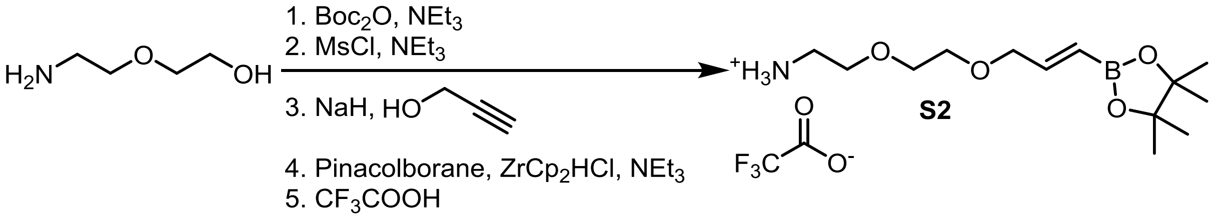

tert-Butyl 2-(2-hydroxyethoxy)ethylcarbamate

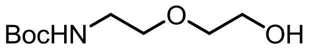

To a solution of di-*tert*-butyl dicarbonate (21.83 g, 100 mmol, 1 eq) in CH_2Cl_2 (70 mL) on ice was added 2-(2-aminoethoxy)ethanol (12.0 mL, 12.6 g, 120 mmol, 1.2 eq) dropwise, subsequently NEt_3 (27.8 mL, 20.24 g, 200 mM, 2 eq). The resulting mixture was stirred at rt for 3 h. The reaction was quenched with sat. NH_4Cl, diluted with EtOAc (400 mL), washed with sat. NH_4Cl (2x200 mL) and brine (200 mL), dried ($MgSO_4$), filtered and solvents were removed *in vacuo*. The title compound was obtained as a clear oil (18.2 g, 88.7 mmol, 89%) and was used in the next step without further purification.

^{1}H NMR (400 MHz, $CDCl_3$) d ppm 1.42 (s, 9 H, Boc-H) 3.01 (s, 1 H, OH) 3.28 (d, *J*=5 Hz, 2 H, CH_2) 3.52 (dt, *J*=9.5, 5 Hz, 4 H, CH_2) 3.65 - 3.73 (m, 2 H, CH_2) 5.24 (s, 1 H, NH); ^{13}C NMR (101 MHz, $CDCl_3$) d ppm 28.4 (($(CH_3)_3$)) 40.3 (CH_2) 61.5 (CH_2) 70.3 (CH_2) 72.2 (CH_2) 79.3 ($C(CH_3)_3$) 156.2 (CONH); LRMS m/z (ESI$^+$): 228.13 $[M+Na]^+$; IR (neat): 3343, 2977, 2361, 1691, 1525 cm^{-1}.

2-(2-(_tert_-Butoxycarbonylamino)ethoxy)ethyl methanesulfonate

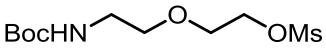

To a solution of *tert*-butyl 2-(2-hydroxyethoxy)ethylcarbamate (17.8 g, 87 mmol, 1 eq) in 100 mL CH_2Cl_2 at 0°C was added NEt_3 (26.7 mL, 19.4 g, 191 mmol, 2.2 eq) and then dropwise CH_3SO_2Cl (7.4 mL, 11 g, 96 mmol, 1.1 eq). The cloudy yellow solution was stirred on ice for 5 h and then quenched with sat. NH_4Cl. The reaction was then diluted with EtOAc (400 mL), washed with sat. NH_4Cl (200 mL), $NaHCO_3$ (200 mL) and brine (200 mL), dried ($MgSO_4$), filtered and solvents were removed *in vacuo*. The product was purified by column chromatography (Biotage KP-SIL SNAP 100 g cartridge 0-50% over 10CV EtOAc in petrol) yielding the title compound (18.35 g, 65 mmol, 75%) as an orange oil.

^{1}H NMR (400 MHz, $CDCl_3$) d ppm 1.42 (s, 9 H, Boc-H) 3.04 (s, 3 H, Ms-H) 3.30 (q, J=5 Hz, 2 H, CH_2) 3.54 (t, J=5 Hz, 2 H, CH_2) 3.68 - 3.75 (m, 2 H, CH_2) 4.30 - 4.38 (m, 2 H, CH_2) 4.92 (s, 1 H, NH); ^{13}C NMR (101 MHz, $CDCl_3$) d ppm 28.4 (($CH_3)_3$C) 37.7 (CH_3S) 40.2, 68.6, 68.8, 70.3 (CH_2OS) 79.4 ($C(CH_3)_3$) 155.9 (OCCNH); LRMS m/z (ESI$^+$): 306.10 $[M+Na]^+$; IR (neat): 3402, 2978, 2936, 2874, 2361, 2342, 1701, 1512 cm^{-1}.

tert-Butyl 2-(2-(prop-2-ynyloxy)ethoxy)ethylcarbamate

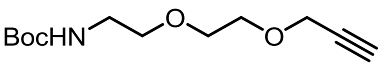

To a solution of propargylic alcohol (5.24 mL, 5.05 g, 90 mmol, 3 eq) in 100 mL THF on ice was added NaH (2.16 g, 90 mmol, 3 eq) portion wise. The resulting suspension was stirred on ice for 30 min then 2-(2-(*tert*-butoxycarbonylamino)ethoxy)ethyl methanesulfonate (8.5 g, 30 mmol, 1 eq) was added dropwise. The resulting suspension was stirred at rt for 19 h. The reaction was then quenched with sat. NH_4Cl (10 mL) and solvents were removed *in vacuo*. The resultant red liquid was partitioned between H_2O (50 mL) and EtOAc (350 mL). The organic phase was washed with sat. NH_4Cl (100

mL), sat. $NaHCO_3$ (100 mL) and brine (100 mL), dried ($MgSO_4$), filtered and solvents were removed *in vacuo*. The crude oil was purified by column chromatography (Biotage KP-SIL SNAP 100 g cartridge 0-50% over 10CV EtOAc in petrol) yielding the title compound (5.81 g, 24 mmol, 80%) as a clear oil.

^{1}H NMR (400 MHz, $CDCl_3$) d ppm 1.42 (s, 9 H, Boc-H) 2.44 (t, J=2.5 Hz, 1 H, CCH) 3.30 (q, J=5 Hz, 2 H, CH_2) 3.53 (t, J=5 Hz, 2 H, CH_2) 3.59 - 3.65 (m, 2 H, CH_2) 3.65 - 3.71 (m, 2 H, CH_2) 4.19 (d, J=2.5 Hz, 2 H, CH_2CCH) 4.99 (s, 1 H, NH); ^{13}C NMR (101 MHz, $CDCl_3$) d ppm 28.4 ($(CH_3)_3$C) 40.3 (CH_2), 58.4 (CH_2CC) 69.0, 70.1, 70.3 (CH_2) 74.7 (CCH) 79.2 (CCH) 79.5 (C$(CH_3)_3$) 156 (OCCNH); LRMS m/z (ESI$^+$): 266.14 $[M+Na]^+$; IR (neat): 3296, 2870, 1698, 1512 cm^{-1}.

(E)-*tert*-Butyl 2-(2-(3-(4,4,5,5-tetramethyl-1,3,2-dioxaborolan-2-yl)allyloxy)ethoxy) ethylcarbamate[3b]

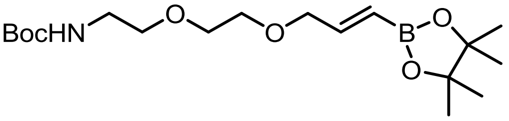

To neat *tert*-butyl 2-(2-(prop-2-ynyloxy)ethoxy)ethylcarbamate (4.86 g, 20 mmol, 1 eq) was added pinacolborane (3.5 mL, 3.07 g, 22 mmol, 1.1 eq), NEt_3 (270 uL, 202 mg, 2 mmol, 0.1 eq) and $ZrCp_2HCl$ (516 mg, 2 mmol, 0.1 eq). The solution was stirred at 65°C for 17 h. After completion the reaction was allowed to cool to rt and was diluted with EtOAc (25 mL) and quenched with sat. NH_4Cl. The mixture was further diluted with EtOAc (150 mL) and the organic layer was washed with sat. NH_4Cl (50 mL), sat. $NaHCO_3$ (50 mL) and brine (50 mL), dried ($MgSO_4$), filtered and solvents were removed *in vacuo*. The product was purified by automated column chromatography (Biotag SNAP 100g cartridge 5-50% over 10CV EtOAc in petrol) yielding the title compound as a yellow oil (6.5 g, 17.5 mmol, 88%).

^{1}H NMR (400 MHz, $CDCl_3$) d ppm 1.24 (s, 12 H, pinacol-H) 1.42 (s, 9 H, Boc-H) 3.29 (s, 2 H, CH_2) 3.52 (s, 2 H, CH_2) 3.55 - 3.62 (m, 4 H, CH_2) 4.04 - 4.12 (m, 2 H, CH_2) 5.05 (s, 1 H, NH) 5.67 (d, *J*=18 Hz, 1 H, CHB) 6.60 (dd, *J*=18, 2.5 Hz, 1 H, CHCH$_2$); ^{13}C NMR (101 MHz, $CDCl_3$) d ppm 24.7 ($(CH_3)_3$C) 28.4 ($(CH_2)_2$C) 40.4, 69.7, 70.3, 72.8 (CH_2) 79.1 ($(CH_3)_3$C) 83.2 ($(CH_2)_2$C) 149.0 (CHB) 156.0 (OCONH); LRMS m/z (ESI$^+$): 394.25 $[M+Na]^+$ IR (neat): 3360, 2978, 2931, 2869, 1713, 1645, 1512 cm^{-1}; HRMS *m/z* (ESI+): Found 394.2367 $(M+Na)^+$; $C_{18}H_{34}BNO_3Na$ requires 394.2375.

(E)-2-(2-(3-(4,4,5,5-tetramethyl-1,3,2-dioxaborolan-2-yl)allyloxy)ethoxy)ethanamine (S2)

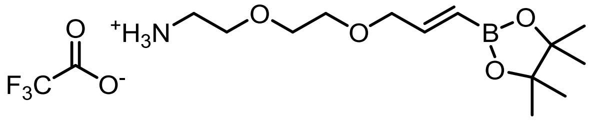

(E)-*tert*-Butyl-2-(2-(3-(4,4,5,5-tetramethyl-1,3,2-dioxaborolan-2-yl)allyloxy)ethoxy)ethylcarbamate (3.71 g, 10 mmol) was dissolved in 10% CF_3COOH in CH_2Cl_2 (50 mL total). The solution was then stirred for 3 h. The solvents were removed *in vacuo* and CF_3COOH was subsequently co-evaporated with toluene (2x30 mL) and CH_2Cl_2 (3x30 mL) yielding **S2** (3.41 g, 8.9 mmol, 89%) as a brown oil.

^{1}H NMR (400 MHz, $CDCl_3$) d ppm 1.26 (s, 12 H, pinacol-H) 3.22 (s, 2 H, CH_2) 3.61 (dd, *J*=6, 2.5 Hz, 2 H, CH_2) 3.67 (dd, *J*=6, 2.5 Hz, 2 H, CH_2) 3.70 - 3.79 (m, 2 H, CH_2) 4.10 (dd, *J*=5, 1.5 Hz, 2 H, CH_2) 5.61 - 5.73 (m, 1 H, CHB) 6.58 (dt, *J*=18, 5 Hz, 1 H, CHCH$_2$) 7.73 (s, 3 H, NH); ^{13}C NMR (101 MHz, CDCl3) d ppm 24.6 ($(CH_3)_3$C) 39.9, 66.4, 69.4, 70.1, 72.6 (CH_2) 83.6 ($(CH_2)_2$C) 118.4 (CHCHB) 148.1 (CHB); LRMS m/z (ESI$^+$): 272.22 $[M+H]^+$; IR (neat): 2984, 1783, 1673, 1646, 1519 cm^{-1}; HRMS *m/z* (ESI+): Found 272.2031 $(M+H)^+$; $C_{13}H_{27}BNO_4$ requires 272.2030.

Amide couplings

3-(3-Methyl-3H-diazirin-3-yl)propanoic acid (S3)[4]

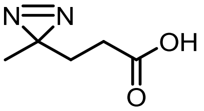

To NH_3 (150 mL) was added leuvenic acid (17.4 g, 150 mmol, 1 eq) at -78°C. The resulting clear solution was stirred at -50 to -40°C for 3.45 h. During this time a white precipitate was formed. The reaction mixture was then cooled to -78°C again and hydroxylamine-O-sulfonicacid (20.36 g, 180 mmol, 1.2 eq) in MeOH (150 mL) was added dropwise over 20 min. The reaction mixture was stirred at -78°C for 1.10 h and then let come to rt overnight. Most MeOH was removed *in vacuo* and the residue was taken up in MeOH (50 mL). NEt_3 was added and the resulting mixture was stirred at 0°C for 15 min. Solvents were removed *in vacuo* and the residue was taken up in MeOH (150 mL) and cooled to 0°C. NEt_3 (60 mL) was added and then I_2 was added in portions. The addition was continued until the colour of iodine persisted (total 33.2 g, 131 mmol). The reaction mixture was stirred at rt for 20 min. Subsequently, the solvents were removed and the residue was partitioned between Et_2O and water. The aqueous layer was acidified with 5M HCl and extracted with Et_2O (2x150 mL). The combined organic layers were washed with 20% $NaHSO_3$ and brine, dried ($MgSO_4$) filtered and solvents were removed *in vacuo* yielding sufficiently pure **S3** as a yellow oil (solidifies at -20°C; 7.82 g, 61 mmol, 40% yield).

^{1}H NMR (400 MHz, $CDCl_3$) d ppm 1.02 (s, 3 H, $C\underline{H}_3$) 1.69 (t, *J*=7.5 Hz, 2 H, $C\underline{H}_2$) 2.21 (t, *J*=7.5 Hz, 2 H, $C\underline{H}_2$) 11.11 (s, 1 H, $CO_2\underline{H}$); ^{13}C NMR (101 MHz, CDCl3) d ppm 19.6 ($C\underline{C}H_3$), 25.0 ($CH_3\underline{C}N_2CH_2$), 28.5, 29.3 ($\underline{C}H_2\underline{C}H_2$), 178.7 ($\underline{C}$OOH); LRMS m/z ($ESI^-$): 125.06 $[M\text{-}H]^-$; IR (neat): 2960, 1709, 1586 cm^{-1}.

(E)-3-(3-Methyl-3H-diazirin-3-yl)-*N*-(2-(2-(3-(4,4,5,5-tetramethyl-1,3,2-dioxaborolan-2-yl)allyloxy)ethoxy)ethyl)propanamide (4)

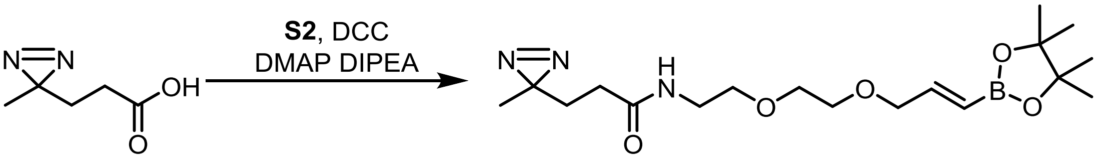

To a solution of **S3** (769 mg, 6 mmol, 1.5 eq) in CH_2Cl_2 (20 mL) on ice was added *N,N'*-dicyclohexylcarbodiimide (DCC; 1.32 g, 6.4 mmol, 1.6 eq). The resulting solution was stirred on ice for 30 min and then 4-dimethylaminopyridine (DMAP; 244 mg, 2 mmol, 0.5 eq) and *N,N*-diisopropylethylamine (DIPEA; 1.4 ml; 1.03 g, 8 mmol, 2 eq) and **S2** (1.54 g, 4 mmol, 1 eq) were added. The reaction mixture was stirred at rt overnight and then filtered (wash with EtOAc or Et_2O) and diluted with EtOAc (100 mL). The organic phase was washed with sat. NH_4Cl (50 mL), sat. $NaHCO_3$ (50 mL) and brine (50 mL), dried ($MgSO_4$), filtered and solvents were removed *in vacuo*. The residue was purified by automated column chromatography (Biotag SNAP 25 g cartridge 0-10% over 10 CV MeOH in CH_2Cl_2) yielding **4** (738 mg, 2.1 mmol, 35%) as a brown oil.

^{1}H NMR (400 MHz, $CDCl_3$) d ppm 1.02 (s, 3 H, $C\underline{H}_3CN_2$) 1.26 (s, 12 H, Pinacol-$C\underline{H}_3$) 1.73 (dd, *J*=9, 7 Hz, 2 H, $CN_2C\underline{H}_2C\underline{H}_2$) 2.01 (dd, *J*=9, 7 Hz, 2 H, $CN_2C\underline{H}_2C\underline{H}_2$) 3.44 (q, *J*=5 Hz, 2 H, $C\underline{H}_2$) 3.54 (s, 2 H, $C\underline{H}_2$) 3.58 - 3.62 (m, 4 H, $C\underline{H}_2$) 4.10 (dd, *J*=5, 2 Hz, 2 H, CH_2) 5.71 (dt, *J*=18, 2 Hz, 1 H, $C\underline{H}B$) 6.20 (br. s., 1 H, $N\underline{H}$) 6.62 (dt, *J*=18, 5 Hz, 1 H, $C\underline{H}CHB$); ^{13}C NMR (101 MHz, $CDCl_3$) d ppm 19.8 ($\underline{C}H_3$) 24.7 ($(\underline{C}H_3)_2C$) 25.4 ($CH_3\underline{C}NN$) 30.1 ($\underline{C}H_2$) 30.6 ($\underline{C}H_2$) 39.3, 69.7, 69.8, 70.3 ($\underline{C}H_2$) 72.7 ($O\underline{C}H_2CH$), 83.3 ($(CH_3)_2\underline{C}$) 148.8 ($\underline{C}HB$) 171.4 ($CH_2\underline{C}ONH$); LRMS *m/z* (ESI^+): 404.25 $[M+Na]^+$; IR (neat): 3308, 2978, 2928, 2869, 1726, 1646, 1551 cm^{-1}; HRMS *m/z* (ESI+): Found 404.2318 $(M+Na)^+$; $C_{18}H_{32}BN_3O_5Na$ requires 404.2330.

(E)-3-(3-Methyl-3H-diazirin-3-yl)-*N*-(3-(4,4,5,5-tetramethyl-1,3,2-dioxaborolan-2-yl)allyl)propanamide (5)

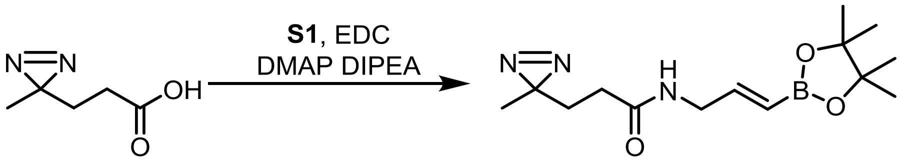

To a solution of *N*-(3-dimethylaminopropyl)-*N*′-ethylcarbodiimide hydrochloride (EDC.HCl; 422 mg, 2.2 mmol, 2.2 eq) in CH_2Cl_2 (15 mL) on ice was added **S3** (256 mg, 2 mmol, 2 eq). The resulting mixture was stirred on ice for 30 min and then 4-dimethylaminopyridine (DMAP; 49 mg, 0.4 mmol, 0.4 eq) and *N,N*-diisopropylethylamine (DIPEA; 435 µL, 323 mg, 2.5 mmol, 2.5 eq) and **S1** (297 mg, 1 mmol, 1 eq) were added. The reaction mixture was stirred at rt overnight then quenched with NH_4Cl (sat., 20 mL) and diluted with EtOAc (80 mL). The organic phase was washed with sat. NH_4Cl (2x30 mL), sat. $NaHCO_3$ (30 mL) and brine (30 mL), dried ($MgSO_4$), filtered and solvents were removed *in vacuo*. The residue was purified by automated column chromatography (Biotag SNAP 25g cartridge 0-10% over 10 CV MeOH in CH_2Cl_2) yielding **5** (266 mg, 0.91 mmol, 91%) as a brown oil.
^{1}H NMR (400 MHz, $CDCl_3$) d ppm 1.02 (s, 3 H, $C\underline{H}_3CN_2$) 1.25 (s, 12 H, CH_2) 1.69 - 1.82 (m, 2 H, $C\underline{H}_2$) 1.95 - 2.07 (m, 2 H, $C\underline{H}_2$) 3.87 - 4.00 (m, 2 H, $C\underline{H}_2CH$) 5.54 (dt, *J*=18, 2 Hz, 1 H, $C\underline{H}B$) 5.69 (s, 1 H, $N\underline{H}$) 6.55 (dt, *J*=18, 5 Hz, 1 H, $C\underline{H}CHB$); ^{13}C NMR (101 MHz, CDCl3) d ppm 19.9 ($\underline{C}H_3$) 24.7 (Pinacol-$C\underline{H}_3$), 25.6 ($\underline{C}N_2$) 30.5 33.9 42.9 ($\underline{C}H_2$) 83.4 ($(CH_3)_2\underline{C}$) 148.2 ($\underline{C}HB$) 171.1 ($\underline{C}ONH$); LRMS m/z (ESI+): 316.19 $[M+Na]^+$; IR (neat): 3296, 3084, 2980, 2929, 2361, 2341, 1718, 1642, 1550 cm^{-1}; HRMS *m/z* (ESI+): Found 316.1806 $(M+Na)^+$; $C_{14}H_{24}BN_3NaO_3$ requires 316.1803.

(E)-4-Benzoyl-*N*-(2-(2-((3-(4,4,5,5-tetramethyl-1,3,2-dioxaborolan-2-yl)allyl)oxy)ethoxy)ethyl)benzamide (6)

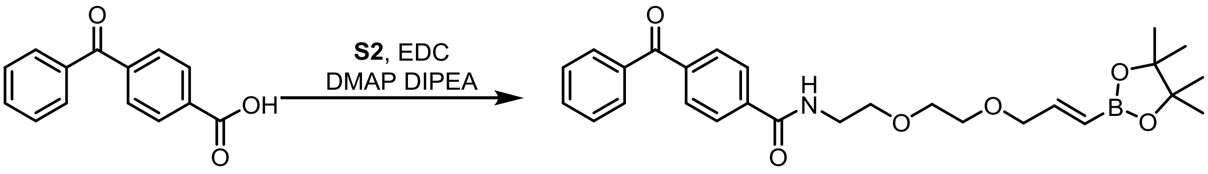

To a solution of *N*-(3-dimethylaminopropyl)-*N*′-ethylcarbodiimide hydrochloride (EDC.HCl; 211 mg, 1.1 mmol, 2.2 eq) in CH_2Cl_2 (10 mL) on ice was added 4-benzoylbenzoic acid (226 mg, 1 mmol, 2 eq). The resulting solution was stirred on ice for 30 min and then 4-dimethylaminopyridine (DMAP; 31 mg, 0.25 mmol, 0.5 eq) and *N,N*-diisopropylethylamine (DIPEA; 304 µL, 226 mg, 1.75 mmol, 3.5 eq) and **S2** (192 mg, 0.5 mmol, 1 eq) were added. The reaction mixture was stirred at rt overnight then quenched with NH_4Cl (sat., 20 mL) and diluted with EtOAc (80 mL). The organic phase was washed with sat. NH_4Cl (2x30 mL), sat. $NaHCO_3$ (30 mL) and brine (30 mL), dried ($MgSO_4$), filtered and solvents were removed *in vacuo*. The residue was purified by automated column chromatography (Biotag SNAP 25g cartridge 0-5% over 10 CV MeOH in CH_2Cl_2) to give **6** (86 mg, 0.18 mmol, 36%)
^{1}H NMR (400 MHz, $CDCl_3$) d ppm 1.22 (s, 12 H, $C\underline{H}_3$) 3.58 - 3.74 (m, 8 H, $C\underline{H}_2$) 4.10 (dd, *J*=5, 2 Hz, 2 H, $C\underline{H}_2CH$) 5.72 (dt, *J*=18, 2 Hz, 1 H, $C\underline{H}B$) 6.62 (dt, *J*=18, 5 Hz, 1 H, $C\underline{H}CHCB$) 6.92 (br. s., 1 H, $N\underline{H}$) 7.45 - 7.53 (m, 2 H, $C\underline{H}CHCCO$) 7.56 - 7.64 (m, 1 H, $C\underline{H}CHCH$) 7.76 - 7.81 (m, 2 H, $C\underline{H}CCO$) 7.83 (m, *J*=8 Hz, 2 H, $C\underline{H}CHCCONH$) 7.90 (m, *J*=8 Hz, 2 H, $C\underline{H}CCONH$); ^{13}C NMR (101 MHz, $CDCl_3$) d ppm 24.7 ($\underline{C}H_3$) 39.8 ($\underline{C}H_2CH$) 69.7 ($\underline{C}H_2$) 69.7 ($\underline{C}H_2$) 70.3 ($\underline{C}H_2$) 72.7 ($\underline{C}H_2$) 83.3 ($\underline{C}CH_3$) 119.7 ($\underline{C}HCHB$) 127.0 ($\underline{C}HCCONH$) 128.4 ($\underline{C}HCHCCO$) 130.1 ($\underline{C}HCHCCONH$ and $\underline{C}HCCO$) 132.8 ($\underline{C}HCHCH$) 137.1 (qAr-$\underline{C}$) 137.8 (qAr-$\underline{C}$) 139.9 (qAr-$\underline{C}$) 148.8 ($\underline{C}HB$) 166.6 ($\underline{C}ONH$) 196.0 ($\underline{C}O$);

LRMS m/z (ESI$^+$): 480.26 [M+H]$^+$; HRMS *m/z* (ESI+): Found 502.2359 (M+Na)$^+$; $C_{27}H_{34}BNO_6Na$ requires 502.2376; IR (neat): 2978, 2361, 1645, 1538 cm^{-1}.

4-((E)-Phenyldiazenyl)-*N*-((E)-3-(4,4,5,5-tetramethyl-1,3,2-dioxaborolan-2-yl)allyl)benzamide (7)

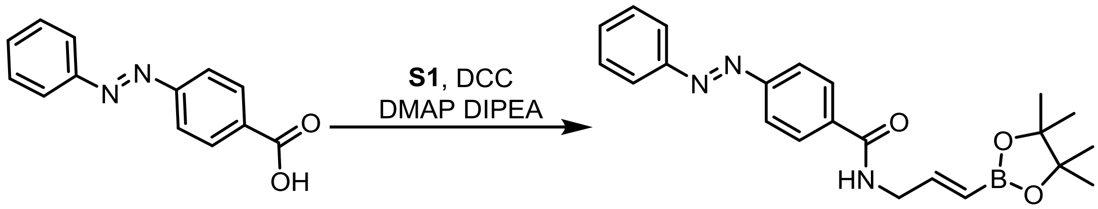

To a solution of 4-(phenyldiazenyl)benzoic acid (226 mg, 1 mmol, 2 eq) in dry Tetrahydrofuran (10 mL) was added *N*-(3-dimethylaminopropyl)-*N′*-ethylcarbodiimide hydrochloride (EDC.HCl; 211 mg, 1.1 mmol, 2.2 eq) at 0°C under N_2. The resulting solution was stirred for 30 min at 0°C and then **S1** (149 mg, 0,5 mmol, 1 eq), *N,N*-diisopropylethylamine (DIPEA; 325 uL, 1.75 mmol, 3.5 eq) and 4-dimethylaminopyridine (DMAP; 25 mg, 0.2 mmol, 0.4 eq) were added. The reaction mixture was allowed to warm to rt and stirred for 16 h. Solvents were removed *in vacuo* and the red oil was taken up in EtOAc (150 mL). The organic layer was washed with sat. NH_4Cl (2 x 50 mL), sat. $NaHCO_3$ (50 mL) and brine (50 mL); dried ($MgSO_4$), filtered and solvents were removed *in vacuo*. The red residue was purified by automated column chromatography (Biotage SNAP 10g; MeOH in CH_2Cl_2; 0% 5CV; 0-5% 10CV) yielding **7** a deep red solid (*trans:cis* ; 7:1; 76 mg, 0.2 mmol, 39%). Values for the minor cis isomer are given in italic. ^{1}H NMR (400 MHz, $CDCl_3$) d ppm 1.20 - 1.32 (m, (12+12x0.15)=14 H, $C\underline{H}_3$) *4.10 - 4.17 (m, (2x0.15) H, $C\underline{H}_2$)* 4.18 - 4.25 (m, 2 H, $C\underline{H}_2$) *5.61 (dt, J=18, 2 Hz, 1x0.15 H, $C\underline{H}$)* 5.68 (dt, *J*=18, 2 Hz, 1 H, $C\underline{H}$) *6.35 (t, J=6 Hz, 1x0.15 H, $N\underline{H}$)* 6.48 (t, *J*=6 Hz, 1 H, $N\underline{H}$) *6.62-6.67 (m, 1x0.15 H, $C\underline{H}$)* 6.65 - 6.76 (m, 1 H, $C\underline{H}$) *6.81 - 6.89 (m, 4x0.15 H, $Ar\underline{H}$) 7.13 - 7.19 (m, 1x0.15 H, $Ar\underline{H}$) 7.22 - 7.29 (m, 2x0.15 H, $Ar\underline{H}$)* 7.45 - 7.59 (m, 3 H, $Ar\underline{H}$) *7.68 (d, J=9 Hz, 2x0.15 H, $Ar\underline{H}$)* 7.88 - 8.00 (m, 6 H, $Ar\underline{H}$); ^{13}C NMR (101 MHz, $CDCl_3$) d ppm 24.7 ($\underline{C}H_3$) 43.4 ($\underline{C}H_2$) 83.4 ($\underline{C}CH_3$) 119.1 ($\underline{C}$H) 120.2 (Ar-$\underline{C}$H (minor)) 120.6 (Ar-$\underline{C}$H (minor)) 122.9 (Ar-$\underline{C}$H (major)) 123.0 (Ar-$\underline{C}$H (major)) 127.7 (Ar-$\underline{C}$H (minor)) 127.9 (Ar-$\underline{C}$H (major)) 128.8 (Ar-$\underline{C}$H (minor)) 129.1 (Ar-$\underline{C}$H (major)) 131.5 (Ar-$\underline{C}$H (major)) 136 (Ar-$\underline{C}$) 148.1 ($\underline{C}$H) 152.4 (Ar-$\underline{C}$) 154.2 (Ar-$\underline{C}$) 166.6 ($\underline{C}$ONH); LRMS m/z (ESI$^+$): 414.18 [M+H]$^+$; HRMS *m/z* (ESI+): Found 414.1960 (M+Na)$^+$; $C_{22}H_{26}BN_3ONa$ requires 414.1963; IR (neat): 3316, 2928, 2360, 2341, 1640, 1541 cm^{-1}.

(E)-*N*-(3-(4,4,5,5-*tetra*-Methyl-1,3,2-dioxaborolan-2-yl)allyl)pyrene-2-carboxamide (10)

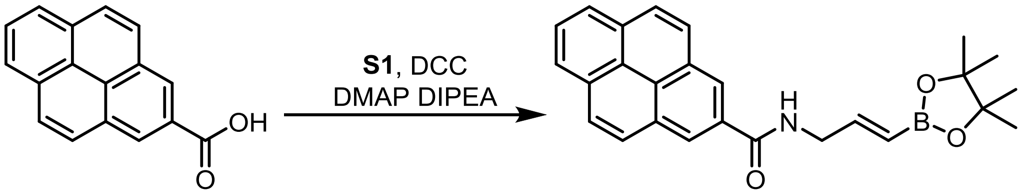

To a solution of *N*-(3-dimethylaminopropyl)-*N′*-ethylcarbodiimide hydrochloride (EDC·HCl, 211 mg, 1.1 mmol, 2.2 eq) in CH_2Cl_2 (10 mL) on ice was added pyrene-2-carboxylic acid (246 mg, 1 mmol, 2 eq). The resulting solution was stirred on ice for 30 min and then 4-dimethylaminopyridine (DMAP; 25 mg, 0.2 mmol, 0.4 eq) and *N,N*-diisopropylethylamine (DIPEA; 304 µL, 226 mg, 1.75 mmol, 3.5 eq) and **S1** (150 mg, 0.5 mmol, 1 eq) were added. The reaction mixture was stirred at rt overnight, then quenched with NH_4Cl (sat., 20 mL) and diluted with EtOAc (80 mL). The organic phase was washed with sat. NH_4Cl (2x30 mL), sat. $NaHCO_3$ (30 mL) and brine (30 mL), dried ($MgSO_4$), filtered and solvents were removed *in vacuo*. The residue was purified by automated column chromatography (Biotag SNAP 25g cartridge 0-5% over 10 CV MeOH in CH_2Cl_2) yielding **10** (120 mg, 0.29 mmol, 58%) as a yellow foam.

^{1}H NMR (400 MHz, $CDCl_3$) d ppm 1.29 (s, 12 H, CH_3) 4.24 - 4.34 (m, 2 H, CH_2) 5.76 (d, *J*=18 Hz, 1 H, CHB) 6.53 (t, *J*=6 Hz, 1 H, NH) 6.78 (dt, *J*=18, 5 Hz, 1 H, CH_2CH) 7.82 - 8.01 (m, 6 H, Ar-H) 8.11 (t, *J*=7 Hz, 2 H, Ar-H) 8.44 (d, *J*=9 Hz, 1 H, Ar-H); ^{13}C NMR (101 MHz, CDCl3) d ppm 24.7 (CH_3) 43.5 (CH_2) 83.3 (CCH_3) 119.2 (CHB) 124.0 (Ar-C) 124.1 (Ar-C) 124.2 (Ar-C) 124.4 (Ar-C) 125.5 (Ar-C) 125.6 (Ar-C) 126.1 (Ar-C) 126.9 (Ar-C) 128.3 (Ar-C) 128.4 (Ar-C) 130.4 (Ar-C) 130.5 (Ar-C) 130.9 (Ar-C) 132.3 (Ar-C) 148.4 (CH_2CH) 169.7 (CONH); LRMS m/z (ESI^+): 434.20 $[M+Na]^+$; HRMS *m/z* (ESI+): Found 434.1888 $(M+Na)^+$; $C_{26}H_{26}BNNaO_3$ requires 434.1903; IR (neat): 2977, 1637, 1534 cm^{-1}.

Small molecule cross-couplings

5-Phenyl-2'-deoxyuridine (1)[5]

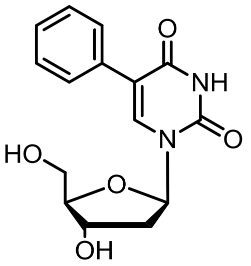

5-Iodo-2'-deoxyuridine (53 mg, 0.15 mmol, 1 eq), phenylboronic acid (28 mg, 0.23 mmol, 1.5 eq) and K_2CO_3 were assembled in a 15 mL vial. 2 mL ddH_2O, 0.5 mL MeCN and Pd***L1** (150 µL of a 0.05 M stock, 7.5 µmol, 5 mol%) were added. The vial was flushed with argon, closed and heated to 55°C for 5 h. The reaction mixture was allowed to cool down to rt, acidified with 2 M HCl (2 drops) and extracted with CH_2Cl_2 (3x10 mL). The combined organic phases were washed with brine, dried over $MgSO_4$ and the solvent was removed *in vacuo*. The crude product was purified by column chromatography (CH_2Cl_2 >9:1 CH_2Cl_2:MeOH) yielding **1** as colorless oil (36 mg, 0.12 mmol, 80%).
^{1}H NMR (400 MHz, CD_3OD) d ppm 2.97 (ddd, *J*=13, 6, 4 Hz, 1 H, $C^{2'}H$) 3.06 (dt, *J*=13, 7 Hz, 1 H, $C^{2'}H$) 4.33 - 4.47 (m, 2 H, $C^{5'}H_2$) 4.63 (q, *J*=3 Hz, 1 H, $C^{4'}H$) 5.06 - 5.13 (m, 1 H, $C^{3'}H$) 5.94 (br. s., 1 H, 5'-OH) 6.08 (br. s., 1 H, 3'-OH) 7.05 (t, *J*=7 Hz, 1 H, $C^{1'}H$) 8.07 - 8.14 (m, 1 H, Ph-H) 8.14 - 8.21 (m, 2 H, Ph-H) 8.32 - 8.40 (m, 2 H, Ph-H) 9.01 (s, 1 H, $C^{6}H$); ^{13}C NMR (101 MHz, CD_3OD) d ppm 49.7 ($C^{1'}H$) 70.6 ($C^{5'}H$) 79.9 ($C^{3'}H$) 94.1 ($C^{1'}H$) 97.2 ($C^{4'}H$) 123.1 (Ar-C) 136.8 (Ph-CH) 137.5 (Ph-CH) 137.8 (Ph-CH) 142.8 (Ar-C) 147.7 ($C^{6}H$) 159.6 (Ar-C) 171.7 (Ar-C) cm^{-1}; LRMS m/z (ESI+): 327.12 $[M+Na]^+$, 343.10 $[M+K]^+$; HRMS *m/z* (ESI+): Found 327.0953 $(M+Na)^+$; $C_{15}H_{16}N_2NaO_5$ requires 327.0951.

N-(2-(2-((E)-3-(1-((2R,4S,5R)-4-hydroxy-5-(hydroxymethyl)tetrahydrofuran-2-yl)-2,4-dioxo-1,2,3,4-tetrahydropyrimidin-5-yl)allyloxy)ethoxy)ethyl)-3-(3-methyl-3H-diazirin-3-yl)propanamide (2)

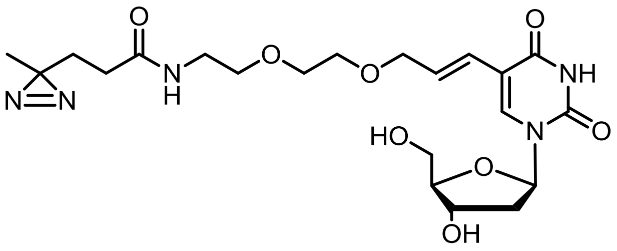

5-Iodo-2'-deoxyuridine (38 mg, 0.1 mmol, 1 eq) and K_2CO_3 (41 mg, 0.3 mmol, 3 eq) were dissolved in 4 mL 3:1 H_2O:MeCN in a 10 mL vial. To the stirred solution was added Pd***L1** (100 µL of a 0.05 M stock, 5 µmol, 5 mol%) and subsequent **4** (57 mg, 0.15 mmol, 1.5 eq). The reaction mixture was stirred at 55°C for 2h after which TLC indicated complete conversion. The solvents were removed *in*

vacuo and the residue was dissolved in MeOH (30 mL), filtered and purified by automated column chromatography (Biotag SNAP 10 g cartridge 0-20% over 20 CV MeOH in CH_2Cl_2) yielding **2** (41 mg, 85 µmol, 85%) as a yellow oil.

^{1}H NMR (400 MHz, CD_3OD) d ppm 1.00 (s, 3 H, $C\underline{H_3}CN_2CH_2$) 1.61 - 1.68 (m, 2 H, $CN_2C\underline{H_2}C\underline{H_2}$) 2.10 (dd, *J*=8, 7 Hz, 2 H, $CN_2C\underline{H_2}C\underline{H_2}$) 2.22 - 2.36 (m, 2 H, $C^{2'}\underline{H_2}$) 3.37 (t, *J*=5 Hz, 2 H, $NC\underline{H_2}CH_2$) 3.54 - 3.58 (m, 2 H, $NCH_2C\underline{H_2}$) 3.63 (s, 4 H, $OC\underline{H_2}C\underline{H_2}O$) 3.77 (dd, *J*=12, 3 Hz, 1 H, $C^{5'}\underline{H_2}$) 3.85 (dd, *J*=12, 3 Hz, 1 H, $C^{5'}\underline{H_2}$) 3.95 (q, *J*=3 Hz, 1 H, $C^{4'}\underline{H}$) 4.12 (dd, *J*=6, 1 Hz, 2 H, $C\underline{H_2}CHCH$) 4.44 (dt, *J*=6, 4 Hz, 1 H, $C^{3'}\underline{H}$) 6.30 (t, *J*=6 Hz, 1 H, $C^{1'}\underline{H}$) 6.38 (d, *J*=16 Hz, 1 H, $CH_2CHC\underline{H}$) 6.57 (dt, *J*=16, 6 Hz, 1 H, $CH_2C\underline{H}$) 8.22 (s, 1 H, $C^6\underline{H}$); ^{13}C NMR (101 MHz, CD_3OD) d ppm 19.9 ($\underline{C}H_3CN_2$) 26.5 ($\underline{C}N_2$) 31.4 ($CN_2\underline{C}H_2\underline{C}H_2$) 31.7 ($CN_2\underline{C}H_2\underline{C}H_2$) 40.6 ($N\underline{C}H_2CH_2O$) 41.8 ($\underline{C}2'$) 62.8 ($\underline{C}5'$) 70.7 ($NCH_2\underline{C}H_2O$) 71.4 ($O\underline{C}H_2\underline{C}H_2O$) 72.1 (C4') 73.2 ($\underline{C}H_2CHCH$) 86.9 ($\underline{C}1'$) 89.2 ($\underline{C}3'$) 112.6 (C5) 125.1 ($CH_2CH\underline{C}H$) 127.9 ($CH_2\underline{C}HCH$) 139.4 ($\underline{C}6$) 151.7 (CO) 164.5 (CO) 174.8 (CO); LRMS m/z (ESI$^+$): 504.24 $[M+Na]^+$ IR (neat): 3400, 3062, 2926, 2871, 2493, 2429, 2361, 2342, 1683, 1644 cm^{-1}; HRMS *m/z* (ESI+): Found 504.2067 $(M+Na)^+$; $C_{21}H_{31}N_5NaO_8$ requires 504.2065.

N-((E)-3-(1-((2R,4S,5R)-4-hydroxy-5-(hydroxymethyl)tetrahydrofuran-2-yl)-2,4-dioxo-1,2,3,4-tetrahydropyrimidin-5-yl)allyl)-3-(3-methyl-3H-diazirin-3-yl)propanamide (3)

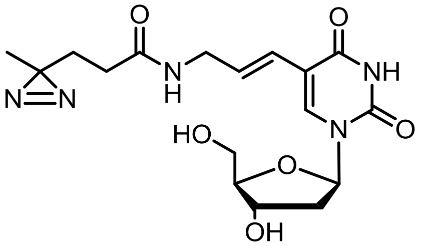

5-Iodo-2'-deoxyuridine (28.3 mg, 0.08 mmol, 1 eq) and K_2CO_3 (33.2 mg, 0.24 mmol, 3 eq) were dissolved in 4 mL 3:1 H_2O:MeCN in a 10 mL vial. To the stirring solution was added Pd***L1** (80 µL of a 0.05 M stock, 4 µmol, 5 mol%) and subsequent **3** (35.2 mg, 0.12 mmol, 1.5 eq). The reaction mixture was stirred at 50°C for 4h. The solvents were removed *in vacuo* and the residue was dissolved in MeOH (30 mL), filtered and purified by automated column chromatography (Biotag SNAP 10 g cartridge 0-20% over 20 CV MeOH in CH_2Cl_2) yielding an inseparable mix of **5c** and starting material (30 mg, 76 µmol, 95%, 60% **3** as determined by NMR) as a yellow oil.

^{1}H NMR (400 MHz, $CDCl_3$) d ppm 1.01 (s, 3 H, $C\underline{H_3}CN_2CH_2$) 1.64 - 1.70 (m, 2 H, $CN_2C\underline{H_2}C\underline{H_2}$) 2.07 - 2.15 (m, 2 H, $CN_2C\underline{H_2}C\underline{H_2}$) 2.18 - 2.34 (m, 4 H, $C^{2'}\underline{H_2}$) 3.35 (s, 1 H) 3.74 (ddd, *J*=12, 7, 3 Hz, 2 H, $C^{5'}\underline{H_2}$) 3.79 - 3.85 (m, 2 H, $C^{5'}\underline{H_2}$) 3.87 (d, *J*=6 Hz, 2 H, $C^{4'}\underline{H}$) 3.93 (q, *J*=3 Hz, 2 H, $C\underline{H_2}CHCH$) 4.38 - 4.44 (m, 2 H, $C^{3'}\underline{H}$) 4.87 (s, 12 H) 6.20 - 6.31 (m, 3 H, $C^{1'}\underline{H}$, $CH_2CHC\underline{H}$) 6.39 - 6.52 (m, 1 H, $CH_2C\underline{H}CH$) 8.15 (s, 1 H, $C^6\underline{H}$) 8.53 (s, 1 H, $C^6\underline{H}$); ^{13}C NMR (101 MHz, $CDCl_3$) d ppm 19.9 ($\underline{C}H_3CN_2$) 31.4 ($CN_2\underline{C}H_2\underline{C}H_2$) 31.7 ($CN_2\underline{C}H_2\underline{C}H_2$) 41.8 ($\underline{C}2'$) 41.9 ($\underline{C}2'$) 42.9 ($\underline{C}H_2CHCH$) 62.6 ($\underline{C}5'$) 62.8 ($\underline{C}5'$) 72.1 (C4') 72.2 (C4') 86.8 (C1') 87.1 (C1') 89.2 ($\underline{C}3'$) 89.2 ($\underline{C}3'$) 112.8 (C5) 124.0 ($CH_2CH\underline{C}H$) 127.8 ($CH_2\underline{C}HCH$) 139.0 (C6) 147.4 (C6) 151.7 (CO) 174.5 (CO); LRMS m/z (ESI$^+$): 432.15 $[M+K]^+$ IR (neat): 3727, 3325, 3068, 2928, 2261, 2341, 1680, 1638, 1548 cm^{-1}; HRMS *m/z* (ESI+): Found 416.1545 $(M+Na)^+$; $C_{17}H_{23}N_5NaO_6$ requires 416.1541

Catalyst preparation

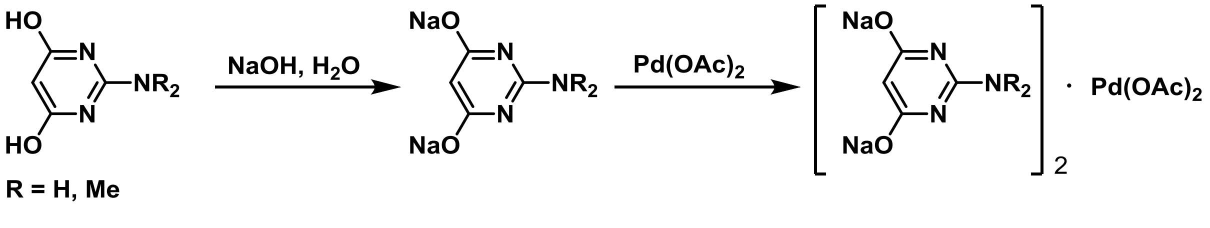

R = H, Me

To 2-aminopyrimidine-4,6-diol (65 mg, 0.5 mmol, 2 eq, R = H) or 2-(dimethylamino)pyrimidine-4,6-diol (78 mg, 0.5 mmol, 2 eq, R = Me) in a 5 mL volumetric flask was added H_2O (3 mL) and NaOH (10 M stock, 100 µL, 1 mmol, 4 eq). The solution was stirred for ~5 min at rt until all solids were dissolved. Then $Pd(OAc)_2$ (55 mg, 0.25 mmol, 1 eq) was added and the flask was stoppered and stirred at 65°C for 60 min. The stirring bar was removed and the solution was adjusted to 5 mL with H_2O to give a 50 mM stock solution.

ODN crosscoupling

General reaction conditions

Unless otherwise noted, 100 µM ODN1-4, 50 mM Tris pH 8.5, 10-50 µM $Pd(OAc)_2$**LX**$_2$ and 10 mM boronic ester were combined in an eppendorf tube and shaken for 4h at 37°C in an incubator. Then the reaction was spun down (2 min, 16000 x g) and the supernatant was analyzed by HPLC and MS. Sometimes precipitation is observed after 24h in the catalyst stock solution. In these cases, probably due to lower actual concentration, the reaction proceeds better with 1 eq (100 µM) $Pd(OAc)_2$**LX**$_2$.

ODN1a

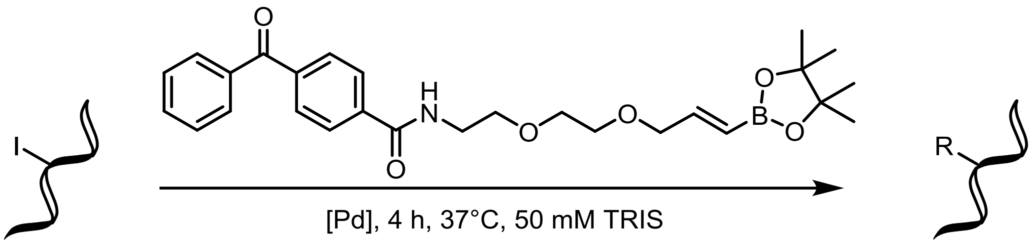

ODN1a elutes at 26.69 min. The peak at 27.29 is a small molecule impurity. Gradient is 5%-20% B in 30 min.

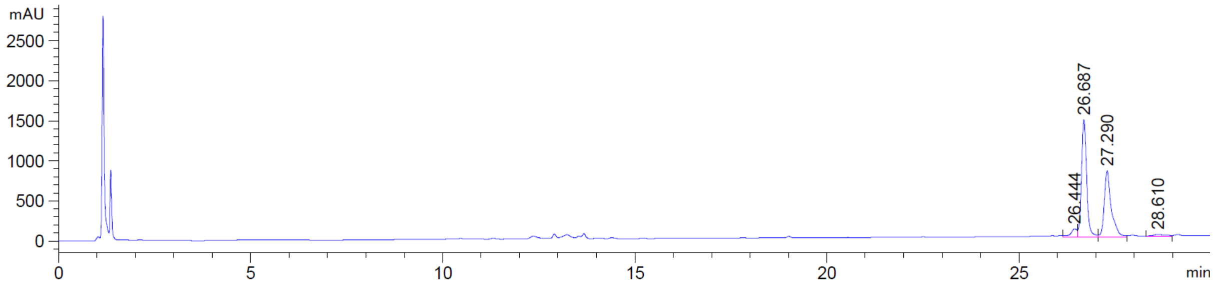

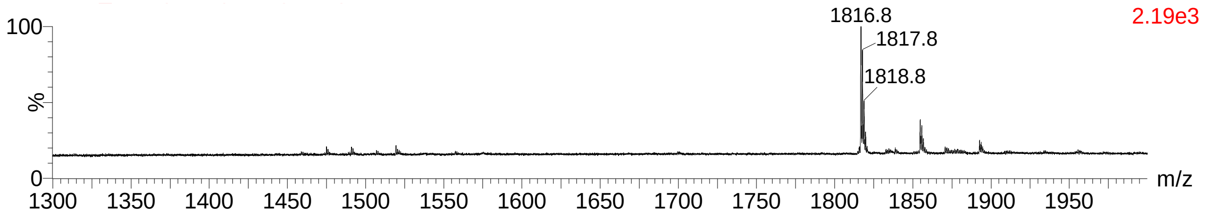

Calc 1816.3 found 1816.8

ODN1b

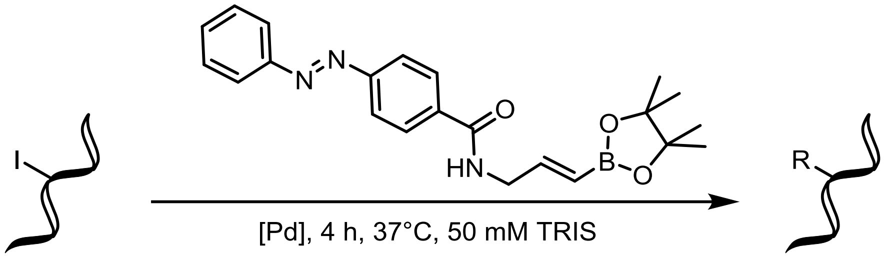

Product elutes at 20.4 min. Gradient is 5%-20% B in 20 min.

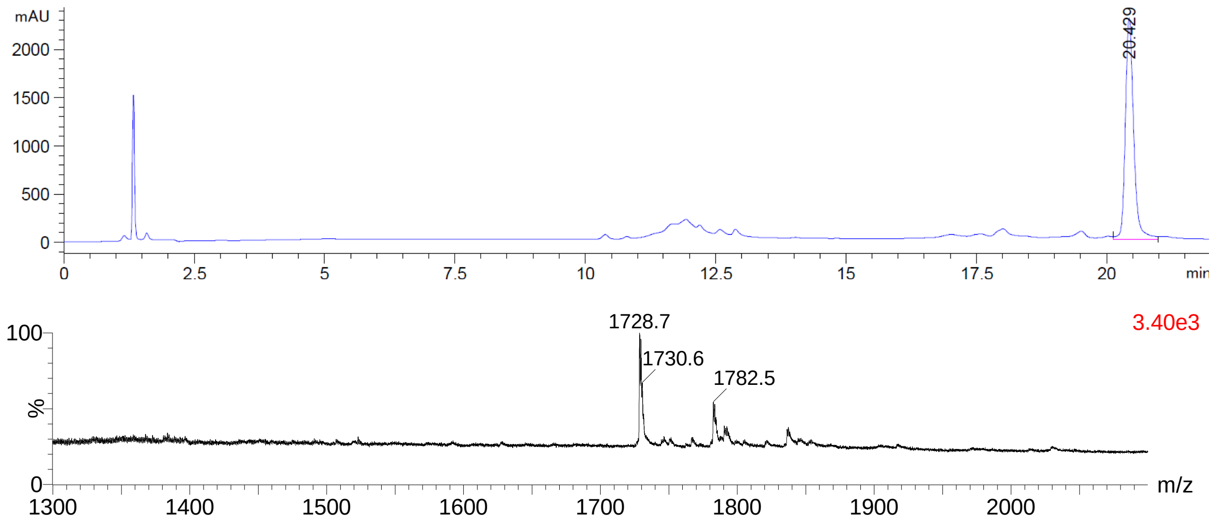

Calc 1728.2 found 1728.7

ODN1c

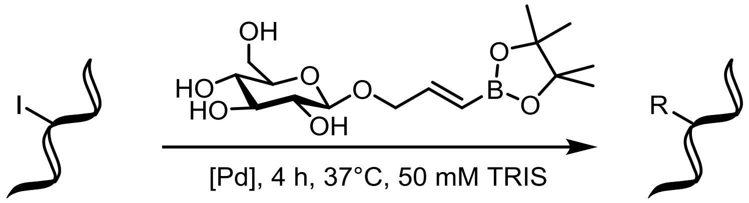

Product elutes at 16 min. Gradient is 0%-30% B in 30 min.

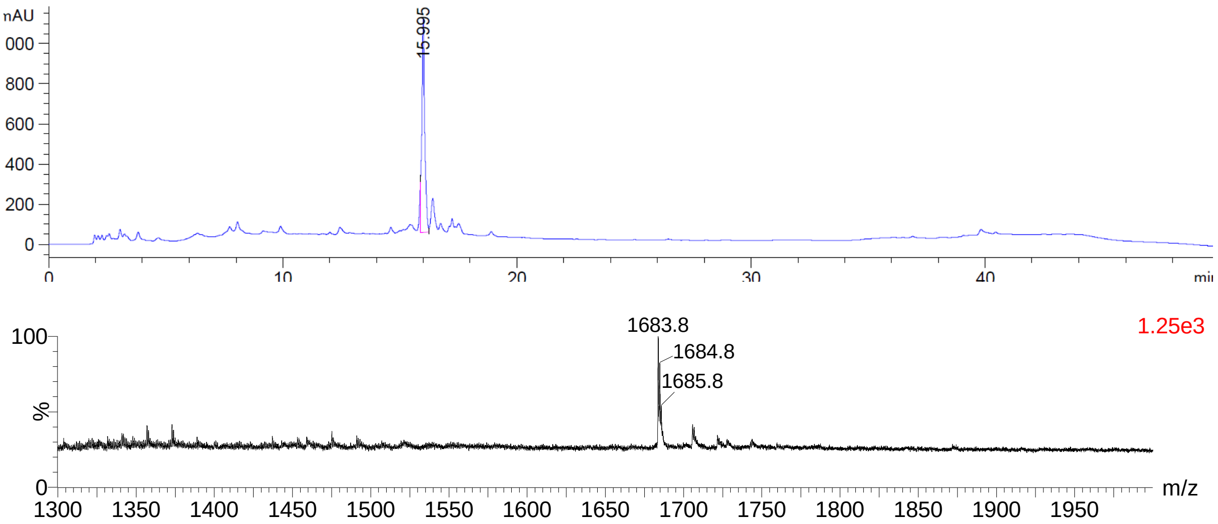

Calc 1683.2 found 1683.8

ODN1d

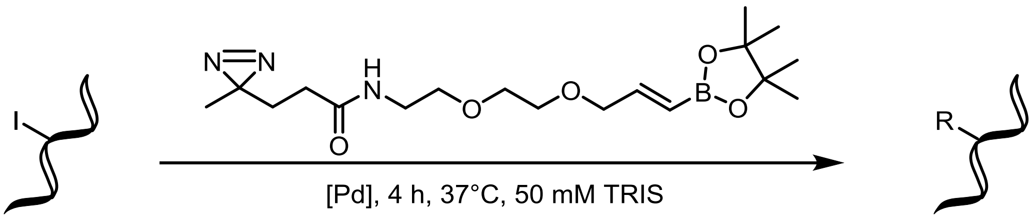

Product elutes at 30.6 min. Gradient is 5%-20% B in 35 min.

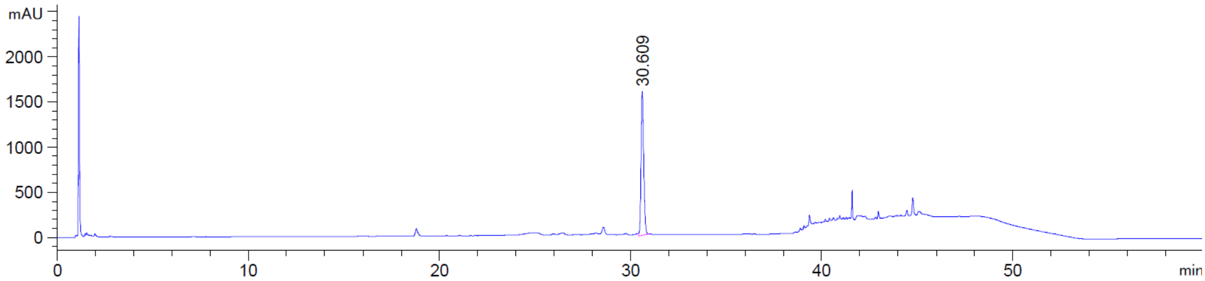

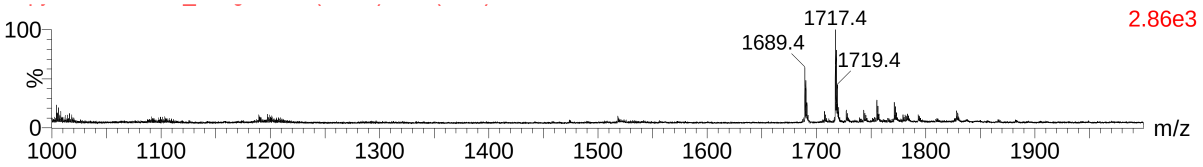

Calc 1718.2 found 1717.4

ODN1e

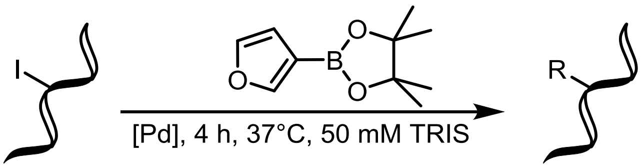

Product elutes at 12.3 min. Gradient is 5%-40% B in 20 min.

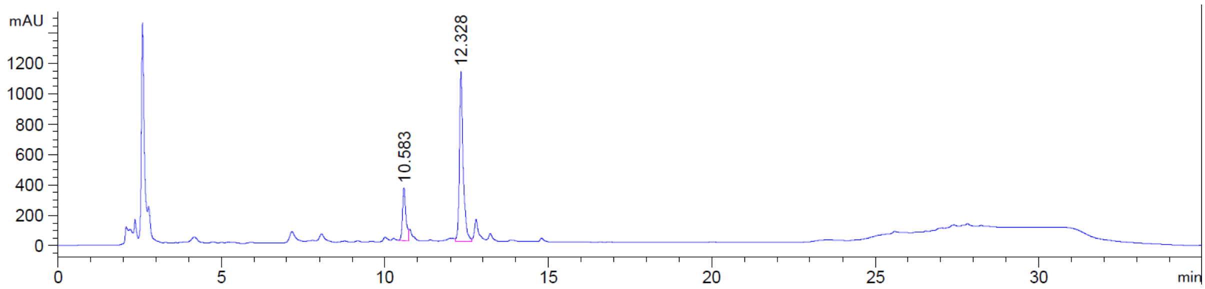

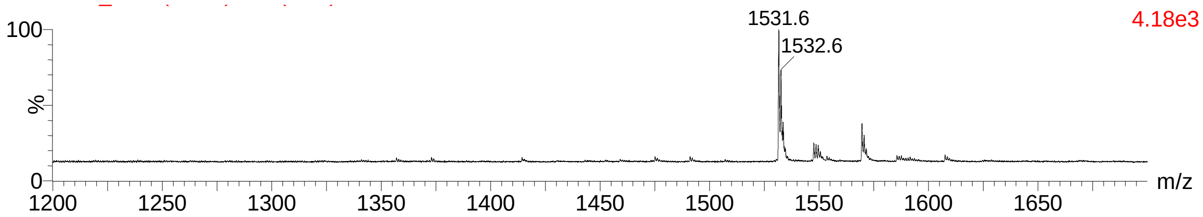

ODN1e reaction with 3-furane boronic acid

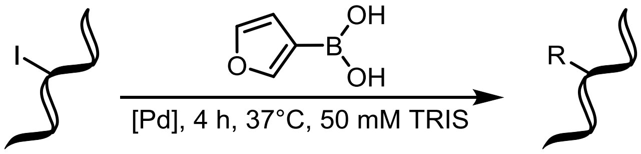

Product elutes at 15.8 min, the product of deiodination at 12.6 min. Gradient is 5%-20% B in 20 min.

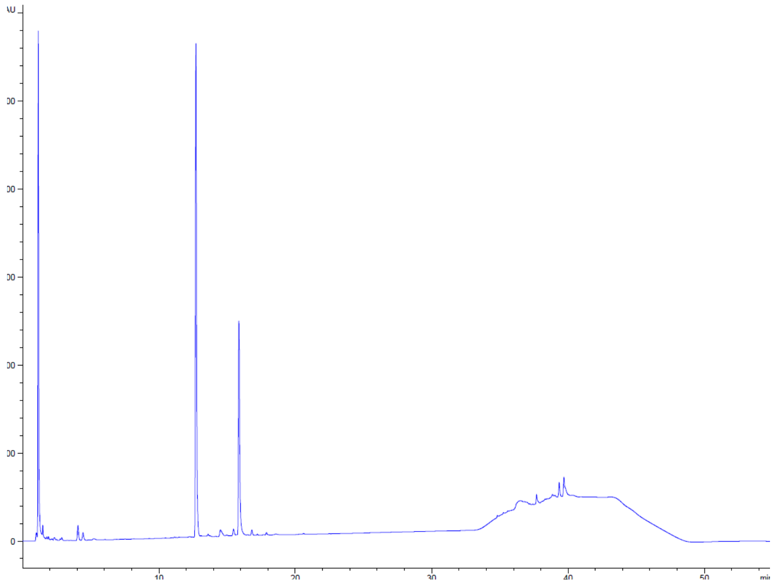

ODN1f

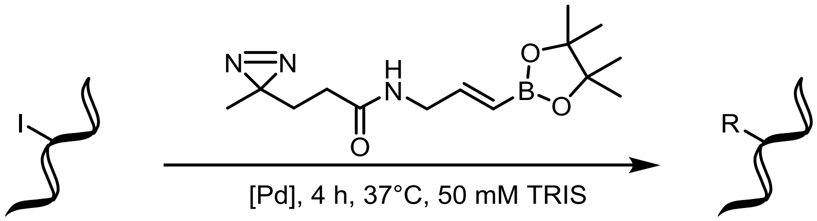

Product elutes after 26 min. Gradient is 5%-20% B in 30 min.

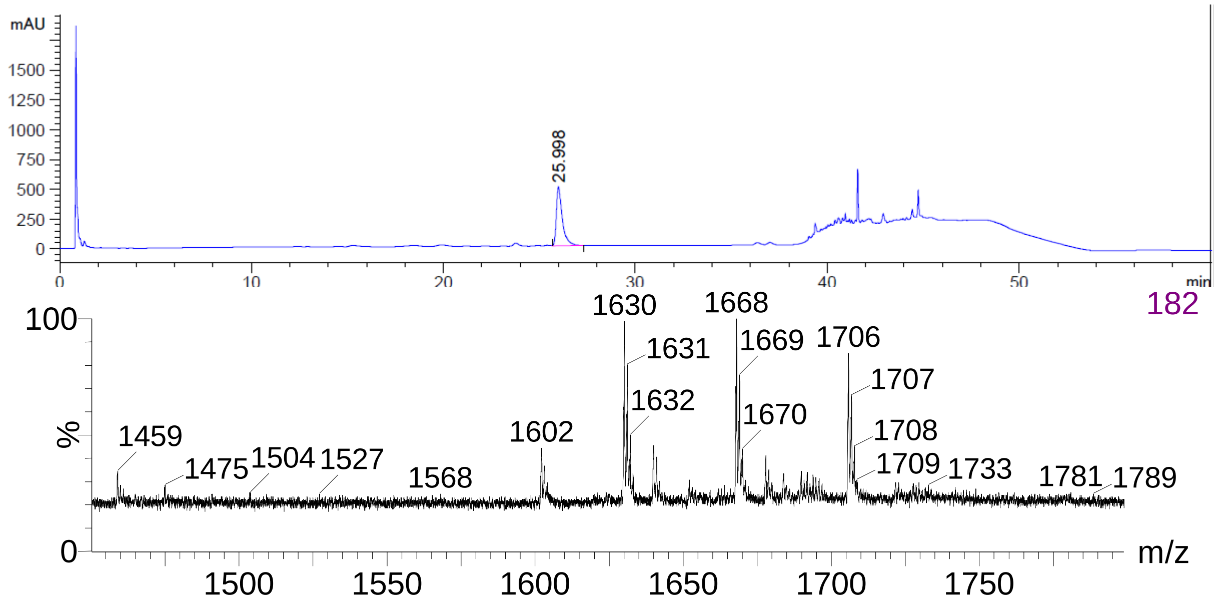

Calc 1630.2 found 1630.3

ODN1g

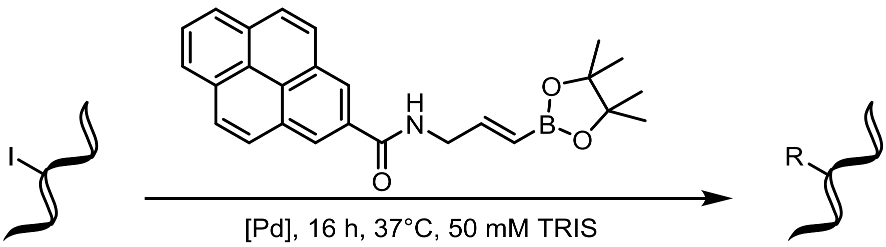

The reaction was performed in 40% MeCN. Product elutes at 27.3 min. Gradient is 5%-30% B in 30 min.

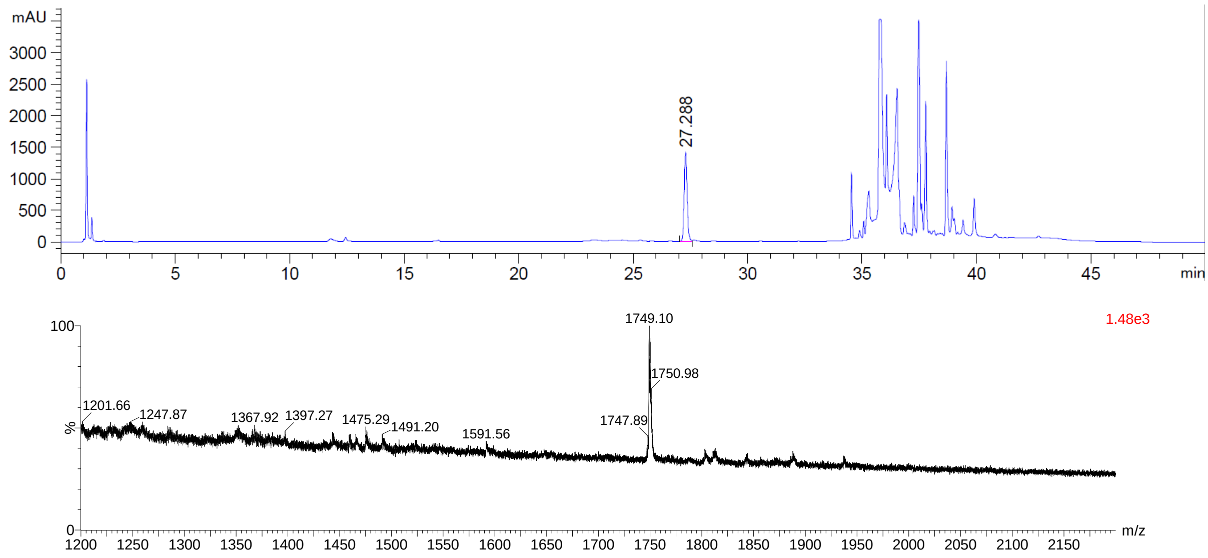

Calc 1748.2 found 1749.1

ODN1 Paladium adduct observed in PO4 buffer

When performing the cross-coupling of **5** to **ODN1d** in phosphate buffer the same species can be observed in the MALDI of the crude reaction mixture.

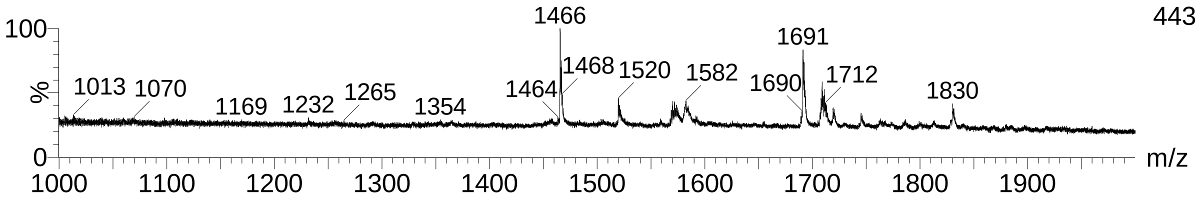

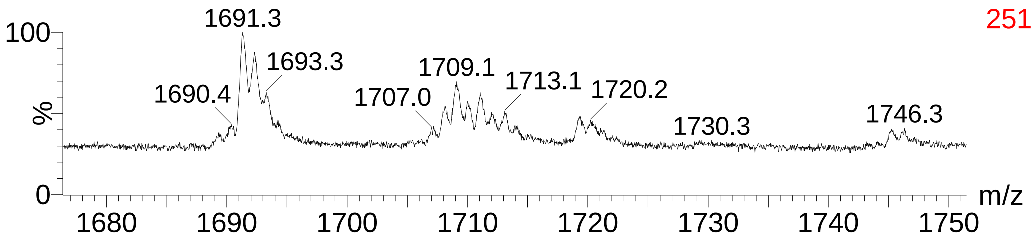

ODN1 reaction intermediates by ESI-MS

The $PdOAc_2$**L1**$_2$ species for this reaction was prepared as describe previously, but substituting NaOH with NH_4OH (33%). The reaction was conducted as described in the general reaction conditions, but using 10 mM NH_4OAc buffer (pH 8.5) and 100 µM Pd $PdOAc_2$**L1**$_2$ (1 eq relative to **ODN1**). The reaction was allowed to proceed for 30 min at 37°C. Then, particulates were removed by centrifugation (2 min, 16k x g) and 10 µL of the reaction mixture were injected into the LCT (ESI-) with the autosampler using a flow of 50:50 H_2O:MeOH (200 µL/min). While more Pd containing species can be observed, these could not be unambiguously identified.

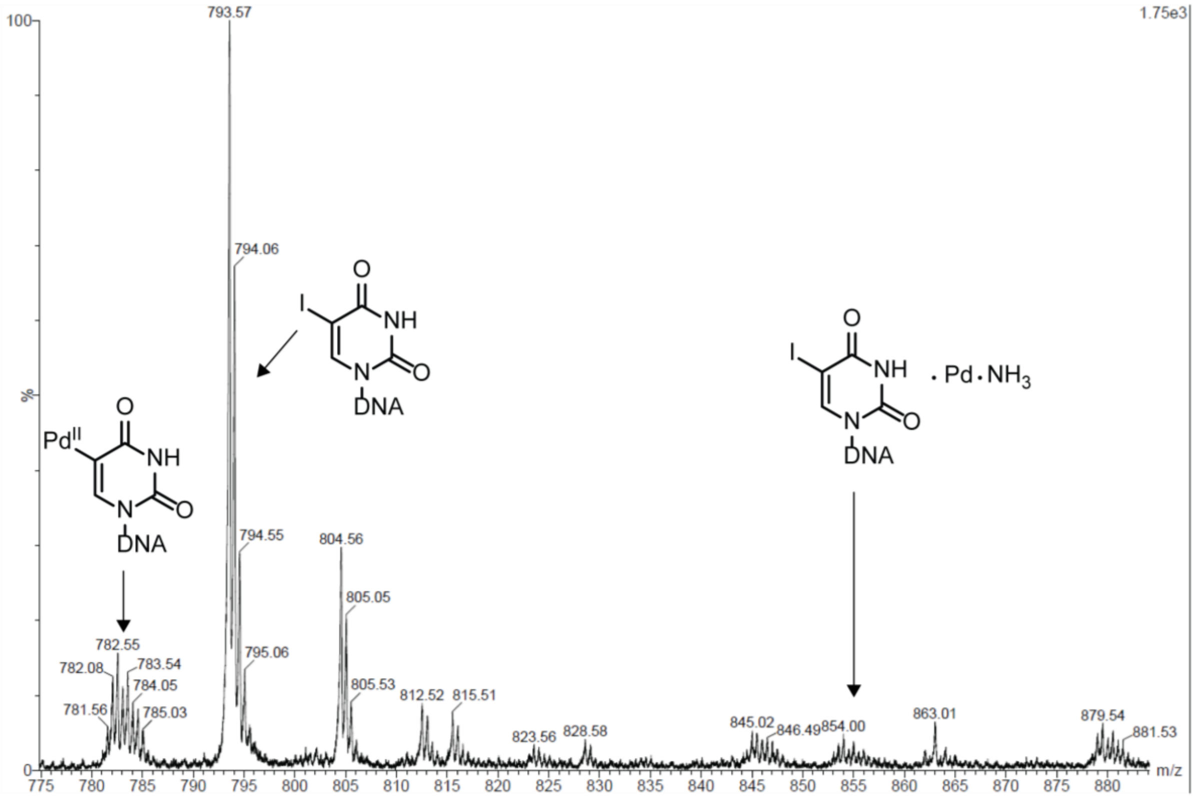

ODN2 (no IdU control)

ODN2 elutes at 31.8 min. Gradient is 0%-20% B in 40 min.

Before reaction:

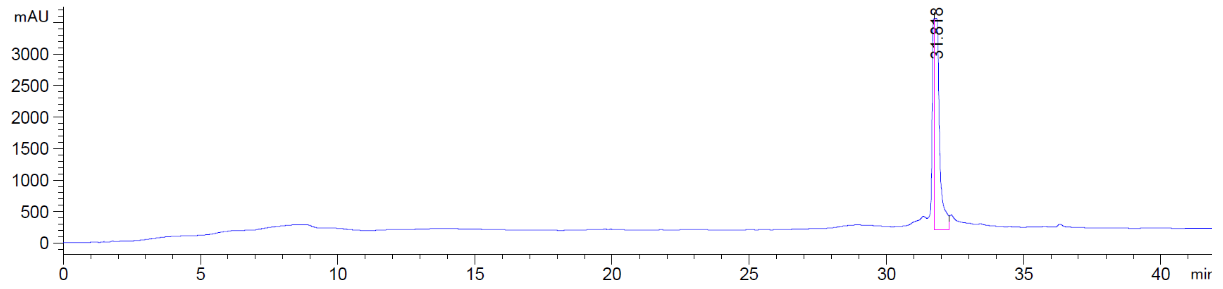

After the reaction:

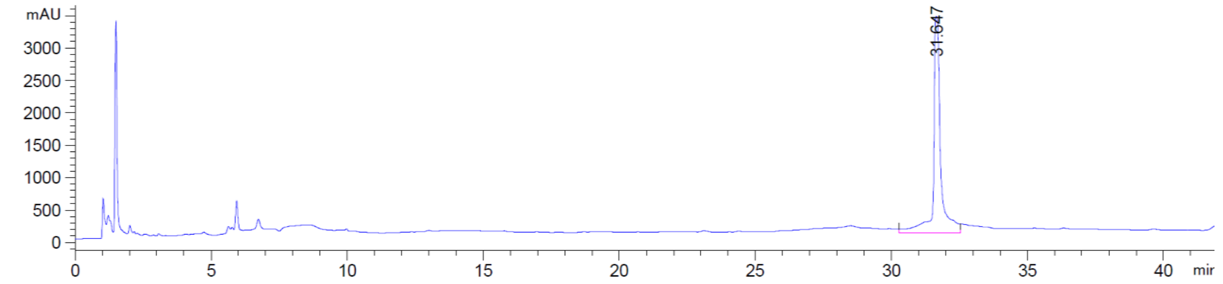

ODN3b
The Product elutes at 18.4 min. ODN was separated on a gradient of 5%-20% B in 20 min.

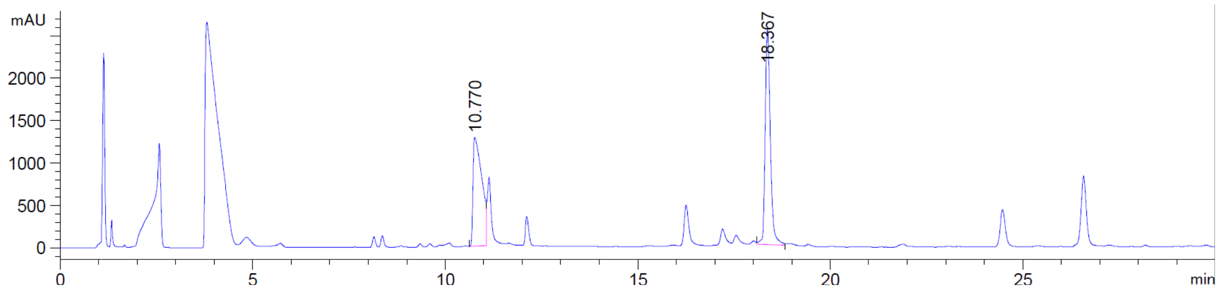

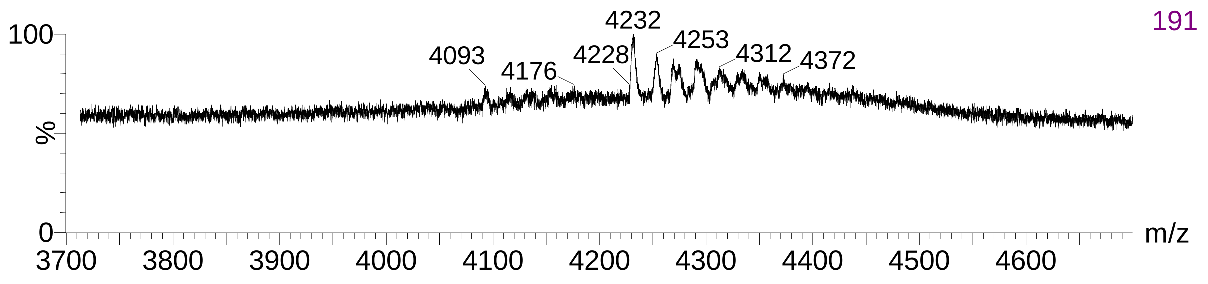

Calc. 4232 found 4232

ODN3c
The Product elutes at 16.9 min. ODN was separated on a gradient of 0%-40% B in 30 min.

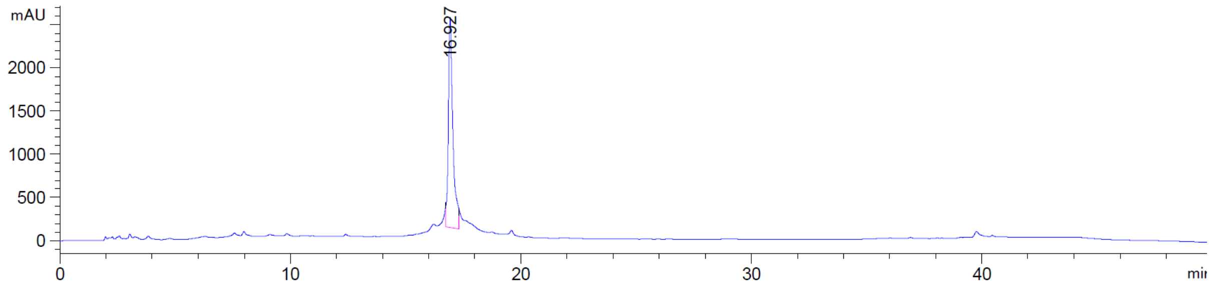

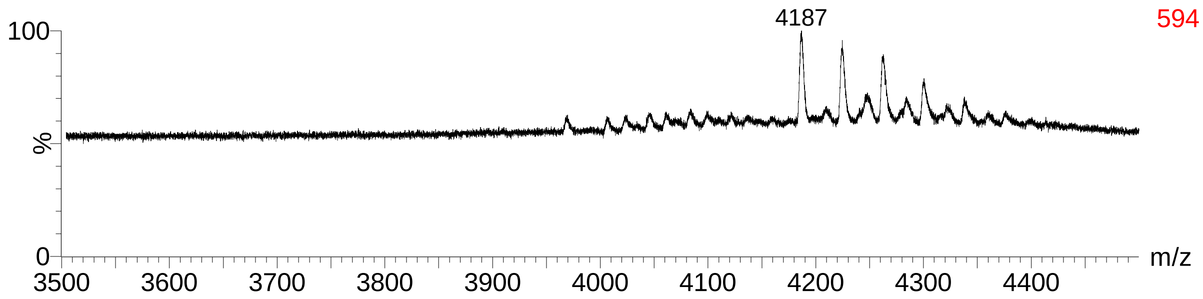

Calc. 4187 found 4187

ODN3d

The Product elutes at 15.3 min. ODN was separated on a gradient of 5%-20% B in 20 min.

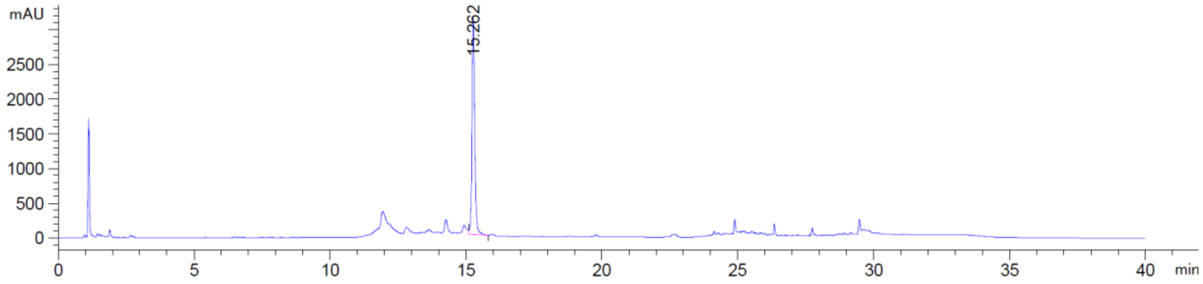

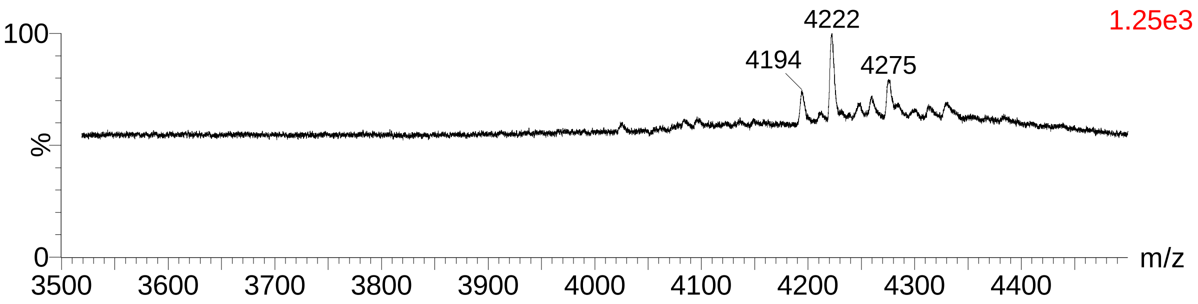

Calc. 4222 found 4222

ODN3f

The Product elutes at 14.2 min. ODN was separated on a gradient of 5%-20% B in 20 min.

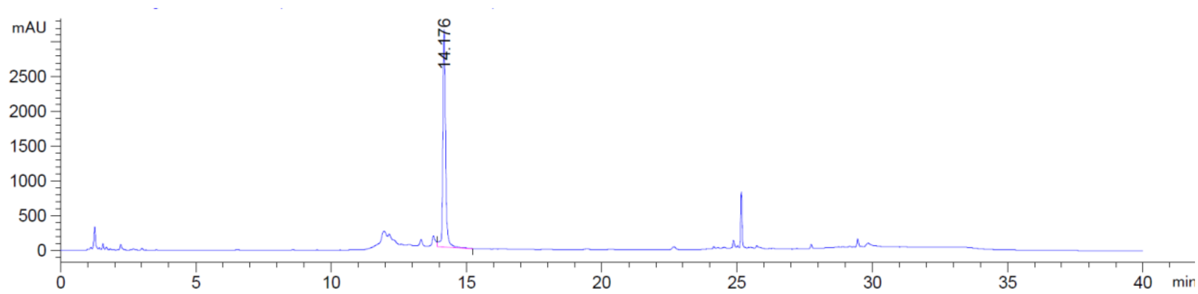

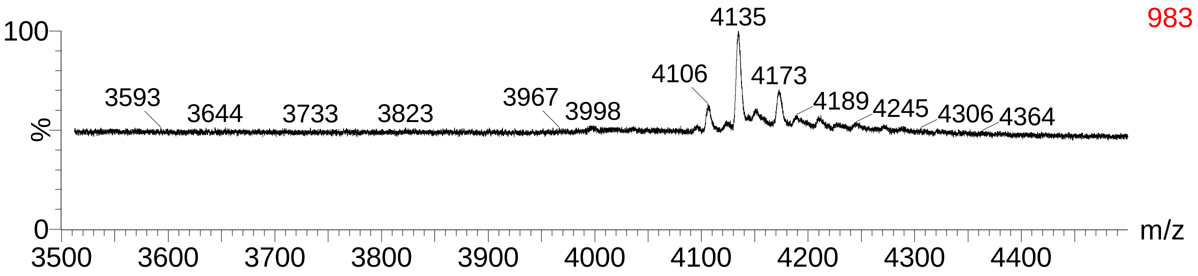

Calc. 4135 found 4135

ODN4 control reactions

Control reactions were set up as described in the general procedure omitting one component where stated. Boronic ester **2** was used for these experiments.

ODN was separated on a gradient of 0%-20% B in 40 min. The starting material elutes at 36.4 min and the product at 40.5 min.

Starting material:

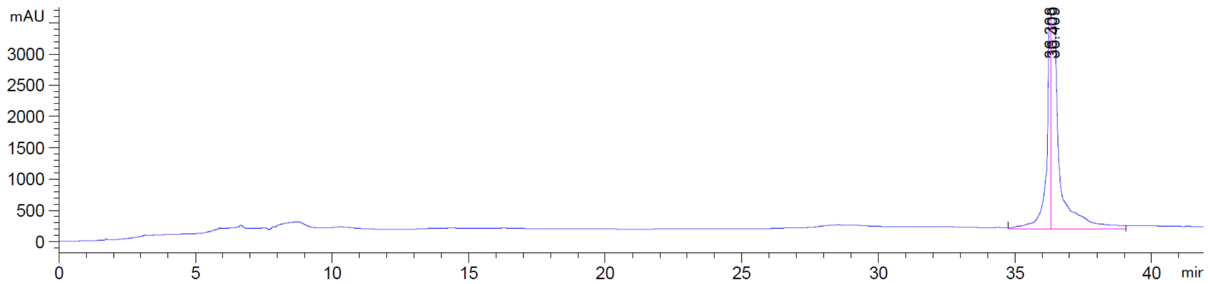

No Boronic ester:

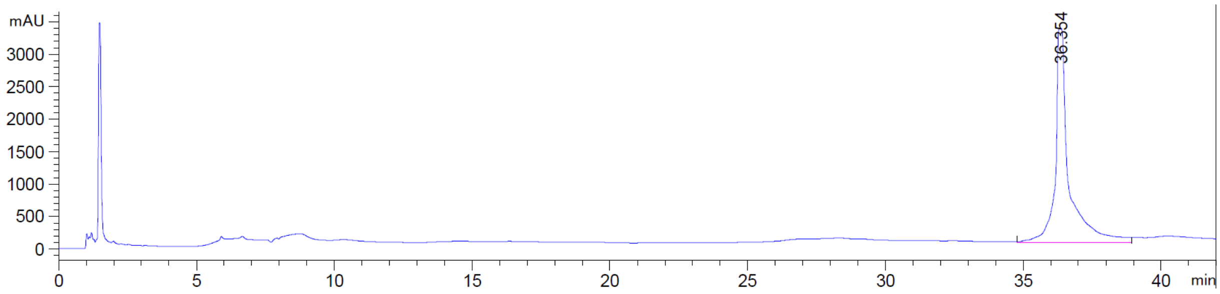

No Palladium:

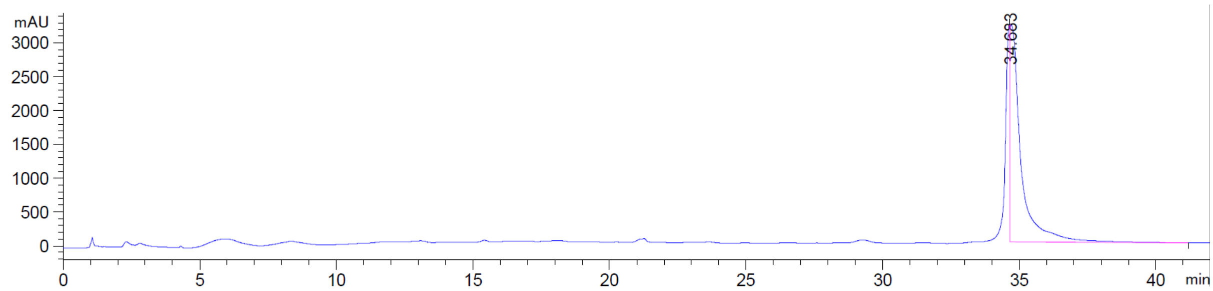

All components:

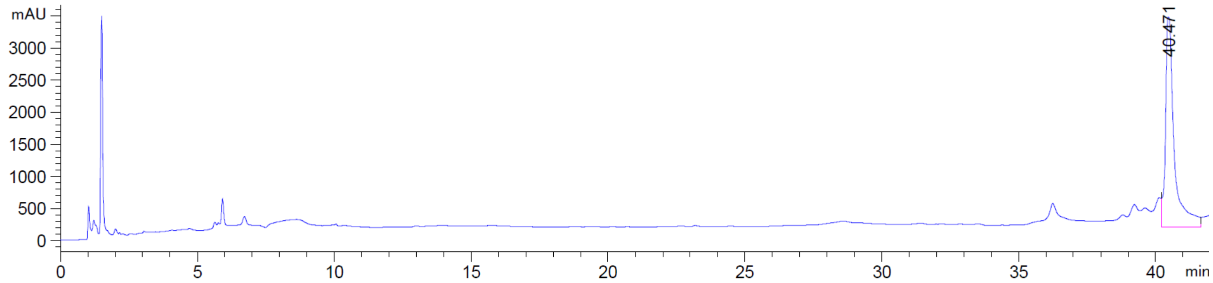

ODN4 – Phosphate and Tris buffer comparison

Reactions were conducted as described in the general procedure but in 100mM PO4 pH 8.5 buffer with Boronic ester **2**. The starting material elutes at 26.2 min and the product at 29.3 min. ODN was separated on a gradient of 0%-30% B in 40 min.

PO4 after 3h:

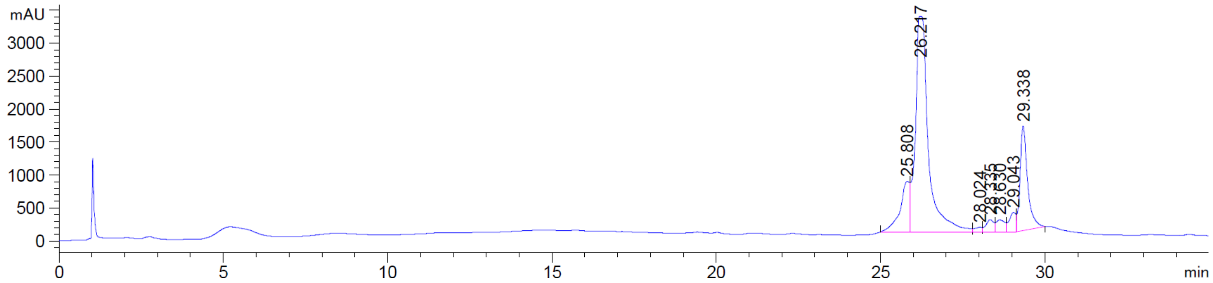

Tris after 3h:

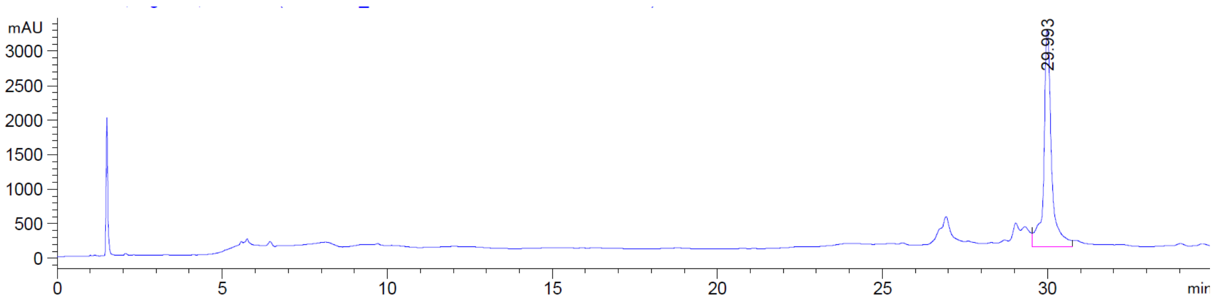

PO4 overnight:

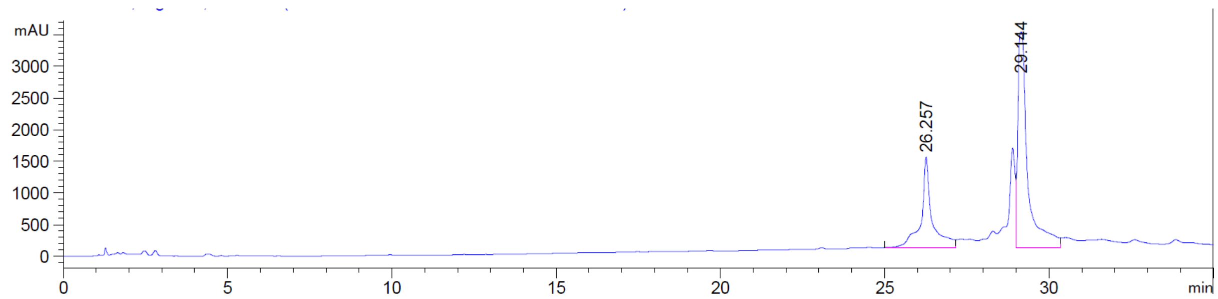

Tris overnight:

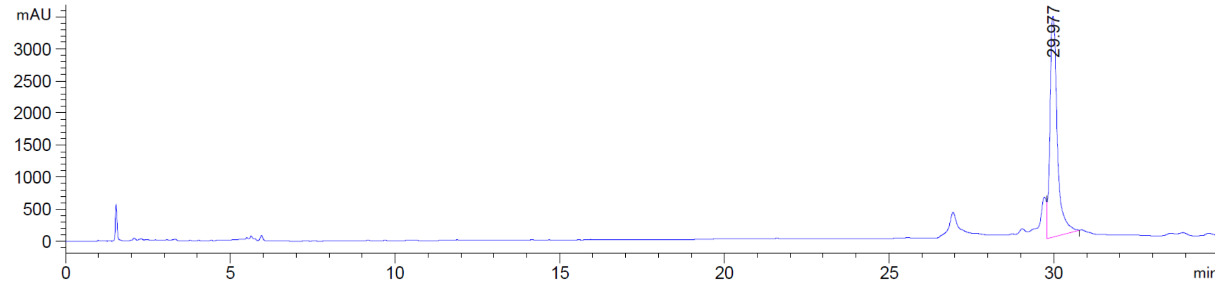

ODN4d

ODN was separated on a gradient of 15%-25% B in 30 min

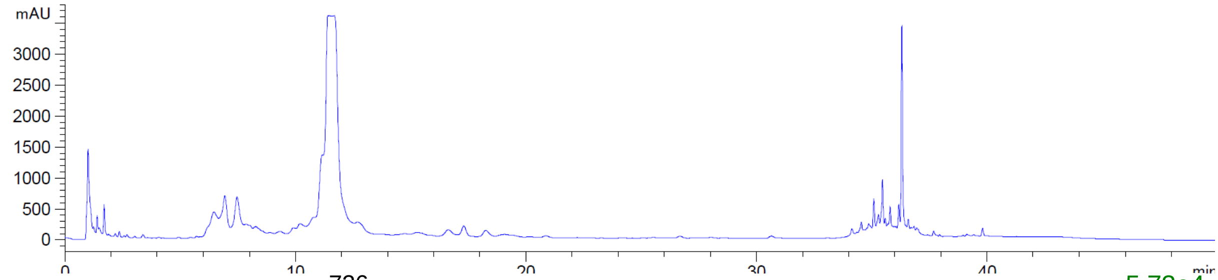

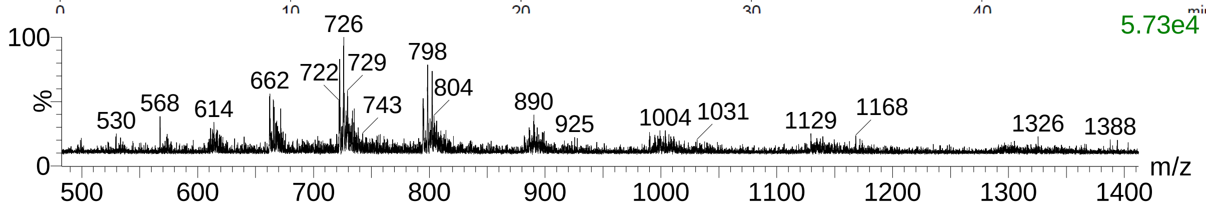

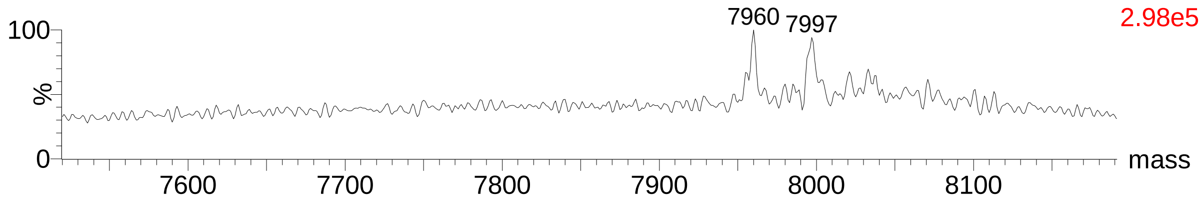

Calc. 7964 found 7960

ODN4f

ODN was separated on a gradient of 0%-20% B in 40 min

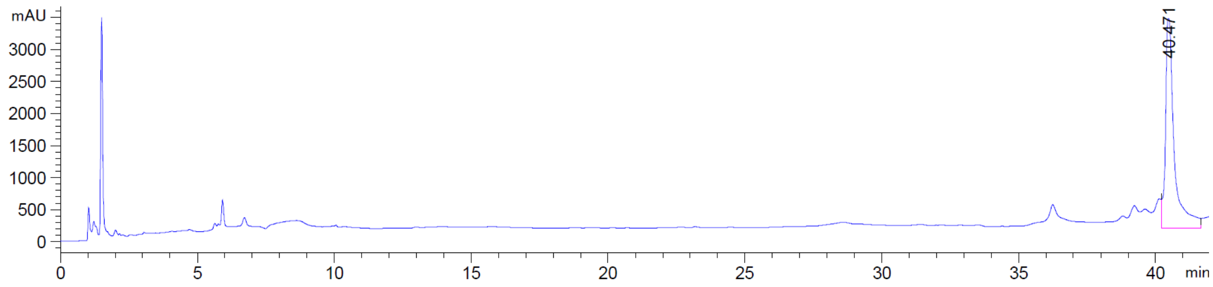

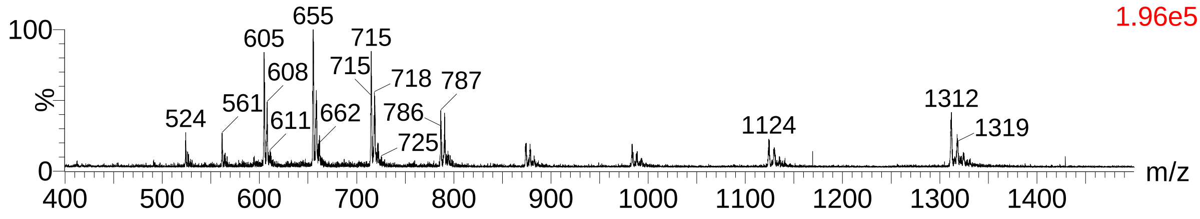

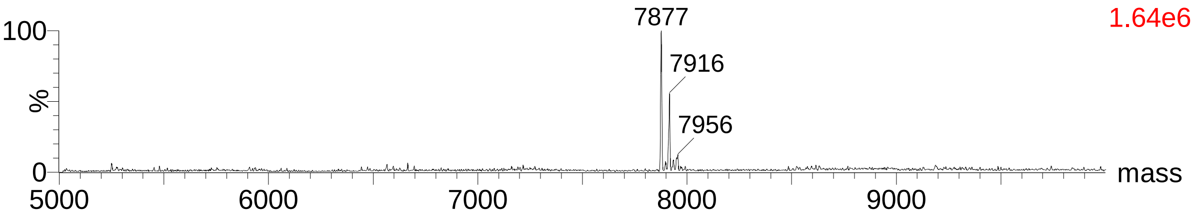

Calc. 7875 found 7877

ODN5f

ODN was separated on a gradient of 10%-30% B in 20 min.

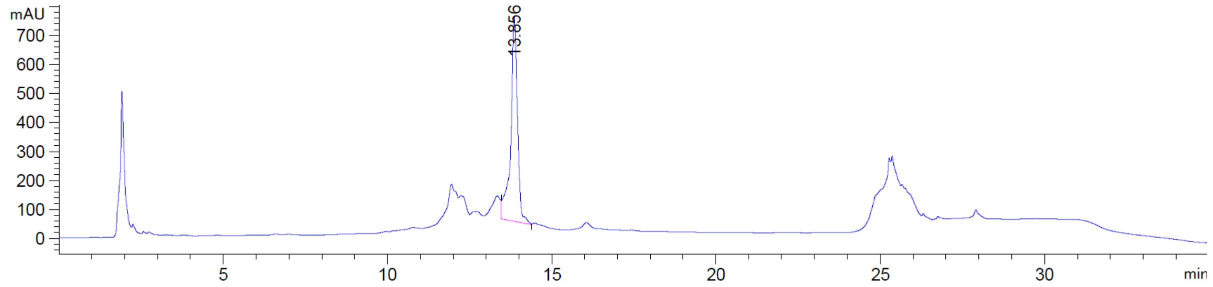

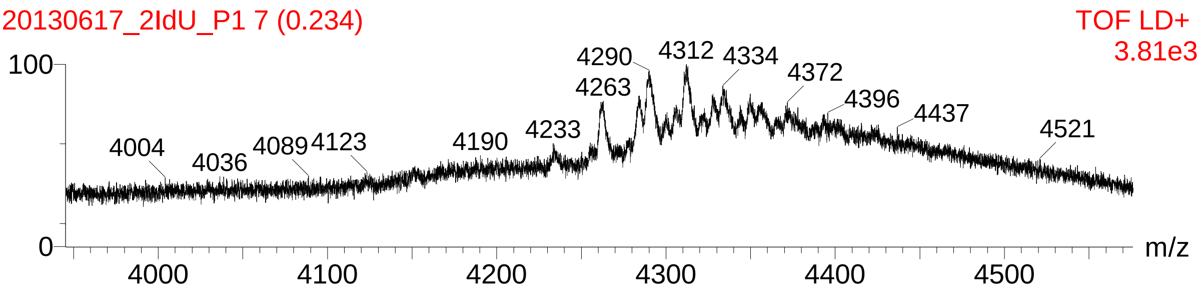

Calc. 4291 found 4290

(other peaks correspond to a Na^+ adduct (4321) or loss of N_2 (4263) and the corresponding Na^+ adduct (4286))

ODN6f

ODN was separated on a gradient of 10%-30% B in 20 min.

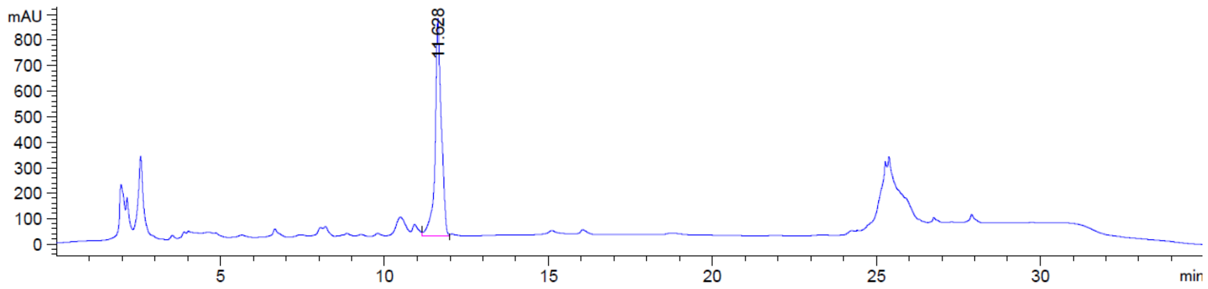

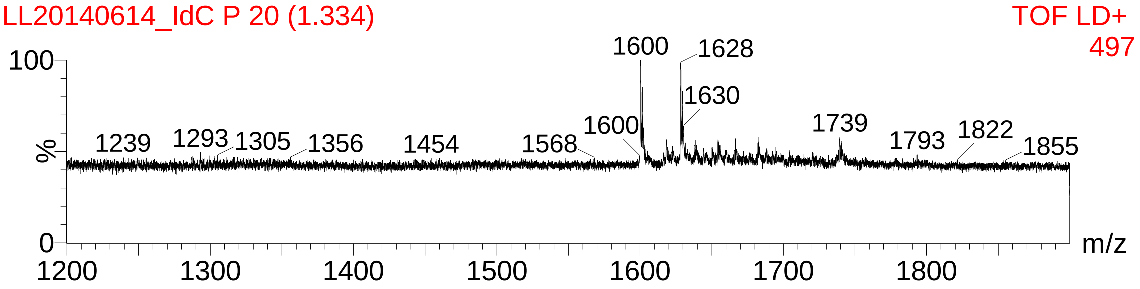

Calc. 1628 found 1628

dsDNA crosscoupling

ODN3 was annealed with the appropriate complementary strand (CTACTGCATCGGT; predicted melting temperature 43°C) by cooling a 150µM solution of each ODN in annealing buffer (10 mM TRIS pH 7.5, 100 mM NaCl) from 90°C to 4°C at 1°C per minute in a PCR block. The resulting dsODN was used as substrate for the crosscoupling reaction under the standard conditions (100 µM dsODN, 100 µM $PdOAc_2$**L2**$_2$**,** 10 mM **5**, 50 mM TRIS pH 8.5) and was shaken at 37°C for 16h. The resulting mixture was analyzed by PAGE (15%, 0.5x TBE; stained with Sybr safe), HPLC (10%-30% buffer B in 20 min) or denaturing HPLC (column was heated at 50°C; 10%-20% B in 30 min). Chromatograms and gel are shown below.

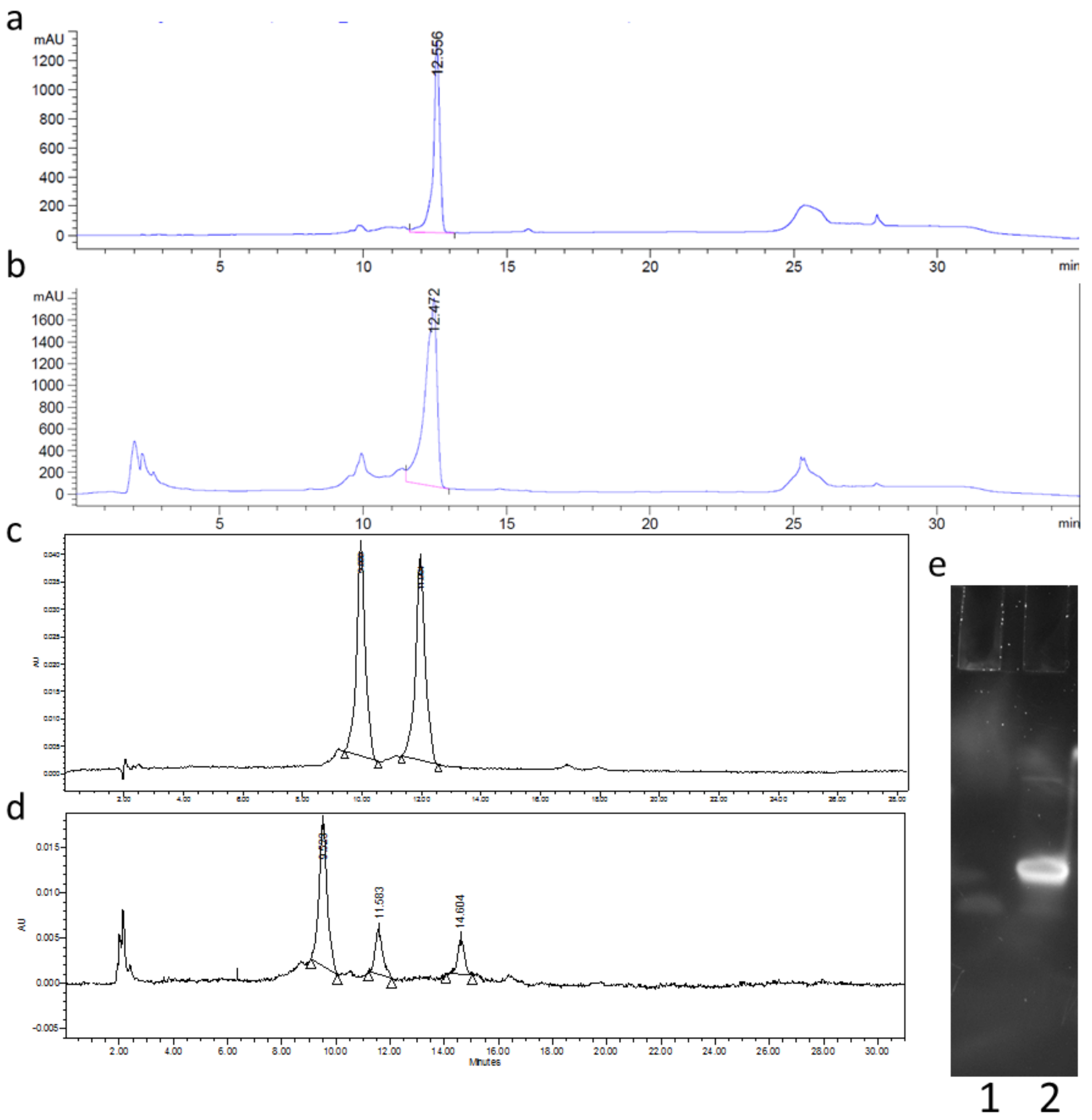

Supplemental Figure S 2. a. HPLC chromatogram of dsODN3 after annealing (retention time: 12.6 min). b. HPLC chromatogram of the crosscoupling reaction on dsODN3. Main peak retention time is 12.5 min, but the peak is broad and a smaller peak with smaller retention time can be observed. c. Denaturing HPLC chromatogram of the dsODN3. 2 Peaks with almost identical area can be observed at retention times of 10 min and 12 min, representing the two ssODN. d. Denaturing HPLC chromatogram of the crosscoupling reaction of dsODN3. 3 main peaks are observed at 9.5 min, 11.5 min and 14.6 min. The peak at 9.5 corresponds to the unreactive ODN, while 11.5 is the IdU containing ODN and 14.6 the crosscoupling product. Yield of the crosscoupling product, when comparing to the unmodified ODN is 21%. e. PAGE gel showing the ssODN3 in lane 1 and dsODN3 after the crosscoupling reaction. The dsODN is largely still hybridized, but a faint band at the hight of the ssODN can be seen, indicating some melting during the reaction.

PCR probe generation

Following template (T1: GCT GCC TGG ACT GAC CTC ACA TGA CCT TAC GCG CAG AGA CCT AGA CAG TGC TTC AGG) and Primers (ODN4d and P2: GCT GCC TGG ACT GAC CTC ACA TGA CC) were used. The PCR was performed with Vent exo^{-} (NEB, M0257S) using the manufacturers recommended buffers and dNTP concentrations. Temperature cycle for the PCR was as follows (55°C, 20 sec; 72°C, 30 sec; 95°C, 15 sec) and was repeated 40x. The PCR product was purified by phenol extraction and ethanol precipitation.

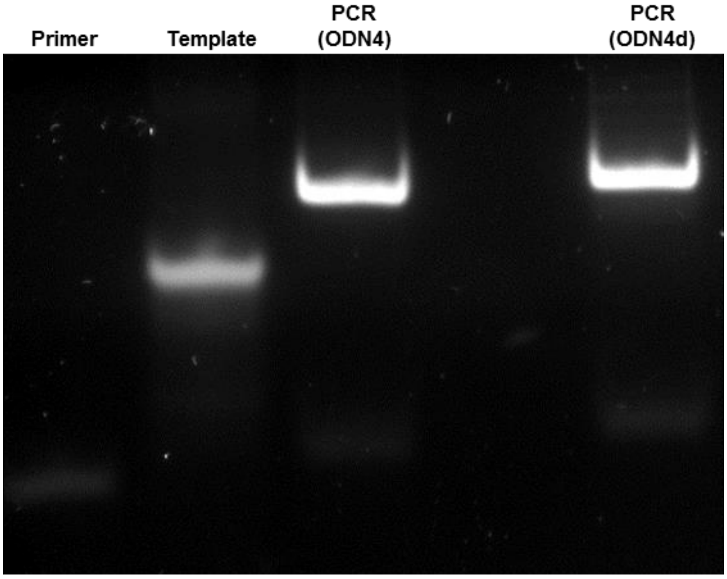

Supplemental Figure S3 PAGE (15%, 0.5% TBE) analysis of the PCR. Primer (lane 1) and template (lane 2; ssDNA) are shown as control. The obtained product molecular weight and yield are not affected by the presence of primer modification (compare lane 3 (IdU; ODN4 **as primer) and lane 4 (**ODN4d **as primer).**

Photocrosslinking

Nuclear extract was prepared using the NE-PER kit (Pierce, 78833) from two confluent 145 mm petri-dishes HeLa cells using the manufacture recommended procedure. Obtained nuclear extract was dialyzed into cross linking buffer (25 mM TRIS pH 7.5, 100 mM NaCl, 1 mM EDTA, 2 mM $MgCl_2$, 0.5 mM DTT, 20% glycerol and one Complete Protease Inhibitor (Pierce, without EDTA) per 50 mL) for 3h. The obtained extract was pre-cleared with Captavidin beads (50 µL, life technology, C-21386), by incubation for 1:30 h at 4°C. The beads were removed by centrifugation and Probes were added (3 nmol, purified from PCR). The mixture was incubated for 20 min on ice and then irradiated for 30 min at 311 nm (caprobox; Caprotec) for silac experiments or at 365 nm using a handheld UV lamp (8W). The solution was adjusted to 1M NaCl and Dynabeads® MyOne™ Streptavidin T1 (200 µL, life technology, 65601) were added. Binding was performed on at room temperature for 1.30 h under constant agitation. The beads were immobilized on DynaMag™- Spin Magnet (life technology, 12320D), and washed with wash buffer (10 mM TRIS, 1 mM EDTA, 1 M NaCl) three times and with wash buffer + 6 M Urea two times. The proteins were released by boiling in Laemli loading buffer for 10 min and separated on a 4-12% gradient gel (NuPAGE® Novex® 4-12% Bis-Tris Gel, Invitrogen). The gel was either visualized by silver stain (Pierce Silver Stain Kit, Thermo Scientific, 24612) or transferred to PVDF membrane using the iBlot (Invitrogen). The membrane was then blocked (3% BSA in TBS-T) for 30 min at room temperature, washed 5 times with TBS-T and then incubated with ExtrAvidin® –Alkaline Phosphatase (Sigma, E2636) for 1h at room temperature. The membrane was then again washed with TBS-T (5x) and water (3x) before visualizing with the BCIP/NBT Liquid Substrate System (Sigma, B1911).

For SILAC experiments the hmC containing probe was incubated and cross-linked with unlabeled cell lysate and the C containing probe with isotope labeled cell lysate (Arg10 and Lys8) for the forward experiment. For the reverse experiment, heavy and light cells were switched. After crosslinking the mixtures were pooled and processed together as described above. After SDS-PAGE the gel was stained with coomassie blue, cut in slices and tryptic digest was performed as described elsewhere (http://www.ccmp.ox.ac.uk/protocols-and-tools).

Streptavidin blot

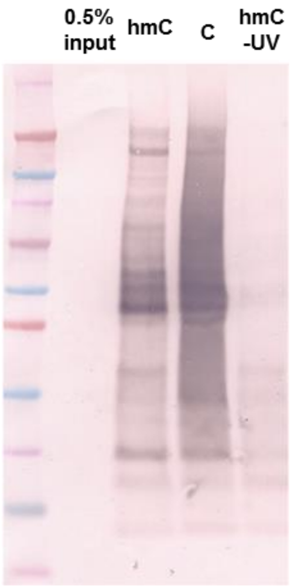

Supplemental Figure S4 Biotin western blot of the photo-crosslinking reaction in HeLa nuclear extract using hmC or C containing probes (lane 2 and 3) and the control without UV irradiation (lane 4). Only upon irradiation covalent DNA-Protein conjugates are observed.

Supplementary Results

SILAC experiments were carried out twice, the first time where the DNA probe containing CTP was incubated with extracts prepared from cells labeled with heavy amino acid isotopes (heavy), whereas extracts from unlabeled cells (light) were incubated with the dhmCTP containing DNA probe. In the second experiment, extracts prepared from heavy labeled cells were incubated with the hmCTP DNA probe and unlabeled cell extracts with the CTP DNA probe. This allowed the SILAC mass spectrometry analysis to be performed in forward and reverse ratios, and proteins differentially present would be expected to exert inverted abundance ratios between the two SILAC experiments (Supplementary Tables 2 A and B). This strategy allowed the elimination of potential “false positives”, and led to the selection of DNA interacting proteins identified and quantified with complementary abundance ratios (Supplementary Table 1). A number of DNA-binding proteins were identified including p20-CGGBP, p52/p100, DHX 9, DDX3X and LIG3 (Supplementary Table 1). CGG triplet repeat-binding protein 1 (P20-CGGBP) binds sequence-specifically to the double-stranded trinucleotide repeat 5'-d(CGG)-3’.[6] Interestingly, the binding is inhibited by complete or partial cytosine-specific DNA methylation of the binding motif in the promoter of the fragile X mental retardation 1 (FMR1) gene,[7] whilst we observed enrichment of p20-CGGBP in hmC versus C containing DNA probes (Supplementary Table 1).

Supplementary Table 1: DNA interacting proteins enriched by either C or hmC DNA probes

Protein Names	Uniprot	PEP	Ratio H/L Normalized	Ratio H/L Variability [%]	Ratio H/L Count	hmC	Ref
54 kDa nuclear RNA- and DNA-binding protein	Q15233	0.00	1.6	110	166	Heavy	[8]
		0.00	0.7	115	44	Light	
100 kDa DNA-pairing protein	P23246-1	0.00	1.8	112	116	Heavy	[8a]
		0.00	0.3	97	23	Light	
ATP-dependent RNA helicase A	Q08211	0.00	2.0	81	41	Heavy	[9]
		0.00	0.2	238	8	Light	
ATP-dependent RNA helicase DDX3X	O00571	0.00	1.5	111	18	Heavy	[10]
		0.00	0.5	133	10	Light	
DNA ligase 3	P49916-1	0.00	1.3	54	3	Heavy	[11]
		0.00	0.6	90	15	Light	
20 kDa CGG-binding protein	Q9UFW	0.01	1.9	-	1	Heavy	[6]
		0.00	0.4	174	2	Light	

DNA ligase 3 (LIG3) is involved in mitochondrial DNA maintenance, interacts with DNA-repair protein XRCC1 and can correct defective DNA strand-break repair and sister chromatid exchange following treatment with ionizing radiation and alkylating agents.[11a, 11d] We observed enriched LIG3 in the presence of hmCTP DNA, indicating a preference for modified nucleotides (Supplementary Table 2).

The ATP-dependent helicases DHX9 and DDX3X were also found to be enriched with hmC containing DNA probes (Supplementary Table 2). Both enzymes unwind DNA and are part of mRNP complexes involved in target gene activation.[9-10, 12]

In addition, we found the two components of the SFPQ-NONO complex, which is a heterotetramer of two 52 kDa (NONO) and two 100 kDa (SFPQ) subunits. NONO is a component of spliceosome and U5.4/6 snRNP complexes. The SFPQ-NONO heteromer may be involved in DNA unwinding by modulating the function of topoisomerase I/TOP1, but also in DNA nonhomologous end joining

(NHEJ) required for double-strand break repair and V(D)J recombination and may stabilize paired DNA ends.[8]

Nothing is known for these proteins identified in our SILAC screen in terms of specific recognition of hmC modifications, but our results indicate potential functional connections that warrant further investigation in hmC associated DNA modification biology, in particular in CpG islands.

[1] J. M. Chalker, C. S. C. Wood, B. G. Davis, *Journal of the American Chemical Society* **2009**, *131*, 16346-16347.

[2] G. A. Molander, F. Cadoret, *Tetrahedron Letters* **2011**, *52*, 2199-2202.

[3] aY. Lee, H. Jang, A. H. Hoveyda, *Journal of the American Chemical Society* **2009**, *131*, 18234-18235; bY. D. Wang, G. Kimball, A. S. Prashad, Y. Wang, *Tetrahedron Letters* **2005**, *46*, 8777-8780.

[4] aR. F. R. Church, M. J. Weiss, *The Journal of Organic Chemistry* **1970**, *35*, 2465-2471; bY. Ikeda, E. J. Behrman, *Synth. Commun.* **2008**, *38*, 2276-2284; cL. Balas, T. Durand, S. Saha, I. Johnson, S. Mukhopadhyay, *Journal of Medicinal Chemistry* **2009**, *52*, 1005-1017; dM. Henze, O. Kreye, S. Brauch, C. Nitsche, K. Naumann, L. A. Wessjohann, B. Westermann, *Synthesis* **2010**, *2010*, 2997,3003.

[5] E. C. Western, J. R. Daft, E. M. Johnson, P. M. Gannett, K. H. Shaughnessy, *The Journal of Organic Chemistry* **2003**, *68*, 6767-6774.

[6] H. Deissler, M. Wilm, B. Genc, B. Schmitz, T. Ternes, F. Naumann, M. Mann, W. Doerfler, *The Journal of biological chemistry* **1997**, *272*, 16761-16768.

[7] H. Muller-Hartmann, H. Deissler, F. Naumann, B. Schmitz, J. Schroer, W. Doerfler, *The Journal of biological chemistry* **2000**, *275*, 6447-6452.

[8] aW. W. Zhang, L. X. Zhang, R. K. Busch, J. Farres, H. Busch, *The Biochemical journal* **1993**, *290 (Pt 1)*, 267-272; bC. L. Bladen, D. Udayakumar, Y. Takeda, W. S. Dynan, *The Journal of biological chemistry* **2005**, *280*, 5205-5210.

[9] S. Zhang, F. Grosse, *The Journal of biological chemistry* **1997**, *272*, 11487-11494.

[10] A. Garbelli, S. Beermann, G. Di Cicco, U. Dietrich, G. Maga, *PloS one* **2011**, *6*, e19810.

[11] aY. F. Wei, P. Robins, K. Carter, K. Caldecott, D. J. Pappin, G. L. Yu, R. P. Wang, B. K. Shell, R. A. Nash, P. Schar, et al., *Molecular and cellular biology* **1995**, *15*, 3206-3216; bJ. Chen, A. E. Tomkinson, W. Ramos, Z. B. Mackey, S. Danehower, C. A. Walter, R. A. Schultz, J. M. Besterman, I. Husain, *Molecular and cellular biology* **1995**, *15*, 5412-5422; cA. W. Kulczyk, J. C. Yang, D. Neuhaus, *Journal of molecular biology* **2004**, *341*, 723-738; dY. Gao, S. Katyal, Y. Lee, J. Zhao, J. E. Rehg, H. R. Russell, P. J. McKinnon, *Nature* **2011**, *471*, 240-244.

[12] T. Nakajima, C. Uchida, S. F. Anderson, C. G. Lee, J. Hurwitz, J. D. Parvin, M. Montminy, *Cell* **1997**, *90*, 1107-1112.

Supplementary Table 2

ODN-to-protein conjugates identified by tandem mass spectrometry and quantified using SILAC. (A) hmC=light; C=heavy; (B) hmC=light; C=heavy. Protein accession numbers are given for the Uniprot database; PEP: posterior error propability; H: heavy; L: light.

See associated excel update spreadsheet.

Supplementary Table 2 A: ODN-to-protein conjugates identified by tandem mass spectrometry and quantified using SILAC (hmC=light; C=heavy)

Protein Names	Gene Names	Protein Descriptions	Uniprot	PEP	Ratio H/L	Ratio H/L Normalized	Ratio H/L Variability [%]	Ratio H/L Count	log2(H/L)
Androgen receptor coactivator 160 kDa protein;Androgen receptor-associated protein of 160 kDa;TATA elem	ARA160;TMF1	Isoform 2 of TATA elem	P82094-2;P82094;	0.069813	0.0077013	0.0058149		1	-7.42603
Putative zinc finger protein		Putative zinc finger pro	Q9P0K0	0.0088382	0.0066263	0.0093325	93.038	2	-6.74352
Coiled-coil domain-containing protein 72	CCDC72;HSPC	Coiled-coil domain-con	Q9Y2S6	0.006873	0.01286	0.018093		1	-5.78842
60S ribosomal protein L24;60S ribosomal protein L30;Putative uncharacterized protein RPL24;Ribosomal pro	RPL24;hCG_2	19 kDa protein;60S ribo	P83731;C9JNW5;C	2.20E-08	0.011647	0.018333	258.07	4	-5.76941
cDNA FLJ54752, highly similar to Poly(rC)-binding protein 2		cDNA FLJ54752, highly	B4DRD7	7.27E-106	0.020091	0.028473		1	-5.13426
Kinesin-like protein KIF14	KIAA0042;KIF	Kinesin-like protein KIF	Q15058	0.089053	0.024161	0.030588		1	-5.03089
		similar to hCG1652800		0.00062443	0.023462	0.035915	10.311	2	-4.79927
Zinc finger protein 646;cDNA FLJ61089, highly similar to Zinc finger protein 646	KIAA0296;ZNF	Isoform 2 of Zinc finger	O15015-2;O15015;	0.088792	0.028676	0.039728	43.717	2	-4.6537
Glyceraldehyde-3-phosphate dehydrogenase, testis-specific;Spermatogenic cell-specific glyceraldehyde 3-ph	GAPD2;GAPD	Glyceraldehyde-3-phos	O14556	6.19E-05	0.031398	0.04366	5.6678	2	-4.51754
High density lipoprotein-binding protein;Vigilin;cDNA, FLJ92652, highly similar to Homo sapiens high density	HBP;HDLBP;V	Vigilin;cDNA FLJ56889,	Q00341;B2R5V9;B	0.0053285	0.033592	0.048823		1	-4.3563
Coding region determinant-binding protein;IGF-II mRNA-binding protein 1;Insulin-like growth factor 2 mRNA	CRDBP;IGF2B	Insulin-like growth fact	Q9NZI8;D3DTW3	0.0018885	0.037391	0.050814		1	-4.29863
Insulin-like growth factor-binding protein 3;Insulin-like growth factor binding protein 3;Insulin-like growth fa	IBP3;IGFBP3;	insulin-like growth fact	P17936;A6XND0;A	1.28E-07	0.037068	0.052608	121.56	6	-4.24857
Enaptin;Myocyte nuclear envelope protein 1;Nesprin-1;Nuclear envelope spectrin repeat protein 1;Synaptic	C6orf98;KIAA	Isoform 1 of Nesprin-1	Q8NF91-1;Q8NF91	0.010116	0.034683	0.055904	18.598	2	-4.1609
High mobility group protein 2;High mobility group protein B2;High-mobility group box 2	HMG2;HMGB	High mobility group pro	P26583;Q5U071;D	2.18E-14	0.0424	0.067693	192.31	4	-3.88485
High mobility group nucleosome-binding domain-containing protein 1;Non-histone chromosomal protein H	HMG14;HMG	Non-histone chromoso	P05114;Q3B790;A	5.57E-15	0.04643	0.069726		1	-3.84216
AU-rich element RNA-binding protein 1;Heterogeneous nuclear ribonucleoprotein D0;cDNA FLJ54150, highl	AUF1;HNRNP	Isoform 2 of Heteroger	Q14103-2;Q14103;	5.37E-118	0.044941	0.070552		1	-3.82517
Dystrophin;DMD protein;Duchenne muscular dystrophy gene;Duchenne muscular dystrophy protein;Dystro	DMD;GS1-19	Isoform 4 of Dystrophi	P11532-1;P11532;	0.0089536	0.055105	0.07077		1	-3.82072
AU-rich element RNA-binding protein 1;Heterogeneous nuclear ribonucleoprotein D0	AUF1;HNRNP	Isoform 1 of Heteroger	Q14103-1;Q14103;	7.97E-175	0.05491	0.079821	144.31	69	-3.64709
BOT;Ig mu heavy chain disease protein;Ig mu chain C region;Full-length cDNA clone CS0DD006YL02 of Neur	IGHM	52 kDa protein;Protein	P04220;P01871-1;	1.31E-09	0.067675	0.083606		1	-3.58025
Putative uncharacterized protein RAN;RAN, member RAS oncogene family, isoform CRA_c;Androgen recept	hCG_1744585	26 kDa protein;27 kDa	B5MDF5;P62826;A	0.0094139	0.06453	0.088324		1	-3.50105
cGMP-binding cGMP-specific phosphodiesterase;cGMP-specific 3',5'-cyclic phosphodiesterase;CGMP-specifi	PDE5;PDE5A	Isoform PDE5A1 of cG	O76074-1;O76074;	0.0042421	0.055282	0.089074		1	-3.48885
Signal recognition particle 9 kDa protein;cDNA FLJ76514, highly similar to Homo sapiens signal recognition	SRP9;hCG_17	Signal recognition part	P49458;A8K0N0;Q	2.29E-11	0.067365	0.093878	153.31	3	-3.41307
cDNA FLJ78093, highly similar to Homo sapiens ribosomal protein L29 (RPL29), mRNA;60S ribosomal protei	RPL29;RP11-6	27 kDa protein;26 kDa	A8K0H3;P47914;Q	8.04E-05	0.069116	0.098074	58.325	2	-3.34999
Dermcidin isoform 2;DCD-1;Dermcidin;Preproteolysin;Survival-promoting peptide;Proteolysis inducing facto	DCD;AIDD;DS	Dermcidin isoform 2;D	A5JHP3;P81605;Q5	0.00021482	0.075223	0.10238		1	-3.28799
Nucleolysin TIA-1 isoform p15;Nucleolysin TIA-1 isoform p40;p15-TIA-1;p40-TIA-1;RNA-binding protein TIA-	TIA1	Isoform Short of Nucle	P31483-2;P31483;	9.60E-46	0.065824	0.10291	11.281	2	-3.28054
DEAD box protein 49;Probable ATP-dependent RNA helicase DDX49;cDNA FLJ77346, highly similar to Homo	DDX49	Probable ATP-depende	Q9Y6V7;A8K7A1;Q	0.0012823	0.07964	0.10591		1	-3.23909
Activated RNA polymerase II transcriptional coactivator p15;p14;Positive cofactor 4;SUB1 homolog;cDNA FL	PC4;RPO2TC1	Activated RNA polyme	P53999;B7Z1Z0;Q5	1.37E-37	0.073206	0.10635	129.01	3	-3.23311
Elongation factor 1-alpha 1;Elongation factor Tu;Eukaryotic elongation factor 1 A-1;Leukocyte receptor clust	EEF1A;EEF1A1	Elongation factor 1-alp	P68104;A8K9C4;A	6.02E-27	0.067829	0.10828	139.08	17	-3.20716
Axonemal beta dynein heavy chain 10;Ciliary dynein heavy chain 10;Dynein heavy chain 10, axonemal;DNAH	DNAH10;KIAA	Isoform 1 of Dynein he	Q8IVF4-1;Q8IVF4;	0.087971	0.078248	0.11224		1	-3.15534
CLL-associated antigen KW-7;Dense fine speckles 70 kDa protein;Lens epithelium-derived growth factor;PC4	DFS70;LEDGF;	Isoform 1 of PC4 and S	O75475-1;O75475;	1.79E-115	0.082223	0.11709	120.76	28	-3.09431
Histone H1.3;Histone H1c;Histone H1.4;Histone H1b;Liver histone H1e;Histone 1, H1e;Histone H1e;cDNA, F	H1F3;HIST1H1	Histone H1.3;Histone	P16402;P10412;A3	3.68E-10	0.076807	0.11802	137.53	4	-3.0829
UPF0688 protein C1orf174	C1orf174	UPF0688 protein C1orf	Q8IYL3	0.00041138	0.093767	0.12966		1	-2.94719
ATP-dependent RNA helicase #46;DEAH box protein 15;Putative pre-mRNA-splicing factor ATP-dependent R	DBP1;DDX15;	Putative pre-mRNA-spl	O43143;B4E0S6	0.0050922	0.09722	0.13023	90.035	2	-2.94087
Nucleolar phosphoprotein B23;Nucleolar protein NO38;Nucleophosmin;Numatrin;Truncated nucleolar phos	NPM;NPM1	Isoform 1 of Nucleoph	P06748-1;P06748;	0.012239	0.095961	0.13528		1	-2.88598
Forkhead box protein A1;Hepatocyte nuclear factor 3-alpha;Transcription factor 3A;cDNA FLJ50362, highly s	FOXA1;HNF3A	Hepatocyte nuclear fac	P55317;B4E257;B7	2.51E-06	0.10571	0.13708	106.53	3	-2.86691
Ubiquitin-like protein FUBI;40S ribosomal protein S30	FAU	ubiquitin-like protein f	P35544;P62861	0.012101	0.099286	0.13928		1	-2.84394
Protein DEK;cDNA FLJ51914, highly similar to Protein DEK;cDNA FLJ53031, highly similar to Protein DEK;cDN	DEK	Protein DEK;cDNA FLJ5	P35659;B4DNW3;	2.16E-123	0.12934	0.14215	137.84	45	-2.81451
Heterogeneous nuclear ribonucleoproteins A2/B1;Putative uncharacterized protein HNRNPA2B1	HNRNPA2B1;	Isoform B1 of Heteroge	P22626-1;P22626;	5.13E-193	0.10804	0.14315	89.552	72	-2.8044
Importin subunit alpha-2;Karyopherin subunit alpha-2;RAG cohort protein 1;SRP1-alpha;cDNA FLJ78270, hig	KPNA2;RCH1;	Importin subunit alpha	P52292;A8K7D9;Q	0.010612	0.11086	0.14373		1	-2.79857
86 kDa subunit of Ku antigen;ATP-dependent DNA helicase 2 subunit 2;ATP-dependent DNA helicase II 80 kD	G22P2;XRCC5	X-ray repair cross-com	P13010;Q53T09;Q	1.58E-63	0.099512	0.14462	118.9	30	-2.78966
Replication factor A protein 1;Replication protein A 70 kDa DNA-binding subunit;Single-stranded DNA-bindi	REPA1;RPA1;R	Replication protein A 7	P27694	4.77E-125	0.10462	0.14601	124.26	33	-2.77586
Heterogeneous nuclear ribonucleoprotein A0	HNRNPA0;HN	Heterogeneous nuclea	Q13151	7.03E-109	0.10881	0.14629	152.89	27	-2.7731
cDNA FLJ56389, highly similar to Elongation factor 1-gamma;eEF-1B gamma;Elongation factor 1-gamma;Euk	EEF1G;EF1G;P	cDNA FLJ56389, highly	B4DTG2;P26641;Q	7.47E-24	0.11408	0.14683	141.27	3	-2.76778
Brain trypsinogen;Mesotrypsinogen;Serine protease 3;Serine protease 4;Trypsin III;Trypsin IV;Trypsin-3;Prot	PRSS3;PRSS4;	Isoform A of Trypsin-3;	P35030-1;P35030;	0.002293	0.10796	0.14859		1	-2.75059
Lamina-associated polypeptide 2, isoform alpha;Splenin;Thymopentin;Thymopoietin;Thymopoietin isoform	LAP2;TMPO	Lamina-associated poly	P42166	5.73E-49	0.11296	0.1491	10.908	2	-2.74565
Glutathione S-transferase kappa 1;Glutathione S-transferase subunit 13;GST 13-13;GST class-kappa;GSTK1-1	GSTK1;HDCM	Isoform 2 of Glutathio	Q9Y2Q3-2;Q9Y2Q3	2.28E-19	0.10584	0.15258	21.528	3	-2.71236
40S ribosomal protein S2;40S ribosomal protein S4;Protein LLRep3;Similar to 40S ribosomal protein S2;Ribo	RPS2;RPS4;LO	40S ribosomal protein	P15880;A4D0Y7;O	0.00017114	0.10956	0.15468	30.855	2	-2.69264
Nuclear factor 1;CCAAT-box-binding transcription factor;Nuclear factor 1 C-type;Nuclear factor I/C;TGGCA-b	NFIC;NFI	Uncharacterized protei	B5MDB4;B7Z4U5;P	6.83E-07	0.1059	0.16149	127.55	3	-2.63048
Putative RNA-binding protein 3;RNA-binding motif protein 3;RNPL	RBM3;RNPL	Putative RNA-binding	P98179	2.18E-15	0.13145	0.16281	29.412	3	-2.61874
Helix-destabilizing protein;Heterogeneous nuclear ribonucleoprotein A1;hnRNP core protein A1;Single-stran	HNRNPA1;HN	Isoform A1-B of Heter	P09651-1;P09651;	2.01E-206	0.12628	0.16694	89.536	63	-2.5826
Histone H1.5;Histone H1a	H1F5;HIST1H1	Histone H1.5	P16401	1.26E-09	0.11325	0.16733	128.84	4	-2.57923
NTEF-1;Protein GT-IIC;TEA domain family member 1;Transcription factor 13;Transcriptional enhancer factor	TCF13;TEAD1;	Transcriptional enhanc	P28347;Q59EF3	5.66E-05	0.12275	0.16862		1	-2.56815
Nucleolin;Protein C23;cDNA FLJ10452 fis, clone NT2RP1000966, highly similar to NUCLEOLIN;Nucleolin, isof	NCL;hCG_339	Nucleolin;cDNA FLJ457	P19338;B3KM80;B	2.47E-50	0.12268	0.17227	137.73	20	-2.53726
cDNA FLJ77718;Cellular nucleic acid binding protein alpha variant 1;Zinc finger protein 9 (A cellular retrovira	CNBP;RNF163	cellular nucleic acid-bi	A8K7V4;Q5U0E9;P	8.10E-10	0.14176	0.17444	75.157	3	-2.5192
ATP-dependent helicase SMARCA2;BRG1-associated factor 190B;Probable global transcription activator SNF	BAF190B;BRM	Isoform Long of Proba	P51531-1;P51531;	9.75E-11	0.18271	0.17456		1	-2.51821
50 kDa nuclear polyadenylated RNA-binding protein;Bruno-like protein 2;CUG triplet repeat RNA-binding pr	BRUNOL2;CEL	Isoform 4 of CUGBP El	Q92879-4;Q92879;	3.19E-38	0.123	0.17905	102.05	21	-2.48157
High mobility group nucleosome-binding domain-containing protein 3;Thyroid receptor-interacting protein	HMGN3;PNAS	Isoform 1 of High mobi	Q15651-1;Q15651;	2.95E-28	0.12038	0.18049	181.68	2	-2.47001
Heterogeneous nuclear ribonucleoprotein L;cDNA, FLJ94229, highly similar to Homo sapiens heterogeneous	HNRNPL;HNR	Heterogeneous nuclea	P14866;B2R959;Q	4.81E-113	0.14368	0.18559	144.68	42	-2.42981

Dimethyladenosine transferase 2, mitochondrial;Hepatitis C virus NS5A-transactivated protein 5;Mitochond	NS5ATP5;TFB2	Dimethyladenosine tra	Q9H5Q4;B4DTJ4	0.00020666	0.13654	0.18564		1	-2.42942
Chromatin assembly factor 1 subunit A;Chromatin assembly factor I p150 subunit	CAF;CAF1P15(	Isoform 1 of Chromatir	Q13111-1;Q13111;	2.45E-10	0.12428	0.18722		1	-2.41719
APOBEC1-binding protein 1;Heterogeneous nuclear ribonucleoprotein A/B;cDNA FLJ60713, highly similar to	ABBP1;HNRNI	Isoform 3 of Heteroger	Q99729-3;Q99729;	9.47E-60	0.14826	0.18958	259.7	17	-2.39912
CAAT box DNA-binding protein subunit C;Nuclear transcription factor Y subunit C;Nuclear transcription facto	NFYC;DKFZp6(	Isoform 3 of Nuclear tr	Q13952-1;Q13952;	2.16E-11	0.13223	0.18981		1	-2.39737
Ribosomal protein S27;40S ribosomal protein S27;Metallopan-stimulin 1;40S ribosomal protein S27-like;Put	LOC392748;tc	Similar to ribosomal pr	A4D1G5;C9JLI6;P42	0.0036727	0.1373	0.19091		1	-2.38904
DEAD box protein 21;Gu-alpha;Nucleolar RNA helicase 2;Nucleolar RNA helicase Gu;Nucleolar RNA helicase	DDX21;OK/SW	Isoform 1 of Nucleolar	Q9NR30-1;Q9NR30	0.0025836	0.14483	0.19613	87.683	4	-2.35012
A18 hnRNP;Cold-inducible RNA-binding protein;Glycine-rich RNA-binding protein CIRP;Cold inducible RNA b	A18HNRNP;CI	Cold-inducible RNA-bir	Q14011;Q53XX5;B3	5.97E-27	0.13937	0.19773		1	-2.3384
PAI1 RNA-binding protein 1;Plasminogen activator inhibitor 1 RNA-binding protein;SERPINE1 mRNA-binding	CGI-55;PAIRBI	SERPINE1 mRNA bindir	Q8NC51-1;Q8NC51	1.28E-63	0.14067	0.19968	138.24	7	-2.32424
Bardet-Biedl syndrome 14 protein;Cancer/testis antigen 87;Centrosomal protein of 290 kDa;Nephrocystin-6	BBS14;CEP29(	Isoform 1 of Centroson	O15078-1;O15078;	0.13248	0.15069	0.20244		1	-2.30443
Microtubule-associated protein 4;cDNA FLJ58263, highly similar to Microtubule-associated protein 4;MAP4	MAP4	245 kDa protein;Isofor	P27816-1;P27816;I	7.17E-58	0.1332	0.20435	130.93	17	-2.29089
ATP-dependent DNA helicase Q1;DNA helicase, RecQ-like type 1;DNA-dependent ATPase Q1;RecQ protein-li	RECQ1;RECQL	ATP-dependent DNA he	P46063	2.58E-80	0.14322	0.20587	106.32	26	-2.28019
ATP-dependent RNA helicase A;DEAH box protein 9;Nuclear DNA helicase II;cDNA FLJ39263 fis, clone OCBBF	DDX9;DHX9;LI	ATP-dependent RNA he	Q08211;B3KU66;Q	1.08E-11	0.15393	0.20758	238.45	8	-2.26826
TAR DNA-binding protein 43;cDNA FLJ57180, highly similar to TAR DNA-binding protein 43;TAR DNA binding	TARDBP;TDP4	Isoform 2 of TAR DNA-I	Q13148-2;Q13148;	3.85E-78	0.1746	0.2091	102.43	11	-2.25774
DNA topoisomerase 1;DNA topoisomerase I;Topoisomerase (DNA) I;TOP1 protein;Putative uncharacterized	TOP1	DNA topoisomerase 1	P11387;B9EG90;Q6	5.71E-58	0.17106	0.21015	126.83	28	-2.25051
75 kDa DNA-pairing protein;Oncogene FUS;Oncogene TLS;POMp75;RNA-binding protein FUS;Translocated ii	FUS;TLS	Fus-like protein (Fragm	P35637-1;P35637;(	2.66E-33	0.189	0.21386	114.74	24	-2.22526
Zinc fingers and homeoboxes protein 1;cDNA FLJ78425, highly similar to Homo sapiens zinc fingers and hom	ZHX1	Zinc fingers and homec	Q9UKY1;A8K0G7	6.29E-17	0.14876	0.22018		1	-2.18324
High mobility group protein 1;High mobility group protein B1;High-mobility group box 1;Putative high mobil	HMG1;HMGB	High mobility group pr	P09429;Q5T7C6;Q	3.79E-17	0.14739	0.22414	114.91	6	-2.15753
RNA-binding motif protein 47;RNA-binding protein 47;RNA binding motif protein 47;cDNA FLJ51456, moder	RBM47	Isoform 1 of RNA-bindi	A0AV96-1;A0AV96;	0.00029068	0.17408	0.22696	40.42	2	-2.13949
Activating transcription factor 1;Cyclic AMP-dependent transcription factor ATF-1;Protein TREB36;cDNA, FLJ	ATF1	Cyclic AMP-dependent	P18846;B2R8K4;B4	6.87E-13	0.16627	0.23314	146.76	8	-2.10073
Serine/threonine-protein kinase VRK1;Vaccinia-related kinase 1;cDNA FLJ75441, highly similar to Homo sapi	VRK1	Serine/threonine-prote	Q99986;A8K245	3.42E-05	0.1878	0.2339	50.238	2	-2.09604
PWP1-interacting protein 17;Single-stranded DNA-binding protein, mitochondrial;cDNA, FLJ93504, Homo sa	SSBP;SSBP1;h	Single-stranded DNA-b	Q04837;A4D1U3;Q	1.03E-38	0.16666	0.23459	102.77	11	-2.09179
Far upstream element-binding protein 3;cDNA FLJ58115, highly similar to Far upstream element-binding pro	FBP3;FUBP3;h	Isoform 1 of Far upstre	Q96I24-1;Q96I24;A	8.31E-181	0.19578	0.24453	70.489	69	-2.03192
Heterogeneous nuclear ribonucleoprotein A3;cDNA FLJ52659, highly similar to Heterogeneous nuclear ribo	HNRNPA3;HNI	Isoform 1 of Heteroger	P51991-1;P51991;I	2.28E-60	0.17397	0.24969	88.835	18	-2.00179
DNA helicase V;Far upstream element-binding protein 1;cDNA FLJ59240, highly similar to Far upstream elem	FUBP1	Isoform 1 of Far upstre	Q96AE4-1;Q96AE4	5.38E-133	0.19398	0.25681	91.772	39	-1.96123
Glycine- and tyrosine-rich RNA-binding protein;Heterogeneous nuclear ribonucleoprotein Q;NS1-associated	HNRPQ;NSAP	Isoform 3 of Heteroger	O60506-3;O60506;	1.42E-127	0.21155	0.25756	124.18	30	-1.95702
Far upstream element-binding protein 2;KH type-splicing regulatory protein;p75;cDNA FLJ51330, highly simi	FUBP2;KHSRP	Isoform 1 of Far upstre	Q92945-1;Q92945;	2.17E-157	0.2016	0.25896	59.322	34	-1.9492
Splicing factor 9G8;Splicing factor, arginine/serine-rich 7;Putative uncharacterized protein SFRS7;cDNA FLJ5	SFRS7	Isoform 1 of Serine/arg	Q16629-1;Q16629;	5.88E-06	0.18703	0.26497	133.6	4	-1.9161
CTD-associated SR protein 11;Renal carcinoma antigen NY-REN-40;SC35-interacting protein 1;SFRS2-interact	CASP11;SFRS2	Isoform 1 of SRSF2-inte	Q99590-1;Q99590;	2.69E-05	0.2249	0.26548	143.66	2	-1.91332
UPF0361 protein C3orf37;cDNA FLJ57398	C3orf37;DC12	UPF0361 protein C3orf	Q96FZ2;B4DMK2;D	1.32E-79	0.18986	0.2688	111.22	16	-1.89539
Polypyrimidine tract binding protein 1;Polypyrimidine tract binding protein 1, isoform CRA_c;57 kDa RNA-bi	hCG_20560;P	polypyrimidine tract-bi	Q9BUQ0;P26599-2	6.90E-210	0.17972	0.27122	126.54	69	-1.88246
Heterogeneous nuclear ribonucleoprotein U;p120;pp120;Scaffold attachment factor A;cDNA FLJ44920 fis, cl	HNRNPU;HNR	Isoform Long of Heterc	Q00839-1;Q00839;	3.26E-137	0.19912	0.2717	142.8	34	-1.87991
DEAH box protein 36;MLE-like protein 1;Probable ATP-dependent RNA helicase DHX36;RNA helicase associa	DDX36;DHX36	Isoform 1 of Probable	Q9H2U1-1;Q9H2U	5.86E-118	0.19905	0.27283	61.319	14	-1.87393
Heterogeneous nuclear ribonucleoprotein K;Transformation up-regulated nuclear protein;cDNA FLJ53312, h	HNRNPK;HNR	Isoform 2 of Heteroger	P61978-2;P61978;I	4.29E-233	0.21745	0.27505	135.77	65	-1.86223
Cold shock domain-containing protein A;DNA-binding protein A;Single-strand DNA-binding protein NF-GMB	CSDA;DBPA	Isoform 1 of DNA-bindi	P16989-1;P16989;I	4.16E-20	0.21785	0.27786	48.342	5	-1.84757
Kinesin-like protein 2;Kinesin-like protein KIFC1;Kinesin-related protein HSET;cDNA FLJ53841, highly similar	HSET;KIFC1;KI	Kinesin-like protein KIF	Q9BW19;B4DMA8;	3.41E-21	0.20576	0.28124	205.21	2	-1.83013
Death-associated transcription factor 1;Death-inducer obliterator 1	C20orf158;DA	Isoform 4 of Death-ind	Q9BTC0-4;Q9BTC0;	5.41E-08	0.19746	0.28484	69.642	3	-1.81178
CCT-gamma;hTRiC5;T-complex protein 1 subunit gamma;cDNA FLJ57603, highly similar to T-complex proteir	CCT3;CCTG;TR	T-complex protein 1 su	P49368;B4DUR8;Q	1.52E-10	0.21061	0.28655	177.64	2	-1.80314
NAD(+) ADP-ribosyltransferase 1;Poly [ADP-ribose] polymerase 1;Poly[ADP-ribose] synthase 1;cDNA, FLJ926	ADPRT;PARP1	Poly [ADP-ribose] poly	P09874;B2R5W3;B	0	0.23292	0.29369	150.54	176	-1.76763
cDNA FLJ56904, highly similar to RNA-binding protein Musashi homolog 2;RNA-binding protein Musashi hor	MSI2	cDNA FLJ56904, highly	B4DHE8;Q96DH6-1	2.06E-08	0.22156	0.29521	182.46	2	-1.76019
Lark homolog;RNA-binding motif protein 4;RNA-binding motif protein 4a;RNA-binding protein 4;cDNA FLJ7	RBM4;RBM4A	Isoform 1 of RNA-bindi	Q9BWF3-1;Q9BWF	8.25E-128	0.22967	0.3017	105.21	42	-1.72881
RNA-binding motif, single-stranded-interacting protein 1;Single-stranded DNA-binding protein MSSP-1;Supp	C2orf12;MSSF	Isoform 1 of RNA-bindi	P29558-1;P29558;(	8.58E-13	0.20884	0.30363	174.97	2	-1.71961
Pre-mRNA-splicing factor SRP20;Splicing factor, arginine/serine-rich 3;cDNA, FLJ92926, Homo sapiens splicir	SFRS3;SRP20;I	Serine/arginine-rich sp	P84103;B2R6F3;B4	5.42E-05	0.22952	0.30679	70.222	2	-1.70468
Heterogeneous nuclear ribonucleoprotein R;cDNA, FLJ93632, highly similar to Homo sapiens heterogeneou	HNRNPR;HNR	71 kDa protein;Isoform	O43390-2;O43390;	1.32E-24	0.22256	0.30712	112.33	9	-1.70313
ATP-dependent RNA helicase DDX48;ATP-dependent RNA helicase eIF4A-3;DEAD box protein 48;Eukaryotic i	DDX48;EIF4A3	Eukaryotic initiation fa	P38919	0.0090536	0.2198	0.30755		1	-1.70111
130 kDa leucine-rich protein;GP130;Leucine-rich PPR motif-containing protein, mitochondrial;cDNA FLJ600	LRP130;LRPPF	Leucine-rich PPR motif	P42704;B4DSR0;E5	4.20E-48	0.24797	0.30974	96.833	15	-1.69087
60S ribosomal protein L3;HIV-1 TAR RNA-binding protein B;Ribosomal protein L3;cDNA FLJ35376 fis, clone S	OK/SW-cl.32;I	60S ribosomal protein	P39023;Q96QL0;B3	0.00014215	0.22227	0.31654	102.61	3	-1.65954
60S ribosomal protein L10;Laminin receptor homolog;Protein QM;Tumor suppressor QM;Ribosomal protein	DXS648E;QM;	60S ribosomal protein	P27635;Q5HY50;Q	0.014478	0.24474	0.3169	165.64	2	-1.6579
Hepatoma-derived growth factor;High mobility group protein 1-like 2;cDNA, FLJ96580, highly similar to Hon	HDGF;HMG1L	Hepatoma-derived gro	P51858;B2RDE8;B7	1.26E-38	0.22528	0.31868	83.161	5	-1.64982
cDNA FLJ75056, highly similar to Homo sapiens phosphatidylinositol binding clathrin assembly protein (PIC	PICALM;PICAL	Uncharacterized protei	A8K5U9;A8MW24;	1.99E-22	0.24046	0.31868	93.095	9	-1.64982
cDNA FLJ60076, highly similar to ELAV-like protein 1;ELAV-like protein 1;Hu-antigen R	ELAVL1;HUR	cDNA FLJ60076, highly	B4DVB8;Q15717	6.07E-53	0.2493	0.32873	98.07	10	-1.60502
Dr1-associated corepressor;Dr1-associated protein 1;Negative co-factor 2-alpha;Putative uncharacterized pi	DRAP1	Uncharacterized protei	Q14919-2;Q14919;	7.08E-06	0.23868	0.32982	99.603	4	-1.60025
Poly(RC)-binding protein 2 isoform b variant;Poly(RC) binding protein 2;Poly(RC) binding protein 2, isoform (	hCG_2017557	poly(rC)-binding protei	Q59HD4;Q6IPF4;Q	5.33E-123	0.23347	0.33282	126.39	16	-1.58719
Heterogeneous nuclear ribonucleoprotein 2H9;Heterogeneous nuclear ribonucleoprotein H3;Heterogeneou	HNRNPH3;HN	Isoform 1 of Heteroger	P31942-1;P31942;(	1.12E-18	0.25647	0.33337	51.078	4	-1.5848
UPF0534 protein C4orf43	C4orf43	Protein;UPF0534 prote	Q96EY4;D6RA57	7.42E-51	0.25504	0.33745	131.71	6	-1.56725
High mobility group protein 2a;High mobility group protein 4;High mobility group protein B3;Non-histone cl	HMG2A;HMG	High mobility group pr	O15347;Q16466	1.02E-27	0.22013	0.33875	103.82	8	-1.56171
100 kDa DNA-pairing protein;DNA-binding p52/p100 complex, 100 kDa subunit;Polypyrimidine tract-binding	PSF;SFPQ	Isoform Long of Splicin	P23246-1;P23246;(	4.96E-69	0.2388	0.33924	97.721	23	-1.55962
Cullin-1;cDNA FLJ38844 fis, clone MESAN2003662, highly similar to Cullin-1;cDNA FLJ39186 fis, clone OCBBI	CUL1	Cullin-1;cDNA FLJ5850	Q13616;B3KTW0;B	0.10854	0.2542	0.33962		1	-1.55801
cDNA FLJ75459;cDNA FLJ76290, highly similar to Homo sapiens TIA1 cytotoxic granule-associated RNA bind	TIAL1;hCG_40	nucleolysin TIAR isofor	A8K4L9;A8K5C4;Q(	1.40E-51	0.25481	0.35613	9.8767	3	-1.48952
Alpha-CP1;Heterogeneous nuclear ribonucleoprotein E1;Nucleic acid-binding protein SUB2.3;Poly(rC)-bindir	PCBP1;hCG_1	Poly(rC)-binding protei	Q15365;Q53SS8	2.46E-111	0.2759	0.36033	111.45	25	-1.47261
Beta-II spectrin;Fodrin beta chain;Spectrin beta chain, brain 1;Spectrin, non-erythroid beta chain 1;Spectrin	SPTB2;SPTBN	Isoform Long of Spectr	Q01082-1;Q01082;	0.0024362	0.25301	0.36142		1	-1.46825
AU-rich element RNA-binding factor;Heterogeneous nuclear ribonucleoprotein D-like;JKT41-binding protein	HNRPDL;JKTBI	Isoform 1 of Heteroger	O14979-1;O14979;	1.09E-10	0.29097	0.36322	135.69	3	-1.46108

Nucleolysin TIA-1 isoform p15;Nucleolysin TIA-1 isoform p40;p15-TIA-1;p40-TIA-1;RNA-binding protein TIA-1	TIA1;hCG_405	Isoform Long of Nuclec	P31483-1;P31483;(	3.34E-45	0.28083	0.37092	72.947	15	-1.43082
cDNA FLJ53554	UBC;DKFZp43	cDNA FLJ53554	B4DGK4	0.0036811	0.30004	0.37271	113.13	4	-1.42387
cDNA FLJ75516, highly similar to Xenopus tropicalis ubiquitin C, mRNA;Ubiquitin C variant;Ubiquitin C splice	UBC;DKFZp43	Polyubiquitin-C;39 kDa	P0CG48;A8K674;Q!	1.14E-25	0.26443	0.37426	198.31	11	-1.41789
Zinc finger and SCAN domain-containing protein 2;Zinc finger protein 29 homolog;cDNA FLJ77532, highly sir	ZFP29;ZSCAN2	Isoform 1 of Zinc finger	Q7Z7L9-1;Q7Z7L9;/	0.32681	0.30043	0.37549		1	-1.41315
70 kDa subunit of Ku antigen;ATP-dependent DNA helicase 2 subunit 1;ATP-dependent DNA helicase II 70 kD	G22P1;XRCC6	X-ray repair cross-comp	P12956;B2RDN9;B1	1.84E-94	0.33118	0.37882	123.56	28	-1.40042
Ribosomal protein S10 variant;Ribosomal protein S10;40S ribosomal protein S10	RP11-375E1__	Ribosomal protein S10	Q59GE4;Q5TZB9;P4	6.07E-05	0.25964	0.37971		1	-1.39703
Putative uncharacterized protein DKFZp779B0247;Cold shock domain-containing protein E1;N-ras upstream	DKFZp779B02	94 kDa protein;cold sh	Q68DF1;O75534-1;	1.75E-24	0.31478	0.38135	135.98	11	-1.39081
Heterogeneous nuclear ribonucleoprotein M;Heterogeneous nuclear ribonucleoprotein M isoform a variant	HNRNPM;HNF	Isoform 1 of Heteroger	P52272-1;P52272;(	2.14E-75	0.31263	0.38213	85.947	24	-1.38786
Dolichyl-diphosphooligosaccharide--protein glycosyltransferase 67 kDa subunit;Dolichyl-diphosphooligosacc	RPN1	Dolichyl-diphosphoolig	P04843;Q53EP4;Q(	0.010782	0.28979	0.39116		1	-1.35417
ATP-dependent RNA helicase eIF4A-2;Eukaryotic initiation factor 4A-II;Eukaryotic translation initiation factor	DDX2B;EIF4A2	Isoform 2 of Eukaryotic	Q14240-2;Q14240;	3.59E-06	0.29707	0.39207	155.24	2	-1.35082
Protein PR264;Splicing component, 35 kDa;Splicing factor SC35;Splicing factor, arginine/serine-rich 2;cDNA I	SFRS2;hCG_27	Serine/arginine-rich sp	Q01130;B3KUY1;Q	5.01E-25	0.31358	0.39317	29.142	6	-1.34677
Basic transcription element-binding protein 4;Krueppel-like factor 16;Novel Sp1-like zinc finger transcription	BTEB4;KLF16;I	Krueppel-like factor 16	Q9BXK1;D6W5Z8	1.53E-31	0.31828	0.39555		1	-1.33807
60 kDa poly(U)-binding-splicing factor;FUSE-binding protein-interacting repressor;Poly(U)-binding-splicing fa	FIR;PUF60;RO	Isoform 1 of Poly(U)-bi	Q9UHX1-1;Q9UHX1	3.51E-21	0.29121	0.39939	65.145	6	-1.32413
Heterogeneous nuclear ribonucleoprotein L-like;Stromal RNA-regulating factor;cDNA FLJ77927;Heterogene	BLOCK24;HNR	Isoform 1 of Heteroger	Q8WVV9-1;Q8WVV	0.00020195	0.31742	0.40056	55.911	2	-1.31991
p195;Ras GTPase-activating-like protein IQGAP1;IQ motif containing GTPase activating protein 1;cDNA FLJ5!	IQGAP1;KIAA(	Ras GTPase-activating-	P46940;A4QPB0;B4	2.41E-16	0.27648	0.40131	104.86	3	-1.31721
Adenovirus early region 1B-associated protein 5;E1B-55 kDa-associated protein 5;Heterogeneous nuclear ril	E1BAP5;HNRN	Isoform 1 of Heteroger	Q9BUJ2-1;Q9BUJ2;	1.70E-19	0.27648	0.40131	120.17	3	-1.31721
20 kDa CGG-binding protein;CGG triplet repeat-binding protein 1;p20-CGGBP DNA-binding protein;Putative	CGGBP;CGGBI	CGG triplet repeat-binc	Q9UFW8;C9JUJ0	1.33E-06	0.2955	0.40248	173.7	2	-1.31301
Cellular apoptosis susceptibility protein;Chromosome segregation 1-like protein;Exportin-2;Importin-alpha r	CAS;CSE1L;XP(	Isoform 1 of Exportin-2	P55060-1;P55060;E	0.014654	0.27639	0.4147	278.71	2	-1.26986
Hepatocellular carcinoma protein 1;RNA-binding motif protein 39;RNA-binding protein 39;RNA-binding regi	HCC1;RBM39;	Isoform 1 of RNA-bindi	Q14498-1;Q14498;	3.73E-59	0.33697	0.41564	68.689	9	-1.26659
DAZ-associated protein 1;Deleted in azoospermia-associated protein 1;cDNA FLJ35575 fis, clone SPLEN2006	DAZAP1	Isoform 1 of DAZ-assoc	Q96EP5-1;Q96EP5;	6.31E-94	0.31466	0.42046	84.474	16	-1.24996
IGF-II mRNA-binding protein 3;Insulin-like growth factor 2 mRNA-binding protein 3;KH domain-containing pr	IGF2BP3;IMP3	Isoform 1 of Insulin-like	O00425-1;O00425	6.47E-11	0.33465	0.42077	87.704	7	-1.2489
CCAAT-binding transcription factor I subunit A;DNA-binding protein B;Enhancer factor I subunit A;Nuclease-s	NSEP1;YB1;YB	Protein;Nuclease-sensi	P67809;A0JLU4;Q0	2.65E-91	0.25598	0.43396	149.46	19	-1.20437
Neural polypyrimidine tract-binding protein;Neurally-enriched homolog of PTB;Polypyrimidine tract-binding	NPTB;PTB;PTE	Isoform 2 of Polypyrim	Q9UKA9-2;Q9UKAS	7.81E-17	0.32231	0.43725	11.35	2	-1.19347
cDNA FLJ31747 fis, clone NT2RI2007377, highly similar to RNA-BINDING PROTEIN EWS;Ewing sarcoma breal	EWSR1;hCG_2	RNA-binding protein EV	Q96MX4;Q01844-1	1.28E-24	0.34603	0.4402	60.348	3	-1.18377
Thymocyte nuclear protein 1;Thymocyte protein Thy28	HSPC144;MDS	Isoform 1 of Thymocyt	Q9P016-1;Q9P016	6.47E-12	0.32384	0.44086	133.08	5	-1.18161
Activator 1 140 kDa subunit;Activator 1 large subunit;Activator 1 subunit 1;DNA-binding protein PO-GA;Rep	RFC1;RFC140;	Isoform 1 of Replicatio	P35251-1;P35251;I	0.003283	0.3151	0.44467		1	-1.16919
Methyl-CpG-binding protein 2;cDNA FLJ75240, highly similar to Homo sapiens methyl CpG binding protein 2	MECP2;hCG_3	Isoform B of Methyl-Cp	P51608-2;P51608;/	1.47E-38	0.3528	0.46218	191.03	2	-1.11347
Heterogeneous nuclear ribonucleoprotein H;Heterogeneous nuclear ribonucleoprotein H, N-terminally proc	HNRNPH1;HN	51 kDa protein;Heterog	P31943;B4DP51;Q(	1.95E-79	0.37484	0.46626	72.011	15	-1.10079
ATP-dependent RNA helicase DDX3X;DEAD box protein 3, X-chromosomal;DEAD box, X isoform;Helicase-like	DBX;DDX3;DD	ATP-dependent RNA he	O00571;B4E3E8;B5	1.60E-31	0.38143	0.47004	133.36	10	-1.08914
40S ribosomal protein S3;Ribosomal protein S3 variant;Ribosomal protein S3	OK/SW-cl.26;F	28 kDa protein;40S ribo	P23396;Q53G83;Q!	8.67E-07	0.33187	0.47354	72.584	5	-1.07844
Bruton tyrosine kinase-associated protein 135;General transcription factor II-I;SRF-Phox1-interacting proteir	BAP135;GTF2I	Isoform 1 of General tr	P78347-1;P78347;E	1.73E-17	0.32284	0.47694	98.937	7	-1.06812
Histone H4	H4/A;H4/B;H4	Histone H4	P62805;B2R4R0;Q(	9.67E-16	0.38387	0.47877	129.47	10	-1.0626
60S ribosomal protein L30	RPL30	60S ribosomal protein	P62888;E5RI99;E5F	3.85E-23	0.37144	0.48455	116.23	4	-1.04528
Gamma-interferon-inducible protein 16;Interferon-inducible myeloid differentiation transcriptional activato	IFI16;IFNGIP1;	Interferon, gamma-ind	Q16666-2;Q16666;	6.66E-103	0.33879	0.48556	124.89	25	-1.04228
Glycylpeptide N-tetradecanoyltransferase 1;Myristoyl-CoA:protein N-myristoyltransferase 1;Peptide N-myris	NMT;NMT1	Isoform Long of Glycylp	P30419-1;P30419;(	0.00012716	0.349	0.48848	6.4037	2	-1.03363
Replication factor A protein 2;Replication protein A 32 kDa subunit;Replication protein A 34 kDa subunit;cDI	REPA2;RPA2;R	Isoform 3 of Replicatio	P15927-3;P15927;I	6.85E-23	0.40183	0.50021	155.12	6	-0.99939
DEAD box polypeptide 17 isoform p82 variant;DEAD box protein 17;DEAD box protein p72;Probable ATP-dep	DDX17;RP3-43	probable ATP-depende	Q59F66;Q92841-4;	5.66E-32	0.41146	0.50126	59.592	7	-0.99637
Glycoprotein p43;Heterogeneous nuclear ribonucleoprotein G;Heterogeneous nuclear ribonucleoprotein G,	HNRPG;RBMX	Heterogeneous nuclea	P38159;B4E352;Q2	1.52E-13	0.37601	0.51458	122.16	3	-0.95853
Nonsense mRNA reducing factor 3B;Regulator of nonsense transcripts 3B;Up-frameshift suppressor 3 homo	RENT3B;UPF3	Isoform 1 of Regulator	Q9BZI7-1;Q9BZI7;C	0.00058423	0.40252	0.51777	117.67	2	-0.94962
Nucleolar and spindle-associated protein 1;cDNA FLJ78441, highly similar to Homo sapiens nucleolar and sp	ANKT;BM-037	Isoform 1 of Nucleolar	Q9BXS6-1;Q9BXS6;	6.22E-23	0.44778	0.54006	117.63	9	-0.88881
Pre-rRNA-processing protein TSR1 homolog	KIAA1401;TSR	Pre-rRNA-processing p	Q2NL82	5.70E-11	0.37939	0.54125	90.668	3	-0.88563
Target of EGR1 protein 1;cDNA FLJ36001 fis, clone TESTI2015213, highly similar to Homo sapiens target of E	TOE1	Target of EGR1 protein	Q96GM8;B3KSC7;B	0.0010216	0.40255	0.54137		1	-0.88531
eNOS-interacting protein;Nitric oxide synthase-interacting protein;cDNA FLJ75703, highly similar to Homo s	CGI-25;NOSIP	Nitric oxide synthase-ir	Q9Y314;A8K670;B4	0.0060592	0.43633	0.54873		1	-0.86583
DNA mismatch repair protein Msh6;G/T mismatch-binding protein;MutS-alpha 160 kDa subunit;cDNA FLJ55	GTBP;MSH6	Isoform GTBP-N of DN/	P52701-1;P52701;I	1.54E-81	0.38163	0.54939	113.91	24	-0.8641
60S ribosomal protein L11;CLL-associated antigen KW-12;Cell growth-inhibiting protein 34;cDNA, FLJ92077,	RPL11;hCG_1	Isoform 1 of 60S ribosc	P62913-1;P62913;(	1.73E-33	0.41962	0.55078	131.73	10	-0.86045
68 kDa TATA-binding protein-associated factor;RNA-binding protein 56;TATA-binding protein-associated fact	RBP56;TAF15;	Isoform Long of TATA-b	Q92804-1;Q92804;	8.65E-37	0.47177	0.58201	16.195	4	-0.78088
DNA ligase 3;DNA ligase III;Polydeoxyribonucleotide synthase [ATP] 3;DNA ligase;Ligase III, DNA, ATP-depen	LIG3	Isoform Alpha of DNA I	P49916-1;P49916;I	1.06E-57	0.47197	0.5973	90.387	15	-0.74347
Mitochondrial transcription factor 1;Transcription factor 6;Transcription factor 6-like 2;Transcription factor A	TCF6;TCF6L2;1	Transcription factor A,	Q00059;E5KSU5;E5	5.98E-57	0.48656	0.59961	132.67	20	-0.7379
DNA topoisomerase 2;DNA topoisomerase 2-alpha;DNA topoisomerase II, alpha isozyme	TOP2A;TOP2	Uncharacterized protei	C9J4C3;P11388-4;F	6.39E-14	0.44586	0.60089	90.401	4	-0.73483
40S ribosomal protein S5;40S ribosomal protein S5, N-terminally processed;Ribosomal protein S5 variant	RPS5	40S ribosomal protein	P46782;Q53G25	1.69E-07	0.46927	0.60462	97.642	6	-0.7259
Inactive ubiquitin-specific peptidase 39;SAD1 homolog;U4/U6.U5 tri-snRNP-associated 65 kDa protein;U4/U	CGI-21;HSPC3	U4/U6.U5 tri-snRNP-as	Q53GS9;B7Z7L9;B9	2.71E-26	0.45748	0.60779	94.209	7	-0.71836
Bromodomain-containing protein 4;Protein HUNK1;Similar to bromodomain-containing 4;BRD4 protein;Bro	BRD4;HUNK1;	Isoform 1 of Bromodor	O60885-1;O60885;	0.0081511	0.41333	0.61217		1	-0.708
PHD finger protein 6;PHD finger protein 6, isoform CRA_d;PHD-like zinc finger protein;cDNA FLJ60207, highl	AC004383.6-0	PHD finger protein 6, is	Q5JRC6;Q8IWS0-1;	2.93E-10	0.48427	0.62059	105.87	8	-0.68829
cDNA FLJ56786, moderately similar to 40S ribosomal protein S16;40S ribosomal protein S16;RPS16 protein	RPS16	cDNA FLJ56786, moder	B4DP32;P62249;Q(	8.92E-07	0.47022	0.62463	98.554	5	-0.67893
Mortality factor 4 like 1;cDNA FLJ38504 fis, clone HCHON2000156, highly similar to Mortality factor 4-like pr	MORF4L1;FW	cDNA FLJ38504 fis, clor	A5D8W6;B3KTM8;(	0.00073968	0.46242	0.63161	35.846	2	-0.66289
MORF-related gene X protein;Mortality factor 4-like protein 2;Protein MSL3-2;Transcription factor-like prote	KIAA0026;MO	Mortality factor 4-like p	Q15014;B3KWX6;B	0.0089338	0.50423	0.63739		1	-0.64975
NOL1/NOP2/Sun domain family member 2;Substrate of AIM1/Aurora kinase B;tRNA (cytosine-5-)-methyltra	NSUN2;SAKI;T	tRNA (cytosine-5-)-met	Q08J23;A8K529;B3	1.88E-62	0.47584	0.64108	89.08	20	-0.64142
DEAD box protein 5;Probable ATP-dependent RNA helicase DDX5;RNA helicase p68;cDNA FLJ53366, highly s	DDX5;G17P1;I	Probable ATP-depende	P17844;B4DN41;B!	1.06E-11	0.52831	0.6607		1	-0.59793
DNA-directed RNA polymerase, mitochondrial;DNA-directed RNA polymerase;cDNA FLJ58806, highly similar	POLRMT	DNA-directed RNA poly	O00411;Q4G0F4;Q	3.47E-10	0.5319	0.67628	7.5559	3	-0.56431
54 kDa nuclear RNA- and DNA-binding protein;55 kDa nuclear protein;DNA-binding p52/p100 complex, 52 k	NONO;NRB54	Non-POU domain-cont	Q15233;A8K525;B7	5.16E-97	0.55701	0.68159	115.12	44	-0.55302
Chromokinesin-A;Chromosome-associated kinesin KIF4A;cDNA FLJ14204 fis, clone NT2RP3003138, highly si	KIF4;KIF4A	Isoform 1 of Chromoso	O95239-1;O95239;	9.75E-55	0.54367	0.68959	97.985	17	-0.53619

DNase IV;Flap endonuclease 1;Flap structure-specific endonuclease 1;Maturation factor 1;cDNA FLJ51365, h	FEN1;RAD2;hC	Flap endonuclease 1	P39748;B4DWZ4;Q	7.53E-101	0.50395	0.6927	150.98	18	-0.5297
DNA topoisomerase 2-beta;DNA topoisomerase II, beta isozyme;Topoisomerase IIb;DNA topoisomerase 2;cI	TOP2B;topIIb;	Isoform Beta-2 of DNA	Q02880-1;Q02880;	6.85E-11	0.51796	0.70427		1	-0.5058
MutY homolog (E. coli);A/G-specific adenine DNA glycosylase;MutY homolog;MYHbeta;MutY homolog (E. co	MUTYH;RP4-5	a/G-specific adenine D	E5KP25;E5KP30;E5	0.001055	0.55805	0.70667	51.827	2	-0.50089
Nuclear envelope pore membrane protein POM 121;Nuclear envelope pore membrane protein POM 121A;I	KIAA0618;NUI	Isoform 1 of Nuclear er	Q96HA1-1;Q96HA1	0.0062477	0.5638	0.70747		1	-0.49926
Nuclear factor 1;Putative uncharacterized protein NFIA;CCAAT-box-binding transcription factor;Nuclear facto	NFIA;RP5-902	nuclear factor 1 A-type	B4DS53;E2QRI7;B1	2.12E-21	0.54265	0.7153	143.27	8	-0.48338
DNA-binding protein/plasminogen activator inhibitor 1 regulator;Helicase-like transcription factor;HIP116;R	HIP116A;HLTF	Isoform 1 of Helicase-li	Q14527-1;Q14527;	4.94E-289	0.57942	0.73174	140.57	89	-0.4506
Activator 1 40 kDa subunit;Activator 1 subunit 2;Replication factor C 40 kDa subunit;Replication factor C sub	RFC2;hCG_18(	Isoform 1 of Replicatio	P35250-1;P35250;E	0.00036469	0.52913	0.73468		1	-0.44481
ATP-dependent helicase SKIV2L2;Superkiller viralicidic activity 2-like 2;cDNA FLJ76877, highly similar to Hom	KIAA0052;SKIV	Superkiller viralicidic ac	P42285;A8K6I4;B4I	0.011265	0.58588	0.74895		1	-0.41706
Forkhead box protein K1;Myocyte nuclear factor;cDNA FLJ16099 fis, clone TESTI2008278, highly similar to H	FOXK1;MNF;K	Isoform 1 of Forkhead	P85037-1;P85037;F	7.14E-18	0.59275	0.75091	52.038	10	-0.41329
NF-A1;Octamer-binding protein 1;Octamer-binding transcription factor 1;POU domain, class 2, transcription	OCT1;OTF1;PC	POU domain, class 2, tı	P14859-1;P14859;E	5.62E-07	0.59826	0.75288		1	-0.40951
RNA-binding motif protein 42;RNA-binding protein 42;cDNA FLJ60187	RBM42	Isoform 1 of RNA-bindi	Q9BTD8-1;Q9BTD8	2.47E-19	0.5586	0.76968	12.154	2	-0.37767
96 kDa nucleoporin;98 kDa nucleoporin;Nuclear pore complex protein Nup96;Nuclear pore complex proteir	ADAR2;NUP98	Isoform 1 of Nuclear po	P52948-1;P52948;A	4.67E-32	0.59973	0.77259	170.25	4	-0.37223
60S ribosomal protein L7;Similar to 60S ribosomal protein L7; similar to P18124 (PID:d133021);Putative unc	RPL7;WUGSC:	32 kDa protein;30 kDa	P18124;O95036;A8	2.98E-24	0.62479	0.77447	105.9	7	-0.36872
Leucine-rich repeat-containing protein 47	KIAA1185;LRR	Leucine-rich repeat-coı	Q8N1G4	9.93E-30	0.63456	0.77533	17.165	5	-0.36712
CAAT box DNA-binding protein subunit B;Nuclear transcription factor Y subunit B;Nuclear transcription facto	HAP3;NFYB	Nuclear transcription fa	P25208	0.012304	0.52126	0.7787		1	-0.36086
Suppressor of hairy wing homolog 4;Zinc finger protein 280D;cDNA FLJ76213, highly similar to Homo sapien	KIAA1584;SUH	Isoform 1 of Zinc finger	Q6N043-1;Q6N043	6.15E-05	0.58553	0.78153	216.72	2	-0.35563
40S ribosomal protein S19;cDNA, FLJ92047, Homo sapiens ribosomal protein S19 (RPS19), mRNA;Ribosoma	RPS19;hCG_19	40S ribosomal protein	P39019;B0ZBD0;Q8	0.00033165	0.63315	0.78357		1	-0.35187
cDNA FLJ60565, highly similar to Nuclear pore complex protein Nup153;Nucleoporin 153kDa;153 kDa nucle	NUP153;NUP1	cDNA FLJ60565, highly	B4DIK2;Q7Z743;P4	2.30E-118	0.62235	0.78413	99.288	16	-0.35084
Membrane-organizing extension spike protein;Moesin;MSN protein	MSN	Moesin	P26038;Q6PJT4	1.32E-12	0.61129	0.78628	186.35	4	-0.34688
Histone H2B;Histone H2B type 1-N;Histone H2B.d;Histone H2B type 1-C/E/F/G/I;Histone H2B.1 A;Histone H2	H2BFD;HIST1H	cDNA FLJ56787, highly	B4DR52;Q99877;B4	2.03E-32	0.64428	0.78798	148.75	9	-0.34377
Double-stranded RNA-binding protein 76;Interleukin enhancer-binding factor 3;M-phase phosphoprotein 4;	DRBF;ILF3;MP	interleukin enhancer-b	Q12906-4;Q12906;	2.50E-26	0.63184	0.79158	97.566	6	-0.33719
T-box transcription factor TBX3;Putative uncharacterized protein TBX3	TBX3	Isoform II of T-box tran	O15119-1;O15119;	1.21E-85	0.61289	0.79197	62.996	10	-0.33648
Cellular transcription factor ILF-1;Forkhead box protein K2;FOXK2;Interleukin enhancer-binding factor 1	FOXK2;ILF;ILF1	Isoform 1 of Forkhead	Q01167-1;Q01167;	3.07E-14	0.65303	0.79363	11.594	2	-0.33346
Nucleolar protein EMG1 homolog;Probable ribosome biogenesis protein NEP1;Protein C2f;cDNA FLJ76620,	C2F;EMG1	Probable ribosome bio	Q92979;A8K6D2;B4	3.10E-06	0.62091	0.79744	18.969	3	-0.32655
Deletion target in pancreatic carcinoma 4;Mothers against decapentaplegic homolog 4;SMAD family membe	DPC4;MADH4	Mothers against decap	Q13485;Q9BYG6;B4	2.72E-08	0.6275	0.80033		1	-0.32133
LDH heart subunit;L-lactate dehydrogenase B chain;Renal carcinoma antigen NY-REN-46;L-lactate dehydroge	LDHB;hCG_24	L-lactate dehydrogenas	P07195;Q5U077;A8	8.16E-17	0.61436	0.80374	101.16	4	-0.3152
NHP2 non-histone chromosome protein 2-like 1 (S. cerevisiae);High mobility group-like nuclear protein 2 ho	CTA-216E10.8	NHP2 non-histone chro	B1AHD1;P55769;Q	1.93E-07	0.66665	0.82214		1	-0.28254
Pescadillo homolog;cDNA, FLJ96584, Homo sapiens pescadillo homolog 1, containing BRCT domain(zebrafis	PES1;hCG_201	Isoform 1 of Pescadillo	O00541-1;O00541;	4.90E-18	0.60845	0.82551	57.269	7	-0.27664
FLYWCH family member 2	FLYWCH2	FLYWCH family membe	Q96CP2	0.016053	0.61261	0.83259		1	-0.26432
ATP-binding cassette 50;ATP-binding cassette sub-family F member 1;TNF-alpha-stimulated ABC protein;ATP	ABC50;ABCF1;	Isoform 1 of ATP-bindir	Q8NE71-1;Q8NE71	3.30E-131	0.55943	0.83481	111.71	29	-0.26048
Radixin isoform b;Radixin isoform e;Radixin isoform f;Radixin, isoform CRA_a;Radixin isoform d;Radixin;cDN	hCG_39182;RI	Radixin, isoform CRA_a	A7YIJ8;A7YIK3;P35	4.12E-13	0.63428	0.84468		1	-0.24352
Eukaryotic translation initiation factor 5B;Translation initiation factor IF-2;EIF5B protein;cDNA FLJ10524 fis, c	EIF5B;IF2;KIAA	Eukaryotic translation i	O60841;A0JLR8;B3	1.58E-10	0.66702	0.84802	221.19	3	-0.23783
CBF-1;J kappa-recombination signal-binding protein;RBP-J kappa;Recombining binding protein suppressor o	IGKJRB;IGKJRE	Isoform APCR-2 of Rec(	Q06330-1;Q06330;	0.0015342	0.70902	0.85491		1	-0.22616
L-lactate dehydrogenase;Cell proliferation-inducing gene 19 protein;LDH muscle subunit;L-lactate dehydrog	LDHA;PIG19;h	L-lactate dehydrogenas	B7Z5E3;P00338-1;F	4.30E-09	0.69554	0.86299		1	-0.21258
Activator 1 38 kDa subunit;Activator 1 subunit 3;Replication factor C 38 kDa subunit;Replication factor C sub	RFC3	Replication factor C sul	P40938;B4DKE6;Q5	2.73E-11	0.69231	0.86519		1	-0.20891
TTF-I-interacting peptide 20;Zinc finger protein 787	ZNF787	Zinc finger protein 787	Q6DD87	2.26E-06	0.72167	0.87537		1	-0.19204
Paraspeckle protein 2;RNA-binding motif protein 14;RNA-binding protein 14;RRM-containing coactivator act	RBM14;SIP	Isoform 1 of RNA-bindi	Q96PK6-1;Q96PK6;	2.34E-113	0.72993	0.87744	35.171	39	-0.18863
DNA-binding factor KBF2;H2TF1;Lymphocyte translocation chromosome 10 protein;Nuclear factor NF-kappa	LYT10;NFKB2;I	Isoform 1 of Nuclear fa	Q00653-1;Q00653;	0.0058312	0.62763	0.89003		1	-0.16807
40S ribosomal protein S9;Ribosomal protein S9;Ribosomal protein S9, isoform CRA_a;cDNA FLJ61636, highly	RPS9;hCG_20(	40S ribosomal protein	P46781;A9C4C1;B7	0.0023886	0.69696	0.89809		1	-0.15507
Cyclophilin B;CYP-S1;Peptidyl-prolyl cis-trans isomerase B;Rotamase B;S-cyclophilin	CYPB;PPIB	Peptidyl-prolyl cis-trans	P23284	8.36E-28	0.70893	0.89904	131.21	20	-0.15354
Cell division control protein 2 homolog;Cyclin-dependent kinase 1;p34 protein kinase;Putative uncharacteri	CDC2;DKFZp68	Putative uncharacterize	P06493-1;P06493;E	1.57E-09	0.72593	0.89991	7.1124	3	-0.15215
Sucrose nonfermenting protein 2 homolog;SWI/SNF-related matrix-associated actin-dependent regulator of	SMARCA5;SNF	SWI/SNF-related matri	O60264;B4DZC0;B7	2.30E-22	0.71575	0.90112	90.789	11	-0.15021
S1 RNA-binding domain-containing protein 1;cDNA FLJ31974 fis, clone NT2RP7008167, weakly similar to 85	SRBD1	Isoform 1 of S1 RNA-bi	Q8N5C6-1;Q8N5C6	4.98E-17	0.72445	0.9095	1.6967	2	-0.13685
Cytovillin;Ezrin;p81;Villin-2;cDNA, FLJ92973, highly similar to Homo sapiens villin 2 (ezrin) (VIL2), mRNA;cDN	EZR;VIL2	Ezrin;69 kDa protein	P15311;B2R6J2;B7	9.21E-19	0.70123	0.91062	89.403	9	-0.13508
cDNA FLJ78091, highly similar to Homo sapiens ubiquitin-like, containing PHD and RING finger domains, 1 (U	UHRF1;ICBP9(	E3 ubiquitin-protein lig	A8K024;B2RBA9;Q	8.34E-303	0.69597	0.91119	101.83	61	-0.13418
cDNA FLJ58953, highly similar to 40S ribosomal protein S20;40S ribosomal protein S20	RPS20	40S ribosomal protein	B4DW28;P60866;E	5.71E-10	0.73884	0.91348	110.32	7	-0.13055
C-2K;Cell division cycle 2-like protein kinase 4;Cell division protein kinase 9;Cyclin-dependent kinase 9;Serin	CDC2L4;CDK9;	Isoform 2 of Cyclin-dep	P50750-2;P50750;E	3.21E-05	0.7221	0.91834		1	-0.1229
Glutaminyl-peptide cyclotransferase-like protein	QPCTL	Glutaminyl-peptide cyc	Q9NXS2	0.0010763	0.73942	0.91973		1	-0.12072
Down-regulator of transcription 1;Negative cofactor 2-beta;Protein Dr1;TATA-binding protein-associated pho	DR1;DKFZp66(	Protein Dr1	Q01658;Q53F47;Q(	0.00015648	0.74286	0.91993		1	-0.1204
Androgen-induced proliferation inhibitor;Androgen-induced prostate proliferative shutoff-associated protei	APRIN;AS3;KIA	165 kDa protein;Isofor	Q9NTI5-1;Q9NTI5;(	1.23E-14	0.73323	0.92028	23.603	2	-0.11986
40S ribosomal protein S19-binding protein 1;Active regulator of SIRT1	AROS;RPS19B	Active regulator of SIRT	Q86WX3	2.51E-05	0.74651	0.92824		1	-0.10743
18 kDa Alu RNA-binding protein;Signal recognition particle 14 kDa protein	SRP14	Signal recognition part	P37108	1.39E-11	0.74794	0.9314	37.186	3	-0.10253
23 kDa highly basic protein;60S ribosomal protein L13a;RPL13A protein;Ribosomal protein L13a variant;FWI	RPL13A;hCG_1	60S ribosomal protein	P40429;Q0VGL3;Q5	0.087812	0.72747	0.93494	5.5781	2	-0.09705
Kinesin-like DNA-binding protein;Kinesin-like protein 4;Kinesin-like protein KIF22;cDNA FLJ53835, highly sim	KID;KIF22;KNS	Kinesin-like protein KIF	Q14807;B7Z265;B7	6.29E-127	0.78155	0.94994	117.79	29	-0.07409
Protein RCC2;RCC1-like protein TD-60;Telophase disk protein of 60 kDa;RCC2 protein	KIAA1470;RCC	Protein RCC2	Q9P258;A5PLK7	2.64E-50	0.73193	0.95118	122.23	20	-0.07221
SMARCA4 protein;SMARCA4 isoform 2;SWI/SNF related, matrix associated, actin dependent regulator of ch	SMARCA4;hC(	SMARCA4 protein;tran	B9EGQ8;Q9HBD4;E	1.26E-28	0.75978	0.95689	11.214	9	-0.06358
ESRRB protein;Estrogen receptor-related receptor beta2-delta-10;ERR beta-2;Estrogen receptor-like 2;Estrog	ESRRB;ERRB2;	steroid hormone recep	A2VDJ2;Q5F0P7;O9	0.0075667	0.72634	0.95795	1.825	2	-0.06198
60S ribosomal protein L13;Breast basic conserved protein 1;Ribosomal protein L13	BBC1;OK/SW-	60S ribosomal protein	P26373;A8K4C8;B4	4.13E-17	0.75668	0.97157	15.4	6	-0.04161
DNA mismatch repair protein Msh2;MutS protein homolog 2;cDNA FLJ57316, highly similar to DNA mismatc	MSH2	DNA mismatch repair p	P43246;B4DL39;B4	7.97E-27	0.75626	0.97247	63.057	10	-0.04027
DNA-dependent protein kinase catalytic subunit;DNPK1;p460;cDNA FLJ57825, highly similar to DNA-depenc	HYRC;HYRC1;F	Isoform 1 of DNA-depe	P78527-1;P78527;F	4.65E-16	0.78394	0.97739	68.77	6	-0.03299
Importin subunit beta-1;Importin-90;Karyopherin subunit beta-1;Nuclear factor p97;Pore targeting complex	KPNB1;NTF97	Importin subunit beta-	Q14974;B2RBR9;B7	7.12E-08	0.77766	0.98508	11.236	3	-0.02169

Excitatory amino acid transporter 1;Sodium-dependent glutamate/aspartate transporter 1;Solute carrier far	EAAT1;GLAST;	Excitatory amino acid t	P43003;Q8N169;Q	0.0062299	0.6958	0.98975		1	-0.01486
Fox-1 homolog B;Hexaribonucleotide-binding protein 2;Repressor of tamoxifen transcriptional activity;RNA-	FOX2;HRNBP2	Isoform 8 of RNA bindi	O43251-8;O43251;	1.16E-16	0.79004	0.99268	50.226	3	-0.0106
RCC1-I;Regulator of chromosome condensation 1;Putative uncharacterized protein RCC1;Cell cycle regulato	RCC1;CHC1;h(	regulator of chromoso	Q16269;Q6NT97;C	2.52E-134	0.79286	0.99339	117.13	21	-0.00957
Elongation factor 2;cDNA FLJ60696, highly similar to Elongation factor 2;cDNA FLJ58164, highly similar to El	EEF2;EF2	Elongation factor 2;cD	P13639;B4DMC6;B	5.88E-11	0.82347	1.0027	73.199	5	0.00389
40S ribosomal protein S25	RPS25	40S ribosomal protein	P62851	4.60E-07	0.77529	1.0052	85.111	5	0.007483
Hairy cell leukemia protein 1;Ribosome biogenesis protein NSA2 homolog;TGF-beta-inducible nuclear prote	HUSSY-29;NSA	Ribosome biogenesis p	O95478;Q5J7U2	0.00045513	0.82193	1.0054		1	0.00777
56 kDa U2AF65-associated protein;ATP-dependent RNA helicase p47;DEAD box protein UAP56;HLA-B-associ	BAT1;UAP56;X	Isoform 2 of Spliceosor	Q13838-2;Q13838;	7.42E-17	0.81485	1.0139	77.286	7	0.019915
CCT-delta;Stimulator of TAR RNA-binding;T-complex protein 1 subunit delta	CCT4;CCTD;SR	T-complex protein 1 su	P50991;A8K3C3;B7	0.0010396	0.75528	1.0177		1	0.025312
Annexin A2;Annexin II;Annexin-2;Calpactin I heavy chain;Calpactin-1 heavy chain;Chromobindin-8;Lipocorti	ANX2;ANX2L4	Isoform 2 of Annexin A	P07355-2;P07355;	5.32E-45	0.75938	1.0182	68.804	5	0.026021
Leucine-rich repeat-containing protein 59;cDNA, FLJ95955	LRRC59;PRO1	Leucine-rich repeat-co	Q96AG4;B2RE83;D	5.58E-11	0.76328	1.0224	110.39	5	0.03196
40S ribosomal protein S14	PRO2640;RPS	40S ribosomal protein	P62263;E5RH77	0.0092178	0.83296	1.0373		1	0.052833
Nuclear receptor subfamily 2 group F member 6;V-erbA-related protein 2;cDNA FLJ51763, highly similar to (	EAR2;ERBAL2;	Nuclear receptor subfa	P10588;B4DQE4;B	3.62E-07	0.82796	1.0447	63.33	3	0.063089
APEX nuclease;Apurinic-apyrimidinic endonuclease 1;DNA-(apurinic or apyrimidinic site) lyase;Protein REF-1	APE;APEX;APE	DNA-(apurinic or apyri	P27695;Q5TZP7	3.23E-161	0.84311	1.0449	126.46	31	0.063365
214 kDa nucleoporin;Nuclear pore complex protein Nup214;Nucleoporin Nup214;Protein CAN;cDNA FLJ523	CAIN;CAN;KIA	Isoform 3 of Nuclear p	P35658-3;P35658;	1.03E-77	0.8177	1.0473	140.42	19	0.066675
Cell proliferation-inducing gene 21 protein;Guanine nucleotide-binding protein subunit beta-2-like 1;Guanin	GNB2L1;HLC7	Guanine nucleotide-bi	P63244;D6R9L0;D6	3.33E-08	0.84464	1.0487	4.9983	2	0.068602
Bromodomain adjacent to zinc finger domain protein 1B;hWALp2;Tyrosine-protein kinase BAZ1B;Williams s	BAZ1B;WBSC1	Isoform 1 of Tyrosine-p	Q9UIG0-1;Q9UIG0;	4.04E-60	0.83297	1.0519	118.57	11	0.072998
40S ribosomal protein S8;Ribosomal protein S8;Similar to ribosomal protein S8	OK/SW-cl.83;	40S ribosomal protein	P62241;Q5JR94;Q5	5.04E-21	0.82342	1.0552	6.6342	2	0.077516
Ally of AML-1 and LEF-1;bZIP-enhancing factor BEF;THO complex subunit 4;Transcriptional coactivator Aly/R	ALY;BEF;THOC	THO complex subunit 4	Q86V81	4.13E-11	0.75781	1.0588	9.1553	2	0.08243
G/T mismatch-specific thymine DNA glycosylase;Thymine-DNA glycosylase;cDNA, FLJ93738, highly similar to	TDG	G/T mismatch-specific	Q13569;B2R848;B4	5.91E-09	0.89091	1.061	120.92	5	0.085425
60S ribosomal protein L19;Ribosomal protein L19	RPL19	60S ribosomal protein	P84098;Q53G49;Q	1.58E-12	0.83229	1.0634	39.276	3	0.088684
Hepatocellular carcinoma-associated antigen 90;TPX2, microtubule-associated, homolog (Xenopus laevis);T	HCA90;hCG_3	TPX2, microtubule-ass	Q96RR5;Q9ULW0;	5.40E-09	0.844	1.0727	5.7337	2	0.101247
NAD(+) ADP-ribosyltransferase 2;Poly [ADP-ribose] polymerase 2;Poly[ADP-ribose] synthase 2;cDNA FLJ755(	ADPRT2;ADPR	Isoform 1 of Poly [ADP-	Q9UGN5-1;Q9UGN	2.56E-07	0.79715	1.0769	166.99	3	0.106884
Antiapoptosis clone 11 protein;Apoptosis inhibitor 5;Cell migration-inducing gene 8 protein;Fibroblast grow	API5;MIG8	apoptosis inhibitor 5 is	Q9BZZ5-4;Q9BZZ5;	2.05E-13	0.88943	1.086	10.225	4	0.119024
Interleukin enhancer-binding factor 2;Nuclear factor of activated T-cells 45 kDa;cDNA FLJ51660, highly simil	ILF2;NF45;PR(	Interleukin enhancer-b	Q12905;B4DY09;Q	4.11E-18	0.88134	1.0885	73.433	4	0.122341
Histone H2A.x;Histone H2A type 1-A;Histone H2A/r;Histone H2A type 1;Histone H2A/p;Histone H2A;Histone	H2AFX;H2AX;	Histone H2A.x;Histone	P16104;Q96QV6;P(	2.68E-18	0.88644	1.089	191.73	3	0.123004
Cytokine-induced protein of 29 kDa;Nuclear protein Hcc-1;Proliferation-associated cytokine-inducible prote	HCC1;HSPC31	52 kDa protein;SAP do	P82979;Q567R9;A4	9.26E-07	0.81997	1.0982	6.2426	2	0.135141
E3 ubiquitin-protein ligase UHRF2;Np95/ICBP90-like RING finger protein;Nuclear protein 97;Nuclear zinc fin	NIRF;RNF107;	Isoform 1 of E3 ubiquit	Q96PU4-1;Q96PU4	9.82E-24	0.77359	1.1098	31.451	6	0.1503
Transcription factor Sp1;Specificity protien 1;Sp1 transcription factor variant	SP1;TSFP1;Sp	Transcription factor Sp	P08047;Q9NR52;C4	9.91E-10	0.91653	1.1135		1	0.155102
GCN1-like protein 1;Translational activator GCN1;cDNA FLJ54481, highly similar to GCN1-like protein 1	GCN1L1;KIAA(	Translational activator	Q92616;B4DM32;E	7.27E-05	0.81923	1.1293	4.6907	2	0.175429
ATP-dependent helicase CHD4;Chromodomain-helicase-DNA-binding protein 4;Mi-2 autoantigen 218 kDa pr	CHD4;CHD3	Isoform 2 of Chromodo	Q14839-2;Q14839;	0.036417	0.88663	1.1325	2.456	2	0.179511
1-phosphatidylinositol-4,5-bisphosphate phosphodiesterase beta-3;Phosphoinositide phospholipase C-beta	PLCB3	1-phosphatidylinositol-	Q01970;Q8N1A4	2.67E-37	0.85112	1.1362	11.312	6	0.184217
Transcription factor MafF;U-Maf;V-maf musculoaponeurotic fibrosarcoma oncogene homolog F;V-maf musc	MAFF;CTA-44	Transcription factor Ma	Q9ULX9;B0QY71;B	0.0052298	0.90053	1.1399	23.25	2	0.188907
60S ribosomal protein L27a;Ribosomal protein L27a	RPL27A;L27a	60S ribosomal protein	P46776;Q6NZ52;Q	2.02E-06	0.92662	1.1515		1	0.203514
Treacher Collins syndrome protein;Treacle protein;cDNA FLJ57828, highly similar to Treacle protein;TCOF1 p	TCOF1;DKFZp	Isoform 4 of Treacle pr	Q13428-4;Q13428;	1.77E-09	0.93359	1.1634		1	0.218347
Putative uncharacterized protein ENSP00000346598;37 kDa laminin receptor precursor;37/67 kDa laminin r	LAMBR;LAMR	33 kDa protein;Unchar	A6NE09;P08865;Q	0.00050167	0.93928	1.1664		1	0.222063
Cell proliferation-inducing gene 54 protein;Sister chromatid cohesion protein 112;Sister chromatid cohesion	KIAA0648;PDS	Isoform 1 of Sister chr	Q29RF7-1;Q29RF7	6.38E-26	0.94192	1.1727	47.972	5	0.229834
Adenine nucleotide translocator 2;ADP,ATP carrier protein 2;ADP,ATP carrier protein, fibroblast isoform;ADP	ANT2;SLC25A	ADP/ATP translocase 2;	P05141;B2RCV1;Q	1.65E-10	0.92052	1.174	83.485	10	0.231432
CXXC-type zinc finger protein 9;DNA (cytosine-5)-methyltransferase 1;DNA methyltransferase HsaI;MCMT;C	AIM;CXXC9;D	Isoform 1 of DNA (cyto	P26358-1;P26358;	2.00E-48	0.94226	1.174	83.532	19	0.231432
Gamma-1-tubulin;Gamma-tubulin complex component 1;Tubulin gamma-1 chain;Gamma-2-tubulin;Tubulin	TUBG;TUBG1;	Tubulin gamma-1 chair	P23258;Q9NRH3	1.90E-28	0.91542	1.1901	77.689	10	0.251083
DEAD/H box-binding protein 1;E3 SUMO-protein ligase PIAS1;Gu-binding protein;Protein inhibitor of activat	DDXBP1;PIAS1	cDNA FLJ37322 fis, clo	O75925;A4ZVS7;A4	0.0030728	0.96349	1.1914		1	0.252658
Asparagine--tRNA ligase;Asparaginyl-tRNA synthetase, cytoplasmic;cDNA FLJ52703, highly similar to Aspara	ASNS;NARS	Asparaginyl-tRNA synth	O43776;B4DN60;B	0.0101	0.97284	1.2044	31.015	3	0.268315
ATP-dependent RNA helicase SUPV3L1, mitochondrial;Suppressor of var1 3-like protein 1;cDNA FLJ50848, hi	SUPV3L1;SUV	ATP-dependent RNA he	Q8IYB8;B7Z611	2.11E-36	0.99057	1.2054	18.262	9	0.269512
Lon protease homolog, mitochondrial;Lon protease-like protein;LONHs;Mitochondrial ATP-dependent prote	LONP1;PRSS1	Lon protease homolog,	P36776;E5KMH8;E	6.29E-06	0.98342	1.2057	103.82	3	0.269871
Sex comb on midleg-like protein 2;cDNA FLJ54781, highly similar to Sex comb on midleg-like protein 2;cDNA	SCML2;RP5-1	Isoform 1 of Sex comb	Q9UQR0-1;Q9UQR	6.38E-08	0.93657	1.2218	24.697	5	0.289008
ULF;TRIP12 protein;Probable E3 ubiquitin-protein ligase TRIP12;Thyroid receptor-interacting protein 12;Puta	TRIP12;KIAA0	TRIP12 protein;Probab	D4HL82;Q14CA3;Q	4.05E-05	0.90068	1.2258	8.802	2	0.293724
Mediator of DNA damage checkpoint protein 1;Nuclear factor with BRCT domains 1;Mediator of DNA dama	KIAA0170;MD	Isoform 1 of Mediator	Q14676-1;Q14676;	1.26E-68	0.97068	1.227	115.08	11	0.295135
3'-5' exonuclease TREX1;DNase III;Three prime repair exonuclease 1;TREX1 protein;Three prime repair exon	TREX1;hCG_1	Isoform 1 of Three prin	Q9NSU2-1;Q9NSU	1.71E-36	1.0108	1.2313	72.827	10	0.300182
Endonuclease III-like protein 1	NTH1;NTHL1;	Endonuclease III-like p	P78549;E5KTI5	4.69E-41	1.0132	1.263	32.497	12	0.336855
Phosphate carrier protein, mitochondrial;Phosphate transport protein;Solute carrier family 25 member 3;cD	OK/SW-cl.48;	Isoform A of Phosphate	Q00325-1;Q00325;	2.76E-11	1.0475	1.2873		1	0.364348
Lamina-associated polypeptide 2, isoforms beta/gamma;Splenin;Thymopentin;Thymopoietin;Thymopoietin	LAP2;TMPO	Isoform Beta of Lamina	P42167-1;P42167;	2.80E-60	0.99137	1.2948	85.098	16	0.372729
Mannosyl-oligosaccharide glucosidase;Processing A-glucosidase I;Glucosidase I;cDNA FLJ75504, highly simil	GCS1;MOGS	Mannosyl-oligosacchar	Q13724;Q58F09;A	0.0011441	1.0381	1.3147		1	0.394734
Divergent upstream protein;DNA mismatch repair protein Msh3;Mismatch repair protein 1;MutS homolog 3	DUC1;DUG;M	DNA mismatch repair p	P20585;A1L480;A8	8.52E-05	1.0459	1.3174	25.324	3	0.397693
Cell division protein kinase 2;p33 protein kinase;D-HSCDK2;cDNA FLJ54979, highly similar to Homo sapiens	CDK2;CDK2de	Cyclin-dependent kina	P24941;O75100;B4	0.00012992	0.97649	1.3248		1	0.405775
3-alkyladenine DNA glycosylase;3-methyladenine DNA glycosidase;ADPG;DNA-3-methyladenine glycosylase	AAG;ANPG;M	Isoform 2 of DNA-3-me	P29372-2;P29372;	1.90E-87	0.99798	1.3259	74.13	37	0.406972
Patatin-like phospholipase domain-containing protein 4;Protein GS2;Putative uncharacterized protein PNPL	DXS1283E;GS	Patatin-like phospholip	P41247;C9IZF6;B4	0.00014715	1.0277	1.3264	2.1196	2	0.407516
47 kDa heat shock protein;Arsenic-transactivated protein 3;Cell proliferation-inducing gene 14 protein;Colla	CBP1;CBP2;H	Serpin H1;cDNA FLJ52	P50454;A8K259;Q	0.0033698	0.93081	1.34		1	0.422233
cDNA FLJ59630, highly similar to Growth hormone-inducible transmembrane protein;GHITM protein;Derma	GHITM;DERP2	cDNA FLJ59630, highly	B4DPG9;Q6FIA7;Q	0.10256	1.0356	1.3463		1	0.429
Zinc finger protein 91 homolog;cDNA FLJ57065, highly similar to Zinc finger protein 91	FKSG11;ZFP91	Isoform 1 of E3 ubiquit	Q96JP5-1;Q96JP5;	3.80E-07	0.87031	1.3601	32.514	2	0.443713
Prohibitin;cDNA FLJ78511, highly similar to Homo sapiens prohibitin (PHB), mRNA;cDNA, FLJ93035, Homo s	PHB;hCG_296	Prohibitin;Uncharacter	P35232;A8K401;Q5	0.00049622	1.0753	1.3953		1	0.480575
Coiled-coil domain-containing protein 124	CCDC124	Coiled-coil domain-con	Q96CT7	4.86E-12	0.96295	1.4361	17.244	7	0.522156
Histone H3.3;Histone H3;Histone H3.1;Histone H3/a;Histone H3/b;Histone H3/c;Histone H3/d;Histone H3/f;	H3.3A;H3.3B;	Histone H3.3;cDNA, FL	P84243;B2R4P9;B2	8.01E-06	1.0843	1.4921	20.471	5	0.577344

Valyl-tRNA synthetase;Protein G7a;Valine--tRNA ligase	DAAP-21F2.2-	Valyl-tRNA synthetase;	B0V043;P26640	1.56E-06	1.1979	1.5348	12.906	4	0.618051
UPF0609 protein C4orf27	C4orf27	UPF0609 protein C4orf	Q9NWY4	2.86E-16	1.226	1.6361	142.54	11	0.710261
BPY2-interacting protein 1;MAP1S heavy chain;MAP1S light chain;Microtubule-associated protein 1S;Microt	BPY2IP1;C19o	Microtubule-associate	Q66K74;A8K940;B4	0.00066442	0.98408	1.6426		1	0.715981
40S ribosomal protein S7;cDNA, FLJ92055, Homo sapiens ribosomal protein S7 (RPS7), mRNA;Putative unch	RPS7;hCG_17	40S ribosomal protein	P62081;Q57Z92;B5	1.67E-05	1.2999	1.8254	84.113	2	0.868213
cDNA FLJ51656, highly similar to Actin-like protein 2;Actin-like protein 2;Actin-related protein 2;ACTR2 prot	ACTR2;ARP2	actin-related protein 2	B4DHK9;P61160;Q	0.016945	1.3077	1.8801		1	0.910809
Transcription factor jun-D	JUND	Transcription factor jur	P17535	6.62E-15	1.5638	1.9391	108.23	4	0.955387
Rap1-interacting factor 1 homolog;Telomere-associated protein RIF1	RIF1	Isoform 1 of Telomere-	Q5UIP0-1;Q5UIP0;	0.0061568	1.4995	2.1479		1	1.102927
62 kDa nucleoporin;Nuclear pore glycoprotein p62;Nucleoporin Nup62;Putative uncharacterized protein pp	NUP62;pp705	Nuclear pore glycoprot	P37198;Q8WYU3	0.004906	1.8031	2.58		1	1.367371
Transcription factor jun-B;Jun B proto-oncogene variant;cDNA, FLJ94337, Homo sapiens jun B proto-oncoge	JUNB;hCG_17	Transcription factor jur	P17275;Q53HP8;Q	4.29E-18	2.1192	2.6232	84.736	3	1.391328
18S rRNA dimethylase;DIM1 dimethyladenosine transferase 1-like;Probable dimethyladenosine transferase;	DIMT1;DIMT1	Probable dimethylader	Q9UNQ2;A8K9K8;E	0.002424	2.0672	3.0832		1	1.624428
Cyclic AMP-responsive element-binding protein 1;cAMP responsive element binding protein 1;CAMP respor	CREB1;hCG_1	Isoform CREB-A of Cycl	P16220-1;P16220;	4.17E-14	2.7056	3.3213	19.383	3	1.731748
cDNA FLJ54188, moderately similar to High mobility group protein HMG-I/HMG-Y;Putative uncharacterized	HMGA1;HMG	cDNA FLJ54188, moder	B4DWA0;C9J292;P	0.0058359	2.7178	3.9783		1	1.992152
Probable tumor suppressor protein MN1;Meningioma (Disrupted in balanced translocation) 1	MN1	Meningioma (Disrupte	Q10571;A5HML1	0.12914	3.5346	4.441	289.97	2	2.150885
Outer mitochondrial membrane protein porin 2;Voltage-dependent anion-selective channel protein 2;cDNA	VDAC2;RP11-	Isoform 1 of Voltage-d	P45880-1;P45880;	0.025034	4.2551	6.0233		1	2.590554

Supplementary Table 2 B: ODN-to-protein conjugates identified by tandem mass spectrometry and quantified using SILAC (hmC=heavy; C=light)

Protein Names	Gene Names	Protein Descriptions	Uniprot	Mol. Weight [kDa]	PEP	Ratio H/L	Ratio H/L Normalized	Ratio H/L Variability [%]	atio H/L Cou	Log2(H/L)
Arginine/proline-rich coiled-coil domain-containing protein 1;MAP7 domain-containing protein 1;P	KIAA1187;MAP7D1;PARCC1;PP2464;RPRC1;I	Isoform 1 of MAP7 domain-containing protein 1;Isoforn	Q3KQU3-1;Q3KQL	92.819	0.0050669	16.103	22.347	95.012	2	4.4820093
cDNA FLJ58590, highly similar to YLP motif-containing protein 1;YLPM1 protein;FLJ00353 protein;N	YLPM1;FLJ00353;C14orf170;ZAP3	YLP motif-containing protein 1;Isoform 1 of YLP motif-cc	B4DMQ9;Q86YA8;	241.64	1.21E-07	5.1737	5.0139	30.67	3	2.3259332
Bcl-2-associated transcription factor 1;BCLAF1 protein;cDNA, FLJ79533, highly similar to Bcl-2-asso	BCLAF1;BTF;KIAA0164	Isoform 1 of Bcl-2-associated transcription factor 1;Isofc	Q9NYF8-1;Q9NYF	106.12	0.024252	5.0929	4.3068	40.879	2	2.1066163
Testis-expressed sequence 10 protein;cDNA FLJ54377;cDNA FLJ57815;cDNA FLJ50343;cDNA FLJ50	L18;Nbla10363;TEX10	Testis-expressed sequence 10 protein;testis-expressed s	Q9NXF1;B4DQR0;	105.67	2.34E-05	3.0351	3.4481	23.068	3	1.7858016
Scaffold attachment factor B2;SAFB2 protein	KIAA0138;SAFB2	Scaffold attachment factor B2	Q14151;A0PJ47	107.47	0.0030361	3.4403	3.0293		1	1.5989845
rRP12-like protein;cDNA FLJ12434 fis, clone NT2RM1000037, highly similar to Homo sapiens KIAA0	TOP2B;topIIb;TOP2A;TOP2;top2beta	Isoform Beta-2 of DNA topoisomerase 2-beta;Isoform B	Q02880-1;Q0288(	183.26	1.60E-12	2.9181	2.8733	39.254	2	1.5227086
Cleavage and polyadenylation specificity factor 100 kDa subunit;Cleavage and polyadenylation spe	CPSF100;CPSF2;KIAA1367	Cleavage and polyadenylation specificity factor subunit	Q9P2I0;B3KN45	88.486	6.35E-05	2.6873	2.8419		1	1.5068558
cDNA FLJ32832 fis, clone TESTI2003196;Embryonic large molecule derived from yolk sac;Protein EL	AHCTF1;ELYS;MSTP108;TMBS62	protein ELYS;Isoform 2 of Protein ELYS;Isoform 1 of Prot	Q96M44;Q8WYP5	256.24	4.33E-08	3.6765	2.8287		1	1.5001392
60S ribosomal protein L15;Ribosomal protein L15	EC45;RPL15;TCBAP0781;hCG_1996483	60S ribosomal protein L15;21 kDa protein;cDNA FLJ514	P61313;Q5U0C0;E	24.146	0.00019599	3.5637	2.7977		1	1.4842413
60S ribosomal protein L10;Laminin receptor homolog;Protein QM;Tumor suppressor QM;Ribosoma	DXS648E;QM;RPL10;XX-FW83563B9.1-011;F	60S ribosomal protein L10;60S ribosomal protein L10-lil	P27635;Q5HY50;C	24.604	0.26025	2.8193	2.7556		1	1.4623665
One-twenty two protein 1;Putative RNA-binding protein 15;RNA-binding motif protein 15;RNA binc	OTT;OTT1;RBM15	Isoform 1 of Putative RNA-binding protein 15;Isoform 3	Q96T37-1;Q96T37	107.19	0.0010035	3.3142	2.7486	55.228	2	1.458697
68 kDa TATA-binding protein-associated factor;RNA-binding protein 56;TATA-binding protein-associ	RBP56;TAF15;TAF2N;hCG_1992163	Isoform Long of TATA-binding protein-associated factor	Q92804-1;Q92804	61.829	3.29E-35	4.2848	2.6415		1	1.4013574
ATP-dependent RNA helicase DDX42;DEAD box protein 42;RNA helicase-like protein;RNA helicase-r	DDX42	Isoform 1 of ATP-dependent RNA helicase DDX42;Isofor	Q86XP3-1;Q86XP3	102.97	9.65E-07	2.8963	2.6245		1	1.3920426
Zinc finger protein 326	ZNF326	Isoform 1 of Zinc finger protein 326	Q5BKZ1-1;Q5BKZ1	65.653	3.03E-06	2.7956	2.6177	37.191	3	1.3882998
Modulator of estrogen-induced transcription;SAFB-like transcription modulator;cDNA FLJ30633 fis,	MET;SLTM	SAFB-like transcription modulator;SAFB-like transcriptio	Q9NWH9;A8K5V8	117.15	0.14794	3.1228	2.6002		1	1.3786226
Coilin;p80	CLN80;COIL	Coilin	P38432	62.608	6.18E-07	2.4354	2.5992	25.358	3	1.3780676
Cell proliferation-inducing gene 4/52 protein;Mitochondrial inner membrane protein;Mitofilin;p87,	HMP;IMMT;PIG4;PIG52	Isoform 1 of Mitochondrial inner membrane protein;Iso	Q16891-1;Q16891	83.677	0.022615	2.3239	2.5898	51.17	2	1.3728407
Apoptotic chromatin condensation inducer in the nucleus;ACIN1 protein;cDNA FLJ50838, highly sir	ACIN1;ACINUS;KIAA0670;DKFZp667N107	Isoform 1 of Apoptotic chromatin condensation inducer	Q9UKV3-1;Q9UKV	151.86	5.52E-44	3.3541	2.5737	36.863	6	1.3638439
Emerin;EMD protein;Emerin (Emery-Dreifuss muscular dystrophy), isoform CRA_a;Emerin (Emery-I	EDMD;EMD;STA;hCG_41343;XX-FW88778H2	Emerin;Emerin (Emery-Dreifuss muscular dystrophy), is	P50402;Q6FI02;Q	28.994	6.91E-08	3.2088	2.5342	6.3089	2	1.3415304
Thyroid hormone receptor-associated protein 3;Thyroid hormone receptor-associated protein com	THRAP3;TRAP150	Thyroid hormone receptor-associated protein 3	Q9Y2W1;Q05D20;	108.66	2.14E-05	2.6093	2.484	33.808	2	1.3126652
Cleavage and polyadenylation specificity factor 160 kDa subunit;Cleavage and polyadenylation spe	CPSF1;CPSF160;hCG_2039719;P/cl.18	Cleavage and polyadenylation specificity factor subunit	Q10570;B4DEF4;D	160.88	7.08E-08	2.6146	2.4789		1	1.3097001
60S ribosomal protein L18;Ribosomal protein L18	RPL18	60S ribosomal protein L18;cDNA FLJ59178, moderately	Q07020;Q0QEW2	21.634	2.30E-21	2.5148	2.3512	6.7436	4	1.2333973
Modulator of non-genomic activity of estrogen receptor;Proline-, glutamic acid- and leucine-rich pr	HMX3;MNAR;PELP1	Isoform 2 of Proline-, glutamic acid- and leucine-rich pr	Q8IZL8-2;Q8IZL8;(	135.49	4.16E-07	2.1986	2.3059	12.623	4	1.2053299
40 kDa SR-repressor protein;FUS-interacting serine-arginine-rich protein 1;Splicing factor SRp38;Sp	FUSIP1;FUSIP2;SFRS13A;TASR;RP11-4M23.6	Isoform 1 of Serine/arginine-rich splicing factor 10;Isofo	O75494-1;O75494	31.3	2.16E-18	2.6556	2.3047	9.5842	5	1.204579
NUMA1 variant protein;Nuclear mitotic apparatus protein 1;SP-H antigen;cDNA FLJ76708, highly si	NUMA1 variant protein;NUMA;NUMA1;NuM	NUMA1 variant protein (Fragment);Isoform 1 of Nuclea	Q4LE64;Q14980-1	238.86	6.08E-65	2.9051	2.3027	16.086	8	1.2033265
Activating enhancer-binding protein 2-delta;Transcription factor AP-2-beta-like 1;Transcription fact	TFAP2BL1;TFAP2D	Transcription factor AP-2-delta	Q7Z6R9	49.577	0.004079	2.4548	2.2633		1	1.1784278
Outer mitochondrial membrane protein porin 2;Voltage-dependent anion-selective channel protei	VDAC2;RP11-375G3.1-010;RP11-375G3.1-00	Isoform 1 of Voltage-dependent anion-selective channe	P45880-1;P45880	33.371	0.014099	2.8908	2.2433		1	1.1656226
220 kDa U5 snRNP-specific protein;p220;Pre-mRNA-processing-splicing factor 8;PRP8 homolog;Spli	PRPC8;PRPF8	Pre-mRNA-processing-splicing factor 8	Q6P2Q9;B4DK16;(	273.6	2.30E-17	3.0826	2.2168	21.213	9	1.1484786
AIR carboxylase;Multifunctional protein ADE2;Phosphoribosylaminoimidazole carboxylase;Phosphc	ADE2;AIRC;PAICS;PAIS;DKFZp781N1372	Multifunctional protein ADE2;multifunctional protein AI	P22234;Q68CQ5;E	49.679	1.56E-15	2.44	2.2138	1.1524	2	1.1465249
Transformer-2 protein homolog A;Transformer-2 protein homolog alpha;cDNA FLJ50614, highly sim	TRA2A;hCG_37979;HSU53209	Isoform Long of Transformer-2 protein homolog alpha;I	Q13595-1;Q13595	32.688	0.0017494	2.4739	2.2015		1	1.1384868
cDNA FLJ76836, highly similar to Homo sapiens splicing factor, arginine/serine-rich 14 (SFRS14), tra	KIAA0365;SFRS14	cDNA FLJ76836, highly similar to Homo sapiens splicing	A8K5G0;Q8IX01-1	121.31	0.016807	2.2552	2.1466		1	1.1020534
300 kDa nuclear matrix antigen;Serine/arginine repetitive matrix protein 2;Serine/arginine-rich spli	HSPC075;KIAA0324;SRL300;SRM300;SRRM2	Isoform 1 of Serine/arginine repetitive matrix protein 2	Q9UQ35-1;Q9UQ3	299.61	0.0022308	2.498	2.1354		1	1.0945063
EPB72-like protein 2;Stomatin-like protein 2;cDNA FLJ61039, highly similar to Stomatin-like protein	HSPC108;SLP2;STOML2	Stomatin-like protein 2;cDNA FLJ61039, highly similar tc	Q9UJZ1;B4E1K7	38.534	0.021587	2.0046	2.1276		1	1.0892269
ATP-dependent RNA helicase p54;DEAD box protein 6;Oncogene RCK;Probable ATP-dependent RN/	DDX6;HLR2;RCK	Probable ATP-dependent RNA helicase DDX6	P26196;B2R858;Q	54.416	0.0049494	2.4675	2.1181		1	1.0827707
RRP12-like protein;cDNA FLJ12434 fis, clone NT2RM1000037, highly similar to Homo sapiens KIAA(	KIAA0690;RRP12	Isoform 1 of RRP12-like protein;RRP12-like protein isofc	Q5JTH9-1;Q5JTH9	143.7	1.98E-08	2.2805	2.1031		1	1.0725175
100 kDa coated vesicle protein A;Adapter-related protein complex 2 alpha-1 subunit;Adaptor prote	ADTAA;AP2A1;CLAPA1	Isoform A of AP-2 complex subunit alpha-1;108 kDa pro	O95782-1;O95782	107.54	0.005984	2.2419	2.0987	1.2092	2	1.069496
Nucleoporin NUP188 homolog	KIAA0169;NUP188	Isoform 1 of Nucleoporin NUP188 homolog;Isoform 2 o	Q5SRE5-1;Q5SRE5	196.04	0.0010557	2.0086	2.0874		1	1.0617071
Glycoprotein p43;Heterogeneous nuclear ribonucleoprotein G;Heterogeneous nuclear ribonucleop	HNRPG;RBMX;RBMXP1;CCBL2;DKFZp547N1	Heterogeneous nuclear ribonucleoprotein G;cDNA FLJ3	P38159;B4E352;B	42.331	3.71E-20	2.4543	2.0681	57.462	18	1.0483059
G patch domain-containing protein 9;RNA-binding motif protein 10;RNA-binding protein 10;RNA-bi	DXS8237E;GPATC9;GPATCH9;KIAA0122;RBM	Putative uncharacterized protein DKFZp686E2459;Isofo	P98175-1;P98175	110.34	1.40E-05	3.1403	2.0527	25.434	3	1.0375228
U1 small nuclear ribonucleoprotein 70 kDa;cDNA FLJ77404, highly similar to Homo sapiens small n	RNPU1Z;RPU1;SNRNP70;SNRP70;U1AP1;DKI	Isoform 1 of U1 small nuclear ribonucleoprotein 70 kDa	P08621-1;P08621	51.556	1.29E-15	2.3944	2.0519	13.374	7	1.0369604
ATP-dependent RNA helicase A;DEAH box protein 9;Nuclear DNA helicase II;cDNA FLJ39263 fis, clor	DDX9;DHX9;LKP;NDH2	ATP-dependent RNA helicase A	Q08211;B3KU66;C	140.96	8.68E-147	2.9925	2.0429	81.264	41	1.0306186
Cytochrome c oxidase polypeptide II;Cytochrome c oxidase subunit 2;Cytochrome c oxidase subuni	COII;COXII;MTCO2;MT-CO2;COX2;CO2	Cytochrome c oxidase subunit 2	P00403;A0S0W7;/	25.565	1.99E-05	2.5888	2.0351		1	1.0250997
Hemidesmosomal protein 1;Plectin-1;HCG1994702;Plectin isoform 1b;Plectin 1, intermediate filam	PLEC1;hCG_1994702;hCG_1994701	Isoform 1 of Plectin;Isoform 2 of Plectin;Isoform 3 of Ple	Q15149-1;Q15149	531.78	0.0042976	1.7844	2.0309		1	1.0221192
Oculospanin;Tetraspanin-10;TSPAN10 protein	OCSP;TSPAN10	Tetraspanin-10	Q9H1Z9;Q6PJ65	36.498	0.0037661	2.8974	2.0235	11.164	2	1.0168528
Small nuclear ribonucleoprotein Sm D1;Sm-D autoantigen;snRNP core protein D1	SNRPD1	Small nuclear ribonucleoprotein Sm D1;Small nuclear ri	P62314;Q7Z5A3	13.281	4.54E-09	1.6183	2.0203	131.03	3	1.0145695
Heterogeneous nuclear ribonucleoprotein M;Heterogeneous nuclear ribonucleoprotein M isoform	HNRNPM;HNRPM;NAGR1;ORF;DKFZp547H1	Isoform 1 of Heterogeneous nuclear ribonucleoprotein	P52272-1;P52272	77.515	2.05E-121	2.741	2.0114	55.273	75	1.0082
Dolichyl-phosphate mannosyltransferase polypeptide 1, catalytic subunit;Dolichol-phosphate manr	DPM1;RP5-914P20.2-003;RP5-914P20.2-005	Dolichyl-phosphate mannosyltransferase polypeptide 1,	Q5QPK0;Q5QPK2;	33.349	0.019022	2.4447	2.0082		1	1.005903
hTREX84;Nuclear matrix protein p84;THO complex subunit 1	HPR1;THOC1	Isoform 1 of THO complex subunit 1;Isoform 2 of THO c	Q96FV9-1;Q96FV9	75.665	2.16E-29	2.2217	2.0012	35.225	8	1.0008654
D-3-phosphoglycerate dehydrogenase;cDNA FLJ35987 fis, clone TESTI2014269, highly similar to D-3	PGDH3;PHGDH;RP4-683H9.1-002	D-3-phosphoglycerate dehydrogenase;Phosphoglycerat	O43175;B3KSC3;C	56.65	0.0094531	2.0143	1.9994	31.34	4	0.9995671
40S ribosomal protein S9;Ribosomal protein S9;Ribosomal protein S9, isoform CRA_a;cDNA FLJ616	RPS9;hCG_2009111;XXbac-BCX105G6.6-001	40S ribosomal protein S9;Similar to 40S ribosomal prote	P46781;A9C4C1;B	22.591	2.08E-05	2.0912	1.9956	28.729	4	0.9968226
60S ribosomal protein L27a;Ribosomal protein L27a	RPL27A;L27a	60S ribosomal protein L27a	P46776;Q6NZ52;C	16.561	3.15E-06	2.6582	1.9822	20.303	3	0.9871025
cDNA FLJ55484, highly similar to ATP-dependent RNA helicase DDX39 (EC 3.6.1.-);ATP-dependent R	DDX39;DKFZp547B159;hCG_1776331	cDNA FLJ55484, highly similar to ATP-dependent RNA h	B4DX78;O00148;C	53.696	5.61E-26	2.1766	1.9696		1	0.9779027
Zinc finger CCCH domain-containing protein 11A;cDNA FLJ58196, highly similar to Zinc finger CCCH	KIAA0663;ZC3H11A;ZC3HDC11A	Zinc finger CCCH domain-containing protein 11A;ZC3H1	O75152;B4DLG2;C	89.13	3.11E-19	2.554	1.9681	7.9014	7	0.9768035
DNA repair protein XRCC1;X-ray repair cross-complementing protein 1;cDNA, FLJ96373, highly simi	XRCC1	DNA repair protein XRCC1;cDNA FLJ56491, highly simila	P18887;B2RCY5;B	69.476	0.0051545	2.0582	1.9553		1	0.96739
20 kDa CGG-binding protein;CGG triplet repeat-binding protein 1;p20-CGGBP DNA-binding protein;	CGGBP;CGGBP1	CGG triplet repeat-binding protein 1;Uncharacterized pr	Q9UFW8;C9JUJ0	18.82	0.007001	2.3836	1.9349		1	0.952259
60S ribosomal protein L8;cDNA FLJ53750, highly similar to 60S ribosomal protein L8	RPL8	32 kDa protein;60S ribosomal protein L8;26 kDa protein	P62917;B4DVG7	31.75	1.65E-05	2.443	1.9303	65.765	3	0.9488251
Sm protein B/B';Small nuclear ribonucleoprotein-associated proteins B and B';Putative uncharacter	COD;SNRPB;SNRPB1;hCG_2039421;HCERN3	Isoform SM-B1 of Small nuclear ribonucleoprotein-asso	P14678-3;P14678	30.032	0.00020308	2.2389	1.9011	7.9344	7	0.9268344
E3 ubiquitin-protein ligase MIB2;Mind bomb homolog 2;Novel zinc finger protein;Putative NF-kapp	MIB2;SKD;ZZANK1	E3 ubiquitin-protein ligase MIB2 isoform 1;E3 ubiquitin-	Q96AX9-9;Q96AX	115.86	0.0065087	2.1871	1.8857	39.563	3	0.9151002
Functional spliceosome-associated protein 79;hTREX90;NF2/meningioma region protein pK1.3;Plac	C22orf19;KIAA0983;THOC5	THO complex subunit 5 homolog;40 kDa protein;Protein	Q13769;C9JXG5	78.507	0.0016864	2.0989	1.8748	2.6464	2	0.9067367
Cleavage and polyadenylation specificity factor 59 kDa subunit;Cleavage and polyadenylation speci	CPSF7	Isoform 3 of Cleavage and polyadenylation specificity fa	Q8N684-3;Q8N68	56.374	2.46E-13	2.4365	1.8709	10.151	3	0.9037324
Alternative-splicing factor 1;pre-mRNA-splicing factor SF2, P33 subunit;Splicing factor, arginine/seri	ASF;OK/SW-cl.3;SF2;SF2P33;SFRS1	Isoform ASF-1 of Serine/arginine-rich splicing factor 1;Is	Q07955-1;Q07955	27.744	5.27E-14	2.0715	1.8709	23.011	9	0.9037324
Importin subunit beta-1;Importin-90;Karyopherin subunit beta-1;Nuclear factor p97;Pore targeting	KPNB1;NTF97	Importin subunit beta-1	Q14974;B2RBR9;E	97.169	6.40E-12	2.1626	1.861	51.364	9	0.8960781
Arsenite-resistance protein 2;Serrate RNA effector molecule homolog	ARS2;ASR2;SRRT	Isoform 1 of Serrate RNA effector molecule homolog;Isc	Q9BXP5-1;Q9BXP5	100.67	0.00011906	2.2699	1.8588		1	0.8943715
60S ribosomal protein L19;Ribosomal protein L19	RPL19	60S ribosomal protein L19	P84098;Q53G49;C	23.466	2.08E-36	2.3923	1.8538	12.724	3	0.8904856
T-cell activation WD repeat-containing protein;WD repeat-containing protein 36;Putative uncharact	WDR36;DKFZp686I1650	Uncharacterized protein;WD repeat-containing protein	D6RAY5;Q8NI36;C	105.28	0.0015502	1.7963	1.853	23.72	3	0.8898629
Epidermal surface antigen;Flotillin-2;Membrane component chromosome 17 surface marker 1;FLO	ESA1;FLOT2;M17S1;hCG_1998851	53 kDa protein;Flotillin-2;FLOT2 protein	Q14254;Q6FG43;C	53.137	2.21E-22	2.1963	1.8497	14.757	3	0.8872913
ATP-dependent RNA helicase DHX8;DEAH box protein 8;RNA helicase HRH1;cDNA FLJ76879, highly	DDX8;DHX8	ATP-dependent RNA helicase DHX8	Q14562;A8K6J9;B	139.31	0.40917	1.908	1.8413		1	0.8807247
Heterogeneous nuclear ribonucleoprotein F;Heterogeneous nuclear ribonucleoprotein F, N-termina	HNRNPF;HNRPF;OK/SW-cl.23	Heterogeneous nuclear ribonucleoprotein F	P52597;B4DKS8;Q	45.671	1.46E-97	2.3718	1.811	92.803	6	0.8567865
Gamma-1-tubulin;Gamma-tubulin complex component 1;Tubulin gamma-1 chain;Gamma-2-tubulin	TUBG;TUBG1;TUBG2	Tubulin gamma-1 chain;Tubulin gamma-2 chain;34 kDa	P23258;Q9NRH3	51.169	0.0031104	1.8531	1.791		1	0.8407653
Nuclear envelope pore membrane protein POM 210;Nuclear pore membrane glycoprotein 210;Nuc	KIAA0906;NUP210;PSEC0245	Isoform 1 of Nuclear pore membrane glycoprotein 210	Q8TEM1-1;Q8TEM	205.11	1.03E-35	2.2599	1.79	47.416	10	0.8399596
100 kDa DNA-pairing protein;DNA-binding p52/p100 complex, 100 kDa subunit;Polypyrimidine trac	PSF;SFPQ	Isoform Long of Splicing factor, proline- and glutamine-r	P23246-1;P23246	76.149	7.84E-170	2.5104	1.7871	112.45	116	0.8376204
Protein FAM195A	C16orf14;FAM195A	Isoform 1 of Protein FAM195A;Isoform 2 of Protein FAN	Q9BUT9-1;Q9BUT	17.828	0.0085195	1.7775	1.787		1	0.8375396

60S ribosomal protein L6;Neoplasm-related protein C140;Tax-responsive enhancer element-binding	RPL6;TXREB1	DNA-binding protein TAXREB107;60S ribosomal protein	B2R4K7;Q9HBB3;(	32.872	3.55E-09	1.9854	1.7846	18.906	8	0.8356007
Novel nuclear protein 1;Nucleolar protein Nop52;Ribosomal RNA processing protein 1 homolog A;I	D21S2056E;NNP1;NOP52;RRP1;RRP1A	Ribosomal RNA processing protein 1 homolog A;cDNA F	P56182;Q53FR7;C	52.839	9.88E-05	1.8708	1.7773	9.8643	2	0.8296872
Myb-binding protein 1A;cDNA FLJ60422, highly similar to Myb-binding protein 1A;cDNA FLJ57836,	MYBBP1A;P160	Isoform 2 of Myb-binding protein 1A;Isoform 1 of Myb-	Q9BQG0-2;Q9BQ(	149.37	0.001988	2.1051	1.7768	27.423	3	0.8292813
Sm protein E;Small nuclear ribonucleoprotein E;Putative small nuclear ribonucleoprotein polypepti	SNRPE;SNRPEL1	Small nuclear ribonucleoprotein E;Putative small nuclea	P62304;Q5VYJ4;A	10.803	2.81E-10	2.2616	1.7694	12.863	6	0.8232602
Phosphoinositide lipid phosphatase;Protein-tyrosine phosphatase mitochondrial 1;PTEN-like phosp	MOSP;PLIP;PNAS-129;PTPMT1	Isoform 1 of Protein-tyrosine phosphatase mitochondri	Q8WUK0-1;Q8WL	22.843	0.00016931	2.0657	1.7689		1	0.8228525
DEAD box protein 5;Probable ATP-dependent RNA helicase DDX5;RNA helicase p68;cDNA FLJ53366	DDX5;G17P1;HELR;HLR1;DKFZp686J01190	Probable ATP-dependent RNA helicase DDX5;cDNA FLJ5	P17844;B4DN41;E	69.147	3.58E-49	2.5056	1.7615	22.431	10	0.8168045
Intron-binding protein aquarius;Intron-binding protein of 160 kDa;cDNA FLJ75449, highly similar tc	AQR;KIAA0560	Intron-binding protein aquarius	O60306;A8K6F0;B	171.38	0.018615	1.8473	1.7584		1	0.8142633
Leucine-rich repeat-containing protein 47	KIAA1185;LRRC47	Leucine-rich repeat-containing protein 47	Q8N1G4	63.472	1.68E-16	1.9279	1.7542	56.949	2	0.8108132
DEAD box polypeptide 17 isoform p82 variant;DEAD box protein 17;DEAD box protein p72;Probable	DDX17;RP3-434P1.4-009;RP3-434P1.4-005	probable ATP-dependent RNA helicase DDX17 isoform 3	Q59F66;Q92841-4	80.457	1.36E-105	2.5744	1.754	84.063	42	0.8106487
Putative uncharacterized protein CHCHD3;Coiled-coil-helix-coiled-coil-helix domain-containing prot	CHCHD3;hCG_2014841;tcag7.1158	28 kDa protein;Uncharacterized protein;Coiled-coil-heli	C9JRZ6;Q9NX63;A	27.735	0.0030977	1.9305	1.7533		1	0.8100729
Putative uncharacterized protein MATR3;Matrin-3;cDNA FLJ53172, highly similar to Matrin-3;Putat	MATR3;KIAA0723;DKFZp686K23100	Uncharacterized protein;Matrin-3;Uncharacterized prot	A8MXP9;P43243;I	99.966	1.44E-117	2.4205	1.7499	88.471	28	0.8072725
Tubulin beta-2A chain;TUBB2A protein;Tubulin beta-2B chain;TUBB2B protein;Homo sapiens clone	TUBB2;TUBB2A;TUBB2B;DKFZp566F223;OK/	Tubulin beta-2A chain;Tubulin beta-2B chain;cDNA, FLJ5	Q13885;A5D906;/	49.906	4.48E-21	2.1804	1.7433	42.386	11	0.8018209
Acetoacetyl-CoA thiolase;Acetyl-CoA acetyltransferase, mitochondrial;T2;ACAT1 protein	ACAT;ACAT1;MAT	Acetyl-CoA acetyltransferase, mitochondrial;ACAT1 prot	P24752;Q96FG8	45.199	0.00080164	1.4699	1.7313		1	0.7918557
FTP-3;Heterogeneous nuclear ribonucleoprotein H';Heterogeneous nuclear ribonucleoprotein H2;c	FTP3;HNRNPH2;HNRPH2	Heterogeneous nuclear ribonucleoprotein H2;cDNA FLJ	P55795;B4DFK9	49.263	2.21E-116	2.0971	1.7292	24.142	9	0.7901047
Paraspeckle protein 2;RNA-binding motif protein 14;RNA-binding protein 14;RRM-containing coact	RBM14;SIP	Isoform 1 of RNA-binding protein 14;RNA binding motif	Q96PK6-1;Q96PK(	69.491	4.94E-37	2.2599	1.7184	19.648	21	0.7810659
Nucleolar phosphoprotein B23;Nucleolar protein NO38;Nucleophosmin;Numatrin;Truncated nucle	NPM;NPM1	Isoform 1 of Nucleophosmin;Isoform 2 of Nucleophosm	P06748-1;P06748	32.575	5.67E-123	2.134	1.7157	104.89	28	0.7787973
Clathrin heavy chain 1;Clathrin heavy chain on chromosome 17	CLH17;CLTC;CLTCL2;KIAA0034	Isoform 1 of Clathrin heavy chain 1;Isoform 2 of Clathrir	Q00610-1;Q0061(	191.61	7.52E-15	2.123	1.7147	79.603	6	0.7779562
Functional spliceosome-associated protein 35;THO complex subunit 6 homolog;WD repeat-contain	PSEC0006;THOC6;WDR58	Isoform 1 of THO complex subunit 6 homolog;Isoform 3	Q86W42-1;Q86W	37.535	3.21E-48	2.1631	1.7137	111.68	8	0.7771146
Nucleoprotein TPR;TPR protein;Tpr protein;Tpr	TPR;Tpr;tpr	Nucleoprotein TPR	P12270;A0PJC9;Q	267.29	0.0018063	1.7546	1.7117		1	0.7754299
Activating signal cointegrator 1 complex subunit 3-like 1;BRR2 homolog;U5 small nuclear ribonucle	ASCC3L1;HELIC2;KIAA0788;SNRNP200	Isoform 1 of U5 small nuclear ribonucleoprotein 200 kD	O75643-1;O7564	244.5	5.02E-14	1.8746	1.7099	31.759	7	0.773912
Female-lethal(2)D homolog;Pre-mRNA-splicing regulator WTAP;Wilms tumor 1-associating protein;	KIAA0105;WTAP;DKFZp686F20131	Isoform 1 of Pre-mRNA-splicing regulator WTAP	Q15007-1;Q1500	44.243	4.03E-30	2.22	1.7088	103.87	3	0.7729836
PABPC4 protein;Poly(A) binding protein, cytoplasmic 4 (inducible form);Poly(A) binding protein, cyt	hCG_2031827;PABPC4;RP11-69E11.6-002;AI	polyadenylate-binding protein 4 isoform 1;Isoform 1 of	Q4VC03;Q53GL4;(	72.39	1.02E-30	2.0756	1.6992		1	0.7648557
Uncharacterized protein C3orf26;cDNA FLJ42396 fis, clone ASTRO2001107;cDNA FLJ58983;HCG20	C3orf26;hCG_2023567	Uncharacterized protein C3orf26;hypothetical protein L	Q9BQ75;B3KW72;	31.884	0.0087477	1.9236	1.6992		1	0.7648557
Splicing factor 9G8;Splicing factor, arginine/serine-rich 7;Putative uncharacterized protein SFRS7;c	SFRS7	Isoform 1 of Serine/arginine-rich splicing factor 7;Uncha	Q16629-1;Q1662	27.366	1.92E-12	1.8878	1.6891	32.111	4	0.7562547
cDNA FLJ31747 fis, clone NT2RI2007377, highly similar to RNA-BINDING PROTEIN EWS;Ewing sarco	EWSR1;hCG_2010995;EWS;AC002059.7-002	RNA-binding protein EWS isoform 1;Isoform EWS of RN	Q96MX4;Q01844-	68.965	8.43E-75	2.7168	1.678	111.49	11	0.7467427
Symplekin;cDNA FLJ61705, highly similar to Symplekin	SPK;SYMPK	Isoform 1 of Symplekin	Q92797-1;Q9279	141.15	9.79E-07	1.891	1.6766		1	0.7455385
Docking protein alpha;Signal recognition particle receptor subunit alpha;cDNA FLJ53386, highly sin	SRPR	Signal recognition particle receptor subunit alpha;signal	P08240;B4E0H3;C	69.81	1.70E-05	1.8378	1.6751	30.582	2	0.7442472
cDNA FLJ43948 fis, clone TESTI4014924, highly similar to Homo sapiens cytoplasmic FMR1 interact	CYFIP1;KIAA0068;CYFIP2;KIAA1168;PIR121;[	cDNA FLJ43948 fis, clone TESTI4014924, highly similar t	B3KWV6;Q7L576-	145.52	0.0001222	1.5885	1.6673	6.1114	3	0.7375137
Npw38-binding protein;SH3 domain-binding protein SNP70;Splicing factor that interacts with PQBI	NPWBP;SIPP1;SNP70;WBP11	WW domain-binding protein 11;cDNA FLJ60194, highly	Q9Y2W2;B4DMD3	69.997	0.05776	1.6757	1.6584		1	0.729792
Elongation factor Tu, mitochondrial;P43	TUFM	Tu translation elongation factor, mitochondrial precursc	P49411	49.874	7.90E-11	2.0493	1.6436	2.6195	2	0.7168592
DEAD box protein 46;Probable ATP-dependent RNA helicase DDX46;PRP5 homolog;cDNA FLJ78679	DDX46;KIAA0801;hCG_16595	cDNA FLJ78679, highly similar to Homo sapiens DEAD (/	Q7L014;A8K6X3;C	117.47	0.00016125	1.5349	1.6381	34.222	3	0.7120234
54 kDa nuclear RNA- and DNA-binding protein;55 kDa nuclear protein;DNA-binding p52/p100 comp	NONO;NRB54	Non-POU domain-containing octamer-binding protein;n	Q15233;A8K525;E	54.231	1.16E-258	2.5096	1.6368	110.05	166	0.7108781
60S ribosomal protein L3;HIV-1 TAR RNA-binding protein B;Ribosomal protein L3;cDNA FLJ35376 fi	OK/SW-cl.32;RPL3;hCG_2015191;rpl3	60S ribosomal protein L3;60S ribosomal protein L3 isofc	P39023;Q96QL0;E	46.108	3.09E-09	1.9243	1.6358	98.548	5	0.7099964
ATP-dependent RNA helicase eIF4A-1;Eukaryotic initiation factor 4A-I;cDNA FLJ78614, highly simila	DDX2A;EIF4A;EIF4A1;DDX2B;EIF4A2;EIF4F;h	Eukaryotic initiation factor 4A-I;Isoform 2 of Eukaryotic	P60842;A8K088;A	46.153	2.75E-18	2.077	1.6334	29.357	6	0.7078781
Flotillin-1;Flotillin 1 variant;FLOT1 protein;Flotillin 1;Flotillin 1, isoform CRA_b;cDNA FLJ54943, high	FLOT1;DADB-118P11.2-001;DAQB-47P19.1-(	Flotillin-1;cDNA FLJ54943, highly similar to Flotillin-1;Flc	O75955;Q53HQ0;	47.355	1.15E-70	2.0741	1.6328	95.925	8	0.7073481
CF-1 50 kDa subunit;Cleavage stimulation factor 50 kDa subunit;Cleavage stimulation factor subuni	CSTF1;hCG_39097;RP5-1167H4.1-001	Cleavage stimulation factor subunit 1;38 kDa protein	Q05048;B4DDG3;(	48.357	8.04E-15	1.857	1.6316	43.42	4	0.7062874
Lark homolog;RNA-binding motif protein 4;RNA-binding motif protein 4a;RNA-binding protein 4;cD	RBM4;RBM4A;RBM30;RBM4B;RBM14/RBM	Isoform 1 of RNA-binding protein 4;RNA-binding proteir	Q9BWF3-1;Q9BW	40.313	1.08E-62	2.0421	1.6274	16.093	3	0.7025689
U2 small nuclear ribonucleoprotein A';Small nuclear ribonucleoprotein polypeptide A' variant	SNRPA1	U2 small nuclear ribonucleoprotein A'	P09661;Q53G21;C	28.415	2.89E-11	2.0909	1.6204	1.3957	2	0.69635
cDNA FLJ59630, highly similar to Growth hormone-inducible transmembrane protein;GHITM prote	GHITM;DERP2;My021;TMBIM5;UNQ244/PR	cDNA FLJ59630, highly similar to Growth hormone-indu	B4DPG9;Q6FIA7;C	37.535	0.011299	2.0623	1.6197		1	0.6957266
4F2 cell-surface antigen heavy chain;4F2 heavy chain antigen;Lymphocyte activation antigen 4F2 la	MDU1;SLC3A2	Isoform 4 of 4F2 cell-surface antigen heavy chain;4F2 ce	P08195-4;P08195	71.122	5.47E-24	1.8635	1.6048	74.766	8	0.6823935
cDNA FLJ56786, moderately similar to 40S ribosomal protein S16;40S ribosomal protein S16;RPS16	RPS16	cDNA FLJ56786, moderately similar to 40S ribosomal pr	B4DP32;P62249;C	16.412	2.90E-09	2.0324	1.6011	103.89	4	0.6790634
Zinc finger protein 207 variant;Zinc finger protein 207;Putative uncharacterized protein ZNF207;Pu	ZNF207;DKFZp761N202	53 kDa protein;zinc finger protein 207 isoform c;Isoforr	E1P660;Q59G94;C	53.008	2.61E-05	2.0754	1.5974	31.37	3	0.6757256
Nuclear matrix protein 200;Pre-mRNA-processing factor 19;PRP19/PSO4 homolog;Senescence eva	NMP200;PRP19;PRPF19;SNEV	Pre-mRNA-processing factor 19	Q9UMS4	55.18	1.57E-12	2.1149	1.5906	17.693	5	0.6695711
155 kDa nucleoporin;Nuclear pore complex protein Nup155;Nucleoporin Nup155;cDNA FLJ56637,	KIAA0791;NUP155	Isoform 1 of Nuclear pore complex protein Nup155;Isof	O75694-1;O7569	155.2	1.63E-06	1.7551	1.5899	40.916	3	0.668936
GCN1-like protein 1;Translational activator GCN1;cDNA FLJ54481, highly similar to GCN1-like prote	GCN1L1;KIAA0219	Translational activator GCN1	Q92616;B4DM32;	292.75	0.00011067	1.6082	1.5896	22.817	4	0.6686638
93 kDa nucleoporin;Nuclear pore complex protein Nup93;Nucleoporin Nup93;cDNA FLJ78686, higł	KIAA0095;NUP93	Nuclear pore complex protein Nup93;cDNA FLJ58756, h	Q8N1F7;A8K897;E	93.487	8.13E-09	2.0237	1.5877	50.021	2	0.6669383
18S rRNA dimethylase;DIM1 dimethyladenosine transferase 1-like;Probable dimethyladenosine tra	DIMT1;DIMT1L;HUSSY-05	Probable dimethyladenosine transferase;cDNA FLJ5671	Q9UNQ2;A8K9K8;	35.236	0.008216	1.8833	1.5653		1	0.6464392
ATP-dependent RNA helicase DDX48;ATP-dependent RNA helicase eIF4A-3;DEAD box protein 48;Eu	DDX48;EIF4A3;KIAA0111	Eukaryotic initiation factor 4A-III	P38919	46.871	6.57E-18	1.994	1.5646	117.92	5	0.6457939
Nuclear poly(A)-binding protein 1;Poly(A)-binding protein II;Polyadenylate-binding nuclear protein	PAB2;PABP2;PABPN1	Isoform 1 of Polyadenylate-binding protein 2;Isoform 2	Q86U42-1;Q86U4	32.749	3.88E-06	1.8114	1.558	24.451	2	0.6396952
Integral nuclear envelope inner membrane protein;Lamin-B receptor;LMN2R	LBR	Lamin-B receptor	Q14739	70.702	7.24E-28	1.825	1.5571	29.692	6	0.6388616
Phosphate carrier protein, mitochondrial;Phosphate transport protein;Solute carrier family 25 mer	OK/SW-cl.48;PHC;SLC25A3	Isoform A of Phosphate carrier protein, mitochondrial;Is	Q00325-1;Q0032	40.094	1.21E-25	1.9453	1.554	32.606	8	0.6359865
Cytoplasmic dynein 1 intermediate chain 2;Cytoplasmic dynein intermediate chain 2;Dynein interr	DNCI2;DNCIC2;DYNC1I2	Isoform 2A of Cytoplasmic dynein 1 intermediate chain	Q13409-1;Q1340	71.456	4.21E-12	1.842	1.5489		1	0.631244
G(i) alpha-3;Guanine nucleotide-binding protein G(k) subunit alpha;cDNA, FLJ92547, Homo sapiens	GNAI3;hCG_1997731;RP5-1160K1.2-001	Guanine nucleotide-binding protein G(k) subunit alpha	P08754;Q5TZX1	40.532	2.24E-05	1.9237	1.5386		1	0.6216182
Pre-mRNA-splicing factor SRp30C;Splicing factor, arginine/serine-rich 9;cDNA FLJ76387, highly simi	SFRS9;SRP30C	Serine/arginine-rich splicing factor 9;cDNA FLJ56571, hi	Q13242;A8K3M9;	25.542	2.80E-08	1.9758	1.5294	9.5941	2	0.6129658
Dolichyl-diphosphooligosaccharide--protein glycosyltransferase 67 kDa subunit;Dolichyl-diphospho	RPN1	Dolichyl-diphosphooligosaccharide--protein glycosyltrar	P04843;Q53EP4;C	72.777	4.45E-21	1.6892	1.5281	25.3	6	0.611739
Cutaneous T-cell lymphoma-associated antigen se33-1;Nuclear protein 220;Zinc finger matrin-like	NP220;ZFML;ZNF638	Isoform 1 of Zinc finger protein 638;Isoform 3 of Zinc fir	Q14966-1;Q1496	220.62	5.37E-06	1.9673	1.5179	57.412	2	0.6020767
Cellular apoptosis susceptibility protein;Chromosome segregation 1-like protein;Exportin-2;Importi	CAS;CSE1L;XPO2;CSE1L-2	Isoform 1 of Exportin-2;Isoform 3 of Exportin-2;cDNA FL	P55060-1;P55060	110.42	0.0021881	1.6525	1.5123	176.82	4	0.5967444
DEAD box protein 47;Probable ATP-dependent RNA helicase DDX47;DEAD (Asp-Glu-Ala-Asp) box pc	DDX47;hCG_27698;MST162;E4-DBP	Probable ATP-dependent RNA helicase DDX47;probable	Q9H0S4;Q53GJ1;C	50.646	7.83E-16	1.3476	1.5063	14.714	9	0.5910091
Vimentin;Vimentin variant 3;Vimentin variant 4;cDNA FLJ34494 fis, clone HLUNG2005030, highly si	VIM	Vimentin	P08670;B0YJC4;B(	53.651	4.57E-105	1.8797	1.5048	87.327	26	0.5895718
60S ribosomal protein L7;Similar to 60S ribosomal protein L7; similar to P18124 (PID:d133021);Puta	RPL7;WUGSC:H_RG054D04.1	32 kDa protein;30 kDa protein;60S ribosomal protein L7	P18124;O95036;A	32.275	1.30E-16	1.7368	1.489	112.58	6	0.5743438
U1 small nuclear ribonucleoprotein C	RP3-375P9.1-002;SNRPC;hCG_16077;RP3-37	U1 small nuclear ribonucleoprotein C;U1 small nuclear	Q53G33;Q5TAL3;F	19.687	1.32E-11	1.9398	1.4886		1	0.5739561
Cytoplasmic dynein 1 heavy chain 1;Cytoplasmic dynein heavy chain 1;Dynein heavy chain, cytosol	DHC1;DNCH1;DNCL;DNECL;DYHC;DYNC1H1;	Cytoplasmic dynein 1 heavy chain 1	Q14204;B4DSR6;(	532.4	6.90E-23	2.0465	1.487	20.512	5	0.5724046
5'-3' exoribonuclease 2;DHM1-like protein;cDNA FLJ10711 fis, clone NT2RP3000917, highly similar	XRN2	Isoform 1 of 5'-3' exoribonuclease 2;Isoform 2 of 5'-3' e	Q9H0D6-1;Q9H0[	108.58	3.42E-07	1.5978	1.4867	10.692	5	0.5721136
116 kDa U5 small nuclear ribonucleoprotein component;Elongation factor Tu GTP-binding domain-	EFTUD2;KIAA0031;SNRP116;U5-116KD	116 kDa U5 small nuclear ribonucleoprotein componen	Q15029;A8KAP3;E	109.43	1.13E-19	1.8424	1.4848	62.925	8	0.5702686
CD2 antigen cytoplasmic tail-binding protein 2;FWP010	CD2BP2;KIAA1178	CD2 antigen cytoplasmic tail-binding protein 2	O95400;Q5QTR6	37.646	0.0060661	1.7798	1.482		1	0.5675454
Heterogeneous nuclear ribonucleoprotein H;Heterogeneous nuclear ribonucleoprotein H, N-termir	HNRNPH1;HNRPH;HNRPH1;DKFZp686A1517	51 kDa protein;Heterogeneous nuclear ribonucleoprote	P31943;B4DP51;C	51.229	1.85E-182	2.1446	1.4729	95.173	46	0.5586595
Adenovirus early region 1B-associated protein 5;E1B-55 kDa-associated protein 5;Heterogeneous r	E1BAP5;HNRNPUL1;HNRPUL1	Isoform 1 of Heterogeneous nuclear ribonucleoprotein	Q9BUJ2-1;Q9BUJ2	95.737	8.83E-51	1.8814	1.4721	95.514	13	0.5578757
75 kDa DNA-pairing protein;Oncogene FUS;Oncogene TLS;POMp75;RNA-binding protein FUS;Trans	FUS;TLS	Fus-like protein (Fragment);Isoform Short of RNA-bindir	P35637-1;P35637	53.376	2.63E-44	1.8649	1.4705	99.61	17	0.5563068
ATP-dependent RNA helicase DDX3X;DEAD box protein 3, X-chromosomal;DEAD box, X isoform;Hel	DBX;DDX3;DDX3X;DBY;DDX3Y	ATP-dependent RNA helicase DDX3X;cDNA FLJ60675, hi	O00571;B4E3E8;B	73.243	4.52E-40	1.7795	1.4484	111.11	18	0.5344601
Activator 1 40 kDa subunit;Activator 1 subunit 2;Replication factor C 40 kDa subunit;Replication fac	RFC2;hCG_18034	Isoform 1 of Replication factor C subunit 2;Isoform 2 of	P35250-1;P35250	39.157	0.00059871	1.6179	1.4477	17.699	2	0.5337627
160 kDa neurofilament protein;Neurofilament 3;Neurofilament medium polypeptide;Neurofilamer	NEF3;NEFM;NFM	Neurofilament medium polypeptide;NEFM protein;cDN	P07197;B4DGN2;/	102.47	0.014178	1.9324	1.4465		1	0.5325663
DNA mismatch repair protein Msh2;MutS protein homolog 2;cDNA FLJ57316, highly similar to DNA	MSH2	DNA mismatch repair protein Msh2;MutS homolog 2 va	P43246;B4DL39;B	104.74	0.0011005	1.3117	1.4435	4.7158	2	0.5295711
Kinesin-like DNA-binding protein;Kinesin-like protein 4;Kinesin-like protein KIF22;cDNA FLJ53835, h	KID;KIF22;KNSL4;hCG_2039829;A-328A3.5;C	Kinesin-like protein KIF22	Q14807;B7Z265;B	73.261	0.00067663	1.7699	1.4421		1	0.5281712
Heterogeneous nuclear ribonucleoprotein U-like protein 2;Scaffold-attachment factor A2	HNRNPUL2;HNRPUL2	Heterogeneous nuclear ribonucleoprotein U-like protei	Q1KMD3	85.104	6.62E-14	1.5466	1.4338	49.874	3	0.5198438
Polyadenylate-binding protein 1;cDNA FLJ57246, highly similar to Poly(A)-binding protein 1;cDNA F	PAB1;PABP1;PABPC1;PABPC2;PABP3;PABPC3	Isoform 1 of Polyadenylate-binding protein 1;cDNA FLJ3	P11940-1;P11940	70.67	1.66E-30	1.8059	1.4314	40.283	3	0.5174269

214 kDa nucleoporin;Nuclear pore complex protein Nup214;Nucleoporin Nup214;Protein CAN;cDN	CAIN;CAN;KIAA0023;NUP214;RP11-544A12.	Isoform 5 of Nuclear pore complex protein Nup214;Isof	P35658-5;P35658	215.4	1.27E-06	1.6152	1.4312	68.791	3	0.5172253
Dolichyl-diphosphooligosaccharide--protein glycosyltransferase 63 kDa subunit;Dolichyl-diphospho	RPN2;hCG_37142;RP3-343K2.2-003;RP3-343	Dolichyl-diphosphooligosaccharide--protein glycosyltrar	P04844;B2RE46;Q	69.283	5.10E-07	1.4623	1.4292	22.593	2	0.5152078
Sm protein F;Small nuclear ribonucleoprotein F	PBSCF;SNRPF	Small nuclear ribonucleoprotein F	P62306	9.7251	0.0022352	1.6507	1.4282		1	0.514198
Heterogeneous nuclear ribonucleoprotein 2H9;Heterogeneous nuclear ribonucleoprotein H3;Hetei	HNRNPH3;HNRPH3	Isoform 1 of Heterogeneous nuclear ribonucleoprotein	P31942-1;P31942	36.926	9.13E-29	1.8634	1.4251	18.213	12	0.5110632
Sex comb on midleg-like protein 2;cDNA FLJ54781, highly similar to Sex comb on midleg-like protei	SCML2;RP5-1129A6.3-002	Isoform 1 of Sex comb on midleg-like protein 2;cDNA FL	Q9UQR0-1;Q9UQ	77.256	0.0044503	1.6925	1.413		1	0.4987615
Pre-mRNA-splicing factor SF3b 155 kDa subunit;Spliceosome-associated protein 155;Splicing factor	SAP155;SF3B1;DKFZp434M052	Splicing factor 3B subunit 1	O75533;A0JLT9;A(	145.83	5.01E-13	2.1988	1.4129	73.047	9	0.4986594
Mannosyl-oligosaccharide glucosidase;Processing A-glucosidase I;Glucosidase I;cDNA FLJ75504, hig	GCS1;MOGS	Mannosyl-oligosaccharide glucosidase;mannosyl-oligos	Q13724;Q58F09;A	91.916	1.29E-05	1.3395	1.3993		1	0.4847053
Serine/threonine-protein phosphatase PP1-beta catalytic subunit;Serine/threonine-protein phosph	PPP1CB	Serine/threonine-protein phosphatase PP1-beta catalyt	P62140;B4DJ75;B	37.186	7.83E-23	1.7782	1.3956		1	0.4808855
Transmembrane and coiled-coil domain-containing protein 1;Transmembrane and coiled-coil doma	PNAS-10;PNAS-136;TMCC4;TMCO1;UNQ151	Isoform 1 of Transmembrane and coiled-coil domain-co	Q9UM00-1;Q9UM	21.175	1.85E-08	1.7475	1.3943		1	0.479541
Lamin-B1;cDNA FLJ50934, highly similar to Lamin-B1;LMNB1 protein	LMN2;LMNB;LMNB1	Lamin-B1;LMNB1 protein	P20700;B4DZT3;Q	66.408	4.07E-28	1.8999	1.3931	73.648	13	0.4782988
Heterogeneous nuclear ribonucleoproteins C1/C2;cDNA FLJ75154, highly similar to Homo sapiens I	HNRNPC;HNRPC;hCG_1641229	Isoform C2 of Heterogeneous nuclear ribonucleoprotei	P07910-1;P07910	33.67	8.97E-46	1.7235	1.3918	108.44	17	0.4769519
Pre-mRNA-splicing factor SRP75;Splicing factor, arginine/serine-rich 4;SRP001LB;cDNA FLJ76859, hi	SFRS4;SRP75;hCG_23483;SFRS6;SRP55;HRS;	Serine/arginine-rich splicing factor 4;Isoform SRP55-1 o	Q08170;A8K644;C	56.678	0.0029898	1.7655	1.3906	6.8785	3	0.4757075
ATP synthase subunit alpha, mitochondrial;ATP synthase subunit alpha;cDNA FLJ54625, highly simi	ATP5A;ATP5A1;ATP5AL2;ATPM	ATP synthase subunit alpha, mitochondrial;cDNA FLJ541	P25705;A8K092;B	59.75	3.22E-20	1.6426	1.3879	21.678	5	0.4729036
Splicing factor U2AF 35 kDa subunit;U2 auxiliary factor 35 kDa subunit;U2 snRNP auxiliary factor sr	U2AF1;U2AF35;U2AFBP;DKFZp313J1712;hC(	Splicing factor U2AF 35 kDa subunit;27 kDa protein;spli	Q01081;B5BU08;(	27.872	4.57E-35	1.5846	1.3775	24.523	6	0.4620523
78 kDa gastrin-binding protein;Long chain 3-hydroxyacyl-CoA dehydrogenase;Long-chain enoyl-Co/	HADH;HADHA	Trifunctional enzyme subunit alpha, mitochondrial;cDN.	P40939;B4DDZ5;B	82.999	0.00066885	1.5632	1.375		1	0.4594316
Lamina-associated polypeptide 2, isoforms beta/gamma;Splenin;Thymopentin;Thymopoietin;Thym	LAP2;TMPO	Isoform Beta of Lamina-associated polypeptide 2, isofor	P42167-1;P42167	50.67	1.30E-163	1.8283	1.3748	66.687	36	0.4592218
Signal sequence receptor subunit delta;Translocon-associated protein subunit delta;Putative uncha	SSR4;TRAPD	Translocon-associated protein subunit delta precursor;l	P51571;A6NLM8	20.213	1.53E-09	1.4751	1.3705	16.733	4	0.4547023
40S ribosomal protein S14	PRO2640;RPS14	40S ribosomal protein S14	P62263;E5RH77	16.273	0.00028311	1.762	1.3684		1	0.45249
cDNA FLJ59809, highly similar to Bone marrow stromal antigen 2;Bone marrow stromal antigen 2;I	BST2	cDNA FLJ59809, highly similar to Bone marrow stromal	B4E0Z6;Q10589	23.537	0.00022268	1.7413	1.3616	6.471	2	0.4453029
60S ribosomal protein L13;Breast basic conserved protein 1;Ribosomal protein L13	BBC1;OK/SW-cl.46;RPL13;hCG_1723872	60S ribosomal protein L13;24 kDa protein	P26373;A8K4C8;B	24.261	9.08E-16	1.7104	1.3508	69.138	5	0.4338141
Complex I-PDSW;NADH dehydrogenase [ubiquinone] 1 beta subcomplex subunit 10;NADH-ubiquin	NDUFB10;hCG_42700	NADH dehydrogenase [ubiquinone] 1 beta subcomplex	O96000;A8K761	20.776	0.009632	1.6489	1.3471		1	0.429857
Heat shock 70 kDa protein 1/2;Heat shock 70 kDa protein 1A/1B;cDNA FLJ75127, highly similar to I	HSPA1;HSPA1A;HSPA1B;DAAP-21F2.7-001;D.	Heat shock 70 kDa protein 1A/1B;cDNA FLJ54392, highl	P08107;A8K5I0;B4	70.051	1.53E-27	1.7395	1.3371	2.452	4	0.4191074
136 kDa double-stranded RNA-binding protein;Double-stranded RNA-specific adenosine deaminas	ADAR;ADAR1;DSRAD;G1P1;IFI4;hCG_18813	Isoform 4 of Double-stranded RNA-specific adenosine d	P55265-4;P55265	140.84	2.42E-41	2.1523	1.3361		1	0.418028
Basement membrane-associated chondroitin proteoglycan;Chondroitin sulfate proteoglycan 6;Chr	BAM;BMH;CSPG6;SMC3;SMC3L1	Structural maintenance of chromosomes protein 3	Q9UQE7;B0AZQ4;	141.54	0.043309	1.4384	1.3349	12.142	2	0.4167317
ATP-dependent helicase SKIV2L2;Superkiller viralicidic activity 2-like 2;cDNA FLJ76877, highly simila	KIAA0052;SKIV2L2	Superkiller viralicidic activity 2-like 2	P42285;A8K6I4;B4	117.8	3.67E-05	1.3961	1.3306	47.751	3	0.4120769
B-cell receptor-associated protein 31, isoform CRA_b;cDNA FLJ33051 fis, clone TRACH1000063, hig	BCAP31;hCG_2004982;BAP31;DXS1357E	B-cell receptor-associated protein 31 isoform a;B-cell re	B3KQ79;C9J0M4;(	34.752	0.14934	1.5605	1.3301		1	0.4115347
Cell proliferation-inducing gene 46 protein;Cytokeratin-18;Keratin, type I cytoskeletal 18;Keratin-18	CYK18;KRT18;PIG46	Keratin, type I cytoskeletal 18;18 kDa protein	P05783;B2RA03	48.057	4.95E-86	1.6096	1.3276	50.843	38	0.4088205
40S ribosomal protein S4, X isoform;SCR10;Single copy abundant mRNA protein;cDNA FLJ40595 fis	CCG2;RPS4;RPS4X;SCAR;hCG_18634;RPS4Y2	40S ribosomal protein S4, X isoform;40S ribosomal prot	P62701;B2R491;Q	29.597	0.00010633	1.7119	1.3214	51.277	4	0.4020672
CD44 antigen;CDw44;Epican;Extracellular matrix receptor III;GP90 lymphocyte homing/adhesion r	CD44;LHR;MDU2;MDU3;MIC4;AL133330.1-(	Isoform 1 of CD44 antigen;Isoform 5 of CD44 antigen;Is	P16070-1;P16070	81.537	3.26E-14	1.6842	1.3202	14.33	3	0.4007565
IGF-II mRNA-binding protein 3;Insulin-like growth factor 2 mRNA-binding protein 3;KH domain-con	IGF2BP3;IMP3;KOC1;VICKZ3	Isoform 1 of Insulin-like growth factor 2 mRNA-binding	O00425-1;O00425	63.704	5.53E-21	1.5343	1.3167	131.29	8	0.3969267
Chromokinesin-B;Chromosome-associated kinesin KIF4B;cDNA FLJ61467, highly similar to Chromos	KIF4B;KIF4;KIF4A	Chromosome-associated kinesin KIF4B;Isoform 1 of Chr	Q2VIQ3;B4DYE2;C	140.03	8.74E-06	1.3168	1.3158		1	0.3959402
38 kDa nuclear protein containing a WW domain;Polyglutamine tract-binding protein 1;Polyglutam	JM26;NPW38;PQBP1	Isoform 1 of Polyglutamine-binding protein 1;Isoform 2	O60828-1;O6082	30.472	0.012481	1.5014	1.3049	42.577	2	0.3839393
Sucrose nonfermenting protein 2 homolog;SWI/SNF-related matrix-associated actin-dependent reg	SMARCA5;SNF2H;WCRF135;SMARCA1;SNF2	SWI/SNF-related matrix-associated actin-dependent reg	O60264;B4DZC0;E	121.9	2.99E-09	1.5516	1.3024	43.82	7	0.3811726
hTREX120;THO complex subunit 2;cDNA FLJ61096, highly similar to THO complex subunit 2;THO cc	CXorf3;THOC2;RP5-1189B24.5-003	Isoform 1 of THO complex subunit 2;THO complex subu	Q8NI27-1;Q8NI27	182.77	3.10E-25	1.8292	1.2994	127.79	5	0.3778456
Aldehyde dehydrogenase 10;Aldehyde dehydrogenase family 3 member A2;Fatty aldehyde dehydr	ALDH10;ALDH3A2;FALDH;DKFZp686E23276;	Isoform 2 of Fatty aldehyde dehydrogenase;Isoform 1 o	P51648-2;P51648	57.669	0.020632	1.4499	1.2922		1	0.3698294
Putative uncharacterized protein SPTAN1;Alpha-II spectrin;Fodrin alpha chain;Spectrin alpha chain,	SPTAN1;SPTA2;DKFZp564P0562	Uncharacterized protein;Isoform 2 of Spectrin alpha cha	A6NG51;Q13813-	284.94	2.52E-07	1.2884	1.2796	2.8414	2	0.3556929
107 kDa nucleoporin;Nuclear pore complex protein Nup107;Nucleoporin Nup107;cDNA FLJ11217 f	NUP107	Nuclear pore complex protein Nup107;NUP107 protein	P57740;B3KMK0;I	106.37	0.0037	1.4215	1.2763		1	0.3519675
96 kDa nucleoporin;98 kDa nucleoporin;Nuclear pore complex protein Nup96;Nuclear pore comple	ADAR2;NUP98	Isoform 1 of Nuclear pore complex protein Nup98-Nup9	P52948-1;P52948	197.58	3.06E-22	1.6034	1.2762	68.2	3	0.3518544
56 kDa U2AF65-associated protein;ATP-dependent RNA helicase p47;DEAD box protein UAP56;HLA	BAT1;UAP56;XXbac-BCX270M2.2-006;DADB-	Isoform 2 of Spliceosome RNA helicase BAT1;Isoform 1	Q13838-2;Q1383	50.679	2.30E-39	1.8752	1.2752	65.671	20	0.3507235
40S ribosomal protein S18;Ke-3;Ribosomal protein S18;Ribosomal protein S18, isoform CRA_b	D6S218E;RPS18;DADB-159G18.1-001;DAMC	40S ribosomal protein S18;18 kDa protein;17 kDa prote	P62269;Q5SUJ3	17.718	2.38E-05	1.3552	1.2674	29.101	4	0.3418719
Putative uncharacterized protein DBN1;Developmentally-regulated brain protein;Drebrin;cDNA FLJ	DBN1;D0S117E;hCG_2039393	Uncharacterized protein;Isoform 2 of Drebrin;Isoform 1	A8MV58;Q16643-	76.299	9.23E-06	1.5626	1.2658		1	0.3400495
Heat shock 70 kDa protein 6;Heat shock 70 kDa protein B';cDNA, FLJ93166, highly similar to Homo	HSP70B';HSPA6;HSP70B;HSPA7;HSPA1B	Heat shock 70 kDa protein 6;Putative heat shock 70 kDa	P17066;B2R6X5;B	71.027	8.11E-30	1.6735	1.2656		1	0.3398215
DNA ligase 3;DNA ligase III;Polydeoxyribonucleotide synthase [ATP] 3;DNA ligase;Ligase III, DNA, AT	LIG3	Isoform Alpha of DNA ligase 3;Isoform Beta of DNA ligas	P49916-1;P49916	112.91	2.11E-08	1.2747	1.2636	53.995	3	0.3375398
Double-stranded RNA-binding protein 76;Interleukin enhancer-binding factor 3;M-phase phosphop	DRBF;ILF3;MPHOSPH4;NF90	interleukin enhancer-binding factor 3 isoform d;Isoform	Q12906-4;Q1290	95.807	9.66E-61	1.7135	1.2549	86.728	22	0.3275724
Isoleucine--tRNA ligase;Isoleucyl-tRNA synthetase, cytoplasmic;cDNA FLJ55373, highly similar to Is	IARS;DKFZp686L17145;DKFZp686L0869;RP1	Isoleucyl-tRNA synthetase, cytoplasmic;IARS protein	P41252;B4DVX2;C	144.5	0.0063994	1.3469	1.2529		1	0.3252713
Heterogeneous nuclear ribonucleoprotein A3;cDNA FLJ52659, highly similar to Heterogeneous nuc	HNRNPA3;HNRPA3;hCG_2005824	Isoform 1 of Heterogeneous nuclear ribonucleoprotein	P51991-1;P51991	39.594	7.90E-53	1.7326	1.2405	35.122	12	0.3109217
Mitsugumin-23;Transmembrane protein 109;cDNA, FLJ94551;cDNA FLJ57860, highly similar to Trar	TMEM109	Transmembrane protein 109;cDNA FLJ57860, highly sim	Q9BVC6;B2R9T9;E	26.21	1.80E-11	1.642	1.233		1	0.3021728
205 kDa nucleoporin;Nuclear pore complex protein Nup205;Nucleoporin Nup205;cDNA FLJ50078,	C7orf14;KIAA0225;NUP205	Nuclear pore complex protein Nup205;176 kDa protein;	Q92621;B4DE72;C	227.92	6.15E-06	1.2086	1.2314		1	0.3002995
Pre-mRNA-splicing factor SRP20;Splicing factor, arginine/serine-rich 3;cDNA, FLJ92926, Homo sapie	SFRS3;SRP20;hCG_14802	Serine/arginine-rich splicing factor 3;cDNA FLJ52832, hi	P84103;B2R6F3;B	19.329	5.14E-17	1.582	1.2271	23.899	10	0.2952528
Leucine-rich repeat-containing protein 59;cDNA, FLJ95955	LRRC59;PRO1855	Leucine-rich repeat-containing protein 59;Uncharacteri	Q96AG4;B2RE83;I	34.93	5.74E-11	1.5766	1.2238	129.19	6	0.2913678
Basic leucine zipper and W2 domain-containing protein 2;cDNA FLJ10398 fis, clone NT2RM400034	BZW2;HSPC028;MSTP017;hCG_19293	Basic leucine zipper and W2 domain-containing protein	Q9Y6E2;B3KM68;I	48.162	0.005815	1.3288	1.2236		1	0.291132
Chromobox protein homolog 8;Polycomb 3 homolog;Rectachrome 1;Putative uncharacterized prot	CBX8;PC3;RC1	Chromobox protein homolog 8;Uncharacterized protein	Q9HC52;C9JM54;(	43.395	0.0033459	1.4639	1.2184	127.07	2	0.2849878
Heterogeneous nuclear ribonucleoprotein K;Transformation up-regulated nuclear protein;cDNA FL	HNRNPK;HNRPK;hCG_1985922;RP11-575L7.	Isoform 2 of Heterogeneous nuclear ribonucleoprotein	P61978-2;P61978	51.028	9.11E-273	1.5037	1.2071	117.96	82	0.2715452
mRNA export factor;mRNA-associated protein mrnp 41;Rae1 protein homolog;cDNA FLJ75499, hig	MRNP41;RAE1;hCG_2018215;RP4-800J21.2-	mRNA export factor	P78406;A8K882;Q	40.968	0.050575	1.479	1.2065		1	0.2708279
GAP-associated tyrosine phosphoprotein p62;KH domain-containing, RNA-binding, signal transduct	KHDRBS1;SAM68;hCG_41606	Isoform 1 of KH domain-containing, RNA-binding, signal	Q07666-1;Q0766	48.227	1.26E-14	1.6143	1.2031	74.997	16	0.2667566
MORF-related gene X protein;Mortality factor 4-like protein 2;Protein MSL3-2;Transcription factor-l	KIAA0026;MORF4L2;MRGX;RP5-1055C14.2-(	Mortality factor 4-like protein 2;Mortality factor 4 like 2	Q15014;B3KWX6;	32.307	0.0061761	1.2321	1.1999		1	0.2629142
Foocen;Neurite outgrowth inhibitor;Neuroendocrine-specific protein;Neuroendocrine-specific prot	KIAA0886;My043;NOGO;RTN4;SP1507;hCG_	Isoform 1 of Reticulon-4;Isoform 5 of Reticulon-4;Isofor	Q9NQC3-1;Q9NQ	129.93	0.0075044	1.4804	1.1991		1	0.261952
91 kDa synaptosomal-associated protein;Clathrin coat assembly protein AP180;Clathrin coat-associ	KIAA0656;SNAP91;PICALM;PICALM variant	Isoform 1 of Clathrin coat assembly protein AP180;cDN/	O60641-1;O60641	92.501	0.012683	1.274	1.1959		1	0.2580968
AFG3-like protein 2;Paraplegin-like protein;AFG3 ATPase family gene 3-like 2 (Yeast), isoform CRA_	AFG3L2;hCG_38275	AFG3-like protein 2	Q9Y4W6;D3DUJ0;	88.583	0.0081591	1.3403	1.1932		1	0.2548359
CapZ alpha-2;F-actin-capping protein subunit alpha-2;Capping protein (Actin filament) muscle Z-lin	CAPZA2;hCG_37884;tcag7.31;CAPZA1	F-actin-capping protein subunit alpha-2;F-actin-capping	P47755;A4D0V4;C	32.949	0.00051536	1.5132	1.1929		1	0.2544731
Adenine nucleotide translocator 2;ADP,ATP carrier protein 2;ADP,ATP carrier protein, fibroblast isof	ANT2;SLC25A5;ANT3;CDABP0051;SLC25A6;I	ADP/ATP translocase 2;ADP/ATP translocase 3;ADP/ATP	P05141;B2RCV1;C	32.852	2.62E-20	1.4665	1.1918	98.726	11	0.2531422
Cytokine-induced protein of 29 kDa;Nuclear protein Hcc-1;Proliferation-associated cytokine-inducil	HCC1;HSPC316;SARNP;CIP29;LOC402290;tc	52 kDa protein;SAP domain-containing ribonucleoprote	P82979;Q567R9;A	51.782	6.31E-11	1.474	1.1907	25.472	6	0.25181
Vasodilator-stimulated phosphoprotein	VASP	Vasodilator-stimulated phosphoprotein	P50552	39.829	9.89E-09	1.3008	1.1882	18.731	2	0.2487777
Protein APMCF1;Signal recognition particle receptor subunit beta;Signal recognition particle recept	PSEC0230;SRPRB;hCG_2023561	Signal recognition particle receptor subunit beta;Uncha	Q9Y5M8;Q549N5;	29.702	4.16E-31	1.5373	1.1843	1.0967	2	0.2440346
Protein phosphatase 1C catalytic subunit;Serine/threonine-protein phosphatase PP1-gamma cataly	PPP1CC;PPP1A;PPP1CA	Isoform Gamma-2 of Serine/threonine-protein phospha	P36873-2;P36873	38.518	2.76E-33	1.4564	1.1824	85.639	4	0.2417182
Lamina-associated polypeptide 2, isoform alpha;Splenin;Thymopentin;Thymopoietin;Thymopoietir	LAP2;TMPO	Lamina-associated polypeptide 2, isoform alpha	P42166	75.491	1.14E-127	1.4268	1.1778	19.957	3	0.2360946
Zinc finger protein 91 homolog;cDNA FLJ57065, highly similar to Zinc finger protein 91	FKSG11;ZFP91	Isoform 1 of E3 ubiquitin-protein ligase ZFP91;Isoform 2	Q96JP5-1;Q96JP5;	63.444	0.0015587	1.1947	1.1735	32.364	2	0.2308178
Pre-mRNA-splicing factor SF3b 130 kDa subunit;Spliceosome-associated protein 130;Splicing factor	KIAA0017;SAP130;SF3B3	Isoform 1 of Splicing factor 3B subunit 3	Q15393-1;Q15393	135.58	8.04E-23	1.3965	1.1618	21.25	4	0.2163617
Cullin-associated and neddylation-dissociated protein 1;Cullin-associated NEDD8-dissociated prote	CAND1;KIAA0829;TIP120;TIP120A	Isoform 1 of Cullin-associated NEDD8-dissociated protei	Q86VP6-1;Q86VP(	136.37	2.43E-09	1.1163	1.1538	45.859	4	0.2063932
40S ribosomal protein S3;Ribosomal protein S3 variant	OK/SW-cl.26;RPS3	28 kDa protein;40S ribosomal protein S3	P23396;Q53G83	28.486	6.24E-36	1.5027	1.1529	61.225	7	0.2052674
Heat shock 84 kDa;Heat shock protein HSP 90-beta;cDNA FLJ77842;cDNA FLJ53619, highly similar t	HSP90AB1;HSP90B;HSPC2;HSPCB	Heat shock protein HSP 90-beta	P08238;A8K3W9;I	83.263	1.60E-14	1.3354	1.1508	91.013	6	0.2026371
CCAAT displacement protein;Homeobox protein cut-like 1;Homeobox protein cux-1;Protein CASP;c	CUTL1;CUX1;Nbla10317	Isoform 3 of Homeobox protein cut-like 1;Isoform 1 of I	P39880-3;P39880	165.69	8.59E-06	1.2275	1.1417	7.2062	2	0.1911836
Actinin alpha 1 isoform 3;ACTN1 protein;cDNA FLJ59209, highly similar to Alpha-actinin-1;Actinin a	ACTN1;ACTN4;hCG_22243	Actinin alpha 1 isoform 3;alpha-actinin-1 isoform a;Alpl	B7TY16;A1L0V1;B	107.14	2.84E-05	1.0655	1.1406	11.095	3	0.1897929
Heat shock 70 kDa protein 8;Heat shock cognate 71 kDa protein;cDNA FLJ77848;cDNA FLJ38781 fis	HSC70;HSP73;HSPA10;HSPA8;HSPA2	Isoform 1 of Heat shock cognate 71 kDa protein;54 kDa	P11142-1;P11142	70.897	3.35E-69	1.4101	1.138	87.574	10	0.1865006
17-beta-hydroxysteroid dehydrogenase 10;3-hydroxy-2-methylbutyryl-CoA dehydrogenase;3-hydro	ERAB;HADH2;HSD17B10;MRPP2;SCHAD;XH	Isoform 1 of 3-hydroxyacyl-CoA dehydrogenase type-2;I	Q99714-1;Q99714	26.923	5.26E-08	1.3928	1.1342		1	0.1816751

ATP-binding cassette sub-family G member 2;Breast cancer resistance protein;CDw338;Mitoxantro	ABCG2;ABCP;BCRP;BCRP1;MXR	Isoform 1 of ATP-binding cassette sub-family G member	Q9UNQ0-1;Q9UN	72.313	2.18E-05	1.1761	1.1297		1	0.1759397
Elongation factor 2;cDNA FLJ60696, highly similar to Elongation factor 2;cDNA FLJ58164, highly sim	EEF2;EF2	Elongation factor 2;cDNA FLJ56548, highly similar to Elo	P13639;B4DMC6;	95.337	5.53E-05	1.4082	1.1279	34.965	3	0.1736392
TAR DNA-binding protein 43;cDNA FLJ57180, highly similar to TAR DNA-binding protein 43;cDNA FL	TARDBP;TDP43;RP4-635E18.2-003	Isoform 2 of TAR DNA-binding protein 43;Isoform 1 of T	Q13148-2;Q1314	44.991	3.76E-51	1.7138	1.1231	96.85	6	0.1674864
B-cell receptor-associated protein BAP37;D-prohibitin;Prohibitin-2;Repressor of estrogen receptor	BAP;PHB2;REA	Prohibitin-2;cDNA FLJ56579, highly similar to Prohibitin	Q99623;Q9BXV3;	33.296	3.80E-106	1.4306	1.1217	77.711	12	0.1656869
Pre-mRNA-splicing factor SF3b 145 kDa subunit;SF3b150;Spliceosome-associated protein 145;Splic	SAP145;SF3B2;DKFZp781L0540	Splicing factor 3B subunit 2;Putative uncharacterized pr	Q13435;Q7Z3K9	100.23	8.43E-06	1.7158	1.1202		1	0.1637563
Putative uncharacterized protein ENSP00000346598;37 kDa laminin receptor precursor;37/67 kDa	LAMBR;LAMR1;RPSA;LAMR1P15	33 kDa protein;Uncharacterized protein;40S ribosomal	A6NE09;P08865;C	33.313	9.56E-06	1.3649	1.12	14.415	2	0.1634987
Tyrosine--tRNA ligase;Tyrosyl-tRNA synthetase, mitochondrial	CGI-04;YARS2	Tyrosyl-tRNA synthetase, mitochondrial	Q9Y2Z4	53.198	0.00042371	1.0583	1.1173	52.007	2	0.1600166
Annexin A2;Annexin II;Annexin-2;Calpactin I heavy chain;Calpactin-1 heavy chain;Chromobindin-8;	ANX2;ANX2L4;ANXA2;CAL1H;LPC2D;ANX2L2	Isoform 2 of Annexin A2;Isoform 1 of Annexin A2;24 kD	P07355-2;P07355	40.411	1.23E-56	1.3937	1.1133	55.982	14	0.1548424
UPF0027 protein C22orf28;cDNA FLJ58027;cDNA FLJ57752;cDNA FLJ57223;Ankyrin repeat domain	C22orf28;HSPC117;ANKRD54;Z97630.4-011	UPF0027 protein C22orf28;cDNA FLJ58027;cDNA FLJ57	Q9Y3I0;B4DNA0;B	55.21	0.0006324	1.4346	1.1131	101.78	3	0.1545832
Putative uncharacterized protein TOR1AIP1;Torsin-1A-interacting protein 1;Torsin A interacting pro	TOR1AIP1;RP11-533E19.1-002	68 kDa protein;Uncharacterized protein;Isoform 1 of To	A8MUM5;Q5JTV8	67.821	4.20E-09	1.464	1.1113	10.508	3	0.1522483
Histone acetyltransferase type B subunit 2;Histone-binding protein RBBP7;Nucleosome-remodeling	RBAP46;RBBP7;RP11-716A19.5-006;hCG_20	Histone-binding protein RBBP7;Retinoblastoma binding	Q16576;Q5JNZ9;C	47.82	4.37E-09	1.3947	1.1064	145.39	7	0.1458731
C-2K;Cell division cycle 2-like protein kinase 4;Cell division protein kinase 9;Cyclin-dependent kinas	CDC2L4;CDK9	Isoform 2 of Cyclin-dependent kinase 9;Isoform 1 of Cy	P50750-2;P50750	53.365	0.0043209	1.0842	1.105		1	0.1440464
75 kDa glucose-regulated protein;Heat shock 70 kDa protein 9;Mortalin;Peptide-binding protein 74	GRP75;HSPA9;HSPA9B	Stress-70 protein, mitochondrial;cDNA FLJ51903, highly	P38646;B7Z1V7;B	73.68	2.01E-21	1.3158	1.1021	7.7323	4	0.1402551
cDNA FLJ56344, highly similar to Implantation-associated protein;Implantation-associated protein;	IAG2;MAGT1;PSEC0084;UNQ628/PRO1244;	magnesium transporter protein 1;Magnesium transport	B4DH58;Q9H0U3;	41.531	0.020553	1.4037	1.1015		1	0.1394695
Prohibitin;cDNA FLJ78511, highly similar to Homo sapiens prohibitin (PHB), mRNA;cDNA, FLJ93035	PHB;hCG_29613	Prohibitin;Uncharacterized protein;Uncharacterized pro	P35232;A8K401;Q	29.804	7.55E-81	1.3098	1.0994	92.099	14	0.1367164
Cell proliferation-inducing gene 54 protein;Sister chromatid cohesion protein 112;Sister chromatid	KIAA0648;PDS5;PDS5A;PIG54	Isoform 1 of Sister chromatid cohesion protein PDS5 ho	Q29RF7-1;Q29RF7	150.83	1.26E-13	1.2454	1.0988	7.0413	3	0.1359288
Activity-dependent neuroprotective protein;Activity-dependent neuroprotector homeobox protein	ADNP;ADNP1;KIAA0784	Activity-dependent neuroprotector homeobox protein	Q9H2P0;B2RBM8;	123.56	0.0025387	1.2563	1.0894		1	0.1235338
Valyl-tRNA synthetase;Protein G7a;Valine--tRNA ligase	DAAP-21F2.2-001;DAMC-117B11.3-001;VAR	Valyl-tRNA synthetase;Valyl-tRNA synthetase;Valyl-tRN	B0V043;P26640;Q	140.46	2.93E-17	1.2447	1.0864	26.148	9	0.1195554
Alpha-CP1;Heterogeneous nuclear ribonucleoprotein E1;Nucleic acid-binding protein SUB2.3;Poly(	PCBP1;hCG_1776997	Poly(rC)-binding protein 1	Q15365;Q53SS8	37.497	2.60E-118	1.3237	1.0845	131.96	11	0.1170301
cDNA FLJ77715, highly similar to Homo sapiens cisplatin resistance-associated overexpressed prote	CREAP1;CROP;LUC7L3;O48	cDNA FLJ77715, highly similar to Homo sapiens cisplati	A8K3C5;O95232-1	58.22	0.00039526	1.307	1.0835		1	0.1156992
Structural maintenance of chromosomes flexible hinge domain-containing protein 1	KIAA0650;SMCHD1	Isoform 1 of Structural maintenance of chromosomes fl	A6NHR9-1;A6NHR	226.37	2.81E-09	1.0319	1.0819	28.659	4	0.1135672
BAG family molecular chaperone regulator 2;Bcl-2-associated athanogene 2;cDNA FLJ10153 fis, clo	BAG2	BAG family molecular chaperone regulator 2	O95816;B3KM36;	23.772	0.002906	1.2136	1.0797		1	0.1106305
70 kDa lamin;Lamin-A/C;Renal carcinoma antigen NY-REN-32;Lamin A/C transcript variant 1;Lamin	LMN1;LMNA;RP11-54H19.1-004;RP11-54H1	Isoform A of Prelamin-A/C;Isoform ADelta10 of Prelami	P02545-1;P02545	74.139	8.99E-111	1.5147	1.0642	60.064	35	0.0897693
Coding region determinant-binding protein;IGF-II mRNA-binding protein 1;Insulin-like growth facto	CRDBP;IGF2BP1;VICKZ1;ZBP1;hCG_27512	Insulin-like growth factor 2 mRNA-binding protein 1;ins	Q9NZI8;D3DTW3;	63.48	1.32E-32	1.3366	1.0632	124.32	3	0.088413
Constitutive coactivator of PPAR-gamma-like protein 1;Oxidative stress-associated Src activator;Pro	C9orf10;FAM120A;KIAA0183;OSSA	Isoform F of Constitutive coactivator of PPAR-gamma-lik	Q9NZB2-6;Q9NZB	125.27	5.95E-05	1.2388	1.0612		1	0.0856966
1-phosphatidylinositol-4,5-bisphosphate phosphodiesterase beta-3;Phosphoinositide phospholipas	PLCB3	1-phosphatidylinositol-4,5-bisphosphate phosphodieste	Q01970;Q8N1A4	138.8	6.68E-05	1.0211	1.0598	4.9266	2	0.083792
DNA-dependent protein kinase catalytic subunit;DNPK1;p460;cDNA FLJ57825, highly similar to DN	HYRC;HYRC1;PRKDC	Isoform 1 of DNA-dependent protein kinase catalytic su	P78527-1;P78527	469.08	5.48E-138	1.6707	1.0584	81.894	31	0.081885
DNA polymerase delta subunit 2;DNA polymerase delta subunit p50;cDNA FLJ78293, highly similar	POLD2;hCG_1745196;tcag7.802	DNA polymerase delta subunit 2;Uncharacterized prote	P49005;A4D2J4;B	51.289	2.23E-05	1.2229	1.0565		1	0.0792928
Ras-related protein Rab-39A;Ras-related protein Rab-6A;cDNA FLJ51066, highly similar to Ras-relat	RAB39;RAB39A;RAB6;RAB6A;RAB6B;RAB6A'	Ras-related protein Rab-39A;Isoform 1 of Ras-related pr	Q14964;P20340-1	25.006	0.0051033	1.3622	1.0544		1	0.0764223
49 kDa TATA box-binding protein-interacting protein;54 kDa erythrocyte cytosolic protein;INO80 co	INO80H;NMP238;RUVBL1;TIP49;TIP49A	Isoform 1 of RuvB-like 1;Isoform 2 of RuvB-like 1;30 kDa	Q9Y265-1;Q9Y265	50.227	4.93E-19	1.349	1.0537	90.695	7	0.0754642
SM22-alpha homolog;Transgelin-2;Putative uncharacterized protein TAGLN3	CDABP0035;KIAA0120;TAGLN2;TAGLN3	24 kDa protein;Transgelin-2;Uncharacterized protein	P37802;C9J5W6	24.454	0.04266	1.1082	1.0513		1	0.0721744
cDNA FLJ13963 fis, clone Y79AA1001299, highly similar to Homo sapiens integrase interactor 1b pr	BAF47;INI1;SMARCB1;SNF5L1;hCG_41099	cDNA FLJ13963 fis, clone Y79AA1001299, highly similar	Q9H836;Q12824-	45.05	0.00049226	1.3334	1.0496		1	0.0698396
U1 small nuclear ribonucleoprotein A;cDNA, FLJ93681, highly similar to Homo sapiens small nuclea	SNRPA	U1 small nuclear ribonucleoprotein A;cDNA FLJ52249, h	P09012;B2R802;B	31.279	4.50E-13	1.1963	1.0496	53.413	6	0.0698396
Transmembrane protein 214;TMEM214 protein;cDNA, FLJ96404;cDNA FLJ39989 fis, clone STOMA2	PP446;TMEM214;FLJ20254;hCG_23721	Transmembrane protein 214;transmembrane protein 2	Q6NUQ4;B3LEU8;	77.15	0.007652	1.1219	1.0462	2.881	2	0.0651587
5F7;Basigin;Collagenase stimulatory factor;Extracellular matrix metalloproteinase inducer;Leukocy	BSG;UNQ6505/PRO21383;hCG_20562;hEM	Isoform 1 of Basigin;Isoform 2 of Basigin;basigin isofor	P35613-1;P35613	42.2	0.22917	1.2797	1.0327		1	0.0464212
Antiapoptosis clone 11 protein;Apoptosis inhibitor 5;Cell migration-inducing gene 8 protein;Fibrob	API5;MIG8	apoptosis inhibitor 5 isoform a;Isoform 1 of Apoptosis i	Q9BZZ5-4;Q9BZZ5	59.004	1.39E-20	1.103	1.0265	5.4472	3	0.0377336
cDNA FLJ60076, highly similar to ELAV-like protein 1;ELAV-like protein 1;Hu-antigen R	ELAVL1;HUR	cDNA FLJ60076, highly similar to ELAV-like protein 1;EL	B4DVB8;Q15717	38.996	2.02E-79	1.3061	1.0248	83.214	31	0.0353424
Nucleolar protein EMG1 homolog;Probable ribosome biogenesis protein NEP1;Protein C2f;cDNA FL	C2F;EMG1;hCG_25956	Probable ribosome biogenesis protein NEP1;cDNA FLJ6	Q92979;A8K6D2;	26.72	4.46E-08	1.3495	1.0239	19.938	3	0.0340748
mRNA export factor TAP;Nuclear RNA export factor 1;Tip-associated protein;Tip-associating protei	NXF1;TAP;DKFZp667O0311;hCG_2020139	Nuclear RNA export factor 1;nuclear RNA export factor	Q9UBU9;Q68CW9	70.182	0.014734	1.0364	1.022	25.024	2	0.0313952
Far upstream element-binding protein 2;KH type-splicing regulatory protein;p75;cDNA FLJ51330, h	FUBP2;KHSRP	Isoform 1 of Far upstream element-binding protein 2;Is	Q92945-1;Q92945	73.114	7.52E-53	1.4381	1.0122	57.174	15	0.0174944
Protein raver-1;Ribonucleoprotein PTB-binding 1	KIAA1978;RAVER1	ribonucleoprotein PTB-binding 1;Isoform 1 of Ribonucle	Q8IY67-2;Q8IY67;	79.578	1.07E-05	1.2402	1.0122		1	0.0174944
Putative uncharacterized protein YAF2;YY1-associated factor 2;cDNA FLJ59574, highly similar to YY	YAF2	YY1 associated factor 2;Isoform 2 of YY1-associated fact	C9JKP3;Q8IY57-1;	22.546	0.0085798	1.1578	1.0112	8.0244	2	0.0160684
47 kDa heat shock protein;Arsenic-transactivated protein 3;Cell proliferation-inducing gene 14 prot	CBP1;CBP2;HSP47;PIG14;SERPINH1;SERPIN	Serpin H1;cDNA FLJ52569, highly similar to Collagen-bi	P50454;A8K259;Q	46.44	8.39E-08	1.2517	1.007		1	0.0100637
DEAD box protein 21;Gu-alpha;Nucleolar RNA helicase 2;Nucleolar RNA helicase Gu;Nucleolar RNA	DDX21;OK/SW-cl.65	Isoform 1 of Nucleolar RNA helicase 2;Isoform 2 of Nucl	Q9NR30-1;Q9NR3	87.343	1.22E-47	1.5092	1.0035	110.56	27	0.0050406
Cob(I)alamin adenosyltransferase;Cob(I)yrinic acid a,c-diamide adenosyltransferase, mitochondrial	MMAB	Cob(I)yrinic acid a,c-diamide adenosyltransferase, mito	Q96EY8;B2R6J3;B	27.388	0.013877	1.1833	1.0034		1	0.0048968
L1880;RAB5L;Ras-related protein Rab-5C	RAB5C;RABL	Ras-related protein Rab-5C	P51148	23.482	0.00012122	1.2258	0.98917	228.33	3	-0.0157096
p195;Ras GTPase-activating-like protein IQGAP1;IQ motif containing GTPase activating protein 1;c	IQGAP1;KIAA0051	Ras GTPase-activating-like protein IQGAP1	P46940;A4QPB0;E	189.25	3.52E-23	1.3493	0.98366	62.359	7	-0.0237684
Eukaryotic translation initiation factor 2 subunit 1;Eukaryotic translation initiation factor 2 subunit	EIF2A;EIF2S1;hCG_21909	Eukaryotic translation initiation factor 2 subunit 1	P05198;Q53XC0	36.112	9.72E-09	1.0191	0.96381	15.243	3	-0.0531793
Uncharacterized protein C1orf77	C1orf77;HT031;PP7704	Isoform 2 of Friend of PRMT1 protein;Isoform 1 of Frien	Q9Y3Y2-3;Q9Y3Y2	26.524	1.27E-26	1.0754	0.95779	17.747	2	-0.0622187
MRG-binding protein;Chromosome 20 open reading frame 20, isoform CRA_a;URCC4 protein	C20orf20;MRGBP;hCG_23417;URCC4	MRG-binding protein	Q9NV56;A8C4L5	22.417	4.42E-11	1.1775	0.95229		1	-0.0705271
140 kDa nucleolar phosphoprotein;Hepatitis C virus NS5A-transactivated protein 13;Nucleolar 130	KIAA0035;NOLC1;NS5ATP13;hCG_25636	Isoform Beta of Nucleolar and coiled-body phosphopro	Q14978-2;Q14978	74.746	1.35E-05	1.0122	0.94873	8.2317	4	-0.0759305
ADP-ribosylation factor-like protein 6-interacting protein 5;Cytoskeleton-related vitamin A-respons	ARL6IP5;DERP11;HSPC127;JWA;PRA2;PRAF3	PRA1 family protein 3;cDNA FLJ52128, highly similar to	O75915;B3KQB4;E	21.614	1.23E-11	1.1645	0.94395	16.202	2	-0.0832177
Antigen NY-CO-4;Elongation factor 1-delta;cDNA, FLJ95068, highly similar to Homo sapiens eukary	EEF1D;EF1D;hCG_1994718	Isoform 2 of Elongation factor 1-delta;71 kDa protein;Is	P29692-2;P29692	71.407	5.34E-31	1.2035	0.94388	96.779	13	-0.0833246
Smu-1 suppressor of mec-8 and unc-52 protein homolog;WD40 repeat-containing protein SMU1;C	SMU1;hCG_30278	WD40 repeat-containing protein SMU1	Q2TAY7;A0MNN4;	57.543	0.060577	1.1395	0.93291		1	-0.1001902
Actin-binding protein 280;Alpha-filamin;Endothelial actin-binding protein;Filamin-1;Filamin-A;Non-	FLN;FLN1;FLNA;FLJ00119;XX-FW83128A1.1-	Isoform 1 of Filamin-A;Isoform 2 of Filamin-A;Filamin A,	P21333-1;P21333	280.74	5.12E-201	1.4768	0.9294	61.151	36	-0.1056285
Protein LAS1 homolog;Las1-like;cDNA FLJ55066, highly similar to LAS1-like protein	LAS1L;MSTP060	Isoform 1 of Protein LAS1 homolog;Isoform 2 of Protein	Q9Y4W2-1;Q9Y4W	83.064	1.08E-05	1.0906	0.92868		1	-0.1067465
Putative uncharacterized protein RPL26;60S ribosomal protein L26-like 1;60S ribosomal protein L2	RPL26;RPL26L1;RPL26P1	Uncharacterized protein;60S ribosomal protein L26-like	A6NE05;Q9UNX3;	18.254	0.0015674	1.0578	0.92505	61.186	4	-0.1123967
Ally of AML-1 and LEF-1;bZIP-enhancing factor BEF;THO complex subunit 4;Transcriptional coactiva	ALY;BEF;THOC4	THO complex subunit 4	Q86V81	27.557	2.60E-41	1.2049	0.92329	24.071	14	-0.1151442
KRAB-associated protein 1;KRAB-interacting protein 1;Nuclear corepressor KAP-1;RING finger prote	KAP1;RNF96;TIF1B;TRIM28	Isoform 1 of Transcription intermediary factor 1-beta;Is	Q13263-1;Q13263	88.549	4.92E-05	0.99244	0.92169	40.798	6	-0.1176465
Autoantigen NOR-90;Nucleolar transcription factor 1;Upstream-binding factor 1;cDNA FLJ77827, h	UBF;UBF1;UBTF;hCG_30294	Isoform UBF1 of Nucleolar transcription factor 1;Isofor	P17480-1;P17480	89.405	1.99E-06	1.169	0.92078	31.472	3	-0.1190716
Histone H1.5;Histone H1a	H1F5;HIST1H1B	Histone H1.5	P16401	22.58	1.04E-06	0.80227	0.9142	138.12	4	-0.1294183
Glycine- and tyrosine-rich RNA-binding protein;Heterogeneous nuclear ribonucleoprotein Q;NS1-a	HNRPQ;NSAP1;SYNCRIP;RP1-3J17.2-003;hC	Isoform 3 of Heterogeneous nuclear ribonucleoprotein	O60506-3;O6050	62.656	8.97E-92	1.3108	0.91168	116.86	36	-0.1334006
40S ribosomal protein S3a;v-fos transformation effector protein;cDNA FLJ78591, highly similar to H	FTE1;MFTL;RPS3A	40S ribosomal protein S3a;30 kDa protein;Uncharacteri	P61247;A8K4W0;	29.945	0.015874	1.1417	0.9041		1	-0.1454457
ER-Golgi SNARE of 24 kDa;SEC22 vesicle-trafficking protein homolog B;SEC22 vesicle-trafficking pro	SEC22B;SEC22L1	Vesicle-trafficking protein SEC22b	O75396	24.593	1.78E-57	1.1598	0.90315	100.26	5	-0.1469625
18 kDa Alu RNA-binding protein;Signal recognition particle 14 kDa protein	SRP14	Signal recognition particle 14 kDa protein;9 kDa protein	P37108	14.57	1.33E-26	1.012	0.90287	17.175	6	-0.1474098
NHP2 non-histone chromosome protein 2-like 1 (S. cerevisiae);High mobility group-like nuclear pro	CTA-216E10.8-008;NHP2L1;CTA-216E10.8-0	NHP2 non-histone chromosome protein 2-like 1;NHP2-	B1AHD1;P55769;	14.627	0.00034368	1.1156	0.89718		1	-0.1565306
Nucleolysin TIA-1 isoform p15;Nucleolysin TIA-1 isoform p40;p15-TIA-1;p40-TIA-1;RNA-binding pro	TIA1;hCG_40580	Isoform Long of Nucleolysin TIA-1 isoform p40;Unchara	P31483-1;P31483	42.963	3.61E-20	1.0716	0.89155	46.903	3	-0.1656124
ATP-dependent RNA helicase #46;DEAH box protein 15;Putative pre-mRNA-splicing factor ATP-dep	DBP1;DDX15;DHX15	Putative pre-mRNA-splicing factor ATP-dependent RNA	O43143;B4E0S6	90.932	2.05E-16	0.82472	0.88723	151.45	8	-0.1726199
GATA zinc finger domain-containing protein 2B;p66/p68;Transcriptional repressor p66-beta;cDNA F	GATAD2B;KIAA1150	Transcriptional repressor p66-beta	Q8WXI9;B3KSZ4	65.26	1.09E-24	1.2058	0.8777	80.84	6	-0.1882002
CCAAT-binding transcription factor I subunit A;DNA-binding protein B;Enhancer factor I subunit A;N	NSEP1;YB1;YBX1	Nuclease-sensitive element-binding protein 1;Protein;N	P67809;A0JLU4;Q	35.924	1.81E-233	0.83556	0.87659	145.97	25	-0.1900259
53 kDa BRG1-associated factor A;Actin-like protein 6A;Actin-related protein Baf53a;ArpNbeta;BRG	ACTL6A;BAF53;BAF53A;INO80K;hCG_16476	Isoform 1 of Actin-like protein 6A;Isoform 2 of Actin-like	O96019-1;O9601	47.46	0.0035577	1.137	0.87601		1	-0.1909808
Mediator of DNA damage checkpoint protein 1;Nuclear factor with BRCT domains 1;Mediator of D	KIAA0170;MDC1;NFBD1;DAQB-47P19.7-006	Isoform 1 of Mediator of DNA damage checkpoint prote	Q14676-1;Q1467	226.66	6.10E-36	1.1462	0.87557	60.448	6	-0.1917056
Inactive ubiquitin-specific peptidase 39;SAD1 homolog;U4/U6.U5 tri-snRNP-associated 65 kDa prot	CGI-21;HSPC332;PRO2855;USP39;hCG_3461	U4/U6.U5 tri-snRNP-associated protein 2;Uncharacteriz	Q53GS9;B7Z7L9;B	65.38	5.09E-20	1.0744	0.8738	63.979	7	-0.194625
Regulation of nuclear pre-mRNA domain-containing protein 2;cDNA FLJ54323	HSPC099;KIAA0460;RPRD2	Isoform 1 of Regulation of nuclear pre-mRNA domain-c	Q5VT52-1;Q5VT5	156.02	0.0022064	0.92095	0.86523	86.272	2	-0.2088444
cDNA FLJ60424, highly similar to Junction plakoglobin;Putative uncharacterized protein JUP	JUP	cDNA FLJ60424, highly similar to Junction plakoglobin	B4DE59;C9JKY1	62.615	1.01E-18	1.0505	0.86156		1	-0.2149768

cDNA FLJ59683, highly similar to Homo sapiens malignant T cell amplified sequence 1 (MCTS1), mR	MCT1;MCTS1	malignant T cell-amplified sequence 1 isoform 2;Isoforn	B4DGY2;Q9ULC4-:	20.55	0.0013696	0.88895	0.86004	12.177	2	-0.2175243
C1 factor;CFF;HCF C-terminal chain 1;HCF C-terminal chain 2;HCF C-termina	HCF1;HCFC1;HFC1	Isoform 1 of Host cell factor 1;Isoform 2 of Host cell fact	P51610-1;P51610	208.73	4.97E-08	1.0959	0.85192	33.69	2	-0.2312101
Heat shock 70 kDa protein 14;Heat shock protein HSP60;HSP70-like protein 1;cDNA FLJ77376, high	HSP60;HSP70L1;HSPA14;hCG_25570	Heat shock 70 kDa protein 14;Heat shock 70kDa proteir	Q0VDF9;A8K8F8;E	54.794	1.62E-06	1.0753	0.84947	7.4048	2	-0.2353651
M-phase phosphoprotein homolog;Zinc finger RNA-binding protein;cDNA FLJ55527, highly similar t	ZFR	Zinc finger RNA binding protein;Uncharacterized proteir	Q96KR1;A8K849;E	117.69	2.82E-07	0.85344	0.84814	125.05	2	-0.2376257
Protein PR264;Splicing component, 35 kDa;Splicing factor SC35;Splicing factor, arginine/serine-rich	SFRS2;hCG_27842;LOC392896;tcag7.956;LO	Serine/arginine-rich splicing factor 2;cDNA FLJ52570, hi	Q01130;B3KUY1;C	25.476	1.27E-23	0.87492	0.84208	16.868	6	-0.2479708
Mammalian branch point-binding protein;Splicing factor 1;Transcription factor ZFM1;Zinc finger ge	SF1;ZFM1;ZNF162	Isoform 5 of Splicing factor 1;Isoform 1 of Splicing facto	Q15637-5;Q15637	80.62	2.28E-05	0.7631	0.84165	52.875	2	-0.2487077
Bromodomain adjacent to zinc finger domain protein 1B;hWALp2;Tyrosine-protein kinase BAZ1B;W	BAZ1B;WBSC10;WBSCR10;WBSCR9;WSTF	Isoform 1 of Tyrosine-protein kinase BAZ1B;Isoform 2 o	Q9UIG0-1;Q9UIG(	170.9	0.0078575	1.0819	0.84063		1	-0.2504572
Cell division control protein 2 homolog;Cyclin-dependent kinase 1;p34 protein kinase;Putative uncl	CDC2;DKFZp686L20222	Putative uncharacterized protein DKFZp686L20222;cell	P06493-1;P06493	34.778	5.36E-05	1.0403	0.83814	43.026	3	-0.2547368
40S ribosomal protein S7;cDNA, FLJ92055, Homo sapiens ribosomal protein S7 (RPS7), mRNA;Puta	RPS7;hCG_1784266	40S ribosomal protein S7;22 kDa protein;19 kDa proteir	P62081;Q57Z92;B	22.127	5.19E-11	0.83042	0.83291	108.34	5	-0.2637675
cDNA FLJ78091, highly similar to Homo sapiens ubiquitin-like, containing PHD and RING finger dom	UHRF1;ICBP90;NP95;RNF106;hCG_23497;hN	E3 ubiquitin-protein ligase UHRF1 isoform 2;E3 ubiquiti	A8K024;B2RBA9;C	91.099	1.48E-10	0.8691	0.8315	57.67	9	-0.2662118
BRG1-associated factor 57;SWI/SNF-related matrix-associated actin-dependent regulator of chrom	BAF57;SMARCE1	Isoform 1 of SWI/SNF-related matrix-associated actin-de	Q969G3-1;Q969G	46.649	0.0005753	1.0695	0.82238		1	-0.2821229
Metastasis-associated 1-like 1;Metastasis-associated protein MTA2;p53 target protein in deacetyla	MTA1L1;MTA2;PID;DKFZp686F2281	Metastasis-associated protein MTA2	Q94776;Q68DB1	75.022	2.24E-57	1.1221	0.82202	74.002	15	-0.2827546
Heterogeneous nuclear ribonucleoproteins A2/B1;Putative uncharacterized protein HNRNPA2B1	HNRNPA2B1;HNRPA2B1	Isoform B1 of Heterogeneous nuclear ribonucleoproteir	P22626-1;P22626	37.429	1.07E-155	1.0891	0.81883	91.267	74	-0.2883641
Protein DB83;Transmembrane protein 33;SHINC3	DB83;TMEM33;SHINC3	Transmembrane protein 33;Uncharacterized protein;Prc	P57088;A6QKW0;	27.978	0.035671	1.0143	0.81808	17.72	2	-0.2896862
cDNA FLJ75459;cDNA FLJ76290, highly similar to Homo sapiens TIA1 cytotoxic granule-associated f	TIAL1;hCG_40603	nucleolysin TIAR isoform 2;Nucleolysin TIAR;TIA1 cytotc	A8K4L9;A8K5C4;Q	43.448	1.42E-53	0.97722	0.81681	95.642	14	-0.2919276
Small nuclear ribonucleoprotein Sm D3;snRNP core protein D3;cDNA FLJ51872, highly similar to Sn	SNRPD3	19 kDa protein;Small nuclear ribonucleoprotein Sm D3;(	P62318;B4DJP7	18.829	1.11E-07	0.856	0.81587	104.21	2	-0.2935888
Protein RCC2;RCC1-like protein TD-60;Telophase disk protein of 60 kDa;RCC2 protein	KIAA1470;RCC2;TD60	Protein RCC2	Q9P258;A5PLK7	56.084	4.91E-27	1.1108	0.81569	82.307	18	-0.2939071
Mitochondrial transcription factor 1;Transcription factor 6;Transcription factor 6-like 2;Transcriptio	TCF6;TCF6L2;TFAM	Transcription factor A, mitochondrial;Protein;Uncharact	Q00059;E5KSU5;E	29.096	7.05E-20	1.0276	0.81305	41.942	13	-0.298584
SMARCA4 protein;SMARCA4 isoform 2;SWI/SNF related, matrix associated, actin dependent regula	SMARCA4;hCG_29955;BAF190A;BRG1;SNF2	SMARCA4 protein;transcription activator BRG1 isoform	B9EGQ8;Q9HBD4;	189.45	1.14E-12	1.157	0.80924	13.685	4	-0.3053605
Poly(RC)-binding protein 2 isoform b variant;Poly(RC) binding protein 2;Poly(RC) binding protein 2,	hCG_2017557;PCBP2	poly(rC)-binding protein 2 isoform a;Poly(rC)-binding pr	Q59HD4;Q6IPF4;C	38.651	1.21E-103	1.0032	0.80506	113.59	27	-0.3128318
DNA-directed RNA polymerase II subunit A;DNA-directed RNA polymerase II subunit RPB1;DNA-dir	POLR2;POLR2A	DNA-directed RNA polymerase II subunit RPB1	P24928;B4DET1;B	217.17	2.87E-08	1.2281	0.80243		1	-0.3175526
Nucleolysin TIA-1 isoform p15;Nucleolysin TIA-1 isoform p40;p15-TIA-1;p40-TIA-1;RNA-binding pro	TIA1	Isoform Short of Nucleolysin TIA-1 isoform p40;Uncharа	P31483-2;P31483	41.8	1.66E-36	0.89041	0.79249		1	-0.3355354
DNA polymerase;DNA polymerase delta catalytic subunit;DNA polymerase subunit delta p125	POLD;POLD1	DNA-directed polymerase delta 1;DNA polymerase delta	Q308M6;P28340;(	126.34	4.37E-05	0.91047	0.79053	52.182	5	-0.3391079
Far upstream element-binding protein 3;cDNA FLJ58115, highly similar to Far upstream element-bi	FBP3;FUBP3;hCG_31253;RP11-57C19.3-002	Isoform 1 of Far upstream element-binding protein 3;Ur	Q96I24-1;Q96I24;	61.64	1.22E-45	0.98549	0.79047	62.396	13	-0.3392174
cDNA FLJ56389, highly similar to Elongation factor 1-gamma;eEF-1B gamma;Elongation factor 1-ga	EEF1G;EF1G;PRO1608;hCG_2039458	cDNA FLJ56389, highly similar to Elongation factor 1-gar	B4DTG2;P26641;C	56.149	9.55E-37	1.0574	0.78943	99.459	24	-0.3411167
Membrane-organizing extension spike protein;Moesin;MSN protein;Radixin isoform b;Radixin isofc	MSN;hCG_39182;RDX;DKFZp434I0812	Moesin;Radixin, isoform CRA_a;Radixin	P26038;Q6PJT4;A	67.819	1.67E-22	0.87406	0.78809	48.829	8	-0.3435677
cDNA FLJ58953, highly similar to 40S ribosomal protein S20;40S ribosomal protein S20	RPS20	40S ribosomal protein S20 isoform 1;40S ribosomal prot	B4DW28;P60866;	16.005	0.001059	0.90418	0.7878		1	-0.3440987
AC40;DNA-directed RNA polymerases I and III 40 kDa polypeptide;DNA-directed RNA polymerases	POLR1C;POLR1E;RP3-337H4.4-009;RP3-337	Isoform 1 of DNA-directed RNA polymerases I and III sul	O15160-1;O1516C	39.249	0.0016019	0.8268	0.78559		1	-0.3481515
ATP-dependent helicase CHD3;Chromodomain-helicase-DNA-binding protein 3;Mi-2 autoantigen 2	CHD3	chromodomain-helicase-DNA-binding protein 3 isoform	Q12873-1;Q12873	233.03	4.83E-14	0.83662	0.78441		1	-0.3503202
Coronin-1C_i2 protein;Coronin-1C_i3 protein;cDNA FLJ13143 fis, clone NT2RP3003230, highly simi	CORO1C;CRNN4	Coronin-1C_i3 protein;Coronin-1C;cDNA FLJ50992, high	A7MAP0;A7MAP1	58.947	2.58E-11	0.91023	0.78332	189.08	3	-0.3523263
ATP-dependent helicase CHD4;Chromodomain-helicase-DNA-binding protein 4;Mi-2 autoantigen 2	CHD4	Isoform 2 of Chromodomain-helicase-DNA-binding prot	Q14839-2;Q14839	220.85	4.30E-40	1.1288	0.77179	80.026	15	-0.3737197
Hepatocellular carcinoma protein 1;RNA-binding motif protein 39;RNA-binding protein 39;RNA-bin	HCC1;RBM39;RNPC2;DKFZp781C0423;DKFZ	Isoform 1 of RNA-binding protein 39;Isoform 2 of RNA-k	Q14498-1;Q14498	59.379	1.88E-54	0.97677	0.77132	90.292	16	-0.3745986
Eukaryotic translation initiation factor 2 subunit 3;Eukaryotic translation initiation factor 2 subunit	EIF2G;EIF2S3;EIF2S3L	Eukaryotic translation initiation factor 2 subunit 3;Isofo	P41091;A8K2Y2;B	51.109	1.61E-06	0.88596	0.76044	193.53	3	-0.3950937
3'-5' exonuclease TREX1;DNase III;Three prime repair exonuclease 1;TREX1 protein;Three prime re	TREX1;hCG_1997849	Isoform 1 of Three prime repair exonuclease 1;Isoform 2	Q9NSU2-1;Q9NSU	38.923	7.79E-05	0.98496	0.75853		1	-0.3987219
cDNA FLJ55039, moderately similar to Hepatoma-derived growth factor;Hepatoma-derived growth	HDGF;HMG1L2;RP11-66D17.10-003;hCG_17	cDNA FLJ55039, moderately similar to Hepatoma-derive	B7Z525;P51858;B	28.819	1.84E-27	0.67013	0.75538	99.394	10	-0.4047255
L-lactate dehydrogenase;Cell proliferation-inducing gene 19 protein;LDH muscle subunit;L-lactate c	LDHA;PIG19;hCG_96677;LDHAL6;LDHAL6B;L	L-lactate dehydrogenase A chain isoform 3;Isoform 1 of	B7Z5E3;P00338-1;	39.837	0.011863	0.93782	0.75305		1	-0.4091824
cDNA FLJ53464, highly similar to Threonyl-tRNA synthetase, cytoplasmic (EC 6.1.1.3);Threonyl-tRNA	TARS;TARSL2	cDNA FLJ53464, highly similar to Threonyl-tRNA synthet	B4DEG8;Q53GX7;	86.86	2.51E-06	0.923	0.75192	41.139	2	-0.4113489
Paraspeckle component 1;Paraspeckle protein 1;cDNA FLJ57805, highly similar to Homo sapiens pa	PSP1;PSPC1	Isoform 1 of Paraspeckle component 1;Isoform 2 of Para	Q8WXF1-1;Q8WX	58.743	1.14E-08	1.0421	0.75148	28.423	2	-0.4121934
Dolichyl-diphosphooligosaccharide--protein glycosyltransferase 48 kDa subunit;cDNA FLJ55714, hig	DDOST;KIAA0115;OK/SW-cl.45;OST48	Dolichyl-diphosphooligosaccharide--protein glycosyltrar	P39656;B4DLI2;B	50.8	3.34E-22	0.94139	0.74203	156.19	4	-0.4304506
DNA-binding protein/plasminogen activator inhibitor 1 regulator;Helicase-like transcription factor;H	HIP116A;HLTF;RNF80;SMARCA3;SNF2L3;ZBU	Isoform 1 of Helicase-like transcription factor;HLTF prot	Q14527-1;Q14527	113.93	5.78E-06	0.96405	0.74037	10.243	3	-0.4336817
Cavin-1;Polymerase I and transcript release factor;cDNA FLJ35087 fis, clone PLACE6005546, highly	FKSG13;PTRF	Isoform 1 of Polymerase I and transcript release factor;(	Q6NZI2-1;Q6NZI2	43.476	2.14E-08	0.87654	0.73995	9.8889	2	-0.4345003
CF-1 77 kDa subunit;Cleavage stimulation factor 77 kDa subunit;Cleavage stimulation factor subuni	CSTF3;hCG_27317	Cleavage stimulation factor subunit 3;cleavage stimulati	Q12996;B4DFQ5;E	82.921	7.30E-07	0.7574	0.73738	116.96	2	-0.4395198
ATP-binding cassette 50;ATP-binding cassette sub-family F member 1;TNF-alpha-stimulated ABC pr	ABC50;ABCF1;DADB-129D20.7-001;DAMA-1	Isoform 1 of ATP-binding cassette sub-family F member	Q8NE71-1;Q8NE7	95.925	2.80E-42	0.914	0.73121	133.88	10	-0.4516423
RCC1-I;Regulator of chromosome condensation 1;Putative uncharacterized protein RCC1;Cell cycle	RCC1;CHC1;hCG_27809;RP4-669K10.2-001	regulator of chromosome condensation isoform a;regul	Q16269;Q6NT97;(	48.145	7.29E-144	0.90155	0.72128	86.582	26	-0.4713687
A34.5;Antisense to ERCC-1 protein;CD3-epsilon-associated protein;DNA-directed RNA polymerase	ASE1;CAST;CD3EAP;PAF49	Isoform 2 of DNA-directed RNA polymerase I subunit RF	O15446-2;O15446	55.171	3.19E-13	0.93814	0.72026		1	-0.4734103
FSCN1 protein;55 kDa actin-bundling protein;Fascin;p55;Singed-like protein;cDNA FLJ37935 fis, clo	FSCN1;FAN1;HSN;SNL	FSCN1 protein (Fragment);Fascin	Q96IH1;Q16658;B	55.135	0.0038352	0.9269	0.71949		1	-0.4749535
Histone deacetylase 2;cDNA FLJ40057 fis, clone TCERX2000431, highly similar to HISTONE DEACETY	HDAC2;HDAC1;RPD3L1;DKFZp686H12203;h(	Histone deacetylase 2;Histone deacetylase 1;cDNA FLJ3	Q92769;B3KUJ5;C	65.537	3.76E-06	0.91989	0.71871	99.755	3	-0.4765183
Putative RNA-binding protein 3;RNA-binding motif protein 3;RNPL	RBM3;RNPL	Putative RNA-binding protein 3	P98179	17.17	2.80E-16	0.87801	0.71852	14.762	8	-0.4768998
Cyclophilin B;CYP-S1;Peptidyl-prolyl cis-trans isomerase B;Rotamase B;S-cyclophilin	CYPB;PPIB	Peptidyl-prolyl cis-trans isomerase B;20 kDa protein	P23284	23.742	1.82E-55	0.91546	0.71274	108.41	29	-0.4885522
Cell proliferation-inducing gene 21 protein;Guanine nucleotide-binding protein subunit beta-2-like	GNB2L1;HLC7;PIG21	Guanine nucleotide-binding protein subunit beta-2-like	P63244;D6REE5;D	35.076	2.23E-24	0.92148	0.71098	57.587	5	-0.4921191
DNA mismatch repair protein Msh6;G/T mismatch-binding protein;MutS-alpha 160 kDa subunit;cD	GTBP;MSH6	Isoform GTBP-N of DNA mismatch repair protein Msh6;	P52701-1;P52701	152.78	7.81E-32	1.0456	0.68474	95.948	13	-0.5463718
40S ribosomal protein S19;cDNA, FLJ92047, Homo sapiens ribosomal protein S19 (RPS19), mRNA;R	RPS19;hCG_1995572	40S ribosomal protein S19	P39019;B0ZBD0;C	16.06	4.09E-08	0.86388	0.68252	94.793	4	-0.5510568
APEX nuclease;Apurinic-apyrimidinic endonuclease 1;DNA-(apurinic or apyrimidinic site) lyase;Prot	APE;APEX;APEX1;APX;HAP1;REF1;hCG_4047	DNA-(apurinic or apyrimidinic site) lyase	P27695;Q5TZP7	35.554	1.47E-159	0.87753	0.68106	72.509	42	-0.5541462
Microtubule-associated protein 4;MAP4 protein;Microtubule-associated protein;cDNA FLJ58263, h	MAP4;DKFZp547H1810	245 kDa protein;Isoform 1 of Microtubule-associated pr	P27816-1;P27816	245.44	9.08E-19	1.0712	0.67236	70.339	6	-0.5726942
2'(3')-polynucleotidase;Bifunctional polynucleotide phosphatase/kinase;DNA 5'-kinase/3'-phospha	PNKP	Bifunctional polynucleotide phosphatase/kinase;PNKP p	Q96T60;Q9BUL2	57.076	0.0030099	0.80973	0.66834		1	-0.5813459
Ribosome-recycling factor, mitochondrial;Ribosome-releasing factor, mitochondrial;Novel protein s	MRRF;RP1-57A13.2;RP1-57A13.2-001	Isoform 1 of Ribosome-recycling factor, mitochondrial;N	Q96E11-1;Q96E11	29.277	0.00024268	0.79096	0.66519	130.17	3	-0.5881616
Bcl-XL-binding protein v68;Phosphoglycerate mutase family member 5;Serine/threonine-protein pl	PGAM5;PGAM5-L	Isoform 1 of Serine/threonine-protein phosphatase PGA	Q96HS1-1;Q96HS	32.004	0.0012435	0.68963	0.66443	111.83	2	-0.5898109
Coiled-coil domain-containing protein 124	CCDC124	Coiled-coil domain-containing protein 124	Q96CT7	25.835	4.96E-06	0.74343	0.65793	17.365	7	-0.603994
Damage-specific DNA-binding protein 1;DDB p127 subunit;DNA damage-binding protein 1;DNA dai	DDB1;XAP1	DNA damage-binding protein 1;cDNA FLJ51067, highly s	Q16531;B7Z2A1;B	126.97	0.00024638	0.84163	0.65378	6.4049	2	-0.6131229
60S ribosomal protein L22;EBER-associated protein;Epstein-Barr virus small RNA-associated proteir	RPL22	60S ribosomal protein L22;11 kDa protein;9 kDa proteir	P35268;Q7Z4W8;(	14.787	0.0036503	0.75948	0.65315		1	-0.6145137
CXXC-type zinc finger protein 9;DNA (cytosine-5)-methyltransferase 1;DNA methyltransferase HsaI;	AIM;CXXC9;DNMT;DNMT1	Isoform 1 of DNA (cytosine-5)-methyltransferase 1;Isofc	P26358-1;P26358	189.56	1.81E-35	0.9343	0.64864	52.558	22	-0.6245101
Histone H2B;Histone H2B type 1-N;Histone H2B.d;Histone H2B type 1-C/E/F/G/I;Histone H2B.1 A;H	H2BFD;HIST1H2BN;H2BFA;H2BFG;H2BFH;H2	cDNA FLJ56787, highly similar to Histone H2B type 2-F;H	B4DR52;Q99877;E	18.041	2.02E-31	0.8638	0.6484	90.738	11	-0.625044
3-alkyladenine DNA glycosylase;3-methyladenine DNA glycosidase;ADPG;DNA-3-methyladenine gly	AAG;ANPG;MID1;MPG;hCG_22412;PIG16;Z(	Isoform 1 of DNA-3-methyladenine glycosylase;Isoform	P29372-1;P29372	32.868	2.01E-52	0.8563	0.64729	24.66	25	-0.6275159
Histone H2A type 1;Histone H2A/p;Histone H2A;Histone H2A type 1-D;Histone H2A.3;Histone H2A,	H2AFC;H2AFD;H2AFI;H2AFN;H2AFP;HIST1H2	Histone H2A type 1;Histone H2A type 1-D;Histone H2A	P0C0S8;A4FTV9;P	14.091	1.03E-38	0.79852	0.64455	142.48	10	-0.6336358
Cleavage and polyadenylation specificity factor 68 kDa subunit;Cleavage and polyadenylation speci	CFIM68;CPSF6	Isoform 2 of Cleavage and polyadenylation specificity fa	Q16630-2;Q1663C	63.47	2.79E-06	0.53762	0.63989		1	-0.6441042
AU-rich element RNA-binding factor;Heterogeneous nuclear ribonucleoprotein D-like;JKT41-bindin	HNRPDL;JKTBP	Isoform 1 of Heterogeneous nuclear ribonucleoprotein	O14979-1;O1497	46.437	4.86E-10	0.79847	0.63578	55.131	4	-0.6534005
cDNA FLJ54030, highly similar to Polymerase delta-interacting protein 3;46 kDa DNA polymerase d	KIAA1649;PDIP46;POLDIP3;DKFZp434G0310	cDNA FLJ54030, highly similar to Polymerase delta-inter	B4E0L0;Q9BY77-1	48.101	1.12E-13	0.66639	0.62456	176.38	2	-0.6790879
Chromatin assembly factor 1 subunit B;Chromatin assembly factor I p60 subunit;M-phase phospho	CAF1A;CAF1P60;CHAF1B;MPHOSPH7;MPP7	Chromatin assembly factor 1 subunit B	Q13112;B2R7X3	61.492	0.0074106	0.70755	0.62091		1	-0.6875439
Mitotic checkpoint protein BUB3;cDNA FLJ57899, highly similar to Mitotic checkpoint protein BUB:	BUB3	Isoform 1 of Mitotic checkpoint protein BUB3;Isoform 2	O43684-1;O43684	37.154	1.16E-11	0.63775	0.61795	138.7	8	-0.694438
Histone H3.3;Histone H3;Histone H3.1;Histone H3/a;Histone H3/b;Histone H3/c;Histone H3/d;Hist	H3.3A;H3.3B;H3F3;H3F3A;H3F3B;PP781;hC(	Histone H3.3;cDNA, FLJ93172, highly similar to Homo sa	P84243;B2R4P9;E	15.328	0.00026702	0.6889	0.61715	27.25	6	-0.6963069
UPF0361 protein C3orf37;cDNA FLJ57398	C3orf37;DC12	UPF0361 protein C3orf37;cDNA FLJ57398;Uncharacteri	Q96FZ2;B4DMK2;	40.574	3.14E-07	0.64101	0.61448	7.7988	2	-0.702562
DAZ-associated protein 1;Deleted in azoospermia-associated protein 1;cDNA FLJ35575 fis, clone SP	DAZAP1	Isoform 1 of DAZ-associated protein 1;Isoform 2 of DAZ	Q96EP5-1;Q96EP5	43.383	4.14E-57	0.81341	0.6076	60.756	15	-0.7188062
Bruton tyrosine kinase-associated protein 135;General transcription factor II-I;SRF-Phox1-interactir	BAP135;GTF2I;WBSCR6	Isoform 1 of General transcription factor II-I;Isoform 3 o	P78347-1;P78347	112.42	3.64E-61	0.78771	0.60587	73.985	21	-0.7229198
cDNA FLJ51656, highly similar to Actin-like protein 2;Actin-like protein 2;Actin-related protein 2;AC	ACTR2;ARP2	actin-related protein 2 isoform a;Actin-related protein 2	B4DHK9;P61160;C	45.376	1.57E-08	0.74191	0.60538	5.1545	3	-0.7240871
Dermal papilla-derived protein 9;ELL-associated protein of 20 kDa;ESCRT-II complex subunit VPS25	DERP9;EAP20;VPS25	Vacuolar protein-sorting-associated protein 25	Q9BRG1	20.747	0.015056	0.73561	0.60223		1	-0.7316135

Treacher Collins syndrome protein;Treacle protein;TCOF1 protein variant;Putative uncharacterized	TCOF1;DKFZp434G1035	Isoform 4 of Treacle protein;Isoform 3 of Treacle protei	Q13428-4;Q1342	156.06	3.84E-95	0.89118	0.59577	108.57	17	-0.7471726
Polypyrimidine tract binding protein 1;Polypyrimidine tract binding protein 1, isoform CRA_c;57 kD	hCG_20560;PTBP1;PTB	polypyrimidine tract-binding protein 1 isoform a;Isofor	Q9BUQ0;P26599-	59.632	8.80E-191	0.67182	0.59485	117.98	59	-0.7494022
WD repeat-containing protein 18	WDR18	WD repeat-containing protein 18	Q9BV38	47.405	0.00081502	0.51722	0.59031		1	-0.7604553
Importin subunit alpha-2;Karyopherin subunit alpha-2;RAG cohort protein 1;SRP1-alpha;cDNA FLJ	KPNA2;RCH1;SRP1	Importin subunit alpha-2;Karyopherin alpha 2	P52292;A8K7D9;C	57.861	3.71E-05	0.6219	0.58655	91.373	4	-0.769674
ATP-dependent DNA helicase VIII;GAP SH3 domain-binding protein 1;Ras GTPase-activating protein	G3BP;G3BP1;DKFZp313K0921;DKFZp686L11	Ras GTPase-activating protein-binding protein 1;CDNA	Q13283;B7Z8K4;E	52.164	0.00013748	0.58954	0.58197	73.127	2	-0.7809833
Tubulin beta-2 chain;Tubulin beta-2C chain;Class IVb beta tubulin;Tubulin, beta 2C;TUBB2C protein	TUBB2C;XTP3TPATP1;DAAP-285E11.4-002;D	Tubulin beta-2C chain;cDNA FLJ11352 fis, clone HEMBA	P68371;Q8IWP6;C	49.83	9.85E-18	0.62187	0.5714	187	2	-0.8074271
Natural killer cell-enhancing factor B;Peroxiredoxin-2;PRP;Thiol-specific antioxidant protein;Thiore	NKEFB;PRDX2;TDPX1;hCG_172488	Peroxiredoxin-2;cDNA FLJ60461, highly similar to Peroxi	P32119;B4DF70;A	21.892	5.67E-06	0.58939	0.56646		1	-0.819954
60 kDa poly(U)-binding-splicing factor;FUSE-binding protein-interacting repressor;Poly(U)-binding-	FIR;PUF60;ROBPI;SIAHBP1	Isoform 2 of Poly(U)-binding-splicing factor PUF60;Isofo	Q9UHX1-2;Q9UH	58.171	3.80E-26	0.60914	0.5573	54.317	12	-0.8434739
NOL1/NOP2/Sun domain family member 2;Substrate of AIM1/Aurora kinase B;tRNA (cytosine-5-)-	NSUN2;SAKI;TRM4;hCG_18368	tRNA (cytosine-5-)-methyltransferase NSUN2;cDNA FLJ	Q08J23;A8K529;B	86.47	7.19E-32	0.62343	0.55577	43.548	6	-0.8474401
Telomeric repeat-binding factor 2-interacting protein 1;TRF2-interacting telomeric protein Rap1	PP8000;RAP1;TERF2IP	Telomeric repeat-binding factor 2-interacting protein 1	Q9NYB0	44.259	5.86E-05	0.44967	0.54059		1	-0.8873933
Arginine-rich 54 kDa nuclear protein;Splicing factor, arginine/serine-rich 11;Splicing factor, arginine	SFRS11;hCG_22837;RP4-677H15.3-002;DKF	Serine/arginine-rich splicing factor 11;Splicing factor, ar	Q05519;Q8IWE6;	53.542	7.15E-06	0.49277	0.53997	54.5	2	-0.8890488
28 kDa heat- and acid-stable phosphoprotein;PDGFA-associated protein 1;PDGF-associated protein	HASPP28;PDAP1	28 kDa heat- and acid-stable phosphoprotein;4 kDa pro	Q13442	20.63	0.0026033	0.44782	0.5372		1	-0.8964688
Interleukin enhancer-binding factor 2;Nuclear factor of activated T-cells 45 kDa;cDNA FLJ51660, hi	ILF2;NF45;PRO3063	Interleukin enhancer-binding factor 2;12 kDa protein	Q12905;B4DY09;C	43.062	3.65E-77	0.53447	0.53326	110.73	12	-0.907089
Splicing factor U2AF 65 kDa subunit;U2 auxiliary factor 65 kDa subunit;U2 snRNP auxiliary factor la	U2AF2;U2AF65	Isoform 1 of Splicing factor U2AF 65 kDa subunit;Isofor	P26368-1;P26368	53.5	6.43E-19	0.5879	0.52334	157.64	4	-0.9341796
Heterogeneous nuclear ribonucleoprotein A0	HNRNPA0;HNRPA0	Heterogeneous nuclear ribonucleoprotein A0	Q13151	30.84	2.92E-104	0.63754	0.51974	133.46	42	-0.944138
Heterogeneous nuclear ribonucleoprotein U;p120;pp120;Scaffold attachment factor A;cDNA FLJ4	HNRNPU;HNRPU;SAFA;U21.1;RP11-11N7.3-	Isoform Long of Heterogeneous nuclear ribonucleopro	Q00839-1;Q0083	90.583	2.58E-222	0.75189	0.51852	136.5	82	-0.9475285
Adenylate cyclase-stimulating G alpha protein;Extra large alphas protein;Guanine nucleotide-bindi	GNAS;GNAS1;GSP;hCG_1788380;GNAL;hCG	Isoform XLas-1 of Guanine nucleotide-binding protein G	Q5JWF2-1;Q5JWF	111.02	3.06E-10	0.52874	0.50413	152.59	2	-0.9881323
DNA helicase V;Far upstream element-binding protein 1;cDNA FLJ59240, highly similar to Far upstr	FUBP1	Isoform 1 of Far upstream element-binding protein 1;c	Q96AE4-1;Q96AE	67.56	1.61E-126	0.56546	0.49544	97.354	17	-1.0132177
RNA-binding protein Musashi homolog 2;cDNA FLJ56904, highly similar to RNA-binding protein Mu	MSI2	Isoform 1 of RNA-binding protein Musashi homolog 2;c	Q96DH6-1;Q96DH	35.196	5.53E-12	0.42093	0.48468	42.971	2	-1.0448955
Cytovillin;Ezrin;p81;Villin-2;cDNA, FLJ92973, highly similar to Homo sapiens villin 2 (ezrin) (VIL2), m	EZR;VIL2	Ezrin;69 kDa protein	P15311;B2R6J2;B	69.412	3.39E-16	0.48971	0.45547	77.121	2	-1.1345721
Eukaryotic translation initiation factor 5B;Translation initiation factor IF-2;EIF5B protein;cDNA FLJ1	EIF5B;IF2;KIAA0741;hCG_38421	Eukaryotic translation initiation factor 5B	O60841;A0JLR8;B	138.83	2.68E-23	0.66345	0.45504	100.38	7	-1.1359347
40S ribosomal protein S5;40S ribosomal protein S5, N-terminally processed;Ribosomal protein S5 v	RPS5	40S ribosomal protein S5	P46782;Q53G25	22.876	0.038659	0.55759	0.43149	76.043	3	-1.212601
33 kDa VAMP-associated protein;Vesicle-associated membrane protein-associated protein A;cDNA	VAP33;VAPA;UNQ484/PRO983;VAPB;hCG_2	Isoform 2 of Vesicle-associated membrane protein-asso	Q9P0L0-2;Q9P0L0	32.613	3.80E-12	0.4286	0.42944	95.805	2	-1.2194715
Beta-II spectrin;Fodrin beta chain;Spectrin beta chain, brain 1;Spectrin, non-erythroid beta chain 1	SPTB2;SPTBN1;hCG_17370	Isoform Long of Spectrin beta chain, brain 1;Isoform Sh	Q01082-1;Q0108	274.61	3.99E-12	0.47514	0.42508	73.037	2	-1.2341937
cDNA FLJ56425, highly similar to Very-long-chain specific acyl-CoAdehydrogenase, mitochondrial (	ACADVL;VLCAD;hCG_42007	cDNA FLJ56425, highly similar to Very-long-chain specif	B4DEA8;Q53HR2;	75.209	7.11E-09	0.35939	0.42141	43.061	2	-1.2467035
Caprin-1;Cell cycle-associated protein 1;Cytoplasmic activation- and proliferation-associated prote	CAPRIN1;GPIAP1;GPIP137;M11S1	Isoform 1 of Caprin-1;Isoform 2 of Caprin-1;cDNA FLJ57	Q14444-1;Q1444	78.365	1.65E-05	0.33876	0.41681		1	-1.2625382
Heterogeneous nuclear ribonucleoprotein L;cDNA, FLJ94229, highly similar to Homo sapiens heter	HNRNPL;HNRPL;P/OKcl.14	Heterogeneous nuclear ribonucleoprotein L;heterogene	P14866;B2R959;Q	64.132	4.54E-43	0.38536	0.41379	113.82	32	-1.2730293
cDNA FLJ57954, highly similar to 60S ribosomal protein L28;60S ribosomal protein L28;Ribosomal	RPL28;hCG_38234	60S ribosomal protein L28 isoform 3;60S ribosomal pro	B4DEP9;P46779;C	19.148	0.010927	0.34496	0.41334		1	-1.2745991
[Acyl-carrier-protein] S-acetyltransferase;[Acyl-carrier-protein] S-malonyltransferase;3-hydroxypaln	FAS;FASN	Fatty acid synthase	P49327;Q13587;C	273.42	4.68E-19	0.45552	0.40696	115.46	2	-1.2970411
Helix-destabilizing protein;Heterogeneous nuclear ribonucleoprotein A1;hnRNP core protein A1;Sir	HNRNPA1;HNRPA1;HNRPA1L3;HNRNPA1L;H	Isoform A1-B of Heterogeneous nuclear ribonucleopro	P09651-1;P09651	38.746	5.91E-166	0.51734	0.40337	101.02	64	-1.3098243
APOBEC1-binding protein 1;Heterogeneous nuclear ribonucleoprotein A/B;cDNA FLJ60713, highly	ABBP1;HNRNPAB;HNRPAB	Isoform 3 of Heterogeneous nuclear ribonucleoprotein	Q99729-3;Q9972	30.588	3.70E-66	0.31851	0.39065	149.22	17	-1.3560515
Epidermal Langerhans cell protein LCP1;TOX high mobility group box family member 4;cDNA FLJ54	C14orf92;KIAA0737;TOX4	TOX high mobility group box family member 4;cDNA FLJ	O94842;B4DPY8;E	66.194	0.0051205	0.30792	0.38212		1	-1.3879023
Autoantigen p542;Heterogeneous nuclear ribonucleoprotein C-like 2;hnRNP associated with lethal	HNRPCL2;P542;RALY;hCG_37136;RP1-64K7.	cDNA FLJ77422, highly similar to Homo sapiens RNA bir	Q9UKM9-1;Q9UK	32.55	0.0067281	0.31645	0.38023		1	-1.3950557
DNase IV;Flap endonuclease 1;Flap structure-specific endonuclease 1;Maturation factor 1;cDNA FL	FEN1;RAD2;hCG_40848	Flap endonuclease 1	P39748;B4DWZ4;	42.592	1.13E-197	0.35446	0.36909	134.47	46	-1.4379554
Chromatin-specific transcription elongation factor 80 kDa subunit;Facilitates chromatin transcriptic	FACT80;SSRP1	FACT complex subunit SSRP1	Q08945	81.074	6.74E-05	0.3076	0.36887	20.259	2	-1.4388156
Natural killer cell-enhancing factor A;Peroxiredoxin-1;Proliferation-associated gene protein;Thiorec	PAGA;PAGB;PRDX1;TDPX2	Peroxiredoxin-1;19 kDa protein;11 kDa protein	Q06830;B2R4P2	22.11	1.05E-07	0.46435	0.35447	162.32	2	-1.4962646
Cyclophilin A;Cyclosporin A-binding protein;Peptidyl-prolyl cis-trans isomerase A;Rotamase A;Pepti	CYPA;PPIA	Peptidyl-prolyl cis-trans isomerase A;cDNA FLJ53060, m	P62937;A8K220;A	18.012	1.29E-10	0.35069	0.3527	157.57	2	-1.5034865
Death-associated transcription factor 1;Death-inducer obliterator 1	C20orf158;DATF1;DIDO1;KIAA0333	Isoform 4 of Death-inducer obliterator 1	Q9BTC0-4;Q9BTC	243.87	2.03E-08	0.29722	0.34691		1	-1.5273667
PWP1-interacting protein 17;Single-stranded DNA-binding protein, mitochondrial;cDNA, FLJ93504,	SSBP;SSBP1;hCG_2014251;tcag7.401	Single-stranded DNA-binding protein, mitochondrial;cD	Q04837;A4D1U3;	17.259	3.13E-162	0.4605	0.34642	100.52	24	-1.5294059
Glutathione S-transferase kappa 1;Glutathione S-transferase subunit 13;GST 13-13;GST class-kappa	GSTK1;HDCMD47P;hCG_20682;LOC51064;t	Isoform 2 of Glutathione S-transferase kappa 1;Isoform	Q9Y2Q3-2;Q9Y2Q	31.566	1.07E-12	0.28612	0.34453		1	-1.5372985
Long-chain acyl-CoA synthetase 3;Long-chain-fatty-acid--CoA ligase 3;cDNA, FLJ95462, highly simil	ACS3;ACSL3;FACL3;LACS3;ACS4;ACSL4;FACL	Long-chain-fatty-acid--CoA ligase 3;Isoform Long of Lon	O95573;B2RBE0;E	80.419	0.001214	0.27122	0.32588	114.37	2	-1.6175873
cDNA FLJ32471 fis, clone SKNMC2000322, highly similar to Peptidyl-tRNA hydrolase 2, mitochondr	BIT1;CGI-147;PTH2;PTRH2	cDNA FLJ32471 fis, clone SKNMC2000322, highly simila	Q96ME4;Q9Y3E5	19.325	0.018848	0.26459	0.31638		1	-1.6602697
Cleavage and polyadenylation specificity factor 25 kDa subunit;Cleavage and polyadenylation speci	CFIM25;CPSF25;CPSF5;NUDT21;DKFZp313O	Cleavage and polyadenylation specificity factor subunit	O43809;B2R6U8;	26.227	2.64E-09	0.26189	0.31496	203	3	-1.6667595
Eukaryotic translation initiation factor 2 subunit 2;Eukaryotic translation initiation factor 2 subunit	EIF2B;EIF2S2;hCG_2018881	Eukaryotic translation initiation factor 2 subunit 2;Euka	P20042;B5BU01;C	38.388	8.66E-33	0.31223	0.31474	54.43	3	-1.6677676
ATP-dependent DNA helicase Q1;DNA helicase, RecQ-like type 1;DNA-dependent ATPase Q1;RecQ	RECQ1;RECQL;RECQL1	ATP-dependent DNA helicase Q1;73 kDa protein	P46063	73.457	8.04E-104	0.27272	0.30991	119.84	38	-1.6900788
Sb1.8;Structural maintenance of chromosomes protein 1A;cDNA FLJ77162, highly similar to Homo	DXS423E;KIAA0178;SB1.8;SMC1;SMC1A;SM	Structural maintenance of chromosomes protein 1A;c	Q14683;A8K7A6;	143.23	0.0031608	0.2449	0.30716	77.189	3	-1.7029377
BRG1-associated factor 170;SWI/SNF complex 170 kDa subunit;SWI/SNF complex subunit SMARCC	BAF170;SMARCC2;BAF155;SMARCC1	Isoform 1 of SWI/SNF complex subunit SMARCC2;Isofor	Q8TAQ2-1;Q8TAQ	132.88	1.33E-09	0.26332	0.30397	51.398	3	-1.7179991
60S acidic ribosomal protein P0;60S ribosomal protein L10E;cDNA FLJ75549, highly similar to Hom	RPLP0	60S acidic ribosomal protein P0;60S acidic ribosomal pr	P05388;A8K4Z4;Q	34.273	3.82E-05	0.25213	0.30304	87.084	2	-1.7224199
Activating transcription factor 1;Cyclic AMP-dependent transcription factor ATF-1;Protein TREB36;	ATF1;CREM;CREB1;hCG_15208;hCREM-1;hC	Cyclic AMP-dependent transcription factor ATF-1;Isofor	P18846;B2R8K4;Q	29.232	0.0024591	0.26584	0.29415	154.01	2	-1.7653761
Caspase recruitment domain-containing protein 6;Caspase recruitment domain family, member 6	CARD6	Caspase recruitment domain-containing protein 6	Q9BX69;Q59EG7	116.47	0.0048408	0.38958	0.29064	250.25	3	-1.7826948
40S ribosomal protein S25	RPS25	40S ribosomal protein S25	P62851	13.742	2.76E-08	0.29876	0.28709	139.47	6	-1.800425
22 kDa neuronal tissue-enriched acidic protein;Brain acid soluble protein 1;Neuronal axonal memb	BASP1;NAP22	Isoform 1 of Brain acid soluble protein 1;Isoform 2 of B	P80723-1;P80723	22.693	0.018686	0.23749	0.28703		1	-1.8007266
86 kDa subunit of Ku antigen;ATP-dependent DNA helicase 2 subunit 2;ATP-dependent DNA helica	G22P2;XRCC5	X-ray repair cross-complementing protein 5	P13010;Q53T09;C	82.704	8.04E-41	0.25111	0.28687	115.83	19	-1.801531
Ribosomal protein S10;40S ribosomal protein S10;Ribosomal protein S10 variant	RP11-375E1__A.4-005;RPS10	Ribosomal protein S10;40S ribosomal protein S10;15 k	Q5TZB9;P46783;C	19.868	0.00026313	0.23271	0.2786	100.76	7	-1.8437328
cDNA FLJ61295, highly similar to Gamma-interferon-inducible protein Ifi-16;Gamma-interferon-ind	IFI16;IFNGIP1;hCG_1995098	cDNA FLJ61295, highly similar to Gamma-interferon-ind	B4DJT8;Q16666-2	82.875	6.34E-33	0.22553	0.27685	72.553	7	-1.8528236
Heterogeneous nuclear ribonucleoprotein R;cDNA, FLJ93632, highly similar to Homo sapiens heter	HNRNPR;HNRPR;hCG_38907;DKFZp686A13	71 kDa protein;Isoform 2 of Heterogeneous nuclear rib	O43390-2;O4339	71.313	6.23E-62	0.23732	0.26451	95.313	12	-1.9186058
50 kDa nuclear polyadenylated RNA-binding protein;Bruno-like protein 2;CUG triplet repeat RNA-b	BRUNOL2;CELF1;CUGBP;CUGBP1;NAB50	Isoform 4 of CUGBP Elav-like family member 1;Isoform	Q92879-4;Q9287	55	5.03E-06	0.25981	0.26393	149.61	4	-1.9217727
70 kDa subunit of Ku antigen;ATP-dependent DNA helicase 2 subunit 1;ATP-dependent DNA helica	G22P1;XRCC6;CTA-216E10.7-002;hCG_2013	X-ray repair cross-complementing protein 6;cDNA FLJ31	P12956;B2RDN9;E	69.842	8.75E-58	0.22931	0.2613	86.259	16	-1.936221
Nucleolin;Protein C23;cDNA FLJ10452 fis, clone NT2RP1000966, highly similar to NUCLEOLIN;Nucle	NCL;hCG_33980	Nucleolin;cDNA FLJ45706 fis, clone FEBRA2028457, hig	P19338;B3KM80;E	76.613	1.22E-48	0.22633	0.26042	90.301	15	-1.9410878
Dr1-associated corepressor;Dr1-associated protein 1;Negative co-factor 2-alpha;Putative uncharac	DRAP1	Uncharacterized protein;Isoform 1 of Dr1-associated co	Q14919-2;Q1491	23.205	0.061773	0.20173	0.24156		1	-2.0495465
Pre-rRNA-processing protein TSR1 homolog	KIAA1401;TSR1	Pre-rRNA-processing protein TSR1 homolog	Q2NL82	91.809	1.63E-08	0.23616	0.23375	168.79	2	-2.0969617
NAD(+) ADP-ribosyltransferase 1;Poly [ADP-ribose] polymerase 1;Poly[ADP-ribose] synthase 1;cDN	ADPRT;PARP1;PPOL	Poly [ADP-ribose] polymerase 1	P09874;B2R5W3;	113.08	0	0.19651	0.23088	111.85	210	-2.1147849
Macrophage myristoylated alanine-rich C kinase substrate;MARCKS-like protein 1;MARCKS-related	MARCKSL1;MLP;MRP	similar to hCG2040253;similar to hCG2040253;MARCKS	P49006	22.922	0.02024	0.18989	0.22705		1	-2.1389181
PAI1 RNA-binding protein 1;Plasminogen activator inhibitor 1 RNA-binding protein;SERPINE1 mRN	CGI-55;PAIRBP1;SERBP1;hCG_2032021;DKF	SERPINE1 mRNA binding protein 1, isoform CRA_d;Isof	Q8NC51-1;Q8NC5	50.92	7.01E-119	0.17971	0.2172	101.77	19	-2.202904
Protein DEK;cDNA FLJ51914, highly similar to Protein DEK;cDNA FLJ53031, highly similar to Protein	DEK	Protein DEK;cDNA FLJ53031, highly similar to Protein D	P35659;B4DNW3;	42.674	2.16E-44	0.18542	0.20997	85.923	13	-2.2517449
Cold shock domain-containing protein A;DNA-binding protein A;Single-strand DNA-binding protein	CSDA;DBPA	Isoform 1 of DNA-binding protein A;Isoform 2 of DNA-b	P16989-1;P16989	40.089	3.49E-65	0.20746	0.20964	81.159	5	-2.2540141
Desmoyokin;Neuroblast differentiation-associated protein AHNAK;cDNA FLJ57801, highly similar t	AHNAK;PM227	Neuroblast differentiation-associated protein AHNAK;U	Q09666;B4DTV0;	629.09	4.81E-30	0.1841	0.20913	119.93	9	-2.2575281
Replication factor A protein 1;Replication protein A 70 kDa DNA-binding subunit;Single-stranded D	REPA1;RPA1;RPA70	Replication protein A 70 kDa DNA-binding subunit	P27694	68.137	1.21E-76	0.1934	0.20825	112.7	11	-2.2636116
Cingulin-like protein 1;Junction-associated coiled-coil protein;Paracingulin;CGNL1 protein	CGNL1;JACOP;KIAA1749	Isoform 1 of Cingulin-like protein 1	Q0VF96-1;Q0VF9	149.04	0.0086443	0.18384	0.20494		1	-2.2867265
Graf-related protein 2;GTPase regulator associated with focal adhesion kinase 2;Rho GTPase-activa	ARHGAP10;GRAF2;FLJ20896;DKFZp667D142	Rho GTPase-activating protein 10;Protein;cDNA FLJ410	A1A4S6;B4E2W2;	89.374	0.27104	0.18355	0.20371		1	-2.2954113
Serine/threonine-protein kinase VRK1;Vaccinia-related kinase 1;cDNA FLJ75441, highly similar to H	VRK1	Serine/threonine-protein kinase VRK1	Q99986;A8K245	45.476	0.0046857	0.15786	0.18877	44.964	2	-2.4052986
Cysteine and glycine-rich protein 1;Cysteine-rich protein 1;cDNA FLJ77815, highly similar to Homo	CSRP;CSRP1;CYRP;hCG_17679;DKFZp686M1	Cysteine and glycine-rich protein 1	P21291;A8K268;B	20.567	6.17E-12	0.15531	0.18511	104.79	2	-2.4335453
CLL-associated antigen KW-7;Dense fine speckles 70 kDa protein;Lens epithelium-derived growth f	DFS70;LEDGF;PSIP1;PSIP2;hCG_27254	Isoform 1 of PC4 and SFRS1-interacting protein;Isoform	O75475-1;O7547	60.103	6.34E-94	0.14444	0.18091	96.323	24	-2.4666559
DNA topoisomerase 1;DNA topoisomerase I;Topoisomerase (DNA) I;TOP1 protein;Putative unchara	TOP1	DNA topoisomerase 1	P11387;B9EG90;C	90.725	1.66E-21	0.1654	0.17977	86.761	7	-2.4757758
ADA2-like protein beta;Transcriptional adapter 2-beta;cDNA FLJ45019 fis, clone BRAWH3015825	ADA2B;TADA2B	Isoform 1 of Transcriptional adapter 2-beta;Isoform 2 o	Q86TJ2-1;Q86TJ2;	48.47	0.021311	0.14232	0.17201		1	-2.5394357

U2 small nuclear ribonucleoprotein B'';Small nuclear ribonucleoprotein polypeptide B'';Similar to s	SNRPB2	U2 small nuclear ribonucleoprotein B''	P08579;B5BTZ8;Q	25.486	9.66E-05	0.14288	0.17136		1	-2.5448977
Histone H4	H4/A;H4/B;H4/C;H4/D;H4/E;H4/G;H4/H;H4/	Histone H4	P62805;B2R4R0;C	11.367	8.02E-22	0.16205	0.15736	110.36	7	-2.6678592
Cytokeratin-6B;Keratin, type II cytoskeletal 6B;Keratin-6B;Type-II keratin Kb10	K6B;KRT6B;KRTL1	Keratin, type II cytoskeletal 6B	P04259	60.066	1.08E-64	0.15036	0.14475	93.795	4	-2.7883647
60S ribosomal protein L24;60S ribosomal protein L30;Putative uncharacterized protein RPL24;Ribo	RPL24;hCG_2023003	19 kDa protein;60S ribosomal protein L24;Uncharacteri	P83731;C9JNW5;C	19.41	0.00053849	0.12527	0.14459	91.496	4	-2.7899603
Replication factor A protein 2;Replication protein A 32 kDa subunit;Replication protein A 34 kDa su	REPA2;RPA2;RPA32;RPA34;RP4-547C9.3-003	Isoform 3 of Replication protein A 32 kDa subunit;Isofor	P15927-3;P15927	38.809	0.00070755	0.11557	0.13309	100.75	3	-2.9095259
High mobility group protein 2;High mobility group protein B2;High-mobility group box 2	HMG2;HMGB2	High mobility group protein B2;Uncharacterized protein	P26583;Q5U071;D	24.033	1.17E-28	0.10965	0.12993	147.88	15	-2.9441935
High mobility group protein 1;High mobility group protein B1;High-mobility group box 1;cDNA FLJ5	HMG1;HMGB1;RP11-550P23.1-002;RP11-55	High mobility group protein B1;High-mobility group box	P09429;Q5T7C6;B	24.893	3.53E-40	0.11702	0.12095	70.795	16	-3.0475173
Elongation factor 1-alpha 1;Elongation factor Tu;Eukaryotic elongation factor 1 A-1;Leukocyte recep	EEF1A;EEF1A1;EF1A;LENG7;EEF1A1L14;PTI-1	Elongation factor 1-alpha 1;Putative elongation factor 1	P68104;A8K9C4;A	50.14	3.43E-22	0.10652	0.11468	116.64	29	-3.1243143
Lipocalin-1;Tear lipocalin;Tear prealbumin;Von Ebner gland protein;Putative lipocalin 1-like protein	LCN1;VEGP;LCN1L1	Lipocalin-1;Putative lipocalin 1-like protein 1	P31025;Q5VSP4	19.25	0.021183	0.12791	0.11386		1	-3.1346671
AU-rich element RNA-binding protein 1;Heterogeneous nuclear ribonucleoprotein D0;cDNA FLJ541	AUF1;HNRNPD;HNRPD	Isoform 1 of Heterogeneous nuclear ribonucleoprotein	Q14103-1;Q14103	38.434	1.23E-120	0.10669	0.11266	93.758	61	-3.1499527
Putative uncharacterized protein RAN;RAN, member RAS oncogene family, isoform CRA_c;Androge	hCG_1744585;RAN;ARA24;OK/SW-cl.81	26 kDa protein;27 kDa protein;Uncharacterized protein;	B5MDF5;P62826;A	26.409	1.96E-05	0.089823	0.10838		1	-3.2058295
Actin, aortic smooth muscle;Alpha-actin-2;Cell growth-inhibiting gene 46 protein;cDNA FLJ32030 fi	ACTA2;ACTSA;ACTVS;GIG46;hCG_22984;ACT	Actin, aortic smooth muscle;Actin, alpha skeletal muscle	P62736;B3KPP5;B	42.009	2.06E-111	0.099511	0.1052	93.833	4	-3.2487934
cDNA FLJ75516, highly similar to Xenopus tropicalis ubiquitin C, mRNA;Ubiquitin C variant;Ubiquiti	UBC;DKFZp434K0435;hCG_1998947;UBB;RP	Polyubiquitin-C;39 kDa protein;UBC protein;33 kDa prot	P0CG48;A8K674;C	77.028	2.03E-18	0.087026	0.10488	126.87	3	-3.2531885
Glyceraldehyde-3-phosphate dehydrogenase, testis-specific;Spermatogenic cell-specific glyceraldel	GAPD2;GAPDH2;GAPDHS;GAPDS;HSD35;HSI	Glyceraldehyde-3-phosphate dehydrogenase, testis-spe	O14556	44.501	6.21E-05	0.086386	0.10397		1	-3.2657608
Developmentally-regulated GTP-binding protein 1;Neural precursor cell expressed developmentally	DRG1;NEDD3;DKFZp434N1827	Developmentally-regulated GTP-binding protein 1;25 kD	Q9Y295;Q9UFA5	40.542	0.019484	0.087837	0.10268		1	-3.2837729
Citrate synthase, mitochondrial;Citrate synthase	CS	Citrate synthase, mitochondrial;48 kDa protein;cDNA FL	O75390;B4DJV2;B	51.712	9.84E-11	0.11006	0.089142	175.21	5	-3.4877509
60S ribosomal protein L11;CLL-associated antigen KW-12;Cell growth-inhibiting protein 34;cDNA, F	RPL11;hCG_1739238;RP11-223J15.3-001;RP	Isoform 1 of 60S ribosomal protein L11;Isoform 2 of 60S	P62913-1;P62913	20.252	3.55E-29	0.066682	0.080799	199.74	3	-3.6295188
Histone H1.3;Histone H1c;Histone H1.4;Histone H1b;Liver histone H1e;Histone 1, H1e;Histone H1e	H1F3;HIST1H1D;H1F4;HIST1H1E;hCG_16457	Histone H1.3;Histone H1.4;Histone H1.2;Histone H1t;Hi	P16402;P10412;A	22.35	5.79E-15	0.063144	0.080785	24.206	5	-3.6297687
Enaptin;Myocyte nuclear envelope protein 1;Nesprin-1;Nuclear envelope spectrin repeat protein 1	C6orf98;KIAA0796;KIAA1262;KIAA1756;MYN	Isoform 1 of Nesprin-1;1011 kDa protein;Isoform 6 of N	Q8NF91-1;Q8NF9	1011	0.0018652	0.056461	0.071808	81.492	3	-3.7997116
High mobility group protein 2a;High mobility group protein 4;High mobility group protein B3;Non-h	HMG2A;HMG4;HMGB3	similar to high mobility group box 3;similar to high mob	O15347;Q16466	23.744	3.68E-24	0.054903	0.070181	27.352	3	-3.8327757
Dermcidin isoform 2;DCD-1;Dermcidin;Preproteolysin;Survival-promoting peptide;Proteolysis indu	DCD;AIDD;DSEP;hCG_1820620;PIF	Dermcidin isoform 2;Dermcidin	A5JHP3;P81605;Q	12.414	0.0010397	0.086486	0.068882	26.893	2	-3.8597292
Eukaryotic translation initiation factor 4B;cDNA FLJ54492, highly similar to Eukaryotic translation in	EIF4B	cDNA FLJ54492, highly similar to Eukaryotic translation	P23588;B4DRM3;I	69.725	0.0041245	0.098923	0.064531	51.957	2	-3.9538638
High mobility group nucleosome-binding domain-containing protein 1;Non-histone chromosomal p	HMG14;HMGN1;hCG_17955	Non-histone chromosomal protein HMG-14;Uncharacte	P05114;Q3B790;A	10.659	7.85E-32	0.049714	0.060085	22.375	5	-4.0568513
Putative uncharacterized protein SSR1;cDNA, FLJ93042, highly similar to Homo sapiens signal sequ	SSR1;PSEC0262;TRAPA	Uncharacterized protein;cDNA, FLJ93042, highly similar	C9IZQ1;B2R6N9;P	33.887	1.62E-30	0.043967	0.053128	178.33	3	-4.2343838
		12 kDa protein		12.457	0.013944	0.038223	0.048091	30.93	3	-4.3780893
High mobility group nucleosome-binding domain-containing protein 3;Thyroid receptor-interacting	HMGN3;PNAS-24;TRIP7	Isoform 1 of High mobility group nucleosome-binding d	Q15651-1;Q15651	10.666	1.37E-33	0.039915	0.046605	287.08	4	-4.4233714
Beta-actin-like protein 2;Kappa-actin	ACTBL2	Beta-actin-like protein 2	Q562R1	42.003	2.82E-43	0.042056	0.046441	26.557	2	-4.4284572
Kinesin-like protein KIF14	KIAA0042;KIF14	Kinesin-like protein KIF14	Q15058	186.49	0.025655	0.028197	0.038309	55.483	4	-4.7061728
Pyridoxal kinase;Pyridoxine kinase;HCG401289, isoform CRA_b;Putative uncharacterized protein PI	C21orf124;C21orf97;PDXK;PKH;PNK;PRED79	Isoform 1 of Pyridoxal kinase;Isoform 2 of Pyridoxal kina	O00764-1;O00764	35.102	0.015446	0.048761	0.037149		1	-4.7505328
Zinc finger protein 646;cDNA FLJ61089, highly similar to Zinc finger protein 646	KIAA0296;ZNF646	Isoform 2 of Zinc finger protein 646;Isoform 1 of Zinc fir	O15015-2;O15015	200.82	0.080539	0.025293	0.020219		1	-5.6281445
Cancer/testis antigen 8;Synaptonemal complex protein 1;CTCL tumor antigen se2-1;SYCP1 protein	SCP1;SYCP1;RP11-109G4.1-003	Synaptonemal complex protein 1;Synaptonemal comple	Q15431;B7ZLS9;Q	114.19	0.021859	0.024181	0.017982		1	-5.7973027
cDNA FLJ52003, highly similar to Homo sapiens nucleoredoxin (NXN), mRNA		cDNA FLJ52003, highly similar to Homo sapiens nucleor	B4DNN6	14.233	0.022064	0.013346	0.017225	147.05	3	-5.8593522
Dystrophin;DMD protein;Duchenne muscular dystrophy gene;Duchenne muscular dystrophy prote	DMD;GS1-19O24.1-006;GS1-19O24.1-002;G	Isoform 4 of Dystrophin;dystrophin Dp427p1 isoform;Is	P11532-1;P11532	426.74	1.34E-05	0.020134	0.015366	49.896	5	-6.0241145
Androgen receptor coactivator 160 kDa protein;Androgen receptor-associated protein of 160 kDa;T	ARA160;TMF1	Isoform 2 of TATA element modulatory factor;Isoform 1	P82094-2;P82094	123.15	0.020868	0.0036165	0.0038854	73.411	2	-8.0077212
Cysteine and glycine-rich protein 2;Cysteine-rich protein 2;LIM domain only protein 5;Smooth mus	CSRP2;LMO5;SMLIM	Cysteine and glycine-rich protein 2;18 kDa protein;12 kD	Q16527	20.954	1.99E-06				0	
Ras-related protein Rab-15;Putative uncharacterized protein DKFZp686J06205;cDNA FLJ53028, hig	RAB15;DKFZp686J06205;RAB8B;MEL;RAB8;I	Isoform 1 of Ras-related protein Rab-15;Isoform 2 of Ra	P59190-1;P59190	24.39	0.0052976				0	
Actin-like protein 3;Actin-related protein 3;cDNA FLJ51082, highly similar to Actin-like protein 3;cD	ACTR3;ARP3;hCG_26669;ACTR3B;ARP11;AR	Actin-related protein 3;Isoform 1 of Actin-related protei	P61158;B4DT29;B	47.371	0.068854				0	
Insulin-like growth factor-binding protein 3;Insulin-like growth factor binding protein 3;Insulin-like g	IBP3;IGFBP3;hCG_1735376	insulin-like growth factor binding protein 3 isoform a pr	P17936;A6XND0;A	32.222	0.0010814				0	
1,4-beta-N-acetylmuramidase C;Lysozyme C	LYZ;LZM;hCG_24462	Lysozyme C	P61626;B2R4C5	16.537	5.80E-48				0	
Protein NICE-4;Ubiquitin-associated protein 2-like;cDNA FLJ55671, highly similar to Ubiquitin-assoc	KIAA0144;NICE4;UBAP2L;RP11-205M9.4-00	Isoform 1 of Ubiquitin-associated protein 2-like;Isoform	Q14157-2;Q14157	114.53	0.0080299				0	
Differentiation-dependent protein A4;Intestinal membrane A4 protein;Proteolipid protein 2	A4;PLP2	Proteolipid protein 2	Q04941	16.691	1.41E-08				0	
Regulator of differentiation 1;ROD1 regulator of differentiation 1 (S. pombe);cDNA FLJ51619, highl	ROD1;RP11-165N19.1-001;RP11-165N19.1-(	Isoform 4 of Regulator of differentiation 1;Isoform 5 of I	O95758-4;O95758	60.417	7.33E-17				0	
A18 hnRNP;Cold-inducible RNA-binding protein;Glycine-rich RNA-binding protein CIRP;Cold inducit	A18HNRNP;CIRBP;CIRP;hCG_21575	Cold-inducible RNA-binding protein	Q14011;Q53XX5	18.648	3.73E-21				0	
Outer mitochondrial membrane protein porin 1;Plasmalemmal porin;Porin 31HL;Porin 31HM;Volta	VDAC;VDAC1	Voltage-dependent anion-selective channel protein 1;Si	P21796;B3KTS5;B	30.772	0.013387				0	
Chromobox protein homolog 3;HECH;Heterochromatin protein 1 homolog gamma;Modifier 2 prote	CBX3;CCDC32;hCG_1745364;hCG_1999239;	Chromobox protein homolog 3	Q13185;A4D177	20.811	6.01E-06				0	
eIF3 p110;eIF3 p116;eIF-3-eta;Eukaryotic translation initiation factor 3 subunit 9;Eukaryotic transla	EIF3B;EIF3S9;hCG_1998988;tcag7.544	Isoform 2 of Eukaryotic translation initiation factor 3 sul	P55884-2;P55884	99.028	0.010084				0	
60S ribosomal protein L14;CAG-ISL 7;cDNA FLJ76611, highly similar to Homo sapiens ribosomal prc	RPL14	Ribosomal protein L14 variant;60S ribosomal protein L1	P50914;A8K3Q9;A	23.787	6.29E-07				0	
		Ankyrin repeat containing protein		19.544	0.017765				0	
